# Mechanisms of GII.4 Norovirus Persistence in Human Populations 

**DOI:** 10.1371/journal.pmed.0050031

**Published:** 2008-02-12

**Authors:** Lisa C Lindesmith, Eric F Donaldson, Anna D LoBue, Jennifer L Cannon, Du-Ping Zheng, Jan Vinje, Ralph S Baric

**Affiliations:** 1 University of North Carolina-Chapel Hill, Chapel Hill, North Carolina, United States of America; 2 Centers for Disease Control and Prevention, Atlanta, Georgia, United States of America; Imperial College London, United Kingdom

## Abstract

**Background:**

Noroviruses are the leading cause of viral acute gastroenteritis in humans, noted for causing epidemic outbreaks in communities, the military, cruise ships, hospitals, and assisted living communities. The evolutionary mechanisms governing the persistence and emergence of new norovirus strains in human populations are unknown. Primarily organized by sequence homology into two major human genogroups defined by multiple genoclusters, the majority of norovirus outbreaks are caused by viruses from the GII.4 genocluster, which was first recognized as the major epidemic strain in the mid-1990s. Previous studies by our laboratory and others indicate that some noroviruses readily infect individuals who carry a gene encoding a functional alpha-1,2-fucosyltransferase *(FUT2)* and are designated “secretor-positive” to indicate that they express ABH histo-blood group antigens (HBGAs), a highly heterogeneous group of related carbohydrates on mucosal surfaces. Individuals with defects in the *FUT2* gene are termed secretor-negative, do not express the appropriate HBGA necessary for docking, and are resistant to Norwalk infection. These data argue that *FUT2* and other genes encoding enzymes that regulate processing of the HBGA carbohydrates function as susceptibility alleles. However, secretor-negative individuals can be infected with other norovirus strains, and reinfection with the GII.4 strains is common in human populations. In this article, we analyze molecular mechanisms governing GII.4 epidemiology, susceptibility, and persistence in human populations.

**Methods and Findings:**

Phylogenetic analyses of the GII.4 capsid sequences suggested an epochal evolution over the last 20 y with periods of stasis followed by rapid evolution of novel epidemic strains. The epidemic strains show a linear relationship in time, whereby serial replacements emerge from the previous cluster. Five major evolutionary clusters were identified, and representative ORF2 capsid genes for each cluster were expressed as virus-like particles (VLPs). Using salivary and carbohydrate-binding assays, we showed that GII.4 VLP-carbohydrate ligand binding patterns have changed over time and include carbohydrates regulated by the human *FUT2* and *FUT3* pathways, suggesting that strain sensitivity to human susceptibility alleles will vary. Variation in surface-exposed residues and in residues that surround the fucose ligand interaction domain suggests that antigenic drift may promote GII.4 persistence in human populations. Evidence supporting antigenic drift was obtained by measuring the antigenic relatedness of GII.4 VLPs using murine and human sera and demonstrating strain-specific serologic and carbohydrate-binding blockade responses. These data suggest that the GII.4 noroviruses persist by altering their HBGA carbohydrate-binding targets over time, which not only allows for escape from highly penetrant host susceptibility alleles, but simultaneously allows for immune-driven selection in the receptor-binding region to facilitate escape from protective herd immunity.

**Conclusions:**

Our data suggest that the surface-exposed carbohydrate ligand binding domain in the norovirus capsid is under heavy immune selection and likely evolves by antigenic drift in the face of human herd immunity. Variation in the capsid carbohydrate-binding domain is tolerated because of the large repertoire of similar, yet distinct HBGA carbohydrate receptors available on mucosal surfaces that could interface with the remodeled architecture of the capsid ligand-binding pocket. The continuing evolution of new replacement strains suggests that, as with influenza viruses, vaccines could be targeted that protect against norovirus infections, and that continued epidemiologic surveillance and reformulations of norovirus vaccines will be essential in the control of future outbreaks.

## Abstract 2

Through phylogenetic analysis of norovirus isolates, Ralph Baric and colleagues show that new epidemic strains arise as the variety of available cellular receptors permits antigenic drift in the viral capsid.

## Editors' Summary

**Background.:**

Noroviruses are the leading cause of viral gastroenteritis (stomach flu), the symptoms of which include nausea, vomiting, and diarrhea. There is no treatment for infection with these highly contagious viruses. While most people recover within a few days, the very young and old may experience severe disease. Like influenza, large outbreaks (epidemics) of norovirus infection occur periodically (often in closed communities such as cruise ships), and most people have several norovirus infections during their lifetime. Currently, 100,000–200,000 people are being infected each week in England with a new GII.4 variant. There are several reasons for this pattern of infection and reinfection. First, the immune response induced by a norovirus infection is short-lived in some people, but not all. Second, there are many different noroviruses. Based on their genomes (genetic blueprints), noroviruses belong to five “genogroups,” which are further subdivided into “genotypes.” An immune response to one norovirus provides little protection against noroviruses of other genogroups or genotypes. Third, like influenza viruses, noroviruses frequently acquire small changes in their genome. This process is called antigenic drift (antigens are the molecules on the surface of infectious agents that stimulate the production of antibodies, proteins that help the immune system recognize and deal with foreign invaders). Norovirus epidemics occur when virus variants emerge to which the human population has no immunity.

**Why Was This Study Done?:**

It is unknown exactly how noroviruses change over time or how they persist in human populations. In addition, little is known about susceptibility to norovirus infections except that secretor-positive individuals—people who express “histoblood group antigens” (HBGAs, a heterogeneous group of sugar molecules by which noroviruses attach themselves to human cells) on the cells that line their mouths and guts—are more susceptible than secretor-negative people, who express these antigens only on red blood cells. Information of this sort is needed to devise effective intervention strategies, therapies, and vaccines to reduce the illness and economic costs associated with norovirus outbreaks. In this study, the researchers investigate the molecular mechanisms governing the emergence and persistence of epidemic norovirus strains in human populations by analyzing how GII.4 norovirus strains (the genotype usually associated with epidemics) have changed over time.

**What Did the Researchers Do and Find?:**

The researchers analyzed the relationships among the sequences of the gene encoding the capsid protein of GII.4 norovirus strains isolated over the past 20 years. The capsid protein forms a shell around noroviruses and is involved in their binding to HBGAs and their recognition by the human immune system. The researchers found that the virus evolved in fits and starts. That is, for several years, one cluster of strains was predominant but then new epidemic strains emerged rapidly from the cluster. In all, the researchers identified five major evolutionary clusters. They then created “virus-like particles” (VLPs) using representative capsid genes from each cluster and showed that these VLPs bound to different HBGAs. Finally they measured the antigenic relatedness of the different VLPs using human blood collected during a 1988 GII.4 outbreak. Antibodies in these samples recognized the VLPs representing early GII.4 strains better than VLPs representing recent GII.4 strains. The ability of the blood samples to block the interaction of VLPs with their matching HBGAs showed a similar pattern.

**What Do These Findings Mean?:**

These findings suggest that the part of the norovirus capsid protein that binds to sugars on host cells is under heavy immune selection and evolves over time by antigenic drift. They show that, like influenza viruses, GII.4 viruses evolve through serial changes in the capsid sequence that occur sporadically after periods of stability, probably to evade the build up of immunity within the human population. Variation in this region of the viral genome is possible because human populations express a great variety of HBGA molecules so there is always likely to be a subpopulation of people that is susceptible to the altered virus. Overall, these findings suggest that it should be possible to develop vaccines to protect against norovirus infections but, just as with influenza virus, surveillance systems will have to monitor how the virus is changing and vaccines will need to be reformulated frequently to provide effective protection against norovirus outbreaks.

**Additional Information.:**

Please access these Web sites via the online version of this summary at http://dx.doi.org/10.1371/journal.pmed.0050031.

- See a related *PLoS Medicine* Perspective article
- The MedlinePlus encyclopedia has a page on viral gastroenteritis (in English and Spanish)
- The US Centers for Disease Control and Prevention provides information on viral gastroenteritis (in English and Spanish) and on noroviruses
- The UK National Health Service's health website (NHS Direct) provides information about noroviruses
- The UK Health Protection Agency and the US Food & Drug Administration also provide information about noroviruses

## Introduction

Globally, noroviruses are the second most important cause of severe viral gastroenteritis in young children, cause 20% of endemic diarrheal disease in families, cause traveler's diarrhea in all ages, and are especially virulent in the elderly, as evidenced by recent reports of 19 deaths associated with norovirus acute gastroenteritis in 2006 in long-term care facilities in the United States [1–4]. In addition to human costs, norovirus infections cause severe economic losses as a single 3 mo hospital outbreak may incur expenses that approach or exceed $650,000 in supplies, staff time off, and closed beds [5]. Although noroviruses were first observed in 1968, the basic principles governing their molecular and evolutionary epidemiology and persistence in human populations are unclear, but are critically important for devising intervention strategies, therapeutics, and vaccines that could minimize the high morbidity, occasional mortality, and extensive economic burdens associated with disease outbreaks.

Noroviruses are approximately 38 nm iscosahedral viruses and contain a 7.5 Kb single-stranded, positive-sense RNA genome that encodes three large open reading frames (ORFs), including the ORF1 replicase polyprotein and the major and minor structural ORFs 2 and 3, respectively. These highly heterogeneous viruses have been genetically grouped into five different genogroups (GI–GV), of which the GI and GII genogroups are further subdivided into more than 25 different genotypes (for example GII.4 is genogroup II genotype 4) and account for the majority of human cases. The ORF2 major capsid protein sequence can diverge by as much as 60% between genogroups and 20%–30% between genotypes within a genogroup. The majority of norovirus outbreaks are caused by the GII.4 genotypes, and pandemic spread was first recognized in the mid-1990s [6]. During 1995–1996, strain US95/96 was responsible for about 55% of the norovirus outbreaks in the US and 85% of the outbreaks in The Netherlands [7]. Between 2000 and 2004, US95/96 was replaced by two new GII.4 variants. In the US, Farmington Hills [8] was ultimately associated with 80% of norovirus acute gastroenteritis outbreaks [9]. Simultaneously in Europe, a new GII.4 variant, GII.4b, emerged and caused outbreaks during the winter, spring, and summer [10–12]. In 2004, the Hunter GII.4 variant was detected in Australia, Europe, and Asia [12–14]. This strain was replaced in early 2006 by two new cocirculating GII.4 variants in the US, Europe, and Asia [4]. One of these was Sakai, which represents a neoteric GII.4 outbreak strain associated with outbreaks in health-care facilities in Southeast Asia [15], although strains that cluster with Sakai have also been identified in the US and The Netherlands. The second 2006 outbreak strain was Minerva, which was identified in the US and is identical to strains identified in The Netherlands [4].

Norwalk virus (NV, a GI.1 virus) readily infects individuals who have the gene that encodes a functional alpha-1,2 fucosyltransferase (FUT2) enzyme that allows expression of histo-blood group antigens (HBGAs) on mucosal surfaces and a secretor-positive phenotype [16]. Individuals who encode defects in the FUT2 enzyme are secretor-negative, do not express the HBGAs necessary for docking and perhaps entry, and are resistant to infection. The association between HBGA expression and norovirus susceptibility has been confirmed for NV and certain other GI and GII strains, including GII.4 [17–21]. However, other enzymes may serve as susceptibility factors as well, since secretor-negative individuals have antibodies against human noroviruses (although at lower titers than secretor-positive individuals [16,17,22]), and develop clinical illness after challenge with Snow Mountain virus (GII.2) [22]. The evolutionary mechanisms governing the persistence and epidemic spread of the GII.4 viruses in human populations are unknown.

Expression of the norovirus ORF2 major capsid protein produces virus-like particles (VLPs) and the NV x-ray crystal structure indicates that dimer formation is required to form the higher-order structure composed of 180 subunits [23]. The monomer has two domains linked by a flexible hinge: the shell domain (S), which forms the inner core, and the protruding domain (P), which forms prominent protrusions extending away from the structure [23] and is subdivided into two subdomains. These include P1 (residues 226–278 and 406–520) and P2, which is the most surface-exposed region of the capsid protein (residues 279–405) [23]. P1 structurally flanks P2.

Mutational analysis of the surface-exposed P2 subdomain supports its role in HBGA binding [24–26], suggesting that it contains the determinants of strain specificity, receptor binding [23,27–30], and potential neutralizing antibody recognition sites [26,31]. More recently, the complex structures of the P domain of a GII.4 virus, VA387, in complex with HBGA trisaccharide A- and B- antigens, revealed a ligand interaction site (site 1) in the P2 subdomain where specific capsid residues form a strong hydrogen bond network with the α-fucose group of the trisaccharide [32]. A second interaction site (site 2) on the VA387 P2 domain was predicted to stabilize binding and enhance ligand affinity by weaker long-distance interactions with the galactose ring (β-gal) of the trisaccharide [32].

In this article, we report the result of our study of the molecular mechanisms governing the emergence and persistence of novel epidemic norovirus strains in human populations.

## Materials and Methods

### Sequences and Sequence Analysis

A total of 167 full-length GII.4 capsid sequences were downloaded from GenBank, six sequences were obtained from our collaborators at the CDC, and three were included from our own in-house collection, for a total of 176 full-length amino acid and nucleotide capsid sequences (Table S1). The amino acid sequences were aligned by ClustalX version 1.83 [33] using the PAM distance matrix and default parameters (Figure S1). A variety of parameters and substitution matrices for the alignment were evaluated using the program TuneClustal version 1.0 (generously provided by Dr. Barry G. Hall) and the PAM series matrix was determined to be the most appropriate, with default gap opening and extension values. The alignment was refined manually, and 211 sites of variation, defined as any site with a quality score of less than 100, were exported in table format and ordered by sequence identity, and then by date of isolation. The sequences were divided into clusters defined as groups of sequences containing a minimum of 98% sequence identity with each other, as determined by the BLASTclust program provided online by the Max Planck Institute (http://toolkit.tuebingen.mpg.de). Clusters were further divided into subclusters, which were defined as a smaller cluster of sequences that shared a minimum of five identical sites that differed from the main cluster. Each cluster and subcluster was ordered by year of isolation. The 211 variable sites were further refined to identify informative sites, such that columns in the table were removed based on the following criteria: (1) columns with single amino acid replacements; (2) columns containing multiple single incongruous amino acid replacements; (3) columns containing random amino acid replacements not associated with a geographic lineage or specific cluster; and (4) lineage specific replacements that were noninformative (Table S2). This refinement reduced the 211 variable sites to 59 informative sites (Table S3). Major clusters were defined as those that contained a minimum of five sequences. One sequence representative of each of the major clusters was selected and aligned along with VA387, which was used as a reference strain.

In addition to the amino acid multiple alignment, the nucleotide sequences were aligned as codons using the program PAL2NAL [34] which aligned the corresponding nucleotide sequences based on the amino acid alignment.

### Positive Selection Analyses

To determine if positive selection occurred during the evolution of the GII.4 strains over the past 20 years, five codon sequences from each of the five major clusters were selected and aligned. The model selection tool of the HyPhy package [35,36] (http://www.hyphy.org/) was used to determine that the Tamura-Nei (TrN) model of evolution was the most appropriate for conducting the analyses, and three different codon-based maximum likelihood methods were used to estimate the dN/dS ratio at every codon position in the alignment to determine if positive selection occurred. These methods included: (1) single-likelihood ancestor counting (SLAC), (2) fixed effects likelihood (FEL), and (3) random effects likelihood (REL).

### Recombination Analysis

In addition, the Genetic Algorithms for Recombination Detection (GARD) and the Single Break-Point (SBP) methods of the HyPhy package were utilized to determine if recombination occurred within the capsid sequences. Further, the Recombination Identification Program (RIP) version 1.9 beta (http://www.hiv.lanl.gov/) was used as an alternative approach to determine if recombination was a factor in capsid sequence evolution.

### Phylogenetic Analyses

Phylogenetic analyses were conducted using two different approaches and by three different strategies. In all cases, amino acid alignments were used to generate trees by both Bayesian inference (BI) of phylogeny using MrBayes version 3.12 [37] and by two maximum parsimony (MP) [38] methods: (1) Molecular Evolutionary Genetics Analysis (MEGA) 4.0 package [39] and (2) Phylogenetic Analysis Using Parsimony (PAUP) version 4.0b10 [40]. For MrBayes, the alignment was exported in the nexus format, the amino acid substitution model was set to Dayhoff [41] using the lset command, and Markov chain Monte Carlo simulation [42–44] was used to approximate the posterior probabilities of trees with sampling conducted on four chains over 500,000 generations [45]. Trees were sampled every 100 generations, and the 5,001 trees collected were summarized with the sumt command set to a burn-in of 1,000, which generated a consensus tree using the 50% majority rule [45]. The burn-in value was determined using the sump command with an arbitrary burn-in of 250, which demonstrated that stationarity occurred prior to the 100,000th generation, indicating that a burn-in of 1,000 was appropriate for the sumt command [45].

For the MP analysis [38] using MEGA 4.0, a bootstrap consensus tree inferred from 100 replicates was generated to represent the evolutionary history of the taxa analyzed [46]. Branches corresponding to partitions reproduced in less than 50% bootstrap replicates were collapsed. The percentage of replicate trees in which the associated taxa clustered together in the bootstrap test (100 replicates) were shown next to the branches [46]. The MP tree was obtained using the close-neighbor-interchange algorithm [47] with search level 3 [46,47] in which the initial trees were obtained with the random addition of sequences (ten replicates). The trees were drawn to scale, with branch lengths calculated using the average pathway method [47] and were displayed in the units of the number of changes over the whole sequence. All positions containing gaps and missing data were eliminated from the dataset.

MP trees were also generated utilizing the tree bisection-reconnection branch-swapping method under the heuristic search option of the parsimony program of PAUP 4.0b10 [40]. Since bootstrapping under the heuristic search option of the parsimony criterion of PAUP 4.0b10 for MP trees composed of more than 150 taxa is computationally intensive, the consensus trees from the PAUP 4.0b10 analyses were compared to the less-robust bootstrapped trees generated by MEGA 4.0. Only the most relevant P2 alignment was bootstrapped under PAUP 4.0b10.

In the first strategy, all 176 full-length capsid amino acid sequences were used to generate trees by each method. In the second strategy, all full-length amino acid sequences were divided into groups based upon geographic isolation, and trees were generated by both methods. In the third strategy, the capsid amino acid sequence of all 176 sequences was divided into the three structurally defined domains and subdomains of S, P1, and P2, and trees were generated for each of these alignments by both methods. All trees generated were viewed using TreeViewPPC version 1.6.6 [48] or with tools from the MEGA 4.0 package.

Further, the bootstrapped MP trees generated by MEGA 4.0 for the entire capsid, S, and P1 were compared to trees generated utilizing the tree bisection-reconnection branch-swapping method under the heuristic search option of the parsimony program of PAUP 4.0b10 [40]. For the P2 subdomain, bootstrapped trees were generated and compared using both methods.

In addition, ancestral sequences were generated for selected nodes using the ANCECON program at the Max Planck Institute (http://toolkit.tuebingen.mpg.de/ancescon).

### Mapping Informative Sites onto the Predicted Structure

The x-ray crystal structure of the P domain of GII.4 virus VA387 in complex with the B-trisaccharide [32] (Protein Data Bank [PDB; http://www.rcsb.org/pdb/home/home.do] accession number 2OBT) was used to generate comparative models of each representative sequence using the program 3D-Jigsaw [49–51] with default parameters, and Modeller version 8.2 using the automodel class [52,53]. Five models of each were generated, and the model with the lowest objective function score was selected for analyses. The PDB files generated by these programs were visualized on molecular modeling tools MacPyMol (DeLano Scientific) and Chimera [54]. The evolving sites were mapped onto each model and onto the tertiary and quaternary structures of the P domain of VA387 in complex with A and B trisaccharides [32]. Rosetta Design [55] was used to generate relevant biological units for GII.4–1987 based upon the VA387 biological unit PDB file (accession no. 2OBT). Briefly, the changes that define GII.4–1987 were made on both chains of the VA387 template using the Rosetta Design Web server (http://RosettaDesign.med.unc.edu/index.html) with default parameters. In addition, residues within 5 Å of the variable sites were relaxed to allow repacking of side chains in the presence of the engineered mutations. Ten models were generated, with all ten predicting nearly identical structures.

### VEE Replicon Particles and Norovirus Virus-Like Particles

Capsid gene constructs for each of the representative strains were designed and synthesized as reported previously [56]. Briefly, the ORF2 genes of GII.4–1997 (LV-NC1) [57] and GII.4–2002 and 2002a were derived from RT-PCR products from outbreak stool samples collected in 1997, 2002, and 2004 [58], while the ORF2 genes of GII.4–1987, GII.4–2004 and GII.4–2005 were synthesized commercially by BioBasic (https://www.biobasic.com/index.php). To create GII.4–1987 D393G, a primer was designed to replace the Asp395 of GII.4–1987 with Gly395 of GII.4–1997 and the amino acid change introduced by PCR. All ORF2 constructs were then inserted directly into the VEE replicon vector for the production of VEE replicon particles (VRPs) (VRP-GII.4–1987, VRP-GII.4–1997, VRP-GII.4–2002a, VRP-GII.4–2002, VRP-GII.4–2004, and VRP-GII.4–2005) [57,59]. The virus-like particles (VLPs) were expressed in VRP-infected BHK cells, purified, and visualized by negative staining EM [57,59].

### Carbohydrate Binding Assays

VLP binding to HBGA-phenotyped salivary samples was determined as reported [57,60], with the exception of the use of mouse anti-VLP antisera (either strain-specific or cocktails) followed by anti-mouse-alkaline phosphatase (Sigma Aldrich) and pNPP (Sigma Aldrich) for VLP binding detection. VLP binding to synthetic HBGAs was determined using Neutri-avidin coated plates (Pierce) treated with 10 μg/ml biotinylated carbohydrate (Glycotech and the Consortium for Functional Glycomics Grant number GM62116) for 1 h and washed with PBS-0.05% Tween-20 before the addition of 1–2 μg/ml VLP for 1.5 h at 37 °C or room temperature. VLP binding was detected as described above. Blockade assays included serum pretreatment of the VLP for 1 h at 37 °C or room temperature before addition to the carbohydrate-bound plate. Assays using mouse antisera for blockade used rabbit polyclonal anti-GII VLP antisera followed by anti-rabbit IgG-AP (Sigma) for VLP binding detection. Blockade titer 50% (BT50) titers were defined as the lowest percentage of sera tested that blocked 50% of binding (BT50, blockade titer 50%) compared to levels determined in the absence of antibody pretreatment. Serum samples that did not reach a BT50 by the maximum percentage of sera tested were assigned a BT50 value equal to 2× the maximum percentage of sera tested for statistical analysis.

### Serology

Samples from an archived GII.4 outbreak occurring in 1988 were obtained from the National Calicivirus Laboratory of the Centers for Diseases Control and Prevention (Atlanta, Georgia, United States) and are summarized in Table 1. Any serum pair with a norovirus-positive stool sample or a ≥ 4-fold increase in anti-LV87 or LV97 IgG response between acute and convalescent samples (seroconversion) was studied for IgG reactivity and HBGA-VLP blockade across the panel of VLPs. Mice were immunized with VRP constructs as described [57]. Geometric mean titers of both human and murine VLP-reactive serum IgG were measured by ELISA [16,22,57] using VLP-binding detection methods as described above. Human and mouse anti-VLP serum IgG was compared to a purified IgG of known concentration for quantitation.

**Table 1:**
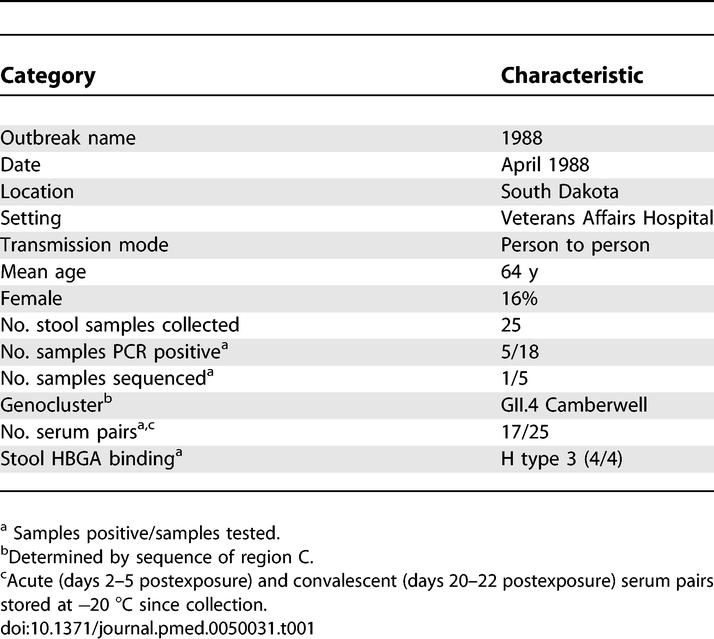
Characteristics of the GII.4 Outbreak Represented by the Samples Used in This Study

### Statistical Analysis

The Mann-Whitney two-tailed test (M-W test) was used to compare the median IgG responses between groups, and linear regression analysis was used to compare trends of IgG reactivity across the VLP panel for human serum samples. The one-way ANOVA was used to compare responses between murine immunization groups.

## Results

### Sequence Analysis and Molecular Phylogeny of the GII.4 Capsid

**Figure 1:**
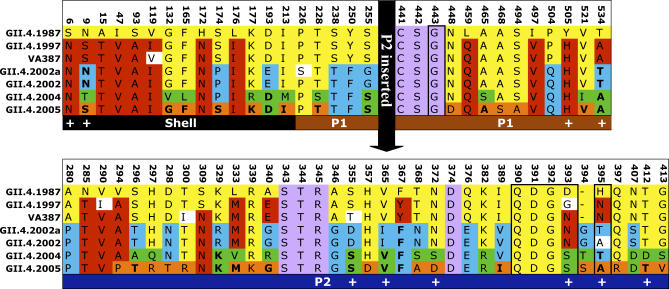
Evolutionary Analysis of Representative GII.4 Strains from 1987 to Present Five major evolutionary patterns were observed in the mutational profiles of the GII.4 sequences, and these are represented by GII.4–1987, GII.4–1997, GII.4–2002, GII.4–2004, and GII.4–2005. Yellow, amino acids present in the late 1980s Camberwell cluster (GII.4–1987); red, changes that occurred to form the Grimsby (GII.4–1997) cluster; blue, changes associated with the Farmington Hills (GII.4–2002) cluster; green, changes specific to the Hunter cluster (GII.4–2004); and orange, substitutions important for the Sakai cluster (GII.4–2005). A second GII.4–2002a sequence is included, as it encodes a single amino acid replacement at positively selected position 395 in the P2 subdomain as compared to GII.4–2002. The P2 region is highlighted in dark blue beneath the amino acids, with the N-terminal and C-terminal flanking regions of heterogeneity noted in black for the S domain and brown for P1. Lavender sites represent residues that hydrogen bond to the ligand at site 1; framed residues have been predicted to interact in a second stabilization domain. Amino acids operating under positive selection are marked below the column with a plus sign. Residues that are not colored represent single amino acid changes that were not seen in other strains in the cluster. Strain VA387 is included for comparison, as it is a Grimsby-like virus with a solved crystal structure of the P domain. Bold residues represent amino acids that reverted to a residue from a previous cluster.

An amino acid multiple alignment of 176 full-length GII.4 capsid sequences was generated (Figure S1) and columns of heterogeneity were exported as a table, and ordered by time. The 211 variable sites were refined to 59 informative sites (Table S2), and six distinct clusters were identified based upon sequence identity (Table S3). The five major evolutionary clusters were associated with and named according to outbreak strains (Table S3). These five clusters include: (1) the Camberwell cluster, which ranges from 1987–1995; (2) the Grimsby cluster from 1995 to 2002; (3) the Farmington Hills cluster from 2002–2004, (4) the Hunter cluster from 2004–2006, and (5) the Sakai cluster which includes viruses isolated 2004–2006 (Table S3). One representative sequence was selected from each major cluster, and these were named according to the date of isolation. For the Camberwell cluster MD145_12.1987.US (gb|AY032605, GenBank: http://www.ncbi.nlm.nih.gov/) was selected and named GII.4–1987. For the Grimsby cluster, an in-house Lordsdale 1997 (LV-NC1) [57] isolate was used as the representative sequence and was named GII.4–1997. Two sequences were used, based upon two in-house isolates, to represent the Farmington Hills clusters, and these were named GII.4–2002 for the cluster representative, and GII.4–2002a for a variant that differs by two replacements, one at position 226 (Pro in GII.4–2002 and Ser in GII.4–2002a) and one at site 395 (Ala395 in GII.4–2002 and Thr395 in GII.4–2002a). The Hunter cluster representative is Hunter284E.04O.AU (gb|DQ078794.2, GenBank: http://www.ncbi.nlm.nih.gov/), and it was named GII.4–2004. The Sakai.04_179.2005.JP (dbj|AB220922.1, GenBank: http://www.ncbi.nlm.nih.gov/) sequence was selected as the representative sequence for the Sakai cluster and was referred to as GII.4–2005. These sequences were aligned with VA387 as a reference sequence and informative sites were exported to a table (Figure 1). In addition, interaction sites identified in a recent structural study of the VA387 P domain were included (Figure 1).

The sixth cluster, named Den Haag, is composed of three viruses isolated in 2006, and also contains Minerva, one of the cocirculating GII.4 strains that caused the GII.4 pandemic in Europe and the United States during the winter of 2006 [4]. The extent of diversity among the clusters of GII.4 viruses is approximately 2%, with a total sequence identity of 90% between the earliest cluster, Camberwell, and the extant clusters Hunter, Sakai, and Den Haag. While variation was noted in the S, P1, and P2 regions of the capsid; the majority of heterogeneity occurred within the P2 subdomain (Figure 1; Table S3). Of the two receptor interaction sites recently reported, site 1 was strictly conserved in all clusters, while site 2 was highly variable at positions 393 through 395 (VA387 numbering) (Figure 1). Of note, strains occurring after the Grimsby cluster encoded an inserted amino acid between positions 393 and 394 of VA387 (Figure 1).

The fact that most amino acid replacements occurred in the surface-exposed P2 subdomain indicated that the different regions of the capsid protein were evolving under different evolutionary constraints, suggesting that the P2 region may be evolving by positive selection in response to herd immunity [61,62]. To determine if positive selection occurred during the evolution between the earliest cluster, Camberwell, and the extant clusters, five sequences were selected from each major cluster (Table S3), the 25 codon sequences were aligned, and the alignment was analyzed using HyPhy to detect positive selection by three different methods. Only five unique sequences were selected per cluster, as this was the maximum number of sequences available for the smallest major cluster, Camberwell (Table S3). This approach indicated that ten sites evolved by positive selection; these included residues at positions 6, 9, 355, 372, 393, 394/395 (site 394 in viruses of the Camberwell and Grimsby clusters and position 395 of all later clusters), 412, 505, and 534 (Table 2). Of note, this analysis was repeated three times using different random selections of five sequences from each of the five major clusters, and in all cases residues 9, 355, 372, 394/395, 505, and 534 were indicated as operating under positive selection (Table 2).

**Table 2:**
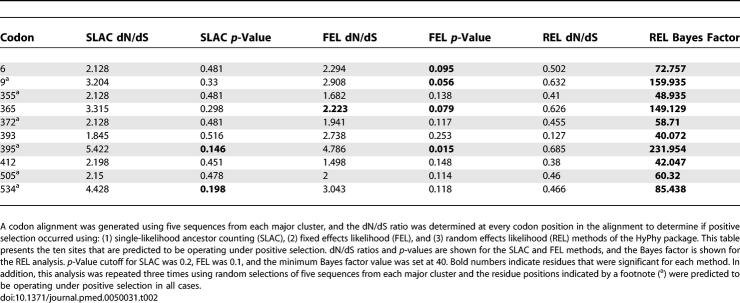
Detection of Positive Selection

### Origin of Outliers

Five outlier sequences were identified that did not appear to be associated with any cluster, and all of these had amino acid replacement patterns similar to two different clusters in different regions of the protein, as would be expected in the event of recombination, which has been reported for several genogroups of noroviruses [63]. Recombination analyses using the HyPhy package and RIP identified a putative break-point at position 794 of ORF2, and four of the five outlier sequences appear to be recombinants based on analyses performed by both methods (Table 3). These include Lanzhou/35666/2002/CHN, GA04/2004/US, Richmont/94/US, and Erfurt/007/00/DE. Further, two additional sequences were identified with substantial sequence anomalies, and these include EmmensE006/NL, which appears to be mosaic (Table 3), and Beijing/CR2905/CHN, which contains multiple unique replacements and a deletion that may be the result of sequencing error (Figure S1).

**Table 3:**
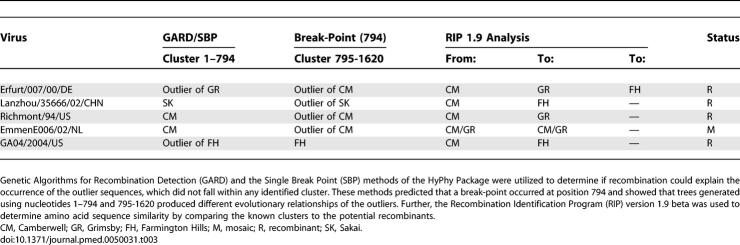
Recombination Detection Analysis

### Patterns of Molecular Evolution

**Figure 2:**
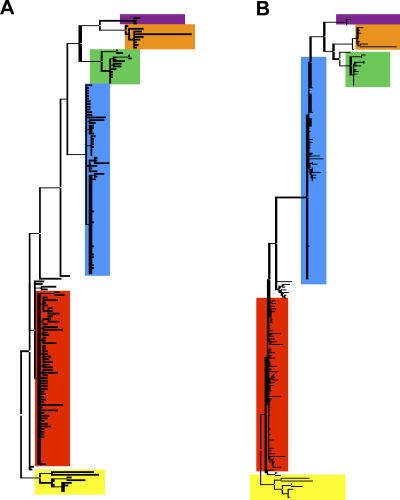
Phylogenic Reconstruction of the GII.4 Capsid Sequence A multiple alignment was generated using 176 full length capsid amino acid sequences and trees were generated using BI and MP analysis. The clusters are marked as follows: yellow, sequences from Camberwell cluster; red, sequences from Grimsby cluster; blue, sequences from the Farmington Hills cluster; green, sequences from the Hunter cluster; orange, sequences from the Sakai cluster; and purple, sequences from the Den Haag cluster. The trees are drawn to similar scales and are rooted with the earliest Camberwell cluster. (A) BI predicts that Camberwell gave rise to Grimsby, which gave rise to an LCA from which Farmington Hills and a LCA for the three extant clusters evolved independently (for full tree see Figure S2). (B) The MP bootstrapped tree generated by MEGA 4.0 was similar to the Bayesian tree with the exception that the Grimsby cluster gave rise to the Farmington Hills cluster, and a LCA of all extant clusters arose from Farmington Hills (for full tree see Figure S3). A MP tree generated by PAUP 4.0b10 predicted similar results (Figure S4).

Analyses of the BI and MP phylogenetic trees suggested that GII.4 capsid evolution was complex. Analysis of the full-length capsid amino acid sequences by these two approaches identified similar but not identical trees in which all six identified clusters were represented (Figures 2A, 2B, S2–S4). However, evolutionary patterns from the earliest to the latest cluster were confounded. While the Camberwell cluster was predicted to give rise to the Grimsby cluster, all extant clusters arose from a single last common ancestor (LCA) that likely evolved from the Grimsby cluster. However, no single evolutionary pattern was discernible, suggesting a complex evolutionary pattern in ORF2. In addition, there were several outliers that did not cluster with any specific group (Figures 2A, 2B, S2–S4).

To rule out the possibility that geographic variation confounded the overall phylogeny, we employed a second approach in which the sequences were separated by geographic region of isolation and analyzed by BI and MP. Four major groupings were analyzed with sequences derived specifically from The Netherlands, the UK, Germany, and the US. However, sampling biases and lack of sequences over the course of the 20-y time frame for each region resulted in trees that for the most part were incomplete, or that provided inconsistent evolutionary patterns (unpublished data). Only The Netherlands tree showed a linear-like progression from the earliest cluster to extant clusters, consistent with a recent report from that country [62].

**Figure 3:**
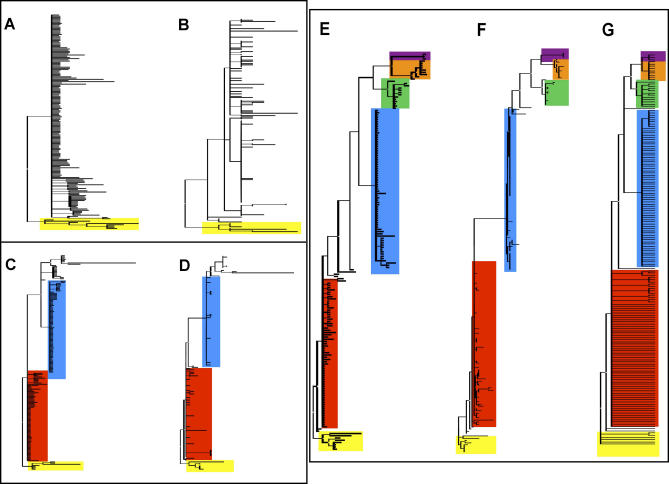
Phylogenic Reconstruction of the GII.4 Shell Domain and P1 and P2 Subdomains Independent multiple alignments were generated using 176 amino acid sequences divided into the S, P1, and P2 regions of the capsid, and trees were generated for each alignment using three different methods, with clusters marked as follows: yellow, sequences from Camberwell cluster; red, sequences from Grimsby cluster; blue, sequences from the Farmington Hills cluster; green, sequences from the Hunter cluster; orange, sequences from the Sakai cluster; and purple, sequences from the Den Haag cluster. Unmarked branches represent sequences that did not group with any specific cluster. The trees are drawn to similar scales and are rooted with the earliest Camberwell cluster. (A) BI of the S domain predicts only two distinct clusters with Camberwell as the first, while everything else groups into a single large cluster (for full tree see Figure S5). (B) The MEGA 4.0 MP tree of the S predicted similar results as the Bayesian tree, although there are two nondistinct clusters arising from the LCA derived from the Camberwell cluster (for full tree see Figure S6). A MP tree generated by PAUP 4.0b10 predicted similar results (Figure S7). (C) BI of the P1 domain predicted that the Camberwell cluster gave rise to the Grimsby cluster from which the Farmington Hills and all later clusters emerged, although the extant clusters were not fully resolved (for full tree see Figure S8). (D) The MEGA 4.0 MP tree of P1 predicted similar results, although it showed that Grimsby gave rise to the Farmington Hills cluster, from which the later clusters emerged. However, the Den Haag cluster falls within the Farmington Hills cluster (for full tree see Figure S9). An MP tree generated by PAUP 4.0b10 predicted similar results (Figure S10). (E) BI of the P2 subdomain predicts that Camberwell gave rise to Grimsby, which in turn gave rise to LCAs from which Farmington Hills and the three extant clusters evolved independently. All six clusters are distinct (for full tree see Figure S11). (F) The MEGA 4.0 MP bootstrapped tree agreed with the Bayesian tree (for full tree see Figure S12). (G) The MP bootstrapped tree generated using PAUP 4.0b10 predicted a nearly identical tree as the Bayesian tree (for full tree see Figure S13). The fact that all three methods generated similar trees with distinct clusters suggests that the P2 subdomain is the most appropriate region with which to determine phylogeny for the GII.4 noroviruses.

Virus proteins contain multiple functional domains, and protein domains are recognized as the units of molecular evolution [61]. Not surprisingly, different domains within a protein may evolve at different rates based on structural and functional constraints of the specific domain, potentially masking informative evolutionary patterns [61]. Analysis of the sequence variation of the three different domains of the GII.4 capsid showed that of the 59 informative sites noted (Table S3), 11 occurred in the S domain (5% of sites; 11/225 S alterations), 18 occurred in the P1 subdomain (11% of sites; 18/166 P1 alterations), and 30 occurred in the P2 subdomain (24% of sites; 30/126 P2 alterations), the surface exposed region of the capsid protein. This observation suggested that the different domains were evolving under different evolutionary constraints. Therefore, we employed a more sophisticated approach whereby the capsid amino acid sequences were divided into the three structurally defined domains and subdomains of S, P1, and P2, and each region was analyzed separately. Analysis of the phylogeny of S, which is the most conserved domain, showed only two predominant clusters represented by the Camberwell-like sequences as one cluster, while the second cluster was composed of all other sequences (Figures 3A, 3B, S5–S7). The P1 domain phylogeny indicated a linear progression from Camberwell to Grimsby to later strains, but the tree did not resolve the evolution of the later clusters of Farmington Hills, Hunter, Sakai, and Den Haag, and more information is necessary to resolve contemporary patterns (Figures 3C, 3D, S8–S10). Intriguingly, the surface-exposed P2 domain phylogeny suggests that the P2 region has evolved in a linear fashion, punctuated by periods of stasis, over the last 20 y in a pattern similar to influenza viruses [64]. Both BI and MP confirmed that the evolution of each cluster was correlated with time, with the Camberwell cluster being near the root of the tree, and the Grimsby cluster having origins in the Camberwell cluster (Figures 3E–3G, S11–S13). Further, the Farmington Hills and all later clusters appeared to have arisen from the Grimsby cluster. Evolution beyond the Farmington Hills cluster is less clear, as it appears that a LCA, which arose from the Grimsby cluster, gave rise to all four later clusters. However, the Bayesian posterior probability at this node was only 57/100 (Figure S11), and an MP (PAUP) bootstrap value was 65/100 (Figure S13), suggesting that there was not enough information to fully define this branching order. Therefore, we computed the predicted ancestral sequence for this node and compared this sequence to the Farmington Hills, Hunter, and Sakai cluster sequences. The LCA sequence was definitively more Farmington Hills-like, which implies that the Farmington Hills cluster is ancestral to the extant clusters (Table S3). This implication further suggests that the GII.4 viruses evolved in a linear manner, with each subsequent cluster giving rise to the next (Figure 1; Table S3) from Camberwell to Farmington Hills.

Although some clusters persisted for 8 y, contemporary clusters appear to have evolved from subsequent populations in much shorter intervals (Table S3), characteristic of epochal evolution. Taken together, these analyses suggest that the P2 region of the GII.4 viruses evolved in a linear direction over the last 20 y, with intense heterogeneity within the P2 region of the capsid sequence facilitating the emergence of new predominant strains.

To further evaluate this hypothesis, homology models of each of the five representative sequences were generated using the VA387 P domain as a template, and these models were analyzed to determine if microevolution in the P2 subdomain altered the capsid structure.

### Structural Modeling of GII.4 Evolution

**Figure 4:**
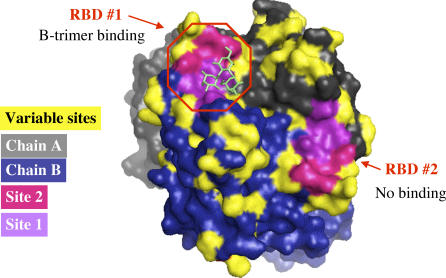
Variation Mapped to the VA387 Dimer Capsid dimerization creates two identical RBD that have been shown to bind to carbohydrate receptors. Each domain contains two sites that facilitate binding, with site 1 directly binding the α-fucose of B-trimer, shown in RBD#1 in this depiction. Interaction site 2 likely governs specificity, as it provides weak, long-distance interactions to the β-gal group of the B-antigen. Purple, site 1; pink, site 2; yellow, sites that have changed over 20 years of evolution; gray, chain A monomer; blue, chain B monomer; green, B-trimer carbohydrate.

Homology models of the P domain of the capsid monomer of each of the representative viruses were generated, and the changes that defined the emergence of each cluster were highlighted on each predicted structure, as well as the VA387 wild-type structure (Figure S14) [32]. The majority of heterogeneous sites mapped 180 degrees distal to a previously reported putative receptor-binding domain (RBD) (Figure S14) [30], with many of the mutations flanking the two receptor interaction sites identified recently (Figure S14) [32]. The biological unit for the GII.4 capsid is a dimer, and dimerization creates two identical RBDs, each of which contains the two sites shown to be important for receptor binding. Many of the sites that evolve differences between the GII.4 clusters map to regions surrounding both of these domains (Figure 4). In general, these residues map to the most distal edge of P2 where they protrude from the surface (Figure 4).

Evolutionary analyses showed that the fucose ligand binding residues reported for site 1 [32] were strictly conserved in the GII.4 viruses, while one amino acid position in interaction site 2 at position 393 was highly variable among the representative viruses. Further, many of the P2 sites operating under positive selection occur near the two interactions sites, with position 395 being an important residue adjacent to interaction site 2.

**Figure 5:**
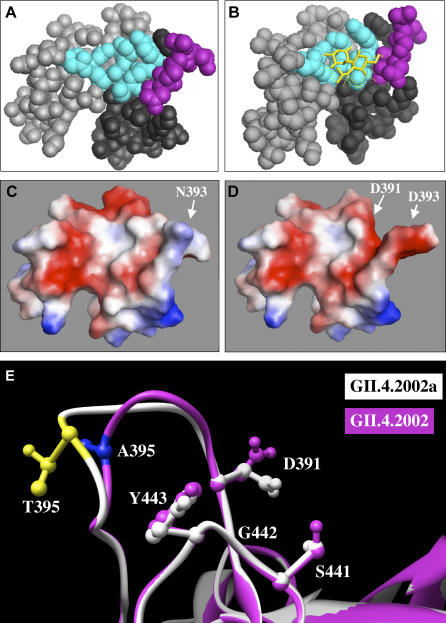
Subtle Changes in Interaction Site 2 May Govern HBGA Carbohydrate Binding (A) Two identical RBDs are formed upon dimerization of two capsid proteins (gray, chain A; black, chain B), with each RBD containing two sites important for HBGA carbohydrate binding. Site 1 (cyan) directly interacts with the ligand, while site 2 (purple) provides weak, long-range interactions that may regulate receptor specificity. (B) In VA387, B-trimer (yellow) binds to site 1 by a strong hydrogen bond network. (C and D) Electrostatic differences between RBDs of VA387 (C) and Rosetta-predicted RBD of GII.4–1987 (D) show that changing an Asp at 393 present in GII.4–1987 to Asn393 found in the GII.4–1997 cluster significantly alters interaction site 2. Repulsion between Asp393 and a conserved Asp391 forces the side chain of Asp393 away from the pocket. These alterations likely lead to differences in binding between GII.4–1987 and the GII.4–1997 cluster. (E) Molecular remodeling by mutation in GII.4–2002 cluster. Homology models for the P domain of GII.4–2002 and GII.4–2002a were generated with Modeller using the coordinates from PDB accession number 2OBT (in VA387 from cluster GII.4–1997), and the structures were superimposed upon one another and analyzed for differences. The predicted structures had an RMSD of 0.201 Å, with the only change in the structures occurring at position 395, where GII.4–2002 has an Ala residue and GII.4–2002a encodes a Thr. The addition of Thr at position 395 remodels interaction site 2, increasing the pocket by 1.111 Å. We predict that this subtle change in site 2 will be sufficient to alter HBGA binding.

These observations suggest that the heterogeneity most likely to interfere with receptor binding occurs in or near site 2. In particular, residues at positions 393–395, which change with each cluster, likely play a distinct role in carbohydrate binding affinity and/or avidity. Comparison of a structural model for the GII.4–1987 biological unit predicted by Rosetta Design, which contained an Asp at position 393, to the VA387 structure (Figure 5A and 5B), which contains Asn at position 393, predicts that this amino acid difference exerts two profound effects upon interaction site 2. First, an Asp at position 393 adds negative potential to site 2, which may inhibit some carbohydrate interactions (compare Figure 5C and 5D). Second, the side chain of Asp393 is likely repelled by a conserved Asp at position 391, resulting in subtle remodeling of interaction site 2 (Figure 5C and 5D), which potentially alters carbohydrate binding. Our model predicts that changes in interaction site 2 between GII.4–1987 and GII.4–1997 would alter carbohydrate binding and/or specificity. Thus, we introduced the Gly393 of GII.4–1997 into the backbone of GII.4–1987 by PCR mutagenesis, creating GII.4–1987 D393G, to study the effect of a single amino acid on HBGA interaction.

To further examine this region, homology models of GII.4–2002 (Ala395) and GII.4–2002a (Thr395), which differ by two amino acids at positions 226 (within the P1 domain) and 395 (within the P2), were compared and shown to have a root mean square distance of 0.201 Å. The two putative structures were superimposed and the region of the mutation was characterized (Figure 5E). Mutating Ala395 to Thr remodels interaction site 2 by increasing the size of the pocket by 1.1 Å (Figure 5E). In addition, this change is predicted to direct the negative side chain of Asp391 toward the pocket. The change at position 226 (Pro to Ser) occurs at the bottom of the P1 subdomain where the protruding domain and the shell domain are connected by the hinge region. This change occurs within five residues of one of the dimer interface sites. Although these changes are subtle, they may have profound influences on carbohydrate binding. From these two models, we hypothesize that microevolution in site 2 alters carbohydrate-binding specificity, and we predict that GII.4–2002 and GII.4–2002a will have different binding characteristics, facilitated by a single amino acid difference in the P2 subdomain.

To further characterize the binding characteristics of the GII.4–2002 viruses as well as the other representative viruses, the Camberwell GII.4–1987, GII.4–1987 D393G, Grimsby GII.4–1997, Farmington Hills GII.4 2002 and 2002a, Hunter GII.4–2004, and Sakai GII.4–2005 ORF 2 sequences were inserted directly into the VEE replicon vector and all seven replicons produced abundant 30–40 nm VLPs following visualization by negative strain EM techniques (Figure S15) [65–67].

### Carbohydrate Binding Profiles of VLPs

**Figure 6:**
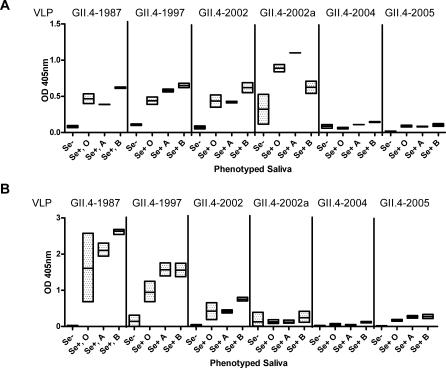
GII.4 VLP-Salivary Binding Patterns GII.4 VLPs were assayed for ability to bind to saliva samples phenotyped for secretor status (Se) and ABO blood type by ELISA. The mean optical density is indicated by the line in the box. The upper and lower boundaries of the box represent the maximum and minimum values. (A) VLP binding at 37 °C. (B) VLP binding at room temperature.

**Figure 7:**
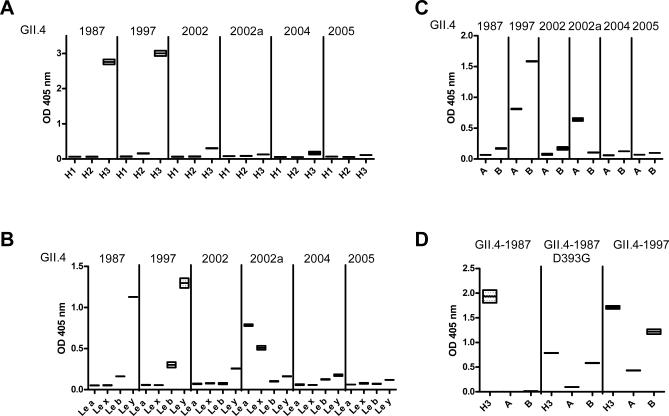
GII.4 VLP–Carbohydrate Binding Patterns at Room Temperature VLPs were assayed for ability to bind to synthetic biotinylated HBGA bound to avidin-coated plates. The mean optical density is indicated by the line in the box. The upper and lower boundaries of the box represent the maximum and minimum values. (A) VLP binding to core chains including an α-1,2-fucose. (B) VLP binding to either core chains or H antigens modified with the Lewis antigen. (C) VLP binding to A or B antigen trimer. (D) Comparison of binding of GII.4–1987, GII.4–1987 D393G, and GII.4–1997 VLPs to select HBGAs.

GII2.4–1987, GII.4–1997 and GII.4–2002/2002a demonstrated binding to secretor-positive saliva from individuals of blood types O, A, and B [60], although binding of GII.4–2002a was temperature dependent (Figure 6). These data are consistent with previously published salivary binding data for VLPs from Grimsby strains isolated in 1997 and 1998 [57,68]. GII.4–2002a also bound weakly to some secretor-negative saliva at 37 °C, suggesting that this strain may bind through the Lewis antigens. In contrast, GII.4–2004 and GII.4–2005 strains bound only weakly (2× background) to secretor-positive, blood type B saliva at room temperature. Since saliva is a complex biological fluid containing many carbohydrates in varying amounts depending on both the donor's genetics and sample integrity, this assay cannot positively identify specific carbohydrate binding partners or identify subtle differential binding patterns within the GII.4 VLP panel [69]. However, in agreement with salivary binding assays, synthetic HBGA (Figure S16) binding assays revealed three patterns of carbohydrate binding for the six VLPs (Figures 7 and S17). Although tested, none of the VLPs bound to any of the core chain precursor molecules (unpublished data). The first pattern exhibited by GII.4–1987, GII.4–1997, and GII.4–2002 utilized known FUT2-dependent HBGAs. GII.4–1987 VLPs bound strongly to H type 3 and less well to Le^y^; GII.4–1997 bound H type 3, but also bound efficiently to Le^y^; and A and B and GII.4–2002 bound to Le^y^ and less efficiently to H type 3 and B. The second pattern exhibited by GII.4–2002a utilized the Lewis enzyme products Le^a^ and Le^x^ as well as the FUT2-dependent A antigen. GII.4–2002a is the first GII.4 strain reported to bind FUT2-independent products, indicating a possible pathway for infection of secretor-negative individuals. GII.4–2004 and GII.4–2005 did not bind strongly to any of the carbohydrates tested (Figure 7), depicting the third binding pattern. It was particularly interesting that GII.4–1987 D393G had an intermediate binding phenotype between GII.4–1987 and GII.4–1997. Replacement of GII.4–1987 Asp393 with GII.4–1997 Gly393 resulted in the addition of binding to B antigen to GII.4–1987 but not the ability to bind A antigen, indicating that position 393 is important in determining HBGA binding, but that other surrounding residues must also play a role. Concordant with the predicted remodeling of the receptor binding pocket, these data support the hypothesis that sequence variation in and around the second carbohydrate-stabilizing domain of ORF2 alters VLP structure and modulates HBGA binding patterns within a genotype, resulting in changes in VLP–carbohydrate ligand binding over time (Figure 7).

### Serologic Relationships among the GII.4 VLPs

As the most prominent surface projection, the P2 subdomain may be a major antigenic determinant [23]. Thus, it is possible that the noted sequence variation across time in the GII.4 ORF2 protein could alter the affinity of antibodies to the individual outbreak strains and help explain the continued prevalence and emergence of new GII.4 strains, worldwide. Consonant with this idea, modeling of the noted GII.4 variation indicates substantial changes throughout the surface exposed P2 domain that would be predicted to alter the serologic relationships (Figure 4). To test this hypothesis, stools and sera collected from infected participants from a 1988 human GII.4 outbreak were studied for HBGA binding and VLP reactivity and blockade. Characteristics of the outbreak and samples collected from it are described in Table 1. Taken in 1988, these serum and stool samples were collected during the emergence phase of the GII.4 strains and potentially represent a baseline (preglobal spread) immune response to the future global epidemic strains.

**Figure 8:**
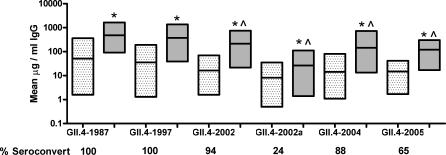
Anti-GII.4 VLP IgG Titers The geometric mean titer of anti-VLP IgG (μg/ml) for acute (dotted bars) and convalescent (shaded bars) serum samples collected from a 1988 GII.4 outbreak and the percentage of individuals who seroconverted to each VLP. The mean titer is indicated by the line in the box. The upper and lower boundaries of the box represent the maximum and minimum values. *Significant increase in titer between acute and convalescent samples (*p* < 0.05, M-W). ^Significant difference between convalescent titers compared to GII.4–1987 (*p* < 0.05, M-W).

Both acute and convalescent serum samples cross-reacted with each of the time-ordered VLPs. Figure 8 shows the percentage of individuals who seroconverted to each VLP and the geometric mean titer of acute and convalescent serum samples. While reactivity to GII.4–1987 and GII.4–1997 VLPs was similar (geometric mean fold increase 16.3 and 15.1, respectively), convalescent sera titers to GII.4–2002 (geometric mean fold increase 13.0, *p* = 0.02), GII.4–2002a (geometric mean fold increase 3.1, *p* < 0.001, M-W test), GII.4–2004 (geometric mean fold increase 10.1, *p* < 0.01, M-W test), and GII.4–2005 (geometric mean fold increase 7.8, *p* < 0.01, M-W test) were significantly and proportionately reduced as compared to GII.4–1987 (Figure 8). Comparison of the IgG titer across VLPs also demonstrated a significant negative trend in reactivity of 1988 outbreak convalescent sera and VLPs representing strains circulating at later times (*p* < 0.001, linear regression analysis). Of note, reactivity to GII.4–2002a was lower than reactivity to all of the other VLPs. Intriguingly, GII.4–2002 and 2002a differ at P226S and A395T only in the ORF2 capsid protein, and yet they vary in antibody reactivity, suggesting that one or both of these sites may encompass strong immunodominant epitopes.

To evaluate the extent of IgG cross-reactivity induced by the 1988 GII.4 infection, five randomly chosen serum pairs were assayed for reactivity to NV, a genogroup 1 strain [70]. The IgG titers were less reactive across genogroup [22] (unpublished data). Substantial changes between the reactivity of acute and convalescent serum pairs to NV VLP were not detected, none of the five tested pairs had a ≥ 4-fold increase in anti-NV titer (unpublished data), indicating that the increased response to all of the GII.4 strains is cluster- or genogroup-specific, not a broad-spectrum increase in total IgG in response to viral infection.

### Blockade Titer Varies over Time

**Figure 9:**
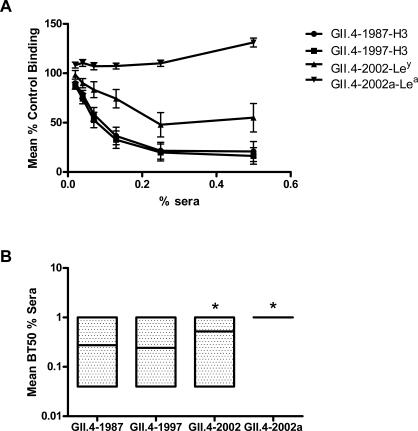
Blockade of GII.4 VLPs Binding to HBGA by Outbreak Sera Convalescent serum samples collected from a GII.4 outbreak in 1988 were assayed for blockade of GII.4–1987 and GII.4–1997-H type 3, GII.4–2002-Le^y^, and GII.4–2002a-Le^a^ interaction and the mean percentage of control binding calculated compared to the no-serum control binding (A). Error bars represent SEM. The floating bar plot (B) shows the mean percentage of sera needed for BT50 for each VLP. The mean titer is indicated by the line in the box. The upper and lower boundaries of the box represent the maximum and minimum values. *Outbreak sera BT50 responses significantly different from GII.4–1987 (*p* < 0.05, M-W)

Blockade experiments provide a biological measure of the ability of antisera to block the interaction of a specific VLP with a carbohydrate ligand partner, a surrogate assay for neutralization [57,60], in the absence of a cell culture system for noroviruses. Figure 9A shows the mean percentage of control binding of VLP in the presence of sera compared to the binding of VLP in the absence of antibody pretreatment. Although the acute serum samples reacted with GII.4–1987, GII.4–1997, GII.4–2002, and GII.4–2002a in the IgG enzyme immunoassay, none of the acute samples collected blocked the VLP–HBGA interactions, even at high serum concentrations (unpublished data). However, convalescent serum collected in 1988 blocked GII.4–1987 and GII.4–1997 interaction with H type 3 but was substantially less able to block GII.4–2002 VLP interaction with Le^y^ and was completely unable to block GII.4–2002a interaction with Le^a^ (Figure 9A). The mean concentration of sera needed to block VLP-HBGA binding by 50% (BT50) was 0.27% for GII.4–1987 and 0.24% for GII.4–1997 interaction with H type 3 (Figure 9B). These titers were significantly different from the sera titer (0.52%) needed to block GII.4–2002-Le^y^ interaction (*p* = 0.03, M-W test) and GII.4–2002a-Le^a^ interaction (>1%) (*p* < 0.001, M-W test), suggesting that the early Camberwell and Grimsby strains share common possible neutralization epitopes with each other that are not common to the later Farmington Hills GII.4 strains.

**Figure 10:**
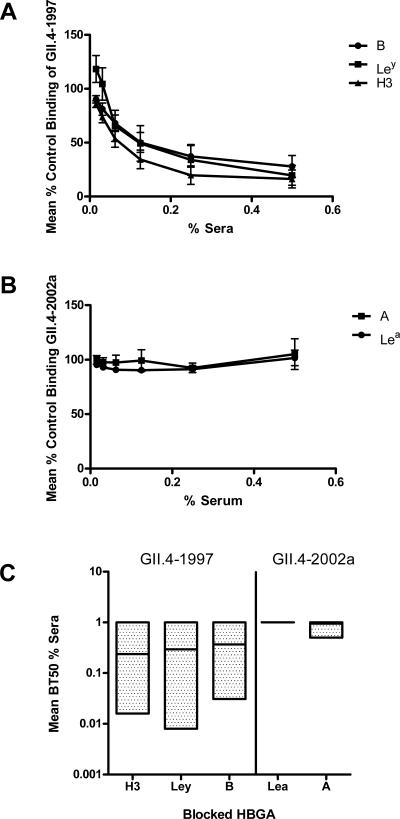
Blockade of Alternative HBGA Ligands by Outbreak Sera Convalescent serum samples collected from a GII.4 outbreak in 1988 were assayed for blockade of GII.4–1997-H type 3, Le^y^, and B-antigen trimer interaction (A) as well as GII.4–2002a-Le^a^ and A-antigen trimer interaction (B) and the mean percentage of control binding calculated compared to the no-serum control binding. Error bars represent standard error of the mean. The floating bar plot (C) shows the mean percentage of sera needed for BT50 for each VLP and alternative HBGA ligand. The mean titer is indicated by the line in the box. The upper and lower boundaries of the box represent the maximum and minimum values.

GII.4–1997 and 2002a VLPs both bind to multiple HBGAs in vitro. Thus, antibody blockade was compared for GII.4–1997 binding to B trimer, Le^y^, and H type 3, and GII.4–2002a binding to A trimer and Le^a^ to determine if the antibody blockade was effective against additional potential carbohydrate binding partners. Convalescent serum samples from the 1988 GII.4 outbreak similarly blocked GII.4–1997 with each of the potential binding partners (Figure 10), indicating that binding sites for additional HBGAs are physically close to each other on the GII.4–1997 VLP. In agreement with blockade of GII.4–2002a interaction with Le^a^, GII.4–2002a interaction with A trimer was unaffected by the 1988 sera (Figures 9 and 10).

To test if the blockade antibody generated after GII.4 infection could block a distant norovirus strain interaction with HBGA, five randomly selected serum pairs were tested for blockade of NV-H type 3 binding. There was no difference in the mean percentage of control binding between acute and convalescent serum samples (unpublished data).

### GII.4 Serologic Relationships Using Murine Sera

**Figure 11:**
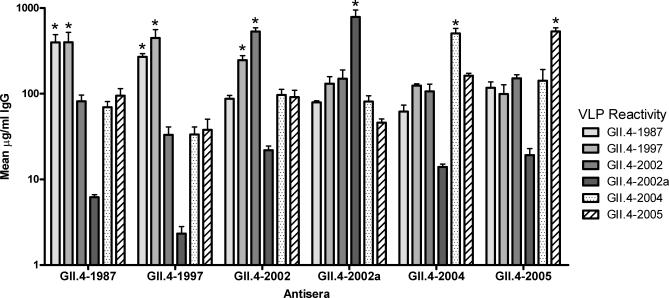
Murine Antisera Cross-Reactivity to GII.4 VLPs Mice were immunized by footpad inoculation on days 1 and 21 with 2.5 × 10^6^ IU VRPs expressing GII.4–1987, GII.4–1997, GII.4–2002, GII.4–2002a, GII.4–2004, and GII.4–2005 ORF2 (*n* = 4 per inoculation group). Antisera were collected on day 35 and analyzed for homotypic and heterotypic IgG responses to each VLP by ELISA. Antibody titers are represented as geometric mean μg/ml serum IgG. Error bars represent standard error of the mean. *VLPs with significantly different reactivity within an immunization group responses (*p* < 0.05, one-way ANOVA).

Analyzing human serum samples is complicated, as norovirus exposure histories are unknown and serologic relationships between strains are not well defined [57], making it challenging to decode the antigenic relationship between the time-ordered GII.4 strains in humans. As mice are not susceptible to human norovirus infection, they provide a clean background in which to study antigenic relatedness between unique time-ordered norovirus VLPs. Therefore, we immunized naïve mice with VRPs encoding the variant ORF2 of each of the GII.4 strains and collected sera for testing IgG cross-reactivity and blockade ability. As seen with human outbreak sera, antisera from mice immunized with VRP-GII.4–1987 or GII.4–1997 reacted similarly to GII.4–1987 and GII.4–1997 VLPs and to a lesser degree to GII.4–2002, GII.4–2002a, GII.4–2004, and GII.4–2005 VLPs (ranging from 1.6% to 24% of GII.4–1987 and 0.5% to 8% of GII.4–1997 homotypic responses for later GII.4 strains, *p* < 0.05, one-way ANOVA), indicating that antigenic sites are maintained more completely in early GII.4 strains while becoming variable in later emergent strains (Figure 11). Immunization with VRP-GII.4–2002 or 2002a, GII.4–2004, or GII.4–2005 elicited a strong homotypic response with weaker cross-reactivity (ranging from 0.03% to 16% of homotypic response) to all of the other strains tested (*p* < 0.01, one-way ANOVA), although GII.4–2002 had cross-reactivity to GII.4–1997 that was significantly higher than the other cross-reactive responses (*p* < 0.05, one-way ANOVA) (Figure 11). Immunization with any of the GII.4 VRPs did not result in a substantial antibody response cross-reactive to NV (unpublished data).

**Figure 12:**
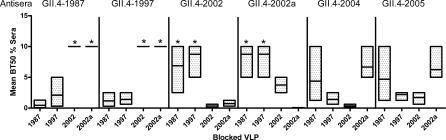
Murine Antisera Blockade of GII.4 VLP Binding to HBGAs Antisera collected from mice immunized against each GII.4 ORF2 were assayed for blockade of GII.4–1987- and GII.4–1997-H type 3, GII.4–2002a-Le^a^, and GII.4–2002-Le^y^ interaction and the mean percentage of control binding calculated compared to the no-serum control. The floating bar plot shows the mean percentage of sera needed for BT50 for each antisera and each VLP; the mean titer is indicated by the line in the box. The upper and lower boundaries of the box represent the maximum and minimum values. Antisera groups that did not block 50% VLP–HBGA binding at the highest serum concentration tested (5%) were assigned an arbitrary value of 10%. *VLPs with significantly different BT50 titer compared to the homotypic antisera-VLP BT50 titer (*p* < 0.05, one-way ANOVA).

Murine cross-reactive IgG data support the trend seen with human serum samples indicating clear serologic differences between the early and late GII.4 strains. To further test this hypothesis, blockade experiments were performed using mouse sera and BT50 values were compared. Antisera raised against GII.4–1987 and GII.4–1997 reacted similarly and effectively blocked both GII.4–1987 and −1997 interactions with H type 3, and both sera were unable to block GII.4–2002/2002a interaction with HBGA ligands (BT50 *p* < 0.01, one-way ANOVA; Figures 12 and S18). Conversely, antisera raised against GII.4–2002 or −2002a effectively blocked 2002/2002a interactions with HBGAs but were significantly less able to block GII.4–1987 (BT50 *p* < 0.05, one-way ANOVA) and GII.4–1997 (BT50 *p* < 0.01, one-way ANOVA) interactions with H type 3, again suggesting that the earlier strains share common blocking epitopes not found in the later Farmington Hills strains. GII.4–1987 and GII.4–1997 interaction with H type 3 was weakly blocked by sera against GII.4–2004 and GII.4–2005 (Figures 12 and S18). GII.4–2004 and GII.4–2005 sera efficiently blocked GII.4–2002-Le^y^ interaction but were significantly less able to block GII.4–2002a interaction with Le^a^ (BT50 *p* < 0.01 and *p* < 0.05, one-way ANOVA, respectively; Figure 12). In fact, GII.4–2002a-Le^a^ interaction was not efficiently blocked by any sera except the GII.4–2002/2002a samples, supporting observations with human sera (Figure 12). Our inability to identify carbohydrates that efficiently bound GII.4–2004 and GII.4–2005 precluded the testing of sera from historic strains to block the binding of contemporary strains to HBGAs. None of the antisera generated to the GII.4 panel blocked NV-H type 3 interactions at any of the serum concentrations tested (unpublished data). These data support the hypothesis that not only does antigenic drift occur in the capsid region of GII.4 norovirus strains over time, but that the variation greatly influences the ability of preexisting herd immunity to neutralize extant strains, based on carbohydrate blockade assays.

## Discussion

In this study we show that GII.4 norovirus evolution is epochal, with periods of stasis followed by the emergence of novel epidemic strains that evolve in a linear manner over time, and we map the antigenic variation onto the surface-exposed P2 capsid structure. Using a time-ordered panel of GII.4 VLPs from 1987–2005, we demonstrate that specific changes proximal to interaction site 2 regulate carbohydrate binding patterns, which have changed over the 20 y interval. Using sera from a human outbreak in 1988 and antisera from mice, we used ELISAs and an in vitro carbohydrate blockade as a surrogate neutralization assay to demonstrate that the noted variation alters the serologic and blockade responses consistent with a model of antigenic drift. Our data suggest a model of molecular evolution in which norovirus GII.4 strains persist by evolving novel carbohydrate-binding domains over time in response to immune-driven selection and by antigenic drift in the receptor-binding regions of the P2 subdomain.

Evolution in the norovirus capsid gene is complex, and our data are in agreement with other recent studies that underscore the critical importance of using protein structure to guide molecular phylogenetic analyses based on the hypothesis that protein domains evolve at different rates dependent on structural and functional constraints and environmental selective pressures [61]. In our analyses, the shell domain appears to be evolving by random drift, as only 5% of changes are informative (i.e., became fixed in the population). To a limited extent the P1 subdomain, and in particular the P2 subdomain, are evolving at higher evolutionary rates, consistent with our hypothesis that surface-exposed residues are evolving in the presence of immune selection. High rates of evolution in surface-exposed residues have also been reported in chronological sets of HIV samples within individual patients [71]. As the majority of the 176 ORF2 sequences included in our study belonged to the Grimsby and Farmington Hills clusters, the limited sequence information for contemporary clusters reaffirms the critical need for continued surveillance, the collection of full-length capsid sequence information, and detailed studies on the ORF2 evolutionary patterns of change noted in 2005 and beyond.

Our phylogenetic and evolutionary analyses of the P2 domain of ORF2 suggest that the GII.4 viruses have evolved linearly over the last 20 y in a fashion similar to influenza viruses, with serial replacements occurring sporadically, suggesting an epochal evolution in which periods of stasis are followed by sudden transitions [72,73]. The periods of stasis are likely the result of entropy barriers that generally occur in highly degenerate genotype-to-fitness populations in which many genotypes give rise to the same phenotype [72,73]. During the evolution of the GII.4 viruses, a long period of stasis of about 8 y or more has occurred within the ancestral Camberwell cluster, prior to the emergence of the epidemic Grimsby cluster. Of note, the majority of informative sites within the S domain occurred during the emergence of the Grimsby cluster, and these sites became fixed in the population. With our analyses, we cannot rule out the possibility that these changes to S were the key changes structurally necessary to facilitate the emergence of the GII.4 cluster as the predominant epidemic strain. The Grimsby cluster endured a shorter period of stasis, after which subsequence clusters appear to have evolved from the previous cluster in a linear manner. Later clusters appear to emerge every 1–2 y from 2002 to present. Although all six clusters are distinct, there is overlap between some clusters, and the dates of isolation of some strains that group with ancestral clusters clearly occur after the emergence of later clusters. This variation suggests that strains from earlier clusters may continue to circulate, but likely cause asymptomatic disease, or persist at low levels in the population prior to going extinct.

Analyses of the evolutionary profiles of the GII.4 viruses suggest that many of the outlier sequences are recombinant viruses, consistent with earlier reports by other groups [63]. The recombination break-point is predicted to occur near the first P1/P2 boundary (nucleotide position 794/amino acid 265), suggesting that viable recombination may be restricted to crossover sites that preserve essential protein domain function (Table 3). In addition, some sites in the P2 region appear to revolve between a select subset of amino acid replacements. These sites include 329, 333, 340, 355, and 365. We predict that these are important sites of antigenic variation, but are structurally limited in that they must maintain a specific physiochemical property important for the overall capsid structure or the interaction with carbohydrate, or are structurally constrained by entry mechanisms.

Sites of heterogeneity predominantly occurred in the exposed P2 subdomain in and around the two carbohydrate-interaction sites that form the receptor binding pocket [23,26,32]. Site 2 was the most variable region in our model and changes in this region affected carbohydrate binding profiles. Our empirical studies suggest that escape from herd immunity may represent the selective force that drives antigenic variation within and around the receptor binding pocket on the surface of the GII.4 P2 domain of ORF2. Variation within the RBD in ORF2 variants is likely under strong coselection to maintain recognition of one or more HBGA carbohydrate receptors for docking and perhaps entry, allowing the GII.4 noroviruses to persist and simultaneously circumvent highly penetrant susceptibility alleles that are common in human populations. Alternatively, as the current contemporary strains do not bind any carbohydrates tested, the receptor binding pocket may evolve to recognize other fucosylated carbohydrates or proteins for docking.

In influenza viruses, herd immunity—mediated primarily by neutralizing IgG antibodies [74]—positively selects for antigenic variation in hemagglutinin, although the exact effect of individual mutations on antigenicity is complex. Mutations may occur in one or more of five neutralizing epitopes or in the sialic acid-binding site in the hemagglutinin glycoprotein, thus selecting for replacement strains that circumvent antibody neutralization [64]. Among noroviruses, the concept of herd immunity is controversial; early human challenge studies suggested that strain-specific, long-term immunity can be elicited following challenge, as 50% of volunteers did not become infected after multiple challenges with NV. However, the same study demonstrated that in some volunteers only short-term immunity was evident [75,76]. In more recent studies, we and others have argued that long-term immunity is possible and that pre-exposure history may influence the duration of the protective immune response against individual strains [16,22,77]. Although early mucosal IgA [16] and T cell [22] responses may include components of a long-term protective immune response in uninfected, challenged volunteers, the role of serum IgG in protective immunity remains controversial. Norovirus-challenged volunteers or outbreak patients mount strong serum IgG antibody responses that block carbohydrate–VLP interactions in a genogroup-specific manner in a surrogate neutralizing assay potentially representing a component of a long-term protective immunity [57,60]. However, IgG antibody levels are usually too low in prechallenge sera, or in salivary or fecal samples, for assaying by these methods. Importantly, the years following the emergence of a new epidemic strain in Europe were characterized by decreased numbers of outbreaks, speculated to be associated with increased herd immunity [62,77]. If herd immunity drives GII.4 norovirus evolution, these data predict that serologic relationships among temporal GII.4 epidemic strains should change over time.

Although GII.4–1987 and GII.4–1997 VLPs differed by seven amino acids, no significant differences in antibody reactivity were noted with sera derived from humans and experimentally immunized mice, suggesting that the few amino acid changes did not significantly alter variation between the two strains during the long period of stasis. We speculate that pre-1995 Camberwell-like strains typically produced low-level endemic disease in human populations. By the mid 1990s, a series of mutations evolved that promoted epidemic spread of the post-1996 Lordsdale/Grimsby strains in human populations, perhaps by allowing for more efficient binding with additional HBGA ligands on mucosal surfaces, altering the stability of the capsid, or promoting transmissibility. The epidemic spread of the GII.4–1997-like strains in human populations may have subsequently allowed for higher levels of herd immunity and selected for faster antigenic changes in future strains. Influenza viruses show similar trends, in that genetic variation oftentimes, but not always, tracks with antigenic variation, because some mutations result in disproportionately large antigenic changes [78]. However, global serologic responses between GII.4–1987/1997 and GII.4–2002/2002a demonstrated significant antigenic differences, reflecting the increased number of variant residues. Concordant with these findings, GII.4–2004 and GII.4–2005 epidemic strains were also serologically quite distinct from GII.4–1987 and GII.4–1997, and to a lesser extent distinct from GII.4–2002, but not from 2002a. Thus, epidemic replacement strain ORF2 capsid sequences were antigenically related yet distinct due to antigenic drift.

Given the high amount of GII.4 cross-reactivity, it is clear that one or more highly conserved epitopes define the serology of this genocluster. Immunodominant neutralizing epitopes have been described for a number of viruses, including West Nile virus [79], HIV-1 [80], and foot-and-mouth disease virus [81]. Findings with the GII.4–2002 and −2002a ORF 2 capsid proteins support the possibility that GII.4 noroviruses may also encode a limited number of strong immunodominant epitopes. Compared to GII.4–2002, the GII.4–2002a norovirus ORF2 protein differs by two residues, defined by changes in P1 (P226S) and P2 (A395T), yet is antigenically quite distinct from all other strains tested (Figures 8, 9, 11, and 12). Previous work with foot-and-mouth disease virus, demonstrated that a single-amino acid change in an immunodominant epitope resulted in two antigenic specificities and a lack of virus cross-neutralization [82]. Although speculative, the noted P2 variation is unlikely to encode this strong serologic change in GII.4–2002a, as other time-ordered VLPs encode amino acid changes at this position as well. Rather, we predict that the alteration in P1 (P226S) might well define a major immunodominant epitope. Experiments are in process to test this interesting hypothesis. On the structural model, the side chain of Ser226 is much smaller than the Pro side chain and it extends away from the surface into an open cavity below the dimer interface region. This change may alter the final conformation of the viral capsid by relaxing the constraints on the hinge movement. Clearly, detailed structure analyses of the time-ordered GII.4 VLP set will likely prove informative.

All convalescent outbreak sera blocked carbohydrate binding of GII.4–1987 and GII.4–1997 VLPs but were less capable of blocking GII.4–2002/2002a binding. Interestingly, the mouse anti-GII.4–2004 and GII.4–2005 sera more efficiently blocked binding of GII.4–1987 and GII.4–1997 to H type 3 than GII.4–2002/2002a binding to Le^y^ and A. Of note, amino acids at positions 329, 355, and 365 in GII.4–2004 and GII.4–2005 are the same as GII.4–1987 and GII.4–1997, but not GII.4–2002/2002a, which implies that these sites may account for the cross blockade of anti-GII.4–2004 and anti-GII.4–2005 sera to GII.4–1987 and GII.4–1997 carbohydrate binding. These sites may also be important determinants of antigenic variation within the GII.4 genocluster.

The absence of a robust cell culture model for noroviruses prevents the development of classical neutralization assays. However, studies with numerous virus families have indicated that antibodies that block virus receptor–ligand interactions provide one mechanism to neutralize virus infectivity [83–85]. Previous studies by our group and others have demonstrated that noroviruses bind to HBGAs, and that HBGAs are necessary for infection, since the *FUT2* gene is a susceptibility allele for Norwalk virus infection in vivo [16,19]. Although a GII.4 human challenge model does not exist, some GII.4 noroviruses have been reported to bind specifically to H type 3 and to a lesser extent to the A and B carbohydrates, suggesting usage of HBGAs in infection [57,86]. Further, GII.4 outbreak investigations have established a strong correlation between a secretor-positive phenotype and symptomatic infection [21]. However, carbohydrate-binding patterns within a temporal panel of norovirus VLPs have not been reported until now. Consonant with clear variations in overall serologic identity among the GII.4 VLP panel, we have also demonstrated that the GII.4 VLPs display variant binding patterns to carbohydrates typically regulated by FUT1 (Le^x^, Le^y^), FUT2 (H Type 3), FUT3, the Lewis enzyme, (Le^a^, Le^y^), and the A and B enzymes. These findings suggest that some GII.4 noroviruses not only bind carbohydrates regulated by the *FUT2* susceptibility allele, but also can bind carbohydrates regulated by *FUT1* and *FUT3* and the *A* and *B* alleles as well. However, to date, FUT1 expression has not been demonstrated in the gut mucosa [87]. As fucosyltransferase enzymes lack tight core chain fidelity in vitro, it is possible that the FUT2 enzyme, or another fucosyltransferase, may express typically FUT1-regulated carbohydrates in the gut, as has been observed in saliva where FUT2 activity produces both Le^x^ and Le^y^ from type 2 core chain [87]. Further, we and others have not seen in vivo evidence of core chain usage by alternative fucosyltransferases, as FUT2-negative individuals were completely resistant to NV infection [16] and more likely to be asymptomatic after GII.4 exposure [21], regardless of the presence of other fucosyltransferases. Most surprisingly, the GII.4–2004 and GII.4–2005 strains did not bind any HBGA carbohydrates or saliva tested, suggesting that their carbohydrate ligands are either not represented within the panel of biotinylated HBGA carbohydrates available for testing, carbohydrate patterns differ in saliva and in intestinal mucosa, or they utilize non-HBGA-mediated pathways for entry. Thus, over time, it is reasonable to predict that noroviruses have the capacity to utilize the large number of related HBGAs as ligands. The potential plasticity in the carbohydrate-binding site would likely accommodate sufficient amounts of antigenic drift to escape herd immunity, while simultaneously preserving carbohydrate-binding potential and altering strain susceptibility to the many different human alleles that regulate HBGA expression.

Fucose ligand binding site 1 was strictly conserved in the GII.4 viruses, including, paradoxically, extant strains that only weakly bind saliva and do not bind any carbohydrate tested. In contrast, the secondary interaction site appears to facilitate carbohydrate specificity as binding characteristics of the time ordered VLP panel varied extensively. In interaction site 2, positions 390, 391, 392, and 443 were conserved throughout the GII.4 strains while sites 393, 394, and 395 were variable. In two instances, binding characteristics could be directly correlated to residue changes within this region. First, structural models predicted that carbohydrate binding would differ between the Camberwell cluster and the Grimsby cluster (including VA387), based primarily upon an Asp-to-Asn change at position 393 in site 2 (Figure 5). In agreement with our hypothesis, binding between GII.4–1987 and GII.4–1997 was different. The substitution of an Asp at position 393 was predicted and then empirically demonstrated to sterically hinder or otherwise alter binding of the larger trisaccharide moieties of A- and B- antigens, as the Camberwell representative VLP binds H type 3 and Le^y^ but not A or B (Figure 7). In contrast, both GII.4–1997 and VA387 bind H type 3, Le^y^, A, and B [57,88]; and they encode Gly and Asn at the 393 position, respectively. Interestingly, our data suggest that the primary impact of the mutations that occurred between the Camberwell and Grimsby clusters led to an expansion of HBGA usage, as representative strains GII.4–1987 and GII.4–1997 were indistinguishable antigenically. In the second case, a Thr at position 395, as exhibited by GII.4–2002a (Figure 5E) altered the receptor binding pattern as this mutant bound to Lewis enzyme products, Le^a^ and Le^x^, as well as the FUT2-dependent product, A antigen. GII.4–2002a is the first GII.4 strain reported to bind FUT2-independent products, indicating a possible pathway for infection of secretor-negative individuals. Alanine at this position facilitates binding of H type 3 in GII.4–2002. These results are also in agreement with our hypothesis that microevolution in site 2 alters carbohydrate-binding interactions; more detailed genetic studies should confirm this hypothesis. Of note, the synthetic HBGAs used in this study lack the complex structures often found in vivo. Larger polysaccharide moieties likely play a crucial role in carbohydrate affinity and avidity by interacting directly with interaction site 2.

Taken together, our structural models (Figure 5) suggest that heterogeneity in the receptor interaction site 2 likely determines HBGA affinity and avidity, and subtle changes in this region may govern HBGA specificity. Tan et al. [30] demonstrated that binding of VA387 to HBGAs could be ablated by mutating the Thr at position 338 to Ala. While Thr338 is not directly involved in ligand binding, it does form hydrogen bonds to Arg345, which directly hydrogen bonds to the ligand [23,26,32]. It seems likely that hydrogen bonding patterns also influence which ligands the virus can bind. Subtle changes to residues that form hydrogen bonds with the primary ligand interaction residues may drastically alter ligand affinity and avidity. In addition, the length and charge of the side chain of a given residue likely allosterically regulates the site by sterically hindering some interactions (Figure 5). Studies with foot-and-mouth disease virus have demonstrated that this virus contains a conserved shallow pocket on its surface that is predisposed to evolve a high affinity for its heparin sulfate receptor. Mutations remodel the surface by increasing the positive charge, which results in an increased affinity for its receptor [89]. Differences in electrostatic potential may regulate HBGA affinity in GII.4 viruses as well, as the addition of a charge at position 393 alters the surface charge and binding of the GII.4–1987 virus (Figure 5A–5D). Studies with influenza virus have shown broad serologic differences between temporally distinct strains consistent with a phenotype of antigenic drift and variation, especially in antigenic sites, receptor-binding sites, and codons previously identified as being under positive selection [64].

Virus recognition of variant carbohydrate receptor moieties is not unprecedented; influenza viruses recognize variant sialic acid moieties for infection of aquatic birds (α2–3 sialic acid) and humans (α2–6 sialic acid), and other viruses utilize similar mechanisms [90–92]. However, the recognition specificities are much more subtle and complex than originally appreciated. Recently developed glycan microarray tools have demonstrated that different human and avian influenza virus strains bind to different glycan ligands depending upon downstream fucosylation, sulfation, and additional sialylation processing patterns, although the biological significance of these interactions are not fully known [93]. Our data and that of others [62,77] suggest that antigenic drift in norovirus ORF2s (HBGA antigens) and perhaps influenza virus (sialic acid-containing antigens) hemagglutinin may evolve by similar mechanisms. The combined flexibility of the ligand-binding pocket and the wide range of variant, yet related carbohydrate ligands, may provide the plasticity in both the receptor targets and viral attachment proteins necessary to allow for extensive antigenic drift in the face of herd immunity.

The data presented in this manuscript provide support for the hypothesis that antigenic drift and receptor switching may function synergistically to maintain the GII.4 noroviruses in the presence of human herd immunity. Our data suggest that strain-specific protective immunity is possible and that vaccines and immune prophylaxis must be formulated to protect against contemporary strains. As shown with influenza viruses, new therapeutic formulations will be necessary. Moreover, continued norovirus surveillance will be essential for maintaining vaccine and drug effectiveness.

At this time, it is unclear whether GII.4 noroviruses will continue to predominate as the major cause of epidemic gastroenteritis worldwide, or (like influenza virus) undergo an antigenic shift to a variant GI or GII genocluster that is currently circulating at low levels in human populations, or whether a new strain will be introduced from zoonotic pools. However, important caveats must be considered when evaluating this work. While it is clear that the mucosal compartment has high concentrations of IgG, carbohydrate–VLP blockade assays use serum IgG, whereas mucosal IgA and IgG responses may be more important in protective immunity [16,57]. Unfortunately, mucosal antibody concentrations are usually not only insufficient for blockade studies, but were not obtained during norovirus outbreaks, preventing the testing of this possibility. In the absence of a robust cell culture model, blockade studies themselves represent a surrogate assay for neutralization, and it is possible that antibodies might neutralize virus infectivity by binding to regions distinct from the carbohydrate-binding pocket or even outside of P2 and inhibit other steps in entry, as shown with West Nile virus, among others [94]. Research is clearly needed to define the number and location of the neutralizing sites in the norovirus particle and the impact of positively selected mutations on the neutralization phenotype. Structural studies solving variant carbohydrate-binding characteristics in the time-ordered VLP set will be imperative for understanding the role of the secondary sites in receptor specificity and binding affinity. Further, the VLPs used in this study are composed of ORF2 major capsid protein, whereas native virions would also include one or two copies of the ORF3 protein [95]. The function of the ORF3 protein is still unclear, and its effect on virus structure and interaction with ligands is unknown. Although no examples of norovirus VLP post-translational modifications have been reported, any such modifications may impact ligand interaction. In this study VLPs were produced in a mammalian expression system, thus post-translational modifications should reflect natural processing. Finally, although HBGAs clearly function as ligands for Norwalk virus entry, clear evidence that HBGAs function for GII.4 docking and entry is less robust, but suggestive, and it is not clear whether differential binding patterns noted in vitro reflect in vivo binding and susceptibility phenotypes [18,21]. In the absence of time-order GII.4 human challenge inocula, it will be difficult to definitively prove that contemporary strains circumvent immune responses to preexisting strains. Additional studies will be needed to determine whether the evolutionary patterns are unique to the GII.4 noroviruses or represent a general evolutionary pattern of the norovirus family. Our study, however, presents a predictive model for future empirical studies investigating the relationships among antigenic change, norovirus pathogenesis, vaccine design, and human disease.

## Supporting Information

Figure S1 The Multiple Alignment Generated Using All 176 Sequences with ClustalX with the PAM Matrix (38 KB PDF)

Figure S2 Bayesian Tree of the Full-Length Capsid Node confidences are marked as posterior probabilities.
(62 KB PDF)

Figure S3 MEGA4.0 MP Tree of the Full-Length Capsid Bootstrap analysis was conducted with 100 replicates. Nodes with less than 50/100 bootstrap support were collapsed.
(21 KB PDF)

Figure S4 PAUP4.0b10 MP Tree of the Full-Length Capsid Node confidences are shown as percent present in equally parsimonious trees.
(24 KB PDF)

Figure S5 Bayesian Tree of the Shell Domain Node confidences are marked as posterior probabilities.
(62 KB PDF)

Figure S6 MEGA4.0 MP Tree of the Shell Domain Bootstrap analysis was conducted with 100 replicates. Nodes with less than 50/100 bootstrap support were collapsed.
(20 KB PDF)

Figure S7 PAUP4.0b10 MP Tree of the Shell Domain Node confidences are shown as percent present in equally parsimonious trees.
(23 KB PDF)

Figure S8 Bayesian Tree of the P1 SubdomainNode confidences are marked as posterior probabilities. (61 KB PDF)

Figure S9 MEGA4.0 MP Tree of the P1 Subdomain Bootstrap analysis was conducted with 100 replicates.
(19 KB PDF)

Figure S10 PAUP4.0b10 MP Tree of the P1 SubdomainNode confidences are shown as percent present in equally parsimonious trees. (23 KB PDF)

Figure S11 Bayesian Tree of the P2 Subdomain Node confidences are marked as posterior probabilities.
(63 KB PDF)

Figure S12 MEGA4.0 MP Tree of the P2 Subdomain Bootstrap analysis was conducted with 100 replicates. Nodes with less than 50/100 bootstrap support were collapsed.
(21 KB PDF)

Figure S13 PAUP4.0b10 MP Tree of the P2 Subdomain Bootstrap analysis was conducted with 100 replicates. Node confidence is reported using bootstrap values.
(21 KB PDF)

Figure S14 GII.4 Evolution Modeled on the VA387 Capsid Monomer The majority of heterogeneity surrounds the receptor interaction sites 1 and 2. Yellow, sites changing over the past 20 y of evolution; purple, ligand-binding site 1; pink, interaction site 2. Each panel represents counterclockwise revolutions on the y-axis.
(1.7 MB PDF)

Figure S15 Electron Micrograph of GII.4 VLPs Purified GII.4 ORF2 VLPs were visualized by negative stain EM.
(1.8 MB PDF)

Figure S16 Biosynthetic Pathway of Histo-Blood Group Antigens HBGA of the intestinal tract are produced by the successive addition of carbohydrate moieties onto core precursor chains of type 1 and type 3 (A), and type 2 core chain (B).
(25 KB PDF)

Figure S17 GII.4 VLP-Carbohydrate Binding Patterns at 37 °C VLPs were assayed for ability to bind to synthetic biotinylated HBGAs bound to avidin-coated plates. The mean optical density is indicated by the line in the box. The upper and lower boundaries of the box represent the maximum and minimum values.
(A) VLP binding to core chains including an α-1,2-fucose.
(B) VLP binding to either core chains or H antigens modified with the Lewis antigen.
(C) VLP binding to A- or B-antigen trimer.
(24 KB PDF)

Figure S18 Murine Antisera Blockade of GII.4 VLP Binding to HBGAs Antisera collected from mice immunized against each GII.4 ORF2 were assayed for blockade of GII.4–1987-H type 3 (A), GII.4–1997-H type 3 (B), GII.4–2002-Le^y^ (C), and GII.4–2002a-Le^a^ (D) interaction and the mean percentage of control binding calculated compared to the no-serum control binding.
(33 KB PDF)

Table S1 176 Norovirus ORF2 Sequences Used in This Study Accession numbers beginning with EU are newly sequenced strains received from CDC.
(36 KB XLS)

Table S2 Identification of Informative Sites A total of 211 variable sites were reduced to 59 informative sites based upon four criteria: (1) columns with single amino acid replacements; (2) columns containing multiple single incongruous amino acid replacements; (3) columns containing random amino acid replacements not associated with a geographic lineage or specific cluster; and (4) lineage-specific replacements that were noninformative.
(17 KB XLS)

Table S3 GII.4 Sequences Used in This Study The 176 sequences used in this study were aligned with ClustalX, refined as described, and 59 informative sites were exported to an Excel table, shown here. The sequences were arranged into clusters based upon at least 98% sequence identity. Clusters were named according to outbreak strains associated with the cluster, and these are marked by color and by name in the “Cluster” column. The Camberwell and Hunter clusters were further divided into subclusters by sequence similarity (at least five residues in common among all subcluster, but not cluster, sequences), and all clusters and subclusters were arranged by date of isolation. Amino acid replacements associated with each cluster are highlighted as follows: yellow, amino acids derived from the Camberwell cluster (1987–1995); red, amino acids from the Grimsby cluster (1995–2002); blue, residues that evolved in the Farmington Hills cluster (2002–2004); green, amino acids that occurred in the Hunter cluster (2004–2006); and orange, residues present in the Sakai cluster (2004–2006). Residues that evolved within a sixth cluster, named Den Haag, composed of three viruses isolated in 2006, are marked in purple. Amino acids that occur primarily in one cluster with secondary representation in another cluster are highlighted in the primary color. Sequences selected to represent each cluster for the tests for positive selection are shown in bold. Sequences defined as outliers are marked by black boxes. Clusters and subclusters are marked with roman numerals to the right. The ancestor for the node that gave rise to all clusters from Farmington Hills to Hunter, Sakai, and Den Haag was generated and is shown in comparison (gray-filled boxes). It most closely resembles Farmington Hills.
CM, Camberwell, DH, Den Haag.
(356 KB PDF)

## Abbreviations

BI: Bayesian inference
BT50: blockade titer 50%
FEL: fixed effects likelihood
GARD: genetic algorithms for recombination detection
HBGA: histo-blood group antigen
LCA: last common ancestor
MP: maximum parsimony
NV: Norwalk virus
ORF: open reading frame
P: protruding [domain]
RBD: carbohydrate ligand receptor-binding domain
REL: random effects likelihood
S: shell [domain]
SBP: single break-point
SLAC: single-likelihood ancestor counting
VLP: virus-like particle
VRP: VEE replicon particle
