# Supplementary material for: Mechanisms of GII.4 Norovirus Persistence in Human Populations
Source: PLoS Med. 2008 Feb 12;5(2):e31. doi: 10.1371/journal.pmed.0050031 (PMC2235898; doi:10.1371/journal.pmed.0050031)
Supplement: Supplementary file 1 [file pmed.0050031.sg001.pdf]

Camberwell.94.AU	MKMASSDANP	SDGSAANLVP	EVNNEVMALE	PVVGAAIAAP	VAGQQNIIDP	WIRNNFVQAP	GGEFTVSPRN	APGEILWSAP	LGPDLNPYLS	HLSRMYNGYA
MD145_12.87.US	----------	----------	----------	----------	----------	----------	----------	----------	----------	----------
GII.4.87	----------	----------	----------	----------	----------	----------	----------	----------	----------	----------
MD134_7.87.US	----------	----T-----	----------	----------	----------	----------	----------	----------	----------	----------
Lord.93.UK	-----N----	----------	----------	----------	------V---	----------	----------	----------	----------	----------
UK3_17.12700.92.GB	-----N----	----------	----------	---D------	------V---	----------	----------	----------	----P-----	----------
EmmenE006.02.NL	-----N----	----------	----------	----------	----------	----------	----------	----------	----------	----------
Richmont.94.US	----------	----------	----------	----------	----------	----------	----------	----------	----------	----------
345.96002726.96.SC.US	-----N----	----T-----	----------	----------	------V---	----------	----------	----------	----------	--A-------
Hamburg135.98.GE	-----N----	----T-----	----------	----------	------V---	---------H	----------	----------	----------	--A-------
004.95M_14.95.AU	-----N----	----T-----	----------	----------	------V---	----------	----------	----------	----------	--A-------
373.96019743.96.SC.US	-----N----	----T-----	----------	----------	------V---	----------	----------	----------	----------	--A-------
OBSM.112.99.DE	-----N----	----T-----	----------	----------	------V---	----------	----------	----------	----------	--A-------
384.96025046.96.FL.US	-----N----	----T-----	----------	----------	------V---	----------	----------	----------	----------	--A-------
416.97003156.96.LA.US	-----N----	----T-----	----------	----------	------V---	----------	----------	----------	----------	--A-------
Mora.97.SE	-----N----	----T-----	----------	----------	------V---	----------	----------	----------	----------	--A-------
Hamburg180.97.GE	-----N----	----T-----	----------	----------	------V---	----------	----------	----------	----------	--A-------
DenHaag015.00.NL	-----N----	----T-----	----------	----------	------V---	----------	----------	----------	----------	--A-------
Dresden153.97.GE	-----N----	----T-----	----------	----------	------V---	----------	----------	----------	----------	--A-------
358.96015107.96.FL.US	-----N----	----T-----	----------	----------	------V---	----------	----------	----------	----------	--A-------
WDVN016.00.NL	-----N----	----T-----	----------	----------	------V---	----------	----------	----------	----------	--A-------
Beeskow.124.00.DE	-----N----	----T-----	----------	----------	------V---	----------	----------	----------	----------	--A-------
BWLND.331.95.US	-----N----	----T-----	----------	----------	------V---	----------	-----A----	----------	----------	--A-------
Berlin.159.98.DE	-----N----	----T-----	----------	----------	------V---	----------	R---------	----------	----------	--A-------
Osaka.98.JPN	-----N----	----T-----	----------	----------	------V---	----------	----------	----------	----------	--A-T-----
Berlin.238.98.DE	-----N----	----T-----	----------	----------	------V---	----------	--G-------	----------	----------	--A-------
Hamburg189.97.GE	-----N----	----T-----	----------	----------	------V---	----------	----------	----------	-S--------	--A-------
FRBG057.97.GE	-----N----	----T-----	----------	----------	------V---	----------	----------	----------	-S--------	--A-------
Grimsby.98.UK	-----N----	----T-----	----------	----------	------V---	----------	----------	----------	----------	--A-------
345_2.96002737.96.SC.US	-----N----	----T-----	----------	----------	------V---	----------	----------	----------	----------	--A-------
364.96019537.96.AZ.US	-----N----	----T-----	----------	----------	------V---	----------	----------	----------	----------	--A-------
379.960984.96.AZ.US	-----N----	----T-----	----------	----------	------V---	----------	----------	----------	----------	--A-------
Dresden245.97.GE	-----N----	----T-----	----------	--------T-	------V---	----------	----------	----------	----------	--A-------
DOUG4770.01.AU	----------	----T-----	----------	--------G-	------V---	----------	--------T-	----------	----------	--A-------
ERL195.97.GE	-----N----	----T-----	----------	----------	------V---	----------	----------	----------	----------	--A-------
FRBG253.98.GE	-----N----	----T-----	----------	----------	----------	----------	----------	----------	----------	--A-------
Bochum024.98.GE	----------	----T-----	----------	----------	------V---	----------	----------	----------	----------	--A-------
Bochum026.97.GE	-----N----	----T-----	----------	----------	----------	----------	----------	----------	----------	--A-------
Narita104.02.JPN	-----N----	----T-----	----------	----------	------V---	----------	----------	----------	----------	--A-------
GII.4.97	-----N--S-	----T-----	----------	----------	------V---	----------	----------	----------	----------	--A-------
Sydney348.97O.AU	-----N----	----------	----------	----------	------V---	----------	----------	----------	--------P-	--A-------
DIJON171.96.FRA	-----N----	----T-----	----------	----------	----------	----------	----------	----------	----------	--A-------
FRBG024.97.GE	-----N----	----T-----	----------	----------	----------	----------	----------	----------	----------	--A-------
Hamburg236.97.GE	-----N----	----T-----	----------	----------	----------	----------	----------	----------	----------	--A-------
Dresden319.97.GE	-----N----	----T-----	----------	----------	----------	----------	----------	----------	----------	--A-------
Hamburg316.98.GE	-----N----	P---T-----	----------	----------	----------	----------	----------	----------	----------	--A-------
LDW.221.99.DE	-----N----	----T-----	----------	----------	------V---	----------	----------	----------	----------	--A-------
LDW.218.99.DE	-----N----	----T-----	----------	----------	------V---	----------	----------	----------	----------	--A-------
Berlin.495.00.DE	-----N----	----T-----	----------	----------	------V---	----------	----------	----------	----------	--A-------
MIBE.326.95.US	-----N----	----T-----	----------	----------	------V---	----------	----------	----------	----------	--A-------
366.96019554.96.ID.US	-----N----	----T-----	----------	----------	------V---	----------	----------	----------	----------	--A-------
Utrecht058.01.NL	-----N----	----T-----	----------	----------	------V---	---S------	----------	----------	----------	--A-------
Altenkirchen140.01.DE	-----N----	----T-----	----------	----------	------V---	----------	----------	----------	----------	--A-------
Schiedam018.01.NL	-----N--T-	----T-----	----------	---------R	------V---	----------	----------	----------	----------	--A-------
Frankfurt.Oder.170.99.DE	-----N----	----T-----	----------	----------	------V---	----------	----------	----------	----------	--A-------
Bochum272.98.GE	-----N----	----T-----	----------	----------	------V---	----------	----------	----------	----------	--A-------
Berlin.491.00.DE	-----N----	----T-----	----------	----------	------V---	----------	----------	----------	----------	--A-------
Dresden267.97.GE	-----N----	----T-----	----------	----------	------V---	-------LG-	W---------	----------	----------	--A-------
KNWH.130.00.DE	-----N----	----T-----	-------T--	----------	------V---	--G--I----	----------	----------	----------	--A-------
Dillingen259.01.DE	-----N----	----T-----	----------	----------	------V---	------I---	----------	----------	----------	--A-------
Bochum136.98.GE	-----N----	----T-----	----------	----------	------V---	----------	----------	----------	----------	--A-------
Bochum224.98.GE	-----N----	----T-----	----------	----------	------V---	----------	----------	----------	----------	--A-------
Dresden174.pUS_NorII.97.GE	-----N----	----T-----	----------	----------	------V---	----------	----------	----------	----------	--A-------
Bochum220.97.GE	-----N----	----T-----	----------	----------	------V---	----------	----------	----------	----------	--A-------
Bochum108.97.GE	-----N----	----T-----	----------	----------	------V---	----------	----------	----------	----------	--A-------
Bochum031.97.GE	-----N----	----T-----	----------	----------	------V---	----------	----------	----------	----------	--A-------
VA98387.98.US	-----N--S-	----T-----	----------	----------	------V---	----------	----------	----------	----------	--A-------
Bochum339.97.GE	-----N----	----T-----	----------	----------	------V---	----------	----------	----------	----------	--A-------
Tiel001.95.NL	-----N--T-	----T-----	----------	----------	------V---	---S------	----------	----------	----------	--A-------
Parkroyal.95.UK	-----NX---	----T----X	----------	----------	------V---	----------	--------K-	----------	----------	--A-------
Symgreen.95.UK	-----N----	----T-----	----------	----------	------V---	---S------	----------	----------	----------	--A-------
YURI.32073.02.JP	-----N----	----T-----	----------	----------	------V---	---S------	----------	----------	----------	--A-------
Hamburg139.97.GE	-----N----	----T-----	----------	----------	----------	----------	----------	----------	----------	--A-V-----
Hamburg137.97.GE	-----N----	----T-----	----------	----------	----------	----------	----------	----------	----------	--A-------
Hamburg048.97.GE	-----N----	----T-----	----------	----------	----------	----------	----------	----------	----------	--A-------
LWRDN043.00.NL	-----N----	----T-----	----------	----------	------V---	----------	----------	----------	----------	--A-------
GCanyon.02.US	----------	----T-----	----------	----------	------V---	----------	----------	----------	----------	--A-------
408.97003012.96.FL.US	-----N----	----T-----	----------	----------	------V---	----------	----------	----------	----------	--A-------
HoUSton.95.US	-----N----	----T-----	----------	----------	------V---	----------	----------	----------	----------	--A-------
Erfurt.007.00.DE	-----N----	----T-----	----------	----------	------V---	----------	----------	----------	----------	--A-------
OXF.B1S16.02.UK	-----N----	----T-----	----------	----------	------V---	----------	----------	----------	----------	--A-------
OXF.B1S21.02.UK	-----N----	----T-----	----------	----------	------V---	----------	----------	----------	----------	--A-------
OXF.B1S12.02.UK	-----N----	----T-----	----------	----------	------V---	----------	----------	----------	----------	--A-------
OXF.B1S11.02.UK	-----N----	----T-----	----------	----------	------V---	----------	----------	----------	----------	--A-------
OXF.B1S4.02.UK	-----N----	----T-----	----------	----------	------V---	----------	----------	----------	----------	--A-------
OXF.B1S2.02.UK	-----N----	----T-----	----------	----------	------V---	----------	----------	----------	----------	--A-------
OXF.B1S1.02.UK	-----N----	----T-----	----------	----------	------V---	----------	----------	----------	----------	--A-------
Warren.02.US	-----N----	----T-----	----------	----------	------V---	----------	----------	----------	----------	--A-------
OXF.B5S22.03.UK	-----N----	----T-----	----------	----------	------V---	----------	----------	----------	----------	--A-------
OXF.B2S10.02.UK	-----N----	----T-----	----------	----------	------V---	----------	----------	----------	----------	--A-------
OXF.B2S11.02.UK	-----N----	----T-----	----------	----------	------V---	----------	----------	----------	----------	--A-------
OXF.B2S14.02.UK	-----N----	----T-----	----------	----------	------V---	----------	----------	----------	----------	--A-------
OXF.B2S15.02.UK	-----N----	----T-----	----------	----------	------V---	----------	----------	----------	----------	--A-------
OXF.B2S17.02.UK	-----N----	----T-----	----------	----------	------V---	----------	----------	----------	----------	--A-------
OXF.B2S18.02.UK	-----N----	----T-----	----------	----------	------V---	----------	----------	----------	----------	--A-------
OXF.B2S20.02.UK	-----N----	----T-----	----------	----------	------V---	----------	----------	----------	----------	--A-------
OXF.B2S23.02.UK	-----N----	----T-----	----------	----------	------V---	----------	----------	----------	----------	--A-------
OXF.B2S24.02.UK	-----N----	----T-----	----------	----------	------V---	----------	----------	----------	----------	--A-------
OXF.B2S25.02.UK	-----N----	----T-----	----------	----------	------V---	----------	----------	----------	----------	--A-------
OXF.B2S26.02.UK	-----N----	----T-----	----------	----------	------V---	----------	----------	----------	----------	--A-------
OXF.B3S4.03.UK	-----N----	----T-----	----------	----------	------V---	----------	----------	----------	----------	--A-------
OXF.B3S16.03.UK	-----N----	----T-----	----------	----------	------V---	----------	----------	----------	----------	--A-------

```
OXF.B3S18.03.UK                -----N---- ----T----- ---------- ---------- ------V--- ---------- ---------- ---------- ---------- --A-------
OXF.B3S19.03.UK                -----N---- ----T----- ---------- ---------- ------V--- ---------- ---------- ---------- ---------- --A-------
OXF.B3S20.03.UK                -----N---- ----T----- ---------- ---------- ------V--- ---------- ---------- ---------- ---------- --A-------
OXF.B6S3.03.UK                 -----N---- ----T----- ---------- ---------- ------V--- ---------- ---------- ---------- ---------- --A-------
OXF.B6S5.03.UK                 -----N---- ----T----- ---------- ---------- ------V--- ---------- ---------- ---------- ---------- --A-------
OXF.B5S23.03.UK                -----N---- ----T----- ---------- ---------- ------V--- ---------- ---------- ---------- ---------- --A-------
OXF.B6S2.03.UK                 -----N---- ----T----- ---------- ---------- ------V--- ---------- ---------- ---------- ---------- --A-------
Witney.B7S2.03.UK              -----N---- ----T----- ---------- ---------- ------V--- ---------- ---------- ---------- ---------- --A-------
OXF.B2S16.02.UK                -----N---- ----T----- ---------- ---------- ------V--- ---------- ---------- ---------- ---------- --A-------
Banbury.B9S23.03.UK            -----N---- ----T----- ---------- ---------- ------V--- ---------- ---------- ---------- ---------- --A-------
CHPNRT.03.UK                   -----N---- ----T----- ---------- ---------- ------V--- ---------- ---------- ---------- ---------- --A-------
Didcot.B9S2.03.UK              -----N---- ----T----- ---------- ---------- ------V--- ---------- ---------- ---------- ---------- --A-------
OXF.B6S4.03.UK                 -----N---- ----T----- ---------- ---------- ------V--- ---------- ---------- ---------- ---------- --A-------
OXF.B6S6.03.UK                 -----N---- ----T----- ---------- ---------- ------V--- ---------- ---------- ---------- ---------- --A-------
DenHaag001.03.NL               -----N---- ----T----- ---------- ---------- ------V--- ---------- ---------- ---------- ---------- --A-------
WeertE022.02.NL                -----N---- ----T----- ---------- ---------- ------V--- ---------- ---------- ---------- ---------- --A-------
CS_G2.02.US                    -----N---- ----T----- ---------- ---------- ------V--- ---------- ---------- ---------- ---------- --A-------
Langen1061.02.DE               -----N---- ----T----- ---------- ---------- ------V--- ---------- ---------- ---------- ---------P --A-------
CS_D1.02.CAN                   -----N---- ----T----- ---------- ---------- ------V--- ---------- ---------- ---------- ---------- --A-------
CS_G4.02.US                    -----N---- ----T----- ---------- ---------- ------V--- ---------- ---------- ---------- ---------- --A-------
Germanton.02.US                -----N---- ----T----- ---------- ---------- ------V--- ---------- ---------- ---------- ---------- --A-------
CS_G102.US                     -----N---- ----T----- ---------- ---------- ------V--- ---------- ---------- ---------- ---------- --A-------
OXF.B5S7.02.UK                 -----N---- ----T----- ---------- ---------- ------V--- ---------- ---------- ---------- ---------- --A-------
OXF.B5S8.02.UK                 -----N---- ----T----- ---------- ---------- ------V--- ---------- ---------- ---------- ---------- --A-------
OXF.B5S9.02.UK                 -----N---- ----T----- ---------- ---------- ------V--- ---------- ---------- ---------- ---------- --A-------
OXF.B5S10.02.UK                -----N---- ----T----- ---------- ---------- ------V--- ---------- ---------- ---------- ---------- --A-------
GII.4.02a                      -----N---- ----T----- ---------- ---------- ------V--- ---------- ---------- ---------- ---------- --A-------
FHills.02.US                   -----N---- ----T----- ---------- ---------- ------V--- ---------- ---------- ---------- ---------- --A-------
Carlow.02.Ire                  -----N---- ----T----- ---------- ---------- ------V--- ---------- ---------- ---------- ---------- --A-------
OXF.B5S1.202.UK                -----N---- ----T----- ---------- ---------- ------V--- ---------- ---------- ---------- ---------- --A-------
OXF.B4S5.02.UK                 -----N---- ----T----- ---------- ---------- ------V--- ---------- ---------- ---------- ---------- --A-------
OXF.B4S6.02.UK                 -----N---- ----T----- ---------- ---------- ------V--- ---------- ---------- ---------- ---------- --A-------
OXF.B4S4.02.UK                 -----N---- ----T----- ---------- ---------- ------V--- ---------- ---------- ---------- ---------- --A-------
OXF.B4S7.02.UK                 -----N---- ----T----- ---------- ---------- ------V--- ---------- ---------- ---------- ---------- --A-------
OXF.B4S2.02.UK                 -----N---- ----T----- ---------- ---------- ------V--- ---------- ---------- ---------- ---------- --A-------
OXF.B8S5.02.UK                 -----N---- ----T----- ---------- ---------- ------V--- ---------- ---------- ---------- ---------- --A-------
OXF.B4S1.02.UK                 -----N---- ----T----- ---------- ---------- ------V--- ---------- ---------- ---------- ---------- --A-------
Middelburg007.04.NL            -----N---- ----T----- ---------- ---------- ------V--- ---------- ---------- ---------- ---------- --A-------
Anchorage.02.US                -----N---- ----T----- ---------- ---------- ------V--- ---------- ---------- ---------- ---------- --A-------
MD_04.04.US                    -----N---- ----T----- ---------- ---------- ------V--- ---------- ---------- ---------- ---------- --A-------
Haarl457.03.NL                 -----N--T- ----T----- ---------- ---------- ------V--- ---------- ---------- ---------- ---------- --A-------
Heerlen003.03.NL               -----N--T- ----T----- ---------- ---------- ------V--- ---------- ---------- ---------- ---------- --A-------
OXF.B5S13.02.UK                -----N---- ----T----- ---------- ---------- ------V--- ---------- ---------- ---------- ---------- --A-------
OXF.B5S19.02.UK                -----N---- ----T----- ---------- ---------- ------V--- ---------- ---------- ---------- ---------- --A-------
Elsloo012.04.NL                -----N--T- ----T----- ---------- ---------- ------V--- ---------- ---------- ---------- ---------- --A-------
DenHelder003.04.NL             -----N---- ----T----- ---------- ---------- ------V--- ---------- ---------- ---------- ---------- --A-------
GII.4.02                       -----N---- ----T----- ---------- ---------- ------V--- ---------- ---------- ---------- ---------- --A-------
Apeldoorn023.03.NL             -----N---- ---------- ---------- ---------- ------V--- ---------- ---------- ---------- ---------- --A-------
GA04.04.US                     -----N---- ----T----- ---------- ---------- ------V--- ---------- ---------- ---------- ---------- --A-------
Lanzhou.35666.02.CHN           -----N--SS ----T----- ---------- ---------- ------V--- ---------- ---------- ---------- ---------- --A-------
Liempde048.04.NL               -----N--T- ----T----- ---------- ---------- ------V--- ---------- ---------- ---------- ---------- --A-------
G2.4.04.NL                     -----N--T- ----T----- ---------- ---------- ------V--- ---------- ---------- ---------- ---------- --A-------
Almelo039.04.NL                -----N--T- ----T----- ---------- ---------- ------V--- ---------- ---------- ---------- ---------- --A-------
DenBosch028.04.NL              -----N--T- ----T----- ---------- ---------- ------V--- ---------- ---------- ---------- ---------- --A-------
Tilburg059.04.NL               -----N--T- ----T----- ---T------ ---------- ------V--- ---------- ---------- ---------- ---------- --A-------
Nijmegen083.04.NL              -----N--T- ----T----- ---------- ---------- ------V--- ---------- ---------- ---------- ---------- --A-------
Dongen46.06.NL                 -----N--T- ----T----- ---------- ---------- ------V--- ---------- ---------- ---------- ---------- --A-------
Hunter284E.04O.AU              -----N--T- ----T----- ---------- ---------- ------V--- ---------- ---------- ---------- ---------- --A-------
GII.4.04                       -----N--T- ----T----- ---------- ---------- ------V--- ---------- ---------- ---------- ---------- --A-------
DenHaag54.06.NL                -----N--T- ----T----- ---------- ---------- ------V--- ---------- ---------- ---------- ---------- --A-------
Yerseke38.06.NL                ---------- ----T----- ---------- ---------- ------V--- ---------- ---------- ---------- ---------- --A-------
Terneuzen70.06.NL              ---------- ----T----- ---------- ---------- ------V--- ---------- ---------- ---------- ---------- --A----S--
Sakai.04_179.05.JP             -----N--S- ----T----- ---------- ---------- ------V--- ---------- ---------- ---------- ---------- --A-------
Beijing.CR2932.04.CHN          -----N--S- ----T----- ---------- ---------- ------V--- ---------- ---------- ---------- ---------- --A-------
GII.4.05                       -----N--S- ----T----- ---------- ---------- ------V--- ---------- ---------- ---------- ---------- --A-------
Chiba.04_1050.05.JP            -----N--S- ----T----- ---------- ---------- ------V--- ---T------ ---------- ---------- ---------- --A-------
GNZH.NVgz01.CHN                ----PN--S- ----T----- ---------- ---------- ------V--- ---------- ---------- ---------- ---------- --A-------
Beijing.CR2905.04.CHN          -----N--S- ----T----- ---------- ---------- ------V--- ----SS---- ---------D ---------- ---------- --A-------
GNZH.NV_VP1.06.CHN             ----TN--S- ----T----- ---------- ---------- ------V--- ---------- ---------- ---------- ---------- --A-------
Ehime.05_30.05.JP              -----N--S- ----T----- ---------- ---------- ------V--- ---------- ---------- ---------- ---------- --A-------
QM2_CS.04.US                   -----N--S- ----T----- ---------- ---------- ------V--- ---------- ---------- ---------- ---------- --A-------
DenHaag89.06.NL                -----N---- ---------- ---------- ---------- ------V--- ---------- ---------- ---------- ---------- --A-------
Kobe034.06.JP                  -----N---- ---------- ---------- ---------- ----T-V--- ---------- ---------- ---------- ---------- --A-----H-
Nijmegen115.06.NL              -----N---- ---------- ---------- ---------- ------V--- ---------- ---------- ---------- ---------- --A------- 100


Camberwell.94.AU               GGFEVQVILA GNAFTAGKVI FAAVPPNFPT EGLSPSQVTM FPHIIVDVRQ LEPVLIPLPD VRNNFYHYNQ SHDSTLKLIA MLYTPLRANN AGDDVFTVSC
MD145_12.87.US                 ---------- ---------- ---------- ---------- ---------- ---------- ---------- ---------- ---------- ----------
GII.4.87                       ---------- ---------- ---------- ---------- ---------- ---------- ---------- ---------- ---------- ----------
MD134_7.87.US                  ---------- ---------- ---------- ---------- ---------- ---------- ---------- ---------- ---------- ----------
Lord.93.UK                     ---------- ---------- ---------- ---------- ---------- ---------- ---------- AN-------- ---------- ----------
UK3_17.12700.92.GB             ---------- ---------- ---------- ---------- ---------- ---M------ ---------- AN-------- T--------- ----------
EmmenE006.02.NL                ---------- ---------- ---------- ---------- ---------- ---------- ---------- A--------- ---------- ----------
Richmont.94.US                 ---------- --------I- ---------- ---------- ---V------ ---------- ---------- A--P-I---- ---------- ----------
345.96002726.96.SC.US          ---------- --------I- ---------- ---------- ---------- ---------- ---------- -N---I---- ---------- ----------
Hamburg135.98.GE               ---------- --------I- ---------- ---------- ---------- ---------- ---------- -N---I---- ---------- ----------
004.95M_14.95.AU               ---------- --------I- ---------- ---------- ---------- ---------- ---------- -N---I---- ---------- ----------
373.96019743.96.SC.US          ---------- --------I- ---------- ---------- ---------- ---------- ---------- -N---I---- ---------- ----------
OBSM.112.99.DE                 ---------- --------I- ---------- ---------- ---------- ---------- ---------- -N---I---- ---------- ----------
384.96025046.96.FL.US          ---------- --------I- ---------- ---------- ---------- ---------- ---------- -N---I---- ---------- ----------
416.97003156.96.LA.US          ---------- --------I- ---------- ---------- ---------- ---------- ---------- -N---I---- ---------- ----------
Mora.97.SE                     ---------- --------I- ---------- ---------- ---------- ---------- ---------- -N---I---- ---------- ----------
Hamburg180.97.GE               ---------- --------I- ---------- ---------- ---------- ---------- ---------- -N---I---- ---------- ----------
DenHaag015.00.NL               ---------- --------I- ---------- ---------- ---------- ---------- ---------- -N---I---- ---------- ----------
Dresden153.97.GE               ---D------ --------I- ---------- ---------- ---------- ---------- ---------- -N---I---- ---------- ----------
358.96015107.96.FL.US          ---------- --------I- ---------- ---------- ---------- ---------- ---------- -N---I---- ---------- ----------
WDVN016.00.NL                  ---------- --------I- ---------- ---------- ---------- ---------- ---------- -N---I---- ---------- --------F-
Beeskow.124.00.DE              ---------- --------I- ---------- ---------- ---------- ---------- ---------- -N---I---- ---------- ----------
BWLND.331.95.US                ---------- --------I- ---------- ---------- ---------- ---------- ---------- -N---I---- ---------- -------A--
Berlin.159.98.DE               ---------- --------I- ---------- ---------- ---------- ---------- ---------- -N---I---- -P-------- ----------
Osaka.98.JPN                   ---------- --------I- ---------- ---------- ---------- ---------- ---------- -N---I---- ---------- ----------
```

```
Berlin.238.98.DE             ---------- -S-S----I- ---------- ---------- ---------- ---------- ---------- -N---I---- ---------- ----------
Hamburg189.97.GE             ---------- --------I- ---------- ---------- ---------- ---------- ---------- -N---I---- ---------- ----------
FRBG057.97.GE                ---------- --------I- ---------- ---------- ---------- ---------- ---------- -N---I---- ---------- ----------
Grimsby.98.UK                ---------- --------I- ---------A ---------- ---------- ---------- ---------- -N---I---- ---------- ----------
345_2.96002737.96.SC.US      ---------- --------I- ---------- ---------- ---------- ---------- ---------- -S---I---- ---------- ----------
364.96019537.96.AZ.US        ---------- --------I- ---------- ---------- ---------- ---------- ---------- -N---I---- ---------- ----------
379.960984.96.AZ.US          ---------- --------I- ---------- ---------- ---------- ---------- ---------- -N---I---- ---------- ----------
Dresden245.97.GE             ---------- --------I- ---------- ---------- ---------- ---------- ---------- -N---I---- ---------- ----------
DOUG4770.01.AU               ---------- --------I- ---------- ---------- ---------- ---------- A--------- -N---I---- ---------- ----------
ERL195.97.GE                 ---------- --------I- ---------- ---------- ---------- ---------- ---------- -N---I---- ---------- ----------
FRBG253.98.GE                ---------- --------I- ---------- ---------- ---------- ---------- ---------- -N---I---- ---------- ----------
Bochum024.98.GE              ---------- --------I- ---------- ---------- ---------- ---------- ---------- -N---I---- ---------- ----------
Bochum026.97.GE              ---------- --------I- ---------- ---------- ---------- ---------- ---S------ -N---I---- ---------- ----------
Narita104.02.JPN             ---------- --------I- ---------- ---------- ---------- ---------- ---------- -N---I---- ---------- ----------
GII.4.97                     ---------- --------I- ---------- ---------- ---------- ---------- ---------- -N---I---- ---------- ----------
Sydney348.97O.AU             ---------- --------I- ---------- ---------- ---------- -D-----S-- ---------- -N---I---- ---------- ----------
DIJON171.96.FRA              ---------- --------I- ---------- ---------- ---------- ---------- ---------- -N---I---- ---------- ----------
FRBG024.97.GE                ---------- --------I- ---------- ---------- ---------- ---------- ---------- -N---I---- ---------- ----------
Hamburg236.97.GE             ---------- --------I- ---------- ---------- ---------- ---------- ---------- -N---I---- ---------- ----------
Dresden319.97.GE             ---------- --------I- ---------- ---G------ ---------- ---------- ---------- -N---I---- ---------- ----------
Hamburg316.98.GE             ---------- --------I- ---------- ---------- ---------- ---------- ---------- -N---I---- ---------- ----------
LDW.221.99.DE                ---------- --------I- ---------- ---------- ---------- ---------- ---------- -N---I---- ---------- ----------
LDW.218.99.DE                ---------- --------I- ---------- ---------- ---------- ---------- ---------- -N---I---- ---------- ----------
Berlin.495.00.DE             ---------- --------I- ---------- ---------- ---------- ---------- ---------- -N---I---- ---------- ----------
MIBE.326.95.US               ---------- --------I- ---------- ---------- --------G- ---------- ---------- -N---I---- ---------- ----------
366.96019554.96.ID.US        ---------- --------I- ---------- ---------- ---------- ---------- ---------- -N---I---- ---------- ----------
Utrecht058.01.NL             ---------- --------I- ---------- ---------- ---------- ---------- ---------- -N---I---- ---------- ----------
Altenkirchen140.01.DE        ---------- --------I- ---------- ---------- ---------- ---------- ---------- -N---I---- ---------- ----------
Schiedam018.01.NL            ---------- --------I- ---------- ---------- ---------- ---------- ---------- -N---I---- ---------- ----------
Frankfurt.Oder.170.99.DE     ---------- --------I- ---------- ---------- ---------- ---------- ---------- -N---I---- ---------- -------A--
Bochum272.98.GE              ---------- --------I- ---------- ---------- ---------- ---------- ---------- -N---I---- ---------- ----------
Berlin.491.00.DE             ---------- --------I- ---------- ---------- ---------- ---------- ---------- -N---I---- ---------- ----------
Dresden267.97.GE             ---------- --------I- ---------- ---------- ---------- ---------- ---------- -N---I---- ---------- ----------
KNWH.130.00.DE               ---------- --------I- ---------- ---------V ---------- ---------- -----H---- -N---I---- ---------- ----------
Dillingen259.01.DE           ---------- --------I- ---------- ---------- ---------- ---------- ---------- -N---I---- ---------- ----------
Bochum136.98.GE              ---------- ---------- ---------- ---------- ---------- ---------- ---------- -N---I---- ---------- ----------
Bochum224.98.GE              ---------- ---------- ---------- ---------- ---------- ---------- ---------- -N---I---- ---------- ----------
Dresden174.pUS_NorII.97.GE   ---------- ---------- ---------- ---------- ---------- ---------- ---------- -N---I---- ------K--- ----------
Bochum220.97.GE              ---------- R--------- ---------- ---------- ---------- ---------- ---------- -N---I---- ---------- ----------
Bochum108.97.GE              ---------- ---------- ---------- ---------- ---------- ---------- ---------- -N---I---- ---------- ----------
Bochum031.97.GE              ---------- ---------- ---------- ---------- ---------- ---------- ---------- -N---I---- ---------- ----------
VA98387.98.US                ---------- ---------- ---------- ---------- ---------- ---------- ---------- -N---I---- ---------- ----------
Bochum339.97.GE              ---------- --------I- ---------- ---------- ---------- ---------- G--------- -N---I---- ---------- ----------
Tiel001.95.NL                ---------- --------I- ---------- ---------- ---------- ---------- ---------- -N---I---- ---------- ----------
Parkroyal.95.UK              ---------- --------I- ---------- ---------- -----I---- ---------- ---------- -N---I---- ---------- ----------
Symgreen.95.UK               --L----V-- --------I- ---------- ---------- ---------- ---------- ---------- -N---I---- ---------D ----------
YURI.32073.02.JP             ---------- --------I- ---------- ---------- ---------- ---------- ---------- -N---I---- ---------- ----------
Hamburg139.97.GE             ---------- --------I- ---------- ---------- ---------- ---------- ---------- -N---I---- ---------- ----------
Hamburg137.97.GE             ---------- ---S----I- ---------- ---------- ---------- ---------- ---------- -N---I---- ---------- ----------
Hamburg048.97.GE             ---------- --------I- ---------- ---------- ---------- ---------- ---------- -N---I---- ---------- ----------
LWRDN043.00.NL               ---------- --------I- ---------- ---------- ---------- ---------- ---------- -N---I---- ---------- ----------
GCanyon.02.US                ------T--- --------I- ---------- ---------- ---------- ---------- ---------- -N-P-I---- ---------- ----------
408.97003012.96.FL.US        ---------- --------I- ---------- ---------- ---------- ---------- ---------- -N---I---- ---------- ----------
HoUSton.95.US                ---------- --------I- ---------- ---------- ---------- ---------- ---------- -N---I---- ---------- ----------
Erfurt.007.00.DE             ---------- --------I- ---------- ---------- ---------- ---------- ---------- -N---I---- ---------- ----------
OXF.B1S16.02.UK              ---------- --------I- ---------- ---------- ---------- ---------- ---------- -N-P-I---- ---------- --E-------
OXF.B1S21.02.UK              ---------- --------I- ---------- ---------- ---------- ---------- ---------- -N-P-I---- ---------- --E-------
OXF.B1S12.02.UK              ---------- --------I- ---------- ---------- ---------- ---------- ---------- -N-P-I---- ---------- --E-------
OXF.B1S11.02.UK              ---------- --------I- ---------- ---------- ---------- ---------- ---------- -N-P-I---- ---------- --E-------
OXF.B1S4.02.UK               ---------- --------I- ---------- ---------- ---------- ---------- ---------- -N-P-I---- ---------- --E-------
OXF.B1S2.02.UK               ---------- --------I- ---------- ---------- ---------- ---------- ---------- -N-P-I---- ---------- --E-------
OXF.B1S1.02.UK               ---------- --------I- ---------- ---------- ---------- ---------- ---------- -N-P-I---- ---------- --E-------
Warren.02.US                 ---------- --------I- ---------- ---------- ---------- ---------- ---------- -N-P-I---- ---------- --E-------
OXF.B5S22.03.UK              ---------- --------I- ---------- ---------- ---------- ---------- ---------- -N-P-I---- ---------- --E-------
OXF.B2S10.02.UK              ---------- --------I- ---------- ---------- ---------- ---------- ---------- -N-P-I---- ---------- --E-------
OXF.B2S11.02.UK              ---------- --------I- ---------- ---------- ---------- ---------- ---------- -N-P-I---- ---------- --E-------
OXF.B2S14.02.UK              ---------- --------I- ---------- ---------- ---------- ---------- ---------- -N-P-I---- ---------- --E-------
OXF.B2S15.02.UK              ---------- --------I- ---------- ---------- ---------- ---------- ---------- -N-P-I---- ---------- --E-------
OXF.B2S17.02.UK              ---------- --------I- ---------- ---------- ---------- ---------- ---------- -N-P-I---- ---------- --E-------
OXF.B2S18.02.UK              ---------- --------I- ---------- ---------- ---------- ---------- ---------- -N-P-I---- ---------- --E-------
OXF.B2S20.02.UK              ---------- --------I- ---------- ---------- ---------- ---------- ---------- -N-P-I---- ---------- --E-------
OXF.B2S23.02.UK              ---------- --------I- ---------- ---------- ---------- ---------- ---------- -N-P-I---- ---------- --E-------
OXF.B2S24.02.UK              ---------- --------I- ---------- ---------- ---------- ---------- ---------- -N-P-I---- ---------- --E-------
OXF.B2S25.02.UK              ---------- --------I- ---------- ---------- ---------- ---------- ---------- -N-P-I---- ---------- --E-------
OXF.B2S26.02.UK              ---------- --------I- ---------- ---------- ---------- ---------- ---------- -N-P-I---- ---------- --E-------
OXF.B3S4.03.UK               ---------- --------I- ---------- ---------- ---------- ---------- ---------- -N-P-I---- ---------- --E-------
OXF.B3S16.03.UK              ---------- --------I- ---------- ---------- ---------- ---------- ---------- -N-P-I---- ---------- --E-------
OXF.B3S18.03.UK              ---------- --------I- ---------- ---------- ---------- ---------- ---------- -N-P-I---- ---------- --E-------
OXF.B3S19.03.UK              ---------- --------I- ---------- ---------- ---------- ---------- ---------- -N-P-I---- ---------- --E-------
OXF.B3S20.03.UK              ---------- --------I- ---------- ---------- ---------- ---------- ---------- -N-P-I---- ---------- --E-------
OXF.B6S3.03.UK               ---------- --------I- ---------- ---------- ---------- ---------- ---------- -N-P-I---- ---------- --E-------
OXF.B6S5.03.UK               ---------- --------I- ---------- ---------- ---------- ---------- ---------- -N-P-I---- ---------- --E-------
OXF.B5S23.03.UK              ---------- --------I- ---------- ---------- ---------- ---------- ---------- -N-P-I---- ---------- --E-------
OXF.B6S2.03.UK               ---------- --------I- ---------- ---------- ---------- ---------- ---------- -N-P-I---- ---------- --E-------
Witney.B7S2.03.UK            ---------- --------I- ---------- ---------- ---------- ---------- ---------- -N-P-I---- ---------- --E-------
OXF.B2S16.02.UK              ---------- --------I- ---------- ---------- ---------- ---------- ---------- -N-P-I---- ---------- --E-------
Banbury.B9S23.03.UK          ---------- --------I- ---------- ---------- ---------- ---------- ---------- -N-P-I---- ---------- --E-------
CHPNRT.03.UK                 ---------- --------I- ---------- ---------- ---------- ---------- ---------- -N-P-I---- ---------- --E-------
Didcot.B9S2.03.UK            ---------- --------I- ---------- ---------- ---------- ---------- ---------- -N-P-I---- ---------- --E-------
OXF.B6S4.03.UK               ---------- --------I- ---------- ---------- ---------- ---------- ---------- -N-P-I---- ---------- --E-------
OXF.B6S6.03.UK               ---------- --------I- ---------- ---------- ---------- ---------- ---------- -N-P-I---- ---------- --E-------
DenHaag001.03.NL             ---------- --------I- ---------- ---------- ---------- ---------- ---------- -N-P-I---- ---------- --E-------
WeertE022.02.NL              ---------- --------I- ---------- ---------- ---------- ---------- ---------- -N-P-I---- ---------- --E-------
CS_G2.02.US                  ---------- --------I- ---------- ---------- ---------- ---------- ---------- -N-P-I---- ---------- --E-------
Langen1061.02.DE             ---------- --------I- ---------- ---------- ---------- ---------- ---------- -N-P-I---- ---------- --E-------
CS_D1.02.CAN                 ---------- --------I- ---------- ---------- ---------- ---------- ---------- -N-P-I---- ---------- --E-------
CS_G4.02.US                  ---------- --------I- ---------- ---------- ---------- ---------- ---------- -N-P-I---- ---------- --E-------
Germanton.02.US              ---------- --------I- ---------- ---------- ---------- ---------- ---------- -N-P-I---- ---------- --E-------
CS_G102.US                   ---------- --------I- ---------- ---------- ---------- ---------- ---------- -N-P-I---- ---------- --E-------
OXF.B5S7.02.UK               ---------- --------I- ---------- ---------- ---------- ---------- ---------- -N-P-I---- ---------- --E-------
OXF.B5S8.02.UK               ---------- --------I- ---------- ---------- ---------- ---------- ---------- -N-P-I---- ---------- --E-------
OXF.B5S9.02.UK               ---------- --------I- ---------- ---------- ---------- ---------- ---------- -N-P-I---- ---------- --E-------
```

```
OXF.B5S10.02.UK             ---------- --------I- ---------- ---------- ---------- ---------- ---------- -N-P-I---- ---------- --E-------
GII.4.02a                   ---------- --------I- ---------- ---------- ---------- ---------- ---------- -N-P-I---- ---------- --E-------
FHills.02.US                ---------- --------I- ---------- ---------- ---------- ---------- ---------- LN-P-I---- ---------- --E-------
Carlow.02.Ire               ---------- --------I- ---------- ---------- ---V------ ---------- ---------- -N-P-I---- ---------- --E-------
OXF.B5S1.202.UK             ---------- --------I- ---------- ---------- ---------- ---------- ---------- -N-P-I---- ---------- --E-------
OXF.B4S5.02.UK              ---------- --------I- ---------- ---------- ---------- ---------- ---------- -N-P-I---- ---------- --E-------
OXF.B4S6.02.UK              ---------- --------I- ---------- ---------- ---------- ---------- ---------- -N-P-I---- ---------- --E-------
OXF.B4S4.02.UK              ---------- --------I- ---------- ---------- ---------- ---------- ---------- -N-P-I---- ---------- --E-------
OXF.B4S7.02.UK              ---------- --------I- ---------- ---------- ---------- ---------- ---------- -N-P-I---- ---------- --E-------
OXF.B4S2.02.UK              ---------- --------I- ---------- ---------- ---------- ---------- ---------- -N-P-I---- ---------- --E-------
OXF.B8S5.02.UK              ---------- --------I- ---------- ---------- ---------- ---------- ---------- -N-P-I---- ---------- --E-------
OXF.B4S1.02.UK              ---------- --------I- ---------- ---------- ---------- ---------- ---------- -N-P-I---- ---------- --E-------
Middelburg007.04.NL         ---------- --------I- ------S--- ---------- ---------- ---------- ---------- -N-P-I---- ---------- --E-------
Anchorage.02.US             ---------- --------IV ---------- ---------- ---------- ---------- ---------- -N-P-I---- ---------- --E-------
MD_04.04.US                 ---------- --------I- ---------- ---------- ---------- ---------- ---------- -N-P-I---- ---------- --E-------
Haarl457.03.NL              ---------- --------I- ---------- ---------- ---------- ---------- ---------- -N-P-I---- ---------- --E-------
Heerlen003.03.NL            -------V-- --------I- ---------- ---------- ---------- ---------- ----L----- -N-P-I---- ---------- --E-------
OXF.B5S13.02.UK             ---------- --------I- ---------- ---------- ---------- ---------- ---------- -N-P-I---- ---------- --E-------
OXF.B5S19.02.UK             ---------- --------I- ---------- ---------- ---------- ---------- ---------- -N-P-I---- ---------- --E-------
Elsloo012.04.NL             ---------- --------I- ------S--- ---------- ---------- ---------- ---------- -N-P-I---- ---------- --E-------
DenHelder003.04.NL          ---------- --------I- ---------- ---------- ---------- ---------- ---------- -N-P-I---- ---------- --E-------
GII.4.02                    ---------- --------I- ---------- ---------- ---------- ---------- ---------- -N-P-I---- ---------- --E-------
Apeldoorn023.03.NL          ---------- --------I- ---------- ---------- ---------- ---------- ---------- -N---I---- ---------- --E-------
GA04.04.US                  ---------- --------I- ---------- ---------- ---------- ---------- ---------- -N---I---- ---------- ----------
Lanzhou.35666.02.CHN        ---------- --------I- ---------- ---------- ---------- ---------- ---------- -N-P-I---- ---------- ----------
Liempde048.04.NL            ---------- --------I- ---------- ---------- ---------- ---------- ---------- -N-P-I---- ---------- ----------
G2.4.04.NL                  ---------- --------I- ---------- ---------- ---------- ---------- ---------- -N-P-I---- ---------- ----------
Almelo039.04.NL             ---------- --------I- ---------- ---------- ---------- ---------- ---------- -N-P-I---- ---------- ----------
DenBosch028.04.NL           ---------- --------I- ---------- ---------- ---------- ---------- ---------- -N-P-I---- ---------- ----------
Tilburg059.04.NL            ---------- --------I- ---------- ---------- ---------- ---------- ---------- -N-P-I---- -------T-- ----------
Nijmegen083.04.NL           ---------- --------I- ---------- ---------- ---------- ---------- ---------- -N-P-I---- ---------- ----------
Dongen46.06.NL              ---------- --------I- ---------- ---------- ---------- ---------- ---------- -N-P-I---- ---------- ----------
Hunter284E.04O.AU           ---------- --------I- ---------- -V-------- ---------- ---------- ----L----- -N-P-IR--- ---------- ----------
GII.4.04                    ---------- --------I- ---------- -V-------- ---------- ---------- ----L----- -N-P-IR--- ---------- ----------
DenHaag54.06.NL             ---------- --------I- ---------- ---------- ---------- ---------- ---------- -N-P-IR--- ---------- ----------
Yerseke38.06.NL             ---------- --------I- ---------- ---------- ---------- ---------- ---------- -N-P-I---- ---------- ----------
Terneuzen70.06.NL           ---------- --------I- ---------- ---------- ---------- ---------- ---------- -N-P-I---- ---------- ----------
Sakai.04_179.05.JP          ---------- --------I- ---------- ---------- ---------- ---------- ---------- -N---I---- ---------- ----------
Beijing.CR2932.04.CHN       ---------- --------I- ---------- ---------- ---------- ---------- ---------- -N---I---- ---------- ----------
GII.4.05                    ---------- --------I- ---------- ---------- ---------- ---------- ---------- -N---I---- ---------- ----------
Chiba.04_1050.05.JP         ---------- --------I- ---------- ---------- ---------- ---------- ---------- -N---I---- ---------- ----------
GNZH.NVgz01.CHN             ---------- --------I- ---------- ---------- ---------- ---------- ---------- -N---I---- ---------- ----------
Beijing.CR2905.04.CHN       ---------- --------I- ---A------ ---------- ------G--- ---------- ---------- -N---I---- ---------- ----------
GNZH.NV_VP1.06.CHN          -----E---- --------I- ---------- ---------- ---------- ---------- ---------- -N---I---- ---------- ----------
Ehime.05_30.05.JP           ---------- --------I- ---------- ---------- ---------- ---------- ---------- -N---I---- ---------- ----------
QM2_CS.04.US                ---------- --------I- ---------- ---------- ---------- ---------- ---------- -N-P-I---- ---------- ----------
DenHaag89.06.NL             ---------- --------I- ---------- ---------- ---------- ---------- ---------- -N---I---- ---------- --E-------
Kobe034.06.JP               ---------- --------I- ---------- ---------- ---------- ---------- ---------- -N---I---- ---------- --E-------
Nijmegen115.06.NL           ---------- --------I- ---------- ---------- ---------- ---------- ---------- -N---I---- ---------- --E------- 200


Camberwell.94.AU            RVLTRPSPDF DFIFLVPPTV ESRTKPFTVP ILTVEEMSNS RFPIPLEKLY TGPSSAFVVQ PQNGRCTTDG VLLGTTQLSA VNICNFRGDV THIVGSHDYT
MD145_12.87.US              ---------- ---------- ---------- ---------- ---------- ---------- ---------- ---------- ---------- ----------
GII.4.87                    ---------- ---------- ---------- ---------- ---------- ---------- ---------- ---------- ---------- ----------
MD134_7.87.US               ---------- ---------- ---------- ---------- ---------- ---------- ---------- ---------- ---------- ----------
Lord.93.UK                  ---------- ---------- ---------- V--------- ---------- ---------- ---------- ---------- ---------- ---A------
UK3_17.12700.92.GB          ---------- ---------- ---------- V--------- ---------- ---------- ---------- ---------- ---------- A--A------
EmmenE006.02.NL             ---------- ---------- ---------- V------T-- ---------- ------I--- ---------- ---------- ----T----- ---A--RN--
Richmont.94.US              ---------- ---------- ---------- ---------- ---------- -----D---- ---------- ---------- ------K--- ---A--R--R
345.96002726.96.SC.US       ---------- ---------- ---------- ---------- ---------- ---------- ---------- ---------- ----T----- ---A------
Hamburg135.98.GE            ---------- ---------- ---------- ---------- ---------- ---------- ---------- ---------- ----T----- ---A------
004.95M_14.95.AU            ---------- ---------- ---------- ---------- ---------- ---------- ---------- ---------- ----T----- ---A------
373.96019743.96.SC.US       ---------- ---------- ---------- ---------- ---------- ---------- ---------- ---------- ----T----- ---A------
OBSM.112.99.DE              ---------- ---------- ---------- ---------- ---------- ---------- ---------- ---------- ----T----- ---A------
384.96025046.96.FL.US       ---------- ---------- ---------- ---------- ---------- ---------- ---------- ---------- ----T----- ---A------
416.97003156.96.LA.US       ---------- ---------- ---------- ---------- ---------- ---------- ---------- ---------- ----T----- ---A------
Mora.97.SE                  ---------- ---------- ---------- ---------- ---------- ---------- ---------- ---------- ----T----- ---A------
Hamburg180.97.GE            ---------- ---------- ---------- ---------- ---------- ---------- ---------- ---------- ----T----- ---A------
DenHaag015.00.NL            ---------- ---------- ---------- ---------- ---------- ---------- ---------- ---------- ----T----- ---A------
Dresden153.97.GE            ---------- ---------- ---------- ---------- ---------- ---------- ---------- ---------- ----T----- ---A------
358.96015107.96.FL.US       ---------- ---------- ---------- ---------- ---------- ---------- ---------- ---------- ----T----- ---A------
WDVN016.00.NL               ---------- ---------- ---------- ---------- ---------- ---------- ---------- ---------- ----T----- ---A------
Beeskow.124.00.DE           ---------- ---------- ---------- ---------- ---------- -------I-- ---------- ---------- ----T----- ---A------
BWLND.331.95.US             ---------- ---------- ---------- ---------- ---------- ---------- --S------- ---------- ----T----- ---A------
Berlin.159.98.DE            ---------- ---------- ---------- ---------- ---------- ---------- ---------- ---------- ----T----- ---A------
Osaka.98.JPN                ---------- ---------- ---------- ---------- ---------- ---------- ---------- ---------- ----T----- ---A------
Berlin.238.98.DE            ---------- ---------- ---------- ---------- ---------- ---------- ---------- ---------- ----T----- ---A------
Hamburg189.97.GE            ---------- ---------- ---I------ ---------- ---------- ---------- ---------- ---------- ----T----- ---A------
FRBG057.97.GE               ---------- ---------- ---------- ---------- ---------- ---------- ---------- ---------- ----T----- ---A------
Grimsby.98.UK               ---------- ---------- ---------- ---------- ---------- ---------- ---------- -X-------- ----T----- ---A------
345_2.96002737.96.SC.US     ---------- ---------- ---------- ---------- ---------- ---------- ---------- ---------- ----T----- ---A------
364.96019537.96.AZ.US       ---------- ---------- ---------- ---------- ---------- ---------- ---------- ---------- ----T----- ---A------
379.960984.96.AZ.US         ---------- ---------- ---------- ---------- ---------- ---------- ---------- ---------- ----T----- ---A------
Dresden245.97.GE            ---------- ---------- ---------- ---------- ---------- ---------- ---------- ---------- ----T----- ---A------
DOUG4770.01.AU              ---------- ---------- ---------- ---------- ---------- -----V---- ---------- ---------- ----T----- ---A------
ERL195.97.GE                ---------- ---------- ---------- ---------- ---------- ---------- ---------- ---------- ----T----- ---A------
FRBG253.98.GE               ---------- ---------- ---------- ---------- ---------- ---------- ---------- ---------- ----T----- ---A------
Bochum024.98.GE             ---------- ---------- ---------- ---------- ---------- ---------- ---------- ---------- ----T----- ---A------
Bochum026.97.GE             ---------- ---------- ---------- ---------- ---------- ---------- ---------- ---------- ----T----- ---A------
Narita104.02.JPN            ---------- ---------- ---------- ---------- ---------- ---------- ---------- ---------- ----T----- ---A------
GII.4.97                    ---------- ---------- ---------- ---------- ---------- ---------- ---------- ---------- ----T----I ---A------
Sydney348.97O.AU            ---------- ---------- ---------- ---------- ---------- ---------- ---------- ---------- ----T----- ---A------
DIJON171.96.FRA             ---------- ---------- ---------- ---------- ---------- ---------- ---------- ---------- ----T----- ---A------
FRBG024.97.GE               ---------- ---------- --------A- ---------- ---------- ---------- ---------- ---------- ----T----- ---A------
Hamburg236.97.GE            ---------- ---------- ---I------ ---------- ---------- ---------- ---------- ---------- ----T----- ---A------
Dresden319.97.GE            ---------- ---------- ---------- ---------- ---V------ ---------- ---------- ---------- ----T----- ---A--R---
Hamburg316.98.GE            ---------- ---------- ---------- ---------- ---------- ---------- ---------- ---------- ----T----- ---A------
LDW.221.99.DE               ---------- ---------- --------I- ---------- ---------- ---------- ---------- ---------- ----T---N- I--A------
LDW.218.99.DE               ---------- ---------- --------I- ---G------ ---------- ---------- ---------- ---------- ----T-K-N- I--A------
Berlin.495.00.DE            ---------- ---------- ---------- ---------- ---------- ---------- ---------- ---------- ----T----- ---A------
MIBE.326.95.US              ---------- ---------- ---------- ---------- ---------- ---------- ---------- ---------- ----T----- ---A------
```

```
366.96019554.96.ID.US        ---------- ---------- ---------- ---------- ---------- ---------- ---------- ---------- ----T----- ---A------
Utrecht058.01.NL             ---------- ---------- ---------- ---------- ---V------ ---------- ---------- ---------- ----T----- ---A------
Altenkirchen140.01.DE        ---------- ---------- ---------- ---------- ---V------ ---------- ---------- ---------- ----T----- ---A------
Schiedam018.01.NL            ---------- ---------- ---------- ---------- ---V------ ---------- ---------- ---------- ----T----- ---A------
Frankfurt.Oder.170.99.DE     ---------- ---------- ---------- ---------- ---------- ---------- ---------- ---------- ----T----- ---A------
Bochum272.98.GE              ---------- ---------- ---------- ---------- ---------- ---------- ---------- ---------- ----T----- ---A------
Berlin.491.00.DE             ---------- ---------- ---------- ---------- ---------- ---------- ---------- ------R--- ----T----- ---A--R---
Dresden267.97.GE             ---------- ---------- ---------- ---------- ---------- ---------- ---------- ---------- ----T----- ---A------
KNWH.130.00.DE               ---------- ---------I ---------- ---------- ---------- ---------- ---------- ---------- ----T----- ---A------
Dillingen259.01.DE           ---------- ---------- ---------- ---------- ---------- ---------- ---------- ---------- ----T----- ---A------
Bochum136.98.GE              ---------- ---------- ---------- ---------- ---------- ---------- ---------- ---------- ----T----- ---A------
Bochum224.98.GE              ---------- ---------- ---------- ---------- ---------- ---------- ---------- ---------- ----T----- ---A------
Dresden174.pUS_NorII.97.GE   ---------- ---------- ---------- ---------- ---------- ---------- ---------- ---------- ----T----- ---A------
Bochum220.97.GE              ---------- ---------- ---------- ---------- ---------- ---------- ---------- ---------- ----T----- ---A------
Bochum108.97.GE              ---------- ---------- ---------- ---------- ---------- ---------- ---------- ---------- ----T-G--- ---A------
Bochum031.97.GE              ---------- ---------- ---------- ---------- ---------- ---------- ---------- ---------- ----T----- ---A------
VA98387.98.US                ---------- ---------- ---------- ---------- ---------- ---------- ---------- ---------- ----T----- ---A-----I
Bochum339.97.GE              ---------- ---------- ---------- ---------- ---------- ---------- ---------- ---------- ----T----- ---A------
Tiel001.95.NL                ---------- ---------- ---------- ---------- ---------- ---------- ---------- ---------- ----T----- ---A------
Parkroyal.95.UK              ---------- ---------- ---------- ---------- ---------- ---------- ---------- ---------- ----TY---- ---A------
Symgreen.95.UK               ---------- ---------- ---------- ---------- ---------- ---------- ---------- ---------- ----T----- ---A------
YURI.32073.02.JP             ---------- ---------- ---------- ---------- ---------- ---------- ---------- ---------- ----TL---- ---A------
Hamburg139.97.GE             ---------- ---------- ---------- ---------- ---------- ---------- ---------- ---------- ----T----- ---A------
Hamburg137.97.GE             ---------- ---------- ---I------ ---------- ---------- ---------- ---------- ---------- ----T----- ---A------
Hamburg048.97.GE             ---------- ---------- ---------- ---------- ---------- ---------- ---------- ---------- ----T----- ---A------
LWRDN043.00.NL               ---------- ---------- ---------- ---------- ---------F ---------- ---------- ---------- ----T----- ---A------
GCanyon.02.US                ---------- ---------- ---------- ---------- ---------- ---------- ---------- ---------- ----T----- ---A------
408.97003012.96.FL.US        ---------- ---------- ---------- ---------- ---------- ---------- ---------- ---------- ----T----- ---A------
HoUSton.95.US                ---------- ---------- ---------- ---------- ---------- ---------- ---------- ---------- ----T----- ---A------
Erfurt.007.00.DE             ---------- ---------- ---S------ ---------- ---------- ---------- ---------- ---------- ----T----- ---T------
OXF.B1S16.02.UK              ---------- ---------- ---------- -------T-- ---------F ----G----- ---------- ---------P ----T----- ---A-T-N--
OXF.B1S21.02.UK              ---------- ---------- ---------- -------T-- ---------F ----G----- ---------- ---------P ----T----- ---A-T-N--
OXF.B1S12.02.UK              ---------- ---------- ---------- -------T-- ---------F ----G----- ---------- ---------P ----T----- ---A-T-N--
OXF.B1S11.02.UK              ---------- ---------- ---------- -------T-- ---------F ----G----- ---------- ---------P ----T----- ---A-T-N--
OXF.B1S4.02.UK               ---------- ---------- ---------- -------T-- ---------F ----G----- ---------- ---------P ----T----- ---A-T-N--
OXF.B1S2.02.UK               ---------- ---------- ---------- -------T-- ---------F ----G----- ---------- ---------P ----T----- ---A-T-N--
OXF.B1S1.02.UK               ---------- ---------- ---------- -------T-- ---------F ----G----- ---------- ---------P ----T----- ---A-T-N--
Warren.02.US                 ---------- ---------- ---------- -------T-- ---------F ----G----- ---------- ---------P ----T----- ---A-T-N--
OXF.B5S22.03.UK              ---------- ---------- ---------- -------T-- ---------F ----G----- ---------- ---------P ----T----- ---A-T-N--
OXF.B2S10.02.UK              ---------- ---------- ---------- -------T-- ---------F ----G----- ---------- ---------P ----T----- ---A-T-N--
OXF.B2S11.02.UK              ---------- ---------- ---------- -------T-- ---------F ----G----- ---------- ---------P ----T----- ---A-T-N--
OXF.B2S14.02.UK              ---------- ---------- ---------- -------T-- ---------F ----G----- ---------- ---------P ----T----- ---A-T-N--
OXF.B2S15.02.UK              ---------- ---------- ---------- -------T-- ---------F ----G----- ---------- ---------P ----T----- ---A-T-N--
OXF.B2S17.02.UK              ---------- ---------- ---------- -------T-- ---------F ----G----- ---------- ---------P ----T----- ---A-T-N--
OXF.B2S18.02.UK              ---------- ---------- ---------- -------T-- ---------F ----G----- ---------- ---------P ----T----- ---A-T-N--
OXF.B2S20.02.UK              ---------- ---------- ---------- -------T-- ---------F ----G----- ---------- ---------P ----T----- ---A-T-N--
OXF.B2S23.02.UK              ---------- ---------- ---------- -------T-- ---------F ----G----- ---------- ---------P ----T----- ---A-T-N--
OXF.B2S24.02.UK              ---------- ---------- ---------- -------T-- ---------F ----G----- ---------- ---------P ----T----- ---A-T-N--
OXF.B2S25.02.UK              ---------- ---------- ---------- -------T-- ---------F ----G----- ---------- ---------P ----T----- ---A-T-N--
OXF.B2S26.02.UK              ---------- ---------- ---------- -------T-- ---------F ----G----- ---------- ---------P ----T----- ---A-T-N--
OXF.B3S4.03.UK               ---------- ---------- ---------- -------T-- ---------F ----G----- ---------- ---------P ----T----- ---A-T-N--
OXF.B3S16.03.UK              ---------- ---------- ---------- -------T-- ---------F ----G----- ---------- ---------P ----T----- ---A-T-N--
OXF.B3S18.03.UK              ---------- ---------- ---------- -------T-- ---------F ----G----- ---------- ---------P ----T----- ---A-T-N--
OXF.B3S19.03.UK              ---------- ---------- ---------- -------T-- ---------F ----G----- ---------- ---------P ----T----- ---A-T-N--
OXF.B3S20.03.UK              ---------- ---------- ---------- -------T-- ---------F ----G----- ---------- ---------P ----T----- ---A-T-N--
OXF.B6S3.03.UK               ---------- ---------- ---------- -------T-- ---------F ----G----- ---------- ---------P ----T----- ---A-T-N--
OXF.B6S5.03.UK               ---------- ---------- ---------- -------T-- ---------F ----G----- ---------- ---------P ----T----- ---A-T-N--
OXF.B5S23.03.UK              ---------- ---------- ---------- -------T-- ---------F ----G----- ---------- ---------P ----T----- ---A-T-N--
OXF.B6S2.03.UK               ---------- ---------- ---------- -------T-- ---------F ----G----- ---------- ---------P ----T----- ---A-T-N--
Witney.B7S2.03.UK            ---------- ---------- ---------- -------T-- ---------F ----G----- ---------- ---------P ----T----- ---A-T-N--
OXF.B2S16.02.UK              ---------- ---------- ---------- -------T-- ---------F ----G----- ---------- ---------P ----T----- ---A-T-N--
Banbury.B9S23.03.UK          ---------- ---------- ---------- -------T-- ---------F ----G----- ---------- ---------P ----T----- ---A-T-N--
CHPNRT.03.UK                 ---------- ---------- ---------- -------T-- ---------F ----G----- ---------- ---------P ----T----- ---A-T-N--
Didcot.B9S2.03.UK            ---------- ---------- ---------- -------T-- ---------F ----G----- ---------- ---------P ----T----- ---A-T-N--
OXF.B6S4.03.UK               ---------- ---------- ---------- -------T-- ---------F ----G----- ---------- ---------P ----T----- ---A-T-N--
OXF.B6S6.03.UK               ---------- ---------- ---------- -------T-- ---------F ----G----- ---------- ---------P ----T----- ---A-T-N--
DenHaag001.03.NL             ---------- ---------- ---------- -------T-- ---------F ----G----- ---------- ---------P ----T----- ---A-T-N--
WeertE022.02.NL              ---------- ---------- ---------- -------T-- ---------F ----G----- ---------- ---------P ----TL---- ---A-T-N--
CS_G2.02.US                  ---------- ---------- ---------- -------T-- ---------F ----G----- ---------- ---------P ----T----- ---A-T-N--
Langen1061.02.DE             ---------- ---------- ---------- -------T-- ---------F ----G----- ---------- ---------P ----T----- ---A-T-N--
CS_D1.02.CAN                 ---------- ---------- ---------- -------T-- ---------F ----G----- ---------- ---------P ----T----- ---A-T-N--
CS_G4.02.US                  ---------- ---------- ---------- -------T-- ---------F ----G----- ---------- ---------P ----T----- ---A-T-N--
Germanton.02.US              ---------- ---------- ---------- -------T-- ---------F ----G----- ---------- ---------P ----T----- ---A-T-N--
CS_G102.US                   ---------- ---------- ---------- -------T-- ---------F ----G----- ---------- ---------P ----T----- ---A-T-N--
OXF.B5S7.02.UK               ---------- ---------- ---------- -------T-- ---------F ----G----- ---------- ---------P ----T----- ---A-T-N--
OXF.B5S8.02.UK               ---------- ---------- ---------- -------T-- ---------F ----G----- ---------- ---------P ----T----- ---A-T-N--
OXF.B5S9.02.UK               ---------- ---------- ---------- -------T-- ---------F ----G----- ---------- ---------P ----T----- ---A-T-N--
OXF.B5S10.02.UK              ---------- ---------- ---------- -------T-- ---------F ----G----- ---------- ---------P ----T----- ---A-T-N--
GII.4.02a                    ---------- ---------- -----S---- -------T-- ---------F ----G----- ---------- ---------P ----T----- ---A-T-N--
FHills.02.US                 ---------- ---------- ---------- -------T-- ---------F ----G----- ---------- ---------P ----T----- ---A-T-N--
Carlow.02.Ire                ---------- ---------- ---------- -------T-- ---------F ----G----- ---------- ---------P ----T----- ---A-T-N--
OXF.B5S1.202.UK              ---------- ---------- ---------- -------T-- ---------F ----G----- ---------- ---------P ----A----- ---A-T-N--
OXF.B4S5.02.UK               ---------- ---------- ---------- -------T-- ---------F ----G----- ---------- ---------P ----A----- ---A-T-N--
OXF.B4S6.02.UK               ---------- ---------- ---------- -------T-- ---------F ----G----- ---------- ---------P ----A----- ---A-T-N--
OXF.B4S4.02.UK               ---------- ---------- ---------- -------T-- ---------F ----G----- ---------- ---------P ----A----- ---A-T-N--
OXF.B4S7.02.UK               ---------- ---------- ---------- -------T-- ---------F ----G----- ---------- ---------P ----A----- ---A-T-N--
OXF.B4S2.02.UK               ---------- ---------- ---------- -------T-- ---------F ----G----- ---------- ---------P ----A----- ---A-T-N--
OXF.B8S5.02.UK               ---------- ---------- ---------- -------T-- ---------F ----G----- ---------- ---------P ----A----- ---A-T-N--
OXF.B4S1.02.UK               ---------- ---------- ---------- -------T-- ---------F ----G----- ---------- ---------P ----A----- ---A-T-N--
Middelburg007.04.NL          ---------- ---------- ---------- -------T-- ---------F ----G----- ---------- ---------P ----T----- ---A-T-N--
Anchorage.02.US              ---------- ---------- ---------- -------T-- ---------F ---------- ---------- ---------P ----T----- ---A-T-N--
MD_04.04.US                  ---------- ---------- ---------- -------T-- ---------F ----G----- ---------- ---------P ----T----- ---ADT-N--
Haarl457.03.NL               ---------- ---------- ---------- -------T-- ---------F ----G----- ---------- ---------P ----T----- ---A-T-N--
Heerlen003.03.NL             ---------- ---------- ---------- ---G---T-- ---------F ----G----- ---------- --------LP ----T----- ---A-T-N--
OXF.B5S13.02.UK              ---------- ---------- ---------- -------T-- ---------F ----G----- ---------- ---------P ----T----- ---A-T-N--
OXF.B5S19.02.UK              ---------- ---------- ---------- -------T-- ---------F ----G----- ---------- ---------P ----T----- ---A-T-N--
Elsloo012.04.NL              ---------- ---------- ---------- -------T-- ---------F ----G----- ---------- ---------P ----T----- ---A-T-N--
DenHelder003.04.NL           ---------- ---------- ---------- -------T-- ---------F ----G----- ---------- ---------P ----T----- ---A-T-N--
GII.4.02                     ---------- ---------- ---------- -------T-- ---------F ----G----- ---------- ---------P ----T----- ---A-T-N--
Apeldoorn023.03.NL           ---------- ---------- ---------- -------T-- ---------F ----G----- ---------- ---------P ----T----- ---A-T-N--
GA04.04.US                   ---------- ---------- ---------- -------T-- ---------F ----G----- ---------- ---------P ----T----- ---A-TRN--
Lanzhou.35666.02.CHN         ---------- ---------- ---------- -------T-- ---------F ---------- ---------- ---------P ----T----- ---A-T----
```

```
Liempde048.04.NL             ---------- ---------- -------S-- -------T-- ---------F ---------- ---------- ---------P ----T----- ---A-TQN--
G2.4.04.NL                   ---------- ---------- -------S-- -------T-- ---------F ---------- ---------- ---------P ----T----- ---A-TQN--
Almelo039.04.NL              ---------- ---------- -------S-- -------T-- ---------F ---------- ---------- ---------P ----T----- ---A-TQN--
DenBosch028.04.NL            ---------- ---------- -------S-- -------T-- ---------F ---------- ---------- ---------P ----T----- ---A-TQN--
Tilburg059.04.NL             ---------- ---------- -------S-- -------T-- ---------F ---------- ---------- ---------P ----T----- ---A-TQN--
Nijmegen083.04.NL            ---------- ---------- -------S-- -------T-- ---------F ---------- ---------- ---------P ----T----- ---A-TQN--
Dongen46.06.NL               ---------- ---------- -------S-- -------T-- ---------F ---------- ---------- ---------P ----T----- ---A-TQN--
Hunter284E.04O.AU            ---------- --M------- -------S-- -------T-- ---------F ---------- ---------- ---------P ----T----- ---A-AQN--
GII.4.04                     ---------- --M------- -------S-- -------T-- ---------F ---------- ---------- ---------P ----T----- ---A-AQN--
DenHaag54.06.NL              ---------- ---------- -------S-- -------T-- ---------F ---------- ---------- ---------P ----T----- ---A-TQN--
Yerseke38.06.NL              ---------- ---------- -------S-- -------T-- ---------F ---------- ---------- ---------P ----T----- ---A-TQE--
Terneuzen70.06.NL            ---------- ---------- -------S-- -------T-- ---------F ---------- ---------- ---------P ----T----- ---A-TQE--
Sakai.04_179.05.JP           ---------- ---------- ---------- -------T-- ---------F ---------- ---------- ---------P ----T----- ---P-TRT-R
Beijing.CR2932.04.CHN        ---------- ---------- ---------- -------T-- ---------F ---------- ---------- ---------P ----T----- ---P-TRT-R
GII.4.05                     ---------- ---------- ---------- -------T-- ---------F ---------- ---------- ---------P ----T----- ---P-TRT-R
Chiba.04_1050.05.JP          ---------- ---------- ---------- -------T-- ---------F ---------- ---------- ---------P ----T----- I--P-TRT-R
GNZH.NVgz01.CHN              ---------- ---------- ---------- -------T-- ---------F ---------- ---------- ---------P -S--T----- ---P-TRI-R
Beijing.CR2905.04.CHN        ---------- ---------- ---------- -------T-- -------NCS RV-AVPLSSN .HKMQ----- ---------P ----T----- ---P-TRT-R
GNZH.NV_VP1.06.CHN           ---------- ---------- ---------- -------T-- ---------F ------L--- ---------- ---------P ----T----- ---S-TRT-R
Ehime.05_30.05.JP            ---------- ---------- ---------- -------T-- ---------F ---------- ---------- ---------P ----T----- ---S-TRT-R
QM2_CS.04.US                 ---------- ---------- ---------- -------T-- ---------F ---------- ---------- ---------P ----T----- ---P-TRT-R
DenHaag89.06.NL              ---------- ---------- ---------- -------T-- ---------F ----G----- ---------- ---------P ----T----- ---A--RN--
Kobe034.06.JP                ---------- ---------- ---------- -------T-- ---------F ----G----- ---------- ---------P ----T----- ---A--RN--
Nijmegen115.06.NL            ---------- ---------- ---------- -------T-- ---------F ----G----- ---------- ---------P ----T----- ---A--RN-- 300


Camberwell.94.AU             MNLASQNWSN YDPTEEIPAP LGTPDFVGKI QGLLTQTTRA DGSTRAHKAT VSTGSVHFTP KLGSVQFTTD TNNDFQTGQN TKFTPVGVIQ DGD.HHQNEP
MD145_12.87.US               ---------- ---------- ---------- ---------- ---------- ---------- ---------- ---------- ---------- ---.------
GII.4.87                     ---------- ---------- ---------- ---------- ---------- ---------- ---------- ---------- ---------- ---.------
MD134_7.87.US                ---V------ ---------- ---------- ---------- ---------- ---------- ---------- ---------- ---------- ---.------
Lord.93.UK                   ---------- ---------- ---------- ---------- ---------- ---------- ---------- ------A--- ---------- ---.------
UK3_17.12700.92.GB           ---------- ---------- ---------- ---------- --L------- ---------- ---------- ------A--- ---------- ---.------
EmmenE006.02.NL              --------N- ---------- ---------- ---------- ---------- ---------- -------S-- -D----P--- ---------- ---.R-----
Richmont.94.US               ---V----N- ---------- ------Q--- --------G- ---------- ----AAN--- ---------- -D-------- ---------- --S.------
345.96002726.96.SC.US        --------N- ---------- ---------- --M------E ---------- ---------- ------Y--- ---------- ---------- --N.N-----
Hamburg135.98.GE             --------NS ---------- ---------- --M------E ---------- ---------- ------Y--- ---------- ---------- --N.N-----
004.95M_14.95.AU             --------N- ---------- ---------- --M------E ---------- ---------- ------Y--- ---------- ---------- --N.N-----
373.96019743.96.SC.US        --------N- ---------- ---------- --M------E ---------- ---------- ------Y--- ---------- ---------- --N.N-----
OBSM.112.99.DE               --------N- ---------- ---------- --M------E ---------- ---------- ------Y--- ---------- ---------- --N.N-----
384.96025046.96.FL.US        --------N- ---------- ---------- --M------E ---------- ---------- ------Y--- ---------- ---------- --N.N-----
416.97003156.96.LA.US        --------N- ---------- ---------- --M------E ---------- ---------- ------Y--- ---------- ---------- --N.N-----
Mora.97.SE                   --------N- ---------- ---------- --M------E ---------- ---------- ------Y--- ---------- ---------- --N.N-----
Hamburg180.97.GE             --------N- ---------- ---------- --M------E ---------- ---------- ------Y--- ---------- ---------- --N.N-----
DenHaag015.00.NL             --------N- ---------- ---------- --M------E ---------- ---------- ------Y--- ---------- ---------- --N.N-----
Dresden153.97.GE             --------N- ---------- ---------- --M------E ---------- ---------- ------Y--- ---------- ---------- --N.N-----
358.96015107.96.FL.US        --------N- ---------- ---------- --M------E ---------- ---------- ------Y--- ---------- ---------- --N.N-----
WDVN016.00.NL                --------N- ---------- ---------- --M------E ---------- ---------- ------Y--- ---------- ---------- --N.N-----
Beeskow.124.00.DE            --------N- ---------- ---------- --M------E ---------- ---------- ------Y--- ---------- ---------- --N.N-----
BWLND.331.95.US              --------N- ---------- ---------- --M------E ---------- ---------- ------Y--- ---------- ---------- --N.N-----
Berlin.159.98.DE             --------N- ---------- ---------- --M------E ---------- ---------- R-----Y--- ---------- ---------- --N.N-----
Osaka.98.JPN                 --------N- ---------- ---------- --M------E ---------- ---------- ------Y--- ---------- ---------- --N.N-----
Berlin.238.98.DE             --------N- ---------- ---------- --M------E ---------- ---------- ------Y--- ---------- ---------- --N.N-----
Hamburg189.97.GE             --------N- ---------- ---------- --M------E ---------- ---------- ------Y--- ---------- ---------- --N.N-----
FRBG057.97.GE                --------N- ---------- ---------- --M------E ---------- ---------- ------Y--- ---------- ---------- --N.N-----
Grimsby.98.UK                --------N- ---------- ---------- --M------E ---------- --------I- ------Y--- ---------- X--------- --N.N-----
345_2.96002737.96.SC.US      --------N- ---------- ---------- --M------E ---------- ---------- ------Y--- ---------- ---------- --N.N-----
364.96019537.96.AZ.US        --------N- ---------- ---------- --M------E ---------- ---------- ------Y--- ------A--- ---------- --N.N-----
379.960984.96.AZ.US          --------N- ---------- ---------- --M------E ---------- ---------- ------Y--- ------A--- ---------- --N.N-----
Dresden245.97.GE             --------N- ---------- ---------- --M------E ---------- ---------- ------Y--- ---------- ---------- --N.N-----
DOUG4770.01.AU               --------N- ---------- ---------- --M------E ------T--- ---------- ------Y-A- ---------- ---------- --S.N-----
ERL195.97.GE                 --------N- ---------- ---------- --M------E ---------- ---------- ------Y--- ---------- ---------- --N.N-----
FRBG253.98.GE                --------N- ---------- ---------- --M------E ---------- ---------- ------Y--- ---------- ---------- --N.N-----
Bochum024.98.GE              --------N- ---------- ---------- --M------E ---------- ---------- ------Y--- --S------- ---------- --N.N-----
Bochum026.97.GE              --------N- ---------- ---------- --M------E ---------- ---------- ------Y--- --S------- ---------- --N.N-----
Narita104.02.JPN             ---------- ---------- ---------- --M------E ---------- ---------- ------Y--- ---------- ---------- --N.N-----
GII.4.97                     ---------- ---------- ---------- --M------E ---------- ---------- ------Y--- ---------- ---------- --G.N-----
Sydney348.97O.AU             --------I- ---------- ---------- --M------E ---------- ---------- ------Y--- ---------D ---------- --N.N-----
DIJON171.96.FRA              --------N- ---------- ---------- --M------E ---------- ---------- ------Y--- ---------- ---------- --N.N-----
FRBG024.97.GE                --------N- ---------- ---------- --M------E ---------- ---------- ------Y--- ---------- ---------- --N.N-----
Hamburg236.97.GE             --------N- ---------- ---------- --V------E ---------- ---------- ------Y--- ---------- ---------- --N.N-----
Dresden319.97.GE             --------N- ---------- ---------- --V------E ---------- ---------- ------Y--- -D-------- ---------- --N.N-----
Hamburg316.98.GE             --------N- ---------- ---------- --V------E ---------- ---------- ------Y--- ---------- ---------- --N.N-----
LDW.221.99.DE                --------N- ------T--- ---S------ ---------E ---------- ---------- ------Y--- ---------- ---------- --N.N-----
LDW.218.99.DE                --------N- ------T--- ---------- ---------E ---------- --A------- ------Y--- ---------- ---------- --N.N-----
Berlin.495.00.DE             --------N- ---------- ---------- --V------E ---------- ---------- ------Y--- ---N------ ---------- --N.N-----
MIBE.326.95.US               --------N- ---------- ---------- --V------E ---------- ---------- ------Y--- ---------- ---------- --N.N-----
366.96019554.96.ID.US        --------N- ---------- ---------- --V-----KE ---------- ---------- ------Y--- ---------- ---------- --N.N-----
Utrecht058.01.NL             --------N- ---------- ---------- --V------E ---------- ---------- ------Y--- ---------- ---------- --S.N-----
Altenkirchen140.01.DE        --------N- ---------- ---------- --V------E ---------- ---------- ------Y--- ---------- ---------- --S.N-----
Schiedam018.01.NL            --------N- ---------- ---------- --V------E ---------- ---------- ------Y--- ---------- ---------- --S.N-----
Frankfurt.Oder.170.99.DE     --------N- ---------- ---------- --V------E ---------- ---------- ------Y--- ---------- ---------- --S.N-----
Bochum272.98.GE              --------N- ---------- ---------- --V-S----E ---------- ---------- ------Y--- ---------- ---------- --G.N-----
Berlin.491.00.DE             --------N- ---------- ---------- --V------E ---------- ---------- ------Y--- ---------- ---------- --N.N-----
Dresden267.97.GE             --------N- ---------- ---------- --V------E ---------- ---------- ------Y--- -S-------- ---------- --N.N-----
KNWH.130.00.DE               --------N- ---------- ---------- --V------E ---------- ---------- ------Y--- -S-------- ---------- --N.N-----
Dillingen259.01.DE           --------N- ---------- -----L---- --M------E ---------- ---------- ------Y--- -S-------- ---------- --N.N-----
Bochum136.98.GE              --------N- ---------- ---------- --M------E ---------- ---------- ------Y--- ---------- ---------- --N.N-----
Bochum224.98.GE              --------N- ---------- ---------- --M------E ---------- ---------- ------Y--- ---------- ---------- --N.N-----
Dresden174.pUS_NorII.97.GE   --------N- ---------- ---------- --M------E ---------- ---------- ------Y--- ---------- ---------- --N.N-----
Bochum220.97.GE              --------N- ---------- ---------- --M------E ---------- ---------- ------Y--- ---------- ---------- --N.N-----
Bochum108.97.GE              --------N- ---------- ---------- --M------E ---------- ---------- ------Y--- ---------- ---------- ---.N-----
Bochum031.97.GE              --------N- ---------- ---------- --T------E ---------- ---------- ------Y--- ---------- ---------- --N.N-----
VA98387.98.US                --------N- ---------- ---------- --M------E ---------- ----T----- ------Y--- ---------- ---------- --N.N-----
Bochum339.97.GE              --------N- ---------- ---------- --T------E ---------- ---------- ----A-Y--- ---------- ---------- --N.N-----
Tiel001.95.NL                --------N- ---------- ---------- --V------G ---------- ---------- ------Y--- ---------- ---------- ---.N-----
Parkroyal.95.UK              --------N- ---------- ---------- --M------G ---------- ---------- ------Y--- ---------- ---------- --N.N-----
Symgreen.95.UK               --------N- ---------- ---------- --M-S----G -D-------- ---------- ------Y--- -----E---- ---------- --N.N-----
YURI.32073.02.JP             --------N- ---------- ---------- --M-S----G ---------- ---------- ------Y--- -----E---- ---------- --N.N-----
Hamburg139.97.GE             --------N- ---------- ---------- --M------E ---------- ---------- ------Y--- ---------- ---------- --N.N-----
Hamburg137.97.GE             --------N- ---------- ---------- --M------E ---------- -----A---- ------Y--- ---------- ---------- --N.N-----
Hamburg048.97.GE             --------N- ---------- ---------- --I------E ---------- ---------- ------Y--- ---------- ---------- --N.N-----
```

```
LWRDN043.00.NL           --------N- ---------- ---------- --M------E ---------- ---------- ------Y--- ---------- ---------- --N.N-----
GCanyon.02.US            --------N- ---------- ---------- --M------E ---------- ---------- ------Y--- ------A--- ---------- --N.N-----
408.97003012.96.FL.US    --------N- ---------- ---------- --M------G ---------- ---------- ---------- ---------- --------T- ---.N-----
HoUSton.95.US            --------N- ---------- ---------- --M------G ---------- ---------- ---------- ---------- ---------- ---.N-----
Erfurt.007.00.DE         --------N- ---------- ---------- --M------G -----G---- ---------- ----I----- -----E---- ---------- --SSA-----
OXF.B1S16.02.UK          --------N- ---------- --------R- --M------G -----G---- ----D----- ----I--N-- -----E---- -R------V- --NGT-----
OXF.B1S21.02.UK          --------N- ---------- --------R- --M------G -----G---- ----D----- ----I--N-- -----E---- -R------V- --NGT-----
OXF.B1S12.02.UK          --------N- ---------- --------R- --M------G -----G---- ----D----- ----I--N-- -----E---- -R------V- --NGT-----
OXF.B1S11.02.UK          --------N- ---------- --------R- --M------G -----G---- ----D----- ----I--N-- -----E---- -R------V- --NGT-----
OXF.B1S4.02.UK           --------N- ---------- --------R- --M------G -----G---- ----D----- ----I--N-- -----E---- -R------V- --NGT-----
OXF.B1S2.02.UK           --------N- ---------- --------R- --M------G -----G---- ----D----- ----I--N-- -----E---- -R------V- --NGT-----
OXF.B1S1.02.UK           --------N- ---------- --------R- --M------G -----G---- ----D----- ----I--N-- -----E---- -R------V- --NGT-----
Warren.02.US             --------N- ---------- --------R- --M------G -----G---- ----D----- ----I--N-- -----E---- -R------V- --NGT-----
OXF.B5S22.03.UK          --------N- ---------- --------R- --M------G -----G---- ----D----- ----I--N-- -----E---- -R------V- --NGT-----
OXF.B2S10.02.UK          --------N- ---------- --------R- --M------G -----G---- ----D----- ----I--N-- -----E---- -R------V- --NGT-----
OXF.B2S11.02.UK          --------N- ---------- --------R- --M------G -----G---- ----D----- ----I--N-- -----E---- -R------V- --NGT-----
OXF.B2S14.02.UK          --------N- ---------- --------R- --M------G -----G---- ----D----- ----I--N-- -----E---- -R------V- --NGT-----
OXF.B2S15.02.UK          --------N- ---------- --------R- --M------G -----G---- ----D----- ----I--N-- -----E---- -R------V- --NGT-----
OXF.B2S17.02.UK          --------N- ---------- --------R- --M------G -----G---- ----D----- ----I--N-- -----E---- -R------V- --NGT-----
OXF.B2S18.02.UK          --------N- ---------- --------R- --M------G -----G---- ----D----- ----I--N-- -----E---- -R------V- --NGT-----
OXF.B2S20.02.UK          --------N- ---------- --------R- --M------G -----G---- ----D----- ----I--N-- -----E---- -R------V- --NGT-----
OXF.B2S23.02.UK          --------N- ---------- --------R- --M------G -----G---- ----D----- ----I--N-- -----E---- -R------V- --NGT-----
OXF.B2S24.02.UK          --------N- ---------- --------R- --M------G -----G---- ----D----- ----I--N-- -----E---- -R------V- --NGT-----
OXF.B2S25.02.UK          --------N- ---------- --------R- --M------G -----G---- ----D----- ----I--N-- -----E---- -R------V- --NGT-----
OXF.B2S26.02.UK          --------N- ---------- --------R- --M------G -----G---- ----D----- ----I--N-- -----E---- -R------V- --NGT-----
OXF.B3S4.03.UK           --------N- ---------- --------R- --M------G -----G---- ----D----- ----I--N-- -----E---- -R------V- --NGT-----
OXF.B3S16.03.UK          --------N- ---------- --------R- --M------G -----G---- ----D----- ----I--N-- -----E---- -R------V- --NGT-----
OXF.B3S18.03.UK          --------N- ---------- --------R- --M------G -----G---- ----D----- ----I--N-- -----E---- -R------V- --NGT-----
OXF.B3S19.03.UK          --------N- ---------- --------R- --M------G -----G---- ----D----- ----I--N-- -----E---- -R------V- --NGT-----
OXF.B3S20.03.UK          --------N- ---------- --------R- --M------G -----G---- ----D----- ----I--N-- -----E---- -R------V- --NGT-----
OXF.B6S3.03.UK           --------N- ---------- --------R- --M------G -----G---- ----D----- ----I--N-- -----E---- -R------V- --NGT-----
OXF.B6S5.03.UK           --------N- ---------- --------R- --M------G -----G---- ----D----- ----I--N-- -----E---- -R------V- --NGT-----
OXF.B5S23.03.UK          --------N- ---------- --------R- --M------G -----G---- ----D----- ----I--N-- -----E---- -R------V- --NGT-----
OXF.B6S2.03.UK           --------N- ---------- --------R- --M------G -----G---- ----D----- ----I--N-- -----E---- -R------V- --NGT-----
Witney.B7S2.03.UK        --------N- ---------- --------R- --M------G -----G---- ----D----- ----I--N-- -----E---- -R------V- --NGT-----
OXF.B2S16.02.UK          --------N- ---------- --------R- --M------G -----G---- ----D----- ----I--N-- -----E---- -R------V- --NGT-----
Banbury.B9S23.03.UK      --------N- ---------- --------R- --M------G -----G---- ----D----- ----I--N-- -----E---- -R------V- --NGT-----
CHPNRT.03.UK             --------N- ---------- --------R- --M------G -----G---- ----D----- ----I--N-- -----E---- -R------V- --NGT-----
Didcot.B9S2.03.UK        --------N- ---------- --------R- --M------G -----G---- ----D----- ----I--N-- -----E---- -R------V- --NGT-----
OXF.B6S4.03.UK           --------N- ---------- --------R- --M------G -----G---- ----D----- ----I--N-- -----E---- -R------V- --NGT-----
OXF.B6S6.03.UK           --------N- ---------- --------R- --M------G -----G---- ----D----- ----I--N-- -----E---- -R------V- --NGT-----
DenHaag001.03.NL         --------N- ---------- --------R- --M------G -----G---- ----D----- -------N-- -----E---- -R------V- --NGT-----
WeertE022.02.NL          --------N- ---------- --------R- --V------G -----G---- ----D----- -------N-- -----EI--- -R------V- --NGT-----
CS_G2.02.US              --------N- ---------- --------R- --M------G -----G---- ----D----- ----I--N-- -----E---- --------V- --NGT-----
Langen1061.02.DE         --------N- -G-------- --------R- --M------G -----G---- ----D----- ----I--N-- -----E---- --------V- --NGT-----
CS_D1.02.CAN             --------N- ---------- --------R- --M------G -----G---- ----D----- ----I--N-- -----E---- --------V- --NGT-----
CS_G4.02.US              --------N- ---------- --------R- --M------G -----G---- ----D----- ----I--N-- -----E---- --------V- --NGT-----
Germanton.02.US          --------N- ---------- --------R- --M------G -----G---- ----D----- ----I--N-- -----E---- --------V- --NGT-----
CS_G102.US               --------N- ---------- --------R- --M------G -----G---- ----D----- ----I--N-- -----E---- --------V- --NGT-----
OXF.B5S7.02.UK           --------N- ---------- --------R- --M------G -----G---- ----D----- ----I--N-- -----E---- --------V- --NGT-----
OXF.B5S8.02.UK           --------N- ---------- --------R- --M------G -----G---- ----D----- ----I--N-- -----E---- --------V- --NGT-----
OXF.B5S9.02.UK           --------N- ---------- --------R- --M------G -----G---- ----D----- ----I--N-- -----E---- --------V- --NGT-----
OXF.B5S10.02.UK          --------N- ---------- --------R- --M------G -----G---- ----D----- ----I--N-- -----E---- --------V- --NGT-----
GII.4.02a                --------N- ---------- --------R- --M------G -----G---- ----D----- ----I--N-- -----E---- --------V- --NGT-----
FHills.02.US             --------N- ---------- --------R- --M------G -----G---- ----D----- ----I--N-- -----E---- --------V- --NGT-----
Carlow.02.Ire            --------N- ---------- --------R- --M------G -----G---- ----D----- ----I--N-- -----E---- --------V- --NGT-----
OXF.B5S1.202.UK          --------N- ---------- --------R- --M------G -----G---- ----D----- ----I--N-- -----E---- --------V- --NGT-----
OXF.B4S5.02.UK           --------N- ---------- --------R- --M------G -----G---- ----D----- ----I--N-- -----E---- --------V- --NGT-----
OXF.B4S6.02.UK           --------N- ---------- --------R- --M------G -----G---- ----D----- ----I--N-- -----E---- --------V- --NGT-----
OXF.B4S4.02.UK           --------N- ---------- --------R- --M------G -----G---- ----D----- ----I--N-- -----E---- --------V- --NGT-----
OXF.B4S7.02.UK           --------N- ---------- --------R- --M------G -----G---- ----D----- ----I--N-- -----E---- --------V- --NGT-----
OXF.B4S2.02.UK           --------N- ---------- --------R- --M------G -----G---- ----D----- ----I--N-- -----E---- --------V- --NGT-----
OXF.B8S5.02.UK           --------N- ---------- --------R- --M------G -----G---- ----D----- ----I--N-- -----E---- --------V- --NGT-----
OXF.B4S1.02.UK           --------N- ---------- --------R- --M------G -----G---- ----D----- ----I--N-- -----E---- --------V- --NGT-----
Middelburg007.04.NL      --------N- ---------- --------R- --M------G -----G---- ----D----- ----I--N-- -----E---- --------V- --NGT-----
Anchorage.02.US          --------N- ---------- --------R- --M------G -----G---- ----D----- ----I--N-- -----E---- --------V- --NGT-----
MD_04.04.US              --------N- ---------- ---------- --M------G -----G---- ----D----- ----I--N-- -----E---- --------V- --NGT-----
Haarl457.03.NL           --------N- ---------- --------R- --M------G -----G---- ----D----- ----I--N-- -----E---- --------V- --NGT-----
Heerlen003.03.NL         --------N- ---------- --------R- --M------G -----G---- ----D----- ----I--N-- -----E---- --------V- --NGT-----
OXF.B5S13.02.UK          --------N- ---------- --------R- --M------G -----G---- ----D----- -------N-- -----E---- --------V- --NGA-----
OXF.B5S19.02.UK          --------N- ---------- --------R- --M------G -----G---- ----D----- -------N-- -----E---- --------V- --NGA-----
Elsloo012.04.NL          --------N- ---------- --------R- --M------G -----G---- ----D----- -------D-- --S--E---- --------V- --NGA-----
DenHelder003.04.NL       --------N- ---------- --------R- --M------G -----G---- ----D----- ----I--N-- -----E---- ---------- --NGA-----
GII.4.02                 --------N- ---------- --------R- --M------G -----G---- ----D----- ----I--N-- -----E---- --------V- --NGA-----
Apeldoorn023.03.NL       --------N- ---------- --------R- --M------G -----G---- ----D----- ----I--N-- -----E---- --------V- --NGA-----
GA04.04.US               --------N- ---------- --------R- --M------R -----G---- ----DA---- ----IM-S-- -D---E---- --------V- --NGT-----
Lanzhou.35666.02.CHN     --------N- ---------- ---------- --V------G -----G---- ---------- ---------- -----E---- --------V- --NST-----
Liempde048.04.NL         --------N- ---------- ---------- --V------R -----G---- ---------- -------S-- -S---E---- -R------V- --STT-----
G2.4.04.NL               --------N- ---------- ---------- --V------R -----G---- ---------- -------S-- -S---E---- -R------V- --STT-----
Almelo039.04.NL          --------N- ---------- ---------- --V------R -----G---- ---------- -------S-- -S---E---- -R------V- --STT-----
DenBosch028.04.NL        --------N- ---------- ---------- --V------R -----G---- ---------- -------S-- -S---E---- -R------V- --STT-----
Tilburg059.04.NL         --------N- ---------- ---------- --V------R -----G---- -----I---- -------S-- -S---E---- -R------V- --STT-----
Nijmegen083.04.NL        --------N- ---------- ---------- --V------R -----G---- -----I---- -------S-- -S---E---- -R------V- --STT-----
Dongen46.06.NL           --------N- ---------- ---------- --V------R -----G---- ---------- -------S-- -S---EA--- -R------V- --STT-----
Hunter284E.04O.AU        --------N- ---------- ---------- --V------R -----G---- ---------- -------S-- -S---E---- -R------V- --STT-----
GII.4.04                 --------N- ---------- ---------- --V------R -----G---- ---------- -------S-- -S---E---- -R------V- --STT-----
DenHaag54.06.NL          --------N- ------V--- ---------- --V------G -----G---- ---------- -------S-- -S---E---- -R------V- --STT-----
Yerseke38.06.NL          --------N- ---------- ---------- --V------R -----G---- ---------- ---RI--S-- -S---E---- -R------V- --STT-----
Terneuzen70.06.NL        --------N- ---------- ---------- --V------R -----G---- ---------- ---RI--S-- -S---E---- -R------V- --STT-----
Sakai.04_179.05.JP       --------N- ---------- ---------- --M-----KG -----G---- ------D--- -------A-- -D---E---- -R-------- --SSA-R---
Beijing.CR2932.04.CHN    --------N- ---------- ---------- --M-----KG -----G---- ------D--- --D----A-- -D---E---- -R-------- --SSA-R---
GII.4.05                 --------N- ---------- ---------- --M-----KG -----G---- ------D--- -------A-- -D---E---- -R-------- --SSA-R---
Chiba.04_1050.05.JP      --------N- ---------- ---------- --M-----KG -----G---- ------D--- -------A-- -D---E---- -R-------- --SSA-R---
GNZH.NVgz01.CHN          --------N- ---------- ---------- --M-----KG -----G---- ------D--- -------A-- -D---E---- -R-------- --SSA-R---
Beijing.CR2905.04.CHN    --------N- ---------- ---------- --M-----KG -----G---- ------D--- -------A-- -D---E---- -R-------- --SST-R---
GNZH.NV_VP1.06.CHN       --------N- ---------- ---------- --M-----KG -----G---- ------D--- -------A-- -D---E---- -R-------- --SSA-R---
Ehime.05_30.05.JP        --------N- ---------- ---------- --M-----KG -----G---- ------D--- -------A-- -D---E---- -R-------- --SST-R---
QM2_CS.04.US             --------N- ---------- ---------- --M-----KG -----G---- ------D--- -------A-- -D---E---- -R-------- --SSA-R---
DenHaag89.06.NL          -----L--N- ---------- ---------- --V-----KG -----G---- -Y---AP--- -------S-- -E---E-H-- ---------- --STT-R---
Kobe034.06.JP            -----L--N- ---------- ---------- --V-----KG -----G---- -Y---AP--- -------S-- -E---E-H-- ---------- --STT-R---
Nijmegen115.06.NL        -----L--N- ---------- ---------- --V-----KG -----G---- -Y---AP--- -------S-- -E---E-H-- ---------- --STT-R--- 400
```

Camberwell.94.AU	QQWVLPNYSG	RTGHNVHLAP	AVAPTFPGEQ	LLFFRSTMPG	CSGYPNMNLD	CLLPQEWVLH	FYQEAAPAQS	DVALLRFVNP	DTGRVLFECK	LHKSGYITVA
MD145_12.87.US	----------	----------	----------	----------	----------	----------	----------	----------	----------	----------
GII.4.87	----------	----------	----------	----------	----------	----------	----------	----------	----------	----------
MD134_7.87.US	------D---	----------	----------	----------	----------	----------	----------	----------	----------	----------
Lord.93.UK	---S------	----------	----------	----------	----------	----------	----------	----------	----------	----------
UK3_17.12700.92.GB	---L------	----------	----------	----------	----------	----------	----------	----------	----------	----------
EmmenE006.02.NL	------DF--	----------	----------	----------	R---------	----------	----S-----	----------	--N-------	----------
Richmont.94.US	----------	--V-------	----------	----------	----------	--------M-	----------	----------	----------	---A------
345.96002726.96.SC.US	----------	----------	----------	----------	----------	--------Q-	----------	----------	----------	------V---
Hamburg135.98.GE	----------	----------	----------	----------	----------	--------Q-	----------	----------	----------	------V---
004.95M_14.95.AU	----------	----------	----------	----------	----------	--------Q-	----------	----------	----------	------V---
373.96019743.96.SC.US	----------	----------	----------	----------	----------	--------Q-	----------	----------	----------	------V---
OBSM.112.99.DE	----------	----------	----------	----------	----------	--------Q-	----------	----------	----------	------V---
384.96025046.96.FL.US	----------	----------	----------	----------	----------	--------Q-	----------	----------	----------	------V---
416.97003156.96.LA.US	----------	----------	----------	----------	----------	--------Q-	----------	----------	----------	------V---
Mora.97.SE	----------	----------	----------	----------	----------	--------Q-	----------	----------	----------	------V---
Hamburg180.97.GE	----------	----------	----------	----------	----------	--------Q-	----------	----------	----------	------V---
DenHaag015.00.NL	----------	----------	----------	----------	----------	--------Q-	----------	----------	----------	------V---
Dresden153.97.GE	----------	----------	----------	----------	----------	--------Q-	----------	----------	----------	------V---
358.96015107.96.FL.US	----------	----------	----------	----------	----------	--------Q-	----------	----------	----------	------V---
WDVN016.00.NL	----------	----------	----------	----------	----------	--------Q-	----------	----------	----------	------V---
Beeskow.124.00.DE	----------	----------	----------	----------	----------	--------Q-	----------	----------	----------	------V---
BWLND.331.95.US	----------	----------	----------	----K-----	----------	--------Q-	----------	----------	----------	------V---
Berlin.159.98.DE	----------	----------	----------	----------	----------	--------Q-	----------	----------	----------	------V---
Osaka.98.JPN	----------	----------	----------	----------	----------	--------Q-	----------	----------	----------	------V---
Berlin.238.98.DE	----------	----------	----------	----------	----------	--------Q-	----------	----------	----------	------V---
Hamburg189.97.GE	----------	----------	----------	----------	----------	--------Q-	----------	----------	----------	------V---
FRBG057.97.GE	----------	----------	----------	----------	----------	--------Q-	----------	----------	----------	------V---
Grimsby.98.UK	----------	----------	----------	----------	----------	--------Q-	----------	----------	----------	-Y----V---
345_2.96002737.96.SC.US	----------	----------	----------	----------	----------	--------Q-	----------	----------	----------	------V---
364.96019537.96.AZ.US	----------	----------	----------	----------	----------	--------Q-	----------	----------	----------	------V---
379.960984.96.AZ.US	----------	----------	----------	----------	----------	--------Q-	----------	----------	----------	------V---
Dresden245.97.GE	----------	----------	----------	----------	----------	--------Q-	----------	----------	----------	------V---
DOUG4770.01.AU	-----T----	----------	----------	----------	----------	--------Q-	----------	----------	----------	------V---
ERL195.97.GE	H---------	----------	----------	----------	----------	--------Q-	----------	----------	----------	------V---
FRBG253.98.GE	H---------	----------	----------	----------	----------	--------Q-	----------	----------	----------	------V---
Bochum024.98.GE	----------	----------	----------	----------	----------	--------Q-	----------	----------	----------	------V---
Bochum026.97.GE	----------	G---------	----------	----------	----------	-YS-----Q-	----------	----------	----------	------V---
Narita104.02.JPN	----------	----------	----------	----------	----------	--------Q-	-C--------	----------	----------	------V---
GII.4.97	----------	----------	----------	----------	----------	--------Q-	----------	----------	----------	------V---
Sydney348.97O.AU	----------	----------	----------	----------	----------	--------Q-	---------P	----------	----------	------V---
DIJON171.96.FRA	----------	----------	----------	----------	----------	--------Q-	----------	----------	----------	------V---
FRBG024.97.GE	----------	----------	----------	----------	----------	--------Q-	----------	----------	----------	------V---
Hamburg236.97.GE	----------	-A--------	----------	----------	----------	--------Q-	----------	----------	----------	------V---
Dresden319.97.GE	----------	-P--------	------L---	----------	----------	--------Q-	----------	----------	----------	------V---
Hamburg316.98.GE	----------	----------	----------	----------	----------	--------Q-	----------	----------	----------	------V---
LDW.221.99.DE	----------	-A--------	----------	----------	----------	--------Q-	----------	----------	----------	------V---
LDW.218.99.DE	----------	-A--------	----------	----------	----------	--------Q-	----------	----------	----------	------V---
Berlin.495.00.DE	----------	----------	----------	----------	----------	--------Q-	----------	----------	----------	------V---
MIBE.326.95.US	----------	----------	----------	----------	----------	--------Q-	----------	----------	----------	------V---
366.96019554.96.ID.US	----------	----------	----------	----------	----------	--------Q-	----------	----------	----------	------V---
Utrecht058.01.NL	----------	----------	----------	----------	----------	--------Q-	----------	----------	----------	------V---
Altenkirchen140.01.DE	----------	----------	----------	----------	----------	--------Q-	----------	----------	----------	------V---
Schiedam018.01.NL	----------	----------	----------	----------	----------	--------Q-	----------	----------	----------	------V---
Frankfurt.Oder.170.99.DE	----------	----------	----------	----------	----------	--------Q-	----------	----------	----------	------V---
Bochum272.98.GE	H---------	----------	----------	----------	----------	--------Q-	----------	----------	----------	------V---
Berlin.491.00.DE	----------	----------	----------	----------	----------	--------Q-	----------	----------	----------	------V---
Dresden267.97.GE	----------	----------	----------	----------	----------	--------Q-	----------	----------	----------	------V---
KNWH.130.00.DE	----------	----------	----------	----------	----------	--------Q-	----------	---------S	----------	------V---
Dillingen259.01.DE	----------	----------	----------	----------	----------	--------Q-	----------	----------	----------	------V---
Bochum136.98.GE	----------	----------	----------	----------	----------	--------Q-	----------	----------	----------	------V---
Bochum224.98.GE	----------	----------	----------	----------	----------	--------Q-	----------	----------	----------	------V---
Dresden174.pUS_NorII.97.GE	----------	----------	----------	----------	----------	--------Q-	----------	----------	----------	------V---
Bochum220.97.GE	----------	----------	----------	----------	----------	--------Q-	----------	----------	----------	------V---
Bochum108.97.GE	----------	----------	----------	----------	----------	--------Q-	----------	----------	----------	------V---
Bochum031.97.GE	----------	----------	----------	----------	----------	--------Q-	----------	----------	----------	------V---
VA98387.98.US	----------	----------	----------	----------	----------	--------Q-	----------	----------	----------	------V---
Bochum339.97.GE	----------	----------	----------	----------	----------	--------Q-	----------	----------	----------	------V---
Tiel001.95.NL	----------	----------	----------	----------	----------	--------Q-	----------	----------	----------	------V---
Parkroyal.95.UK	----------	----------	----------	----------	----------	--------Q-	----------	----------	----------	------V---
Symgreen.95.UK	----------	----------	----------	----------	----------	--------Q-	----------	----------	----------	------V---
YURI.32073.02.JP	----------	----------	----------	----------	----------	--------Q-	----------	----------	----------	------V---
Hamburg139.97.GE	------S---	----------	----------	----------	----------	--------Q-	----------	----------	----------	------V---
Hamburg137.97.GE	------S---	----------	----------	----------	----------	--------Q-	----------	----------	----------	------V---
Hamburg048.97.GE	------S---	----------	----------	----------	----------	--------Q-	----------	----------	----------	------V---
LWRDN043.00.NL	----------	----------	----------	----------	-------D--	--------Q-	----------	----------	----------	------V---
GCanyon.02.US	----------	----------	----------	----------	----------	--------Q-	----------	----------	----------	------V---
408.97003012.96.FL.US	----------	----------	----------	----------	----------	--------Q-	----------	----------	----------	------V---
HoUSton.95.US	----------	----------	----------	----------	----------	--------Q-	----------	----------	----------	------V---
Erfurt.007.00.DE	--------L-	-P--------	----------	----------	----------	--------Q-	----------	----------	----------	------V---
OXF.B1S16.02.UK	------S---	----------	----------	----------	----------	--------Q-	----------	----------	----------	------V---
OXF.B1S21.02.UK	------S---	----------	----------	----------	----------	--------Q-	----------	----------	----------	------V---
OXF.B1S12.02.UK	------S---	----------	----------	----------	----------	--------Q-	----------	----------	----------	------V---
OXF.B1S11.02.UK	------S---	----------	----------	----------	----------	--------Q-	----------	----------	----------	------V---
OXF.B1S4.02.UK	------S---	----------	----------	----------	----------	--------Q-	----------	----------	----------	------V---
OXF.B1S2.02.UK	------S---	----------	----------	----------	----------	--------Q-	----------	----------	----------	------V---
OXF.B1S1.02.UK	------S---	----------	----------	----------	----------	--------Q-	----------	----------	----------	------V---
Warren.02.US	------S---	----------	----------	----------	----------	--------Q-	----------	----------	----------	------V---
OXF.B5S22.03.UK	------S---	----------	----------	----------	----------	--------Q-	----------	----------	----------	------V---
OXF.B2S10.02.UK	------S---	----------	----------	----------	----------	--------Q-	----------	----------	----------	------V---
OXF.B2S11.02.UK	------S---	----------	----------	----------	----------	--------Q-	----------	----------	----------	------V---
OXF.B2S14.02.UK	------S---	----------	----------	----------	----------	--------Q-	----------	----------	----------	------V---
OXF.B2S15.02.UK	------S---	----------	----------	----------	----------	--------Q-	----------	----------	----------	------V---
OXF.B2S17.02.UK	------S---	----------	----------	----------	----------	--------Q-	----------	----------	----------	------V---
OXF.B2S18.02.UK	------S---	----------	----------	----------	----------	--------Q-	----------	----------	----------	------V---
OXF.B2S20.02.UK	------S---	----------	----------	----------	----------	--------Q-	----------	----------	----------	------V---
OXF.B2S23.02.UK	------S---	----------	----------	----------	----------	--------Q-	----------	----------	----------	------V---
OXF.B2S24.02.UK	------S---	----------	----------	----------	----------	--------Q-	----------	----------	----------	------V---
OXF.B2S25.02.UK	------S---	----------	----------	----------	----------	--------Q-	----------	----------	----------	------V---
OXF.B2S26.02.UK	------S---	----------	----------	----------	----------	--------Q-	----------	----------	----------	------V---
OXF.B3S4.03.UK	------S---	----------	----------	----------	----------	--------Q-	----------	----------	----------	------V---
OXF.B3S16.03.UK	------S---	----------	----------	----------	----------	--------Q-	----------	----------	----------	------V---

```
OXF.B3S18.03.UK           ------S--- ---------- ---------- ---------- ---------- --------Q- ---------- ---------- ---------- ------V---
OXF.B3S19.03.UK           ------S--- ---------- ---------- ---------- ---------- --------Q- ---------- ---------- ---------- ------V---
OXF.B3S20.03.UK           ------S--- ---------- ---------- ---------- ---------- --------Q- ---------- ---------- ---------- ------V---
OXF.B6S3.03.UK            ------S--- ---------- ---------- ---------- ---------- --------Q- ---------- ---------- ---------- ------V---
OXF.B6S5.03.UK            ------S--- ---------- ---------- ---------- ---------- --------Q- ---------- ---------- ---------- ------V---
OXF.B5S23.03.UK           ------S--- ---------- ---------- ---------- ---------- --------Q- ---------- ---------- ---------- ------V---
OXF.B6S2.03.UK            ------S--- ---------- ---------- ---------- ---------- --------Q- ---------- ---------- ---------- ------V---
Witney.B7S2.03.UK         ------S--- ---------- ---------- ---------- ---------- --------Q- ---------- ---------- ---------- ------V---
OXF.B2S16.02.UK           ------S--- ---------- ---------- ---------- ---------- --------Q- ---------- ---------- ---------- ------V---
Banbury.B9S23.03.UK       ------S--- ---------- ---------- ---------- ---------- --------Q- ---------- ---------- ---------- ------V---
CHPNRT.03.UK              ------S--- ---------- ---------- ---------- ---------- --------Q- ---------- ---------- ---------- ------V---
Didcot.B9S2.03.UK         ------S--- ---------- ---------- ---------- ---------- --------Q- ---------- ---------- ---------- ------V---
OXF.B6S4.03.UK            ------S--- ---------- ---------- ---------- ---------- --------Q- ---------- ---------- ---------- ------V---
OXF.B6S6.03.UK            ------S--- ---------- ---------- -------T-- ---------- --------Q- ---------- ---------- ---------- ------V---
DenHaag001.03.NL          ------S--- ---------- ---------- ---------- ---------- --------Q- ---------- ---------- ---------- ------V---
WeertE022.02.NL           ------S--- ---------- ---------- ---------- ---------- --------Q- ---------- ---------- ---------- ------V---
CS_G2.02.US               ------S--- ---------- ---------- ---------- ---------- --------Q- ---------- ---------- ---------- ------V---
Langen1061.02.DE          ------S--- ---------- ---------- ---------- ---------- --------Q- ---------- ---------- ---------- ------V---
CS_D1.02.CAN              ------S--- ---------- ---------- ---------- ---------- --------Q- ---------- ---------- ---------- ------V---
CS_G4.02.US               ------S--- ---------- ---------- ---------- ---------- --------Q- ---------- ---------- ---------- ------V---
Germanton.02.US           ------S--- ---------- ---------- ---------- ---------- --------Q- ---------- ---------- ---------- ------V---
CS_G102.US                ------S--- ---------- ---------- ---------- ---------- --------Q- ---------- ---------- ---------- ------V---
OXF.B5S7.02.UK            ------S--- ---------- ---------- ------A--- ---------- --------Q- ---------- ---------- ---------- ------V---
OXF.B5S8.02.UK            ------S--- ---------- ---------- ------A--- ---------- --------Q- ---------- ---------- ---------- ------V---
OXF.B5S9.02.UK            ------S--- ---------- ---------- ------A--- ---------- --------Q- ---------- ---------- ---------- ------V---
OXF.B5S10.02.UK           ------S--- ---------- ---------- ------A--- ---------- --------Q- ---------- ---------- ---------- ------V---
GII.4.02a                 ------S--- ---------- ---------- ---------- ---------- --------Q- ---------- ---------- ---------- ------V---
FHills.02.US              ------S--- ---------- ---------- ---------- ---------- --------Q- ---------- ---------- ---------- ------V---
Carlow.02.Ire             ------S--- ---------- ---------- ---------- ---------- --------Q- ---------- ---------- ---------- ------V---
OXF.B5S1.202.UK           ------S--- ---------- ---------- ---------- ---------- --------Q- ---------- ---------- ---------- ------V---
OXF.B4S5.02.UK            ------S--- ---------- ---------- ---------- ---------- --------Q- ---------- ---------- ---------- ------V---
OXF.B4S6.02.UK            ------S--- ---------- ---------- ---------- ---------- --------Q- ---------- ---------- ---------- ------V---
OXF.B4S4.02.UK            ------S--- ---------- ---------- ---------- ---------- --------Q- ---------- ---------- ---------- ------V---
OXF.B4S7.02.UK            ------S--- ---------- ---------- ---------- ---------- --------Q- ---------- ---------- ---------- ------V---
OXF.B4S2.02.UK            ------S--- ---------- ---------- ---------- ---------- --------Q- ---------- ---------- ---------- ------V---
OXF.B8S5.02.UK            ------S--- ---------- ---------- ---------- ---------- --------Q- ---------- ---------- ---------- ------V---
OXF.B4S1.02.UK            ------S--- ---------- ---------- ---------- ---------- --------Q- ---------- ---------- ---------- ---T--V---
Middelburg007.04.NL       ------S--- ---------- ---------- ---------- ---------- --------Q- ---------- ---------- ---------- ------V---
Anchorage.02.US           ------S--- ---------- ---------- ---------- ---------- --------Q- ---------- ---------- ---------- ------V---
MD_04.04.US               ------S--- ---------- ---------- ---------- ---------- --------Q- ---------- ---------- ---------- ------V---
Haarl457.03.NL            ------S--- ---------- ---------- ---------- ---------- --------Q- ---------- ---------- ---------- ------L---
Heerlen003.03.NL          ------S--- --S------- ---------- ---------- ---------- --------Q- ---------- ---------- ---------- ------V---
OXF.B5S13.02.UK           ------S--- ---------- ---------- ---------- ---------- --------Q- ---------- ---------- ---------- ------V---
OXF.B5S19.02.UK           ------S--- ---------- ---------- ---------- ---------- --------Q- ---------- ---------- ---------- ------V---
Elsloo012.04.NL           ------S--- -----A---- ---------- ---------- ---------- --------Q- ---------- ---------- ---------- ------V---
DenHelder003.04.NL        ------S--- ---------- ---------- ---------- ---------- --------Q- ---------- ---------- ---------- ----------
GII.4.02                  ------S--- ---------- ---------- ---------- ---------- --------Q- ---------- ---------- ---------- ------V---
Apeldoorn023.03.NL        ------S--- ---------- ---------- ---------- ---------- --------Q- ---------- ---------- ---------- ------V---
GA04.04.US                ------S--- ---------- ---------- ---------- ---------- --------Q- ---------- ---------- ---------- ------V---
Lanzhou.35666.02.CHN      ---------- ---------- ---------- ---------- ---------- --------Q- ---------- ---------- ---------- ------V---
Liempde048.04.NL          ------D--- -DS------- ---------- ---------- ---------- --------Q- ----S----- ---------- ---------- ------V---
G2.4.04.NL                ------D--- -DS------- ---------- ---------- ---------- --------Q- ----S----- ---------- ---------- ------V---
Almelo039.04.NL           ------D--- -DS------- ---------- ---------- ---------- --------Q- ----S----- ---------- ---------- ------V---
DenBosch028.04.NL         ------D--- -DS------- ---------- ---------- ---------- --------Q- ----S----- ---------- ---------- ------V---
Tilburg059.04.NL          ------D--- -DS------- ---------- ---------- ---------- --------Q- ----S----- ---------- ---------- ------V---
Nijmegen083.04.NL         ------D--- -DS------- ---------- ---------- ---------- --------Q- ----S----- ---------- ---------- ------V---
Dongen46.06.NL            ------D--- -DS------- ---------- ---------- ---------- --------Q- ----S----- ---------- ---------- ------V---
Hunter284E.04O.AU         ------D--- -DS------- ---------- ---------- ---------- --------Q- ----S----- ---------- ---------- ------V---
GII.4.04                  ------D--- -DS------- ---------- ---------- ---------- --------Q- ----S----- ---------- ---------- ------V---
DenHaag54.06.NL           ------D--- -DN------- ---------- ---------- ---------- --------Q- ----S----- ---------- ---------- ------V---
Yerseke38.06.NL           ------D--- -DS------- ----S----- ---------- ---------- --------Q- ---------- ---------- ---------- ------V---
Terneuzen70.06.NL         ---------- -DS------- ----S----- ---------- ---------- --------Q- ---------- ---------- ---------- ------V---
Sakai.04_179.05.JP        ------D--- --V------- ---------- ---------- -------D-- --------Q- -------S-- ---------- ---------- ---A--V---
Beijing.CR2932.04.CHN     ------D--- --V------- ---------- ---------- -------D-- --------Q- -------S-- ---------- ---------- ---A--V---
GII.4.05                  ------D--- --V------- ---------- ---------- -------D-- --------Q- -------S-- ---------- ---------- ---A--V---
Chiba.04_1050.05.JP       ------D--- --V------- ---------- ---------- -------D-- --------Q- -------S-- ---------- ---------- ---A--V---
GNZH.NVgz01.CHN           ------D--- --V------- ---------- ---------- -------D-- --------Q- -------S-- ---------- ---------- ---A--V---
Beijing.CR2905.04.CHN     ------D--- --V------- ---------- ---------- -------D-- --------Q- -------S-- ---------- ---------- ---A--V---
GNZH.NV_VP1.06.CHN        ------D--- --V------- ---------- -P-------- -------D-- --------Q- -------S-- ---------- ---------- ---A--V---
Ehime.05_30.05.JP         ------D--- --V------- ---------- ---------- -------D-- --------Q- -------S-- ---------- ---------- ---A--V---
QM2_CS.04.US              ------D--- --V------- ---------- ---------- -------D-- --------Q- ---------- ---------- ---------- ---T--V---
DenHaag89.06.NL           ------S--- -NV------- ---------- ---------- -------D-- --------Q- ---------- ---------- ---------- ------V---
Kobe034.06.JP             ------S--- -NV------- ---------- ---------- -------D-- --------Q- ---------- ---------- ---------- ------V---
Nijmegen115.06.NL         ------S--- -NV------- ---------- ---------- -------D-- --------Q- ---------- ---------- ---------- ------V--- 500


Camberwell.94.AU          HTGPFDLVIPPNGYFRFDSWVNQFYTLAPMGNGTGRRRAL
MD145_12.87.US            ----Y-----------------------------------
GII.4.87                  ----Y-----------------------------------
MD134_7.87.US             ----Y-----------------------------------
Lord.93.UK                ----Y---L-------------------------------
UK3_17.12700.92.GB        ----Y---L-------------------------------
EmmenE006.02.NL           ---HH--IL------------------------A-----.
Richmont.94.US            ---QH----------------------------A------
345.96002726.96.SC.US     ----H----------------------------A------
Hamburg135.98.GE          ----H----------------------------A------
004.95M_14.95.AU          ----H----------------------------A------
373.96019743.96.SC.US     ----H----------------------------A------
OBSM.112.99.DE            ----H----------------------------A------
384.96025046.96.FL.US     ----H----------------------------A------
416.97003156.96.LA.US     ----H----------------------------A------
Mora.97.SE                ----H----------------------------A------
Hamburg180.97.GE          ----H----------------------------A------
DenHaag015.00.NL          ----H----------------------------A------
Dresden153.97.GE          ----H----------------------------A------
358.96015107.96.FL.US     ----H------S---------------------A------
WDVN016.00.NL             ----H----------------------------A------
Beeskow.124.00.DE         ----H----------------------------A------
BWLND.331.95.US           ----H----------------------------A------
Berlin.159.98.DE          ----H----------------------------A------
Osaka.98.JPN              ----H----------------------------A------
```

```
Berlin.238.98.DE            ----H----------------------------A------
Hamburg189.97.GE            ----H----------------------------A------
FRBG057.97.GE               ----H------------------V---------A------
Grimsby.98.UK               ----H------------------X---------A------
345_2.96002737.96.SC.US     ----H----------------------------A------
364.96019537.96.AZ.US       ----H----------------------------A------
379.960984.96.AZ.US         ----H----------------------------A------
Dresden245.97.GE            ----H----------------------------A------
DOUG4770.01.AU              ----H-------------P--------------A------
ERL195.97.GE                ----H----------------------------A------
FRBG253.98.GE               ----H----------------------------A------
Bochum024.98.GE             ----H----------------------------A------
Bochum026.97.GE             ----H----------------------------A------
Narita104.02.JPN            ----H----------------------------A------
GII.4.97                    ----H----------------------------A------
Sydney348.97O.AU            ----H----------------------------A------
DIJON171.96.FRA             ----H----------------------------A------
FRBG024.97.GE               ----H----------------------------A------
Hamburg236.97.GE            ----H--------------------A-------A------
Dresden319.97.GE            ----H----------------------------A------
Hamburg316.98.GE            ----H----------------------------A------
LDW.221.99.DE               ----H----------------------------A------
LDW.218.99.DE               ----H----------------------------A------
Berlin.495.00.DE            ----H----------------------------A------
MIBE.326.95.US              ----H---T------------------------A------
366.96019554.96.ID.US       ----H----------------------------A------
Utrecht058.01.NL            ----H----------------------------A------
Altenkirchen140.01.DE       ----H----------------------------A------
Schiedam018.01.NL           ----H----------------------------A--SS--
Frankfurt.Oder.170.99.DE    ----H----------------------------A------
Bochum272.98.GE             ----H----------------------------A------
Berlin.491.00.DE            ----H----------------------------V------
Dresden267.97.GE            ----Y----------------------------A------
KNWH.130.00.DE              ----H----------------------------A------
Dillingen259.01.DE          ----H----------------------------A------
Bochum136.98.GE             ----H----------------------------A------
Bochum224.98.GE             ----H----------------------------A------
Dresden174.pUS_NorII.97.GE  ----H----------------------------A------
Bochum220.97.GE             ----H----------------------------A------
Bochum108.97.GE             ----H----------------------------A------
Bochum031.97.GE             ----H---------------I------------A------
VA98387.98.US               ----H----------------------------A------
Bochum339.97.GE             ----H----------------------------A------
Tiel001.95.NL               ----H----------------------------A------
Parkroyal.95.UK             ----H----------------------------A------
Symgreen.95.UK              ----H------------------L---------A------
YURI.32073.02.JP            ----H----------------------------A------
Hamburg139.97.GE            ----H----------------------------A------
Hamburg137.97.GE            ----H-------------------------R--A------
Hamburg048.97.GE            ----H----------------------------A------
LWRDN043.00.NL              ----H---------------I------------A------
GCanyon.02.US               ----H-----------------------------------
408.97003012.96.FL.US       ----H----------------------------A------
HoUSton.95.US               ----H-----------------------------------
Erfurt.007.00.DE            Y---H----------------------------------V
OXF.B1S16.02.UK             ---QH-----------------------------------
OXF.B1S21.02.UK             ---QH-----------------------------------
OXF.B1S12.02.UK             ---QH-----------------------------------
OXF.B1S11.02.UK             ---QH-----------------------------------
OXF.B1S4.02.UK              ---QH-----------------------------------
OXF.B1S2.02.UK              ---QH-----------------------------------
OXF.B1S1.02.UK              ---QH-----------------------------------
Warren.02.US                ---QH-----------------------------------
OXF.B5S22.03.UK             ---QH-----------------------------------
OXF.B2S10.02.UK             ---QH-----------------------------------
OXF.B2S11.02.UK             ---QH-----------------------------------
OXF.B2S14.02.UK             ---QH-----------------------------------
OXF.B2S15.02.UK             ---QH-----------------------------------
OXF.B2S17.02.UK             ---QH-----------------------------------
OXF.B2S18.02.UK             ---QH-----------------------------------
OXF.B2S20.02.UK             ---QH-----------------------------------
OXF.B2S23.02.UK             ---QH-----------------------------------
OXF.B2S24.02.UK             ---QH-----------------------------------
OXF.B2S25.02.UK             ---QH-----------------------------------
OXF.B2S26.02.UK             ---QH-----------------------------------
OXF.B3S4.03.UK              ---QH-----------------------------------
OXF.B3S16.03.UK             ---QH-----------------------------------
OXF.B3S18.03.UK             ---QH-----------------------------------
OXF.B3S19.03.UK             ---QH-----------------------------------
OXF.B3S20.03.UK             ---QH-----------------------------------
OXF.B6S3.03.UK              ---QH-----------------------------------
OXF.B6S5.03.UK              ---QH-----------------------------------
OXF.B5S23.03.UK             ---QH-----------------------------------
OXF.B6S2.03.UK              ---QH-----------------------------------
Witney.B7S2.03.UK           ---QH-----------------------------------
OXF.B2S16.02.UK             ---QH-----------------------------------
Banbury.B9S23.03.UK         ---QH-----------------------------------
CHPNRT.03.UK                ---QH-----------------------------------
Didcot.B9S2.03.UK           ---QH-----------------------------------
OXF.B6S4.03.UK              ---QH-----------------------------------
OXF.B6S6.03.UK              ---QH-----------------------------------
DenHaag001.03.NL            ---QH-----------------------------------
WeertE022.02.NL             ---QH-----------------------------------
CS_G2.02.US                 ---QH-----------------------------------
Langen1061.02.DE            ---QH-----------------------------------
CS_D1.02.CAN                ---QH-----------------------------------
CS_G4.02.US                 ---QH-----------------------------------
Germanton.02.US             ---QH-----------------------------------
CS_G102.US                  ---QH-----------------------------------
OXF.B5S7.02.UK              ---QH-----------------------------------
OXF.B5S8.02.UK              ---QH-----------------------------------
OXF.B5S9.02.UK              ---QH-----------------------------------
```

```
OXF.B5S10.02.UK             ---QH-----------------------------------
GII.4.02a                   ---QH-----------------------------------
FHills.02.US                ---QH-----------------------------------
Carlow.02.Ire               ---QH-----------------------------------
OXF.B5S1.202.UK             ---QH-----------------------------------
OXF.B4S5.02.UK              ---QH-----------------------------------
OXF.B4S6.02.UK              ---QH-----------------------------------
OXF.B4S4.02.UK              ---QH-----------------------------------
OXF.B4S7.02.UK              ---QH-----------------------------------
OXF.B4S2.02.UK              ---QH-----------------------------------
OXF.B8S5.02.UK              ---QH-----------------------------------
OXF.B4S1.02.UK              ---QH-----------------------------------
Middelburg007.04.NL         ---QH-----------------------------------
Anchorage.02.US             ---QH-----------------------------------
MD_04.04.US                 ---QH-----------------------------------
Haarl457.03.NL              ---QH-----------------------------------
Heerlen003.03.NL            ---QH-----------------------------------
OXF.B5S13.02.UK             ---QH-----------------------------------
OXF.B5S19.02.UK             ---QH-----------------------------------
Elsloo012.04.NL             ---QH-----------------------------------
DenHelder003.04.NL          ---QH-----------------------------------
GII.4.02                    ---QH-----------------------------------
Apeldoorn023.03.NL          ---QH-----------------------------------
GA04.04.US                  ---QH-----------------------------------
Lanzhou.35666.02.CHN        ---QH----------------------------A------
Liempde048.04.NL            ---QH----------------------------A------
G2.4.04.NL                  ---QH----------------------------A------
Almelo039.04.NL             ---QH----------------------------A------
DenBosch028.04.NL           ---QH----------------------------A------
Tilburg059.04.NL            ---QH----------------------------A------
Nijmegen083.04.NL           ---QH----------------------------A------
Dongen46.06.NL              Y--QH----------------------------A------
Hunter284E.04O.AU           ---QH---------------I------------A------
GII.4.04                    ---QH---------------I------------A------
DenHaag54.06.NL             ---QH----------------------------A------
Yerseke38.06.NL             ---QH-----------------------------------
Terneuzen70.06.NL           ---QH-----------------------------------
Sakai.04_179.05.JP          ---QH----------------------------A------
Beijing.CR2932.04.CHN       ---QH----------------------------A------
GII.4.05                    ---QH----------------------------A------
Chiba.04_1050.05.JP         ---QH----------------------------A------
GNZH.NVgz01.CHN             ---QH----------------------------A------
Beijing.CR2905.04.CHN       ---QH----------------------------A------
GNZH.NV_VP1.06.CHN          ---QH------------------S---------A------
Ehime.05_30.05.JP           ---QH----------------------------A------
QM2_CS.04.US                ---QH----------------------------A------
DenHaag89.06.NL             ---QH-----------------------------------
Kobe034.06.JP               ---QH-----------------------------------
Nijmegen115.06.NL           ---QH----------------------------------- 540
```
