# Supplementary material for: Mechanisms of GII.4 Norovirus Persistence in Human Populations
Source: PLoS Med. 2008 Feb 12;5(2):e31. doi: 10.1371/journal.pmed.0050031 (PMC2235898; doi:10.1371/journal.pmed.0050031)
Supplement: Supplementary file 2 [file pmed.0050031.sg002.pdf]

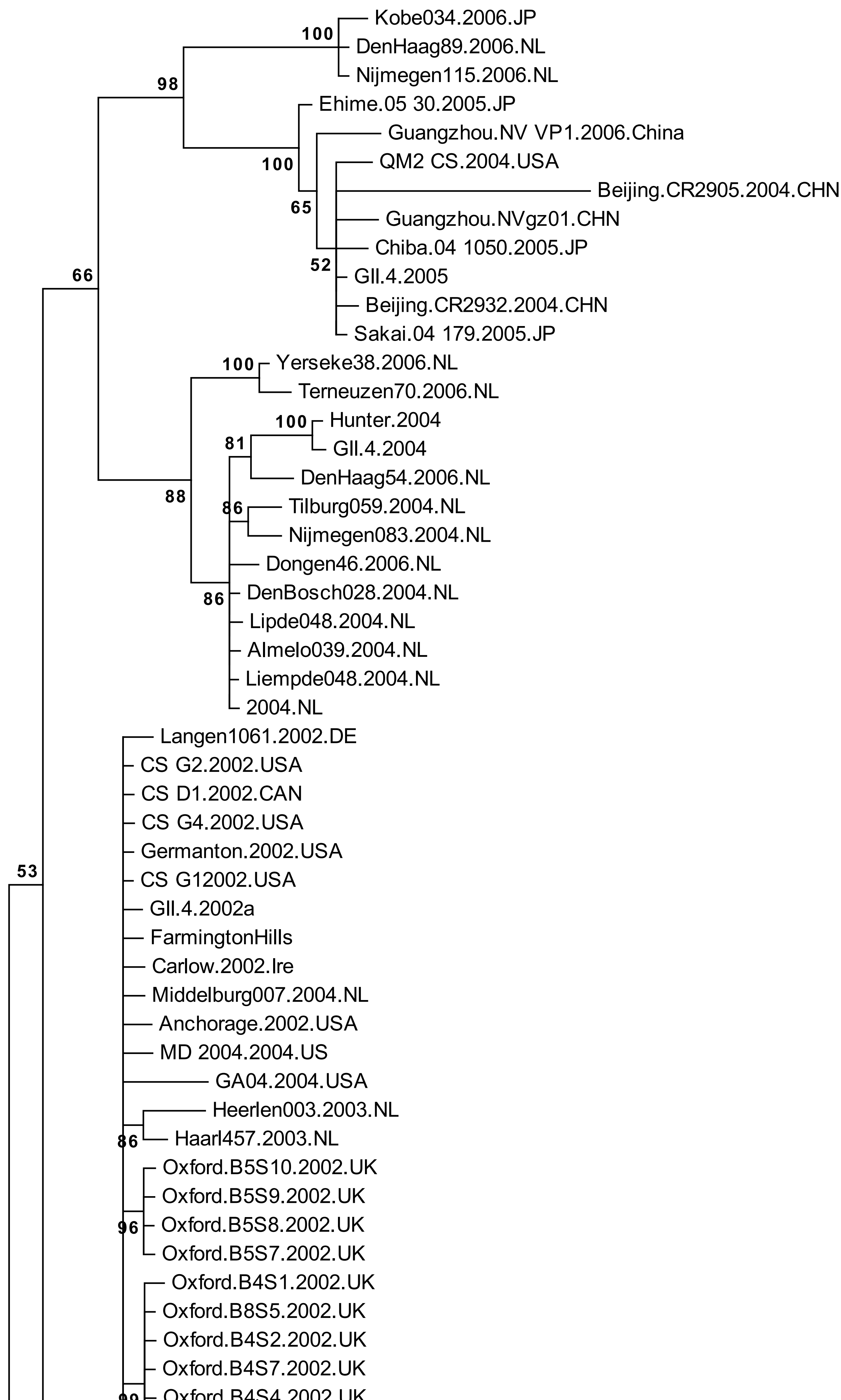

Kobe034.2006.JP
100
DenHaag89.2006.NL
Nijmegen115.2006.NL
98
Ehime.05 30.2005.JP
Guangzhou.NV VP1.2006.China
QM2 CS.2004.USA
100
Beijing.CR2905.2004.CHN
65
Guangzhou.NVgz01.CHN
Chiba.04 1050.2005.JP
52
GII.4.2005
66
Beijing.CR2932.2004.CHN
Sakai.04 179.2005.JP
100
Yerseke38.2006.NL
Terneuzen70.2006.NL
100
Hunter.2004
81
GII.4.2004
DenHaag54.2006.NL
88
86
Tilburg059.2004.NL
Nijmegen083.2004.NL
Dongen46.2006.NL
86
DenBosch028.2004.NL
Lipde048.2004.NL
Almelo039.2004.NL
Liempde048.2004.NL
2004.NL
Langen1061.2002.DE
CS G2.2002.USA
CS D1.2002.CAN
CS G4.2002.USA
Germanton.2002.USA
53
CS G12002.USA
GII.4.2002a
FarmingtonHills
Carlow.2002.Ire
Middelburg007.2004.NL
Anchorage.2002.USA
MD 2004.2004.US
GA04.2004.USA
Heerlen003.2003.NL
86
Haarl457.2003.NL
Oxford.B5S10.2002.UK
Oxford.B5S9.2002.UK
96
Oxford.B5S8.2002.UK
Oxford.B5S7.2002.UK
Oxford.B4S1.2002.UK
Oxford.B8S5.2002.UK
Oxford.B4S2.2002.UK
Oxford.B4S7.2002.UK

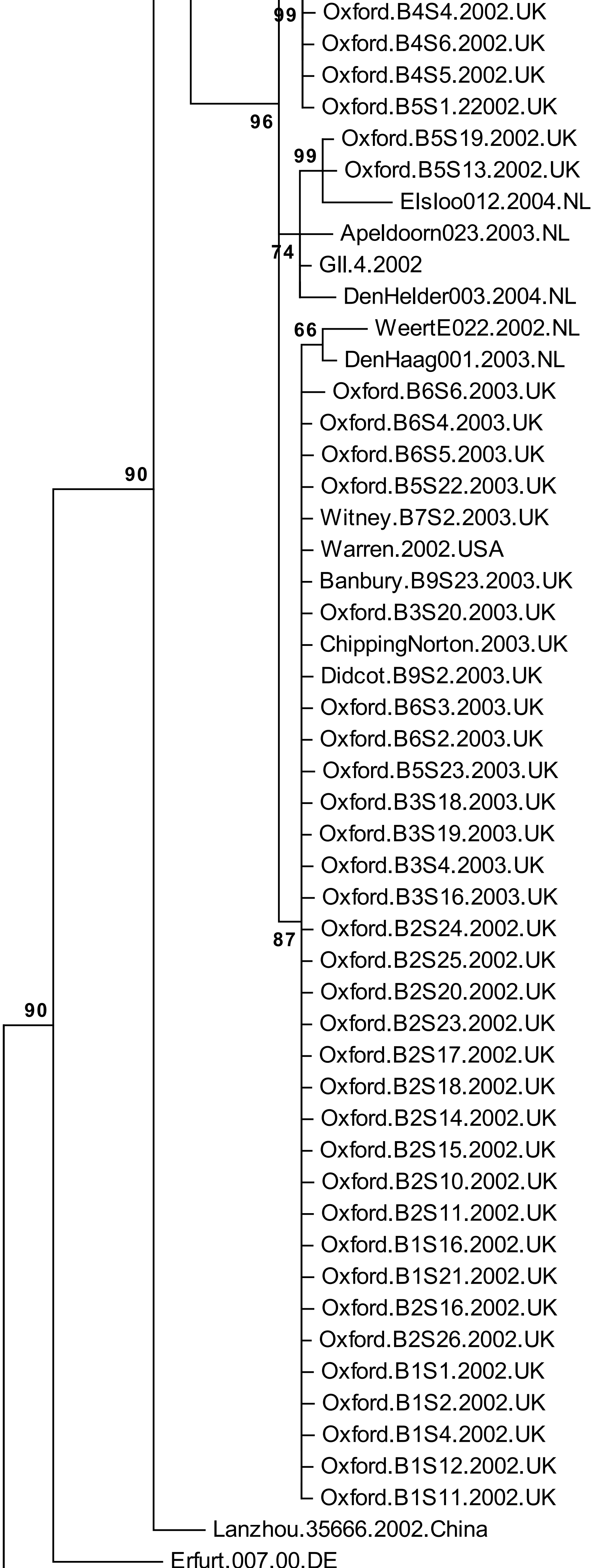

99
Oxford.B4S4.2002.UK
Oxford.B4S6.2002.UK
Oxford.B4S5.2002.UK
Oxford.B5S1.22002.UK
96
Oxford.B5S19.2002.UK
99
Oxford.B5S13.2002.UK
Elsloo012.2004.NL
Apeldoorn023.2003.NL
74
GII.4.2002
DenHelder003.2004.NL
66
WeertE022.2002.NL
DenHaag001.2003.NL
Oxford.B6S6.2003.UK
Oxford.B6S4.2003.UK
Oxford.B6S5.2003.UK
90
Oxford.B5S22.2003.UK
Witney.B7S2.2003.UK
Warren.2002.USA
Banbury.B9S23.2003.UK
Oxford.B3S20.2003.UK
ChippingNorton.2003.UK
Didcot.B9S2.2003.UK
Oxford.B6S3.2003.UK
Oxford.B6S2.2003.UK
Oxford.B5S23.2003.UK
Oxford.B3S18.2003.UK
Oxford.B3S19.2003.UK
Oxford.B3S4.2003.UK
Oxford.B3S16.2003.UK
Oxford.B2S24.2002.UK
87
Oxford.B2S25.2002.UK
Oxford.B2S20.2002.UK
90
Oxford.B2S23.2002.UK
Oxford.B2S17.2002.UK
Oxford.B2S18.2002.UK
Oxford.B2S14.2002.UK
Oxford.B2S15.2002.UK
Oxford.B2S10.2002.UK
Oxford.B2S11.2002.UK
Oxford.B1S16.2002.UK
Oxford.B1S21.2002.UK
Oxford.B2S16.2002.UK
Oxford.B2S26.2002.UK
Oxford.B1S1.2002.UK
Oxford.B1S2.2002.UK
Oxford.B1S4.2002.UK
Oxford.B1S12.2002.UK
Oxford.B1S11.2002.UK
Lanzhou.35666.2002.China
Erfurt.007.00.DE

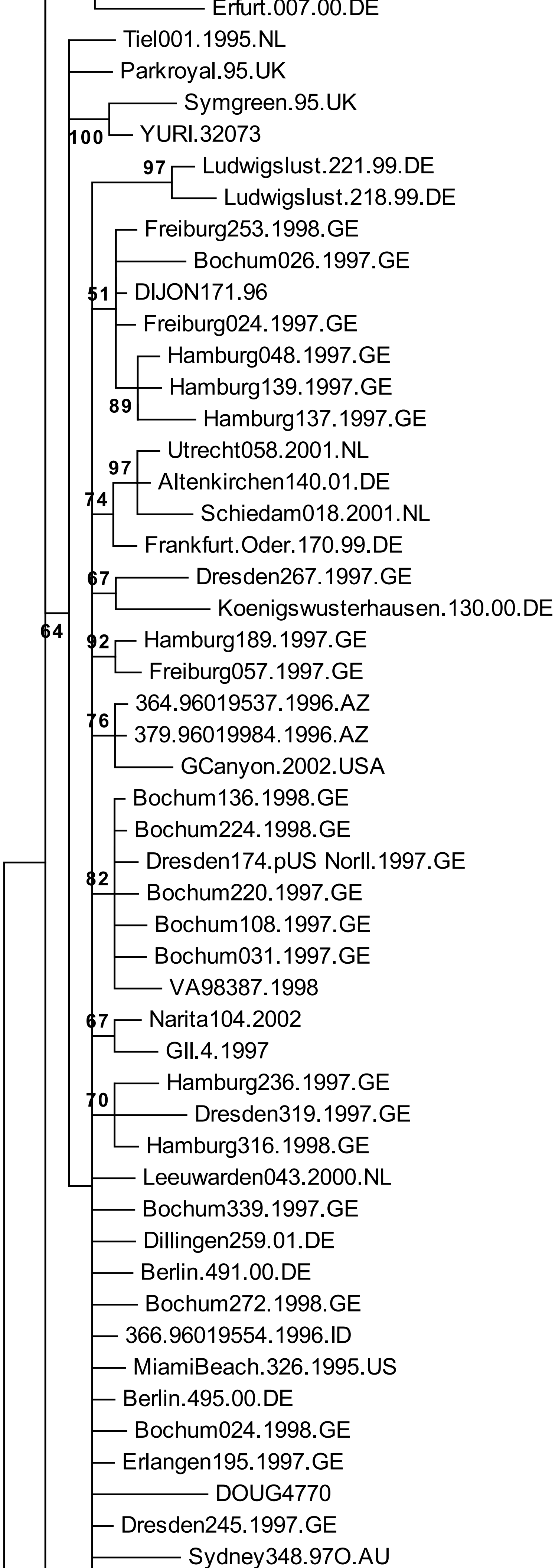
Erfurt.007.00.DE
Tiel001.1995.NL
Parkroyal.95.UK
Symgreen.95.UK
100
YURI.32073
97
Ludwigslust.221.99.DE
Ludwigslust.218.99.DE
Freiburg253.1998.GE
Bochum026.1997.GE
51
DIJON171.96
Freiburg024.1997.GE
Hamburg048.1997.GE
Hamburg139.1997.GE
89
Hamburg137.1997.GE
Utrecht058.2001.NL
97
Altenkirchen140.01.DE
74
Schiedam018.2001.NL
Frankfurt.Oder.170.99.DE
67
Dresden267.1997.GE
Koenigswusterhausen.130.00.DE
64
92
Hamburg189.1997.GE
Freiburg057.1997.GE
364.96019537.1996.AZ
76
379.96019984.1996.AZ
GCanyon.2002.USA
Bochum136.1998.GE
Bochum224.1998.GE
Dresden174.pUS NorII.1997.GE
82
Bochum220.1997.GE
Bochum108.1997.GE
Bochum031.1997.GE
VA98387.1998
67
Narita104.2002
GII.4.1997
Hamburg236.1997.GE
70
Dresden319.1997.GE
Hamburg316.1998.GE
Leeuwarden043.2000.NL
Bochum339.1997.GE
Dillingen259.01.DE
Berlin.491.00.DE
Bochum272.1998.GE
366.96019554.1996.ID
MiamiBeach.326.1995.US
Berlin.495.00.DE
Bochum024.1998.GE
Erlangen195.1997.GE
DOUG4770
Dresden245.1997.GE
Sydney348.97O.AU

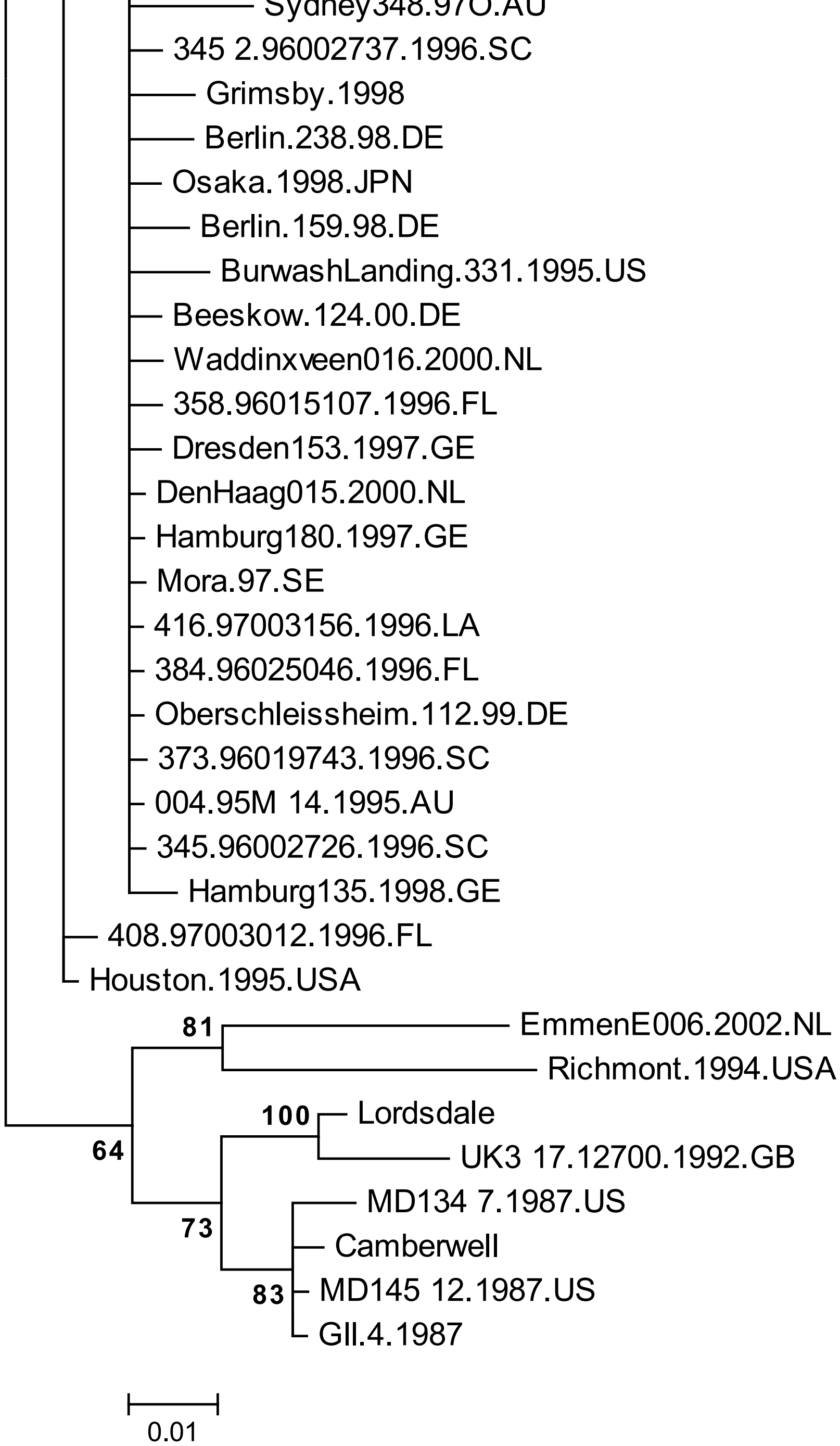

Sydney348.97O.AU
345 2.96002737.1996.SC
Grimsby.1998
Berlin.238.98.DE
Osaka.1998.JPN
Berlin.159.98.DE
BurwashLanding.331.1995.US
Beeskow.124.00.DE
Waddinxveen016.2000.NL
358.96015107.1996.FL
Dresden153.1997.GE
DenHaag015.2000.NL
Hamburg180.1997.GE
Mora.97.SE
416.97003156.1996.LA
384.96025046.1996.FL
Oberschleissheim.112.99.DE
373.96019743.1996.SC
004.95M 14.1995.AU
345.96002726.1996.SC
Hamburg135.1998.GE
408.97003012.1996.FL
Houston.1995.USA
81
EmmenE006.2002.NL
Richmont.1994.USA
100
Lordsdale
64
UK3 17.12700.1992.GB
MD134 7.1987.US
73
Camberwell
83
MD145 12.1987.US
GII.4.1987
0.01
