# Supplementary material for: Mechanisms of GII.4 Norovirus Persistence in Human Populations
Source: PLoS Med. 2008 Feb 12;5(2):e31. doi: 10.1371/journal.pmed.0050031 (PMC2235898; doi:10.1371/journal.pmed.0050031)
Supplement: Supplementary file 3 [file pmed.0050031.sg003.pdf]

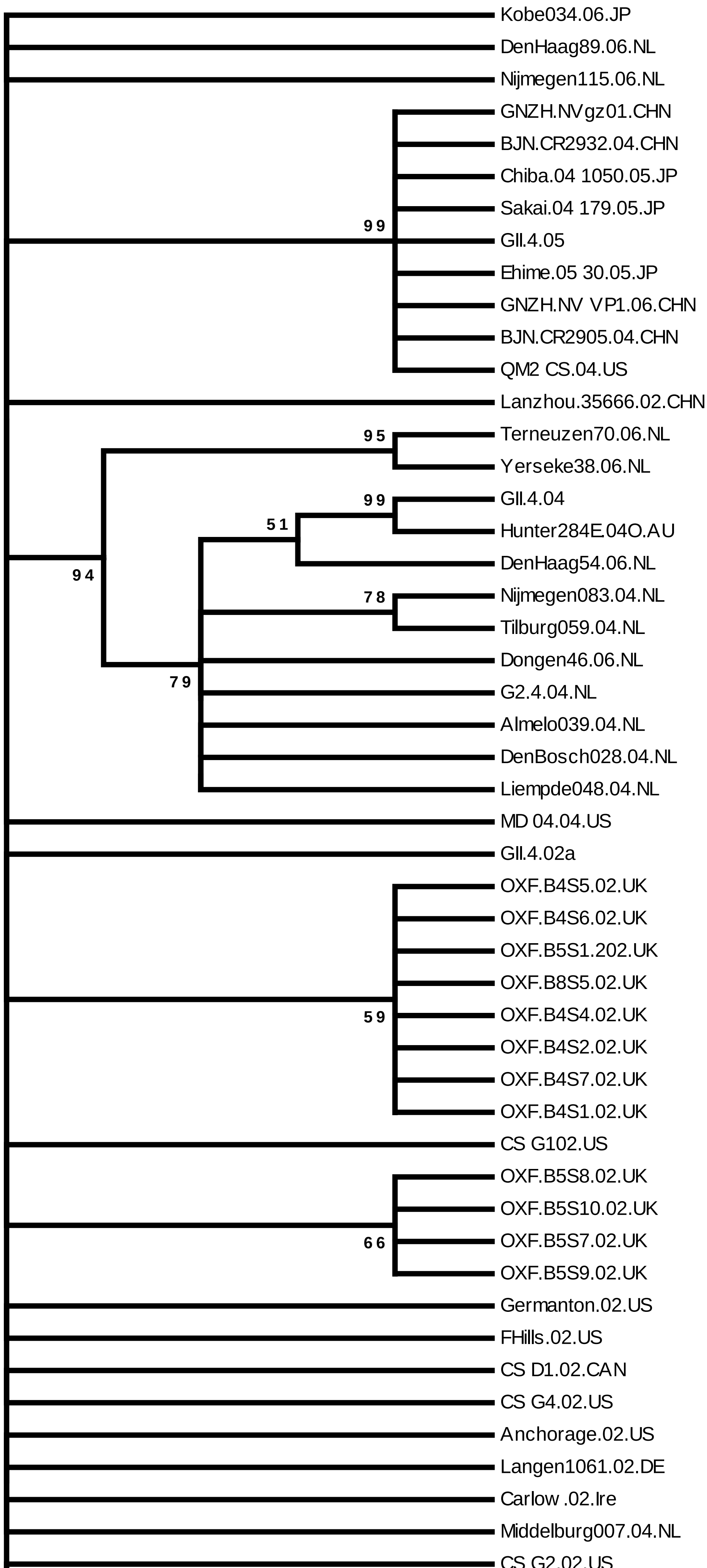
Kobe034.06.JP
DenHaag89.06.NL
Nijmegen115.06.NL
GNZH.NVgz01.CHN
BJN.CR2932.04.CHN
Chiba.04 1050.05.JP
Sakai.04 179.05.JP
99
GII.4.05
Ehime.05 30.05.JP
GNZH.NV VP1.06.CHN
BJN.CR2905.04.CHN
QM2 CS.04.US
Lanzhou.35666.02.CHN
95
Terneuzen70.06.NL
Yerseke38.06.NL
99
GII.4.04
51
Hunter284E.04O.AU
94
DenHaag54.06.NL
78
Nijmegen083.04.NL
Tilburg059.04.NL
Dongen46.06.NL
79
G2.4.04.NL
Almelo039.04.NL
DenBosch028.04.NL
Liempde048.04.NL
MD 04.04.US
GII.4.02a
OXF.B4S5.02.UK
OXF.B4S6.02.UK
OXF.B5S1.202.UK
OXF.B8S5.02.UK
OXF.B4S4.02.UK
59
OXF.B4S2.02.UK
OXF.B4S7.02.UK
OXF.B4S1.02.UK
CS G102.US
OXF.B5S8.02.UK
OXF.B5S10.02.UK
OXF.B5S7.02.UK
66
OXF.B5S9.02.UK
Germanton.02.US
FHills.02.US
CS D1.02.CAN
CS G4.02.US
Anchorage.02.US
Langen1061.02.DE
Carlow .02.Ire
Middelburg007.04.NL
CS G2.02.US

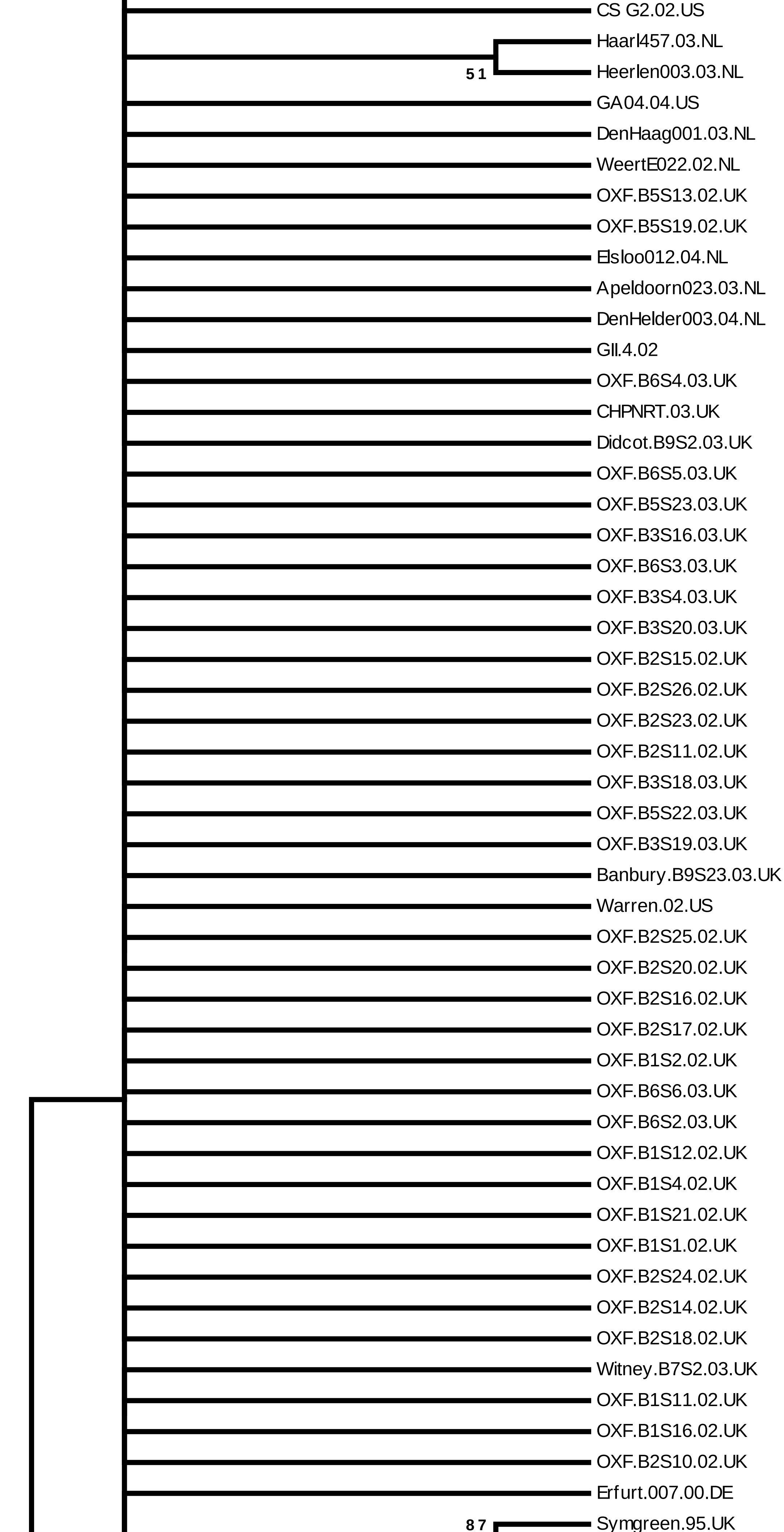
CS G2.02.US
Haarl457.03.NL
51
Heerlen003.03.NL
GA04.04.US
DenHaag001.03.NL
WeertE022.02.NL
OXF.B5S13.02.UK
OXF.B5S19.02.UK
Elsloo012.04.NL
Apeldoorn023.03.NL
DenHelder003.04.NL
GII.4.02
OXF.B6S4.03.UK
CHPNRT.03.UK
Didcot.B9S2.03.UK
OXF.B6S5.03.UK
OXF.B5S23.03.UK
OXF.B3S16.03.UK
OXF.B6S3.03.UK
OXF.B3S4.03.UK
OXF.B3S20.03.UK
OXF.B2S15.02.UK
OXF.B2S26.02.UK
OXF.B2S23.02.UK
OXF.B2S11.02.UK
OXF.B3S18.03.UK
OXF.B5S22.03.UK
OXF.B3S19.03.UK
Banbury.B9S23.03.UK
Warren.02.US
OXF.B2S25.02.UK
OXF.B2S20.02.UK
OXF.B2S16.02.UK
OXF.B2S17.02.UK
OXF.B1S2.02.UK
OXF.B6S6.03.UK
OXF.B6S2.03.UK
OXF.B1S12.02.UK
OXF.B1S4.02.UK
OXF.B1S21.02.UK
OXF.B1S1.02.UK
OXF.B2S24.02.UK
OXF.B2S14.02.UK
OXF.B2S18.02.UK
Witney.B7S2.03.UK
OXF.B1S11.02.UK
OXF.B1S16.02.UK
OXF.B2S10.02.UK
Erfurt.007.00.DE
87
Symgreen.95.UK

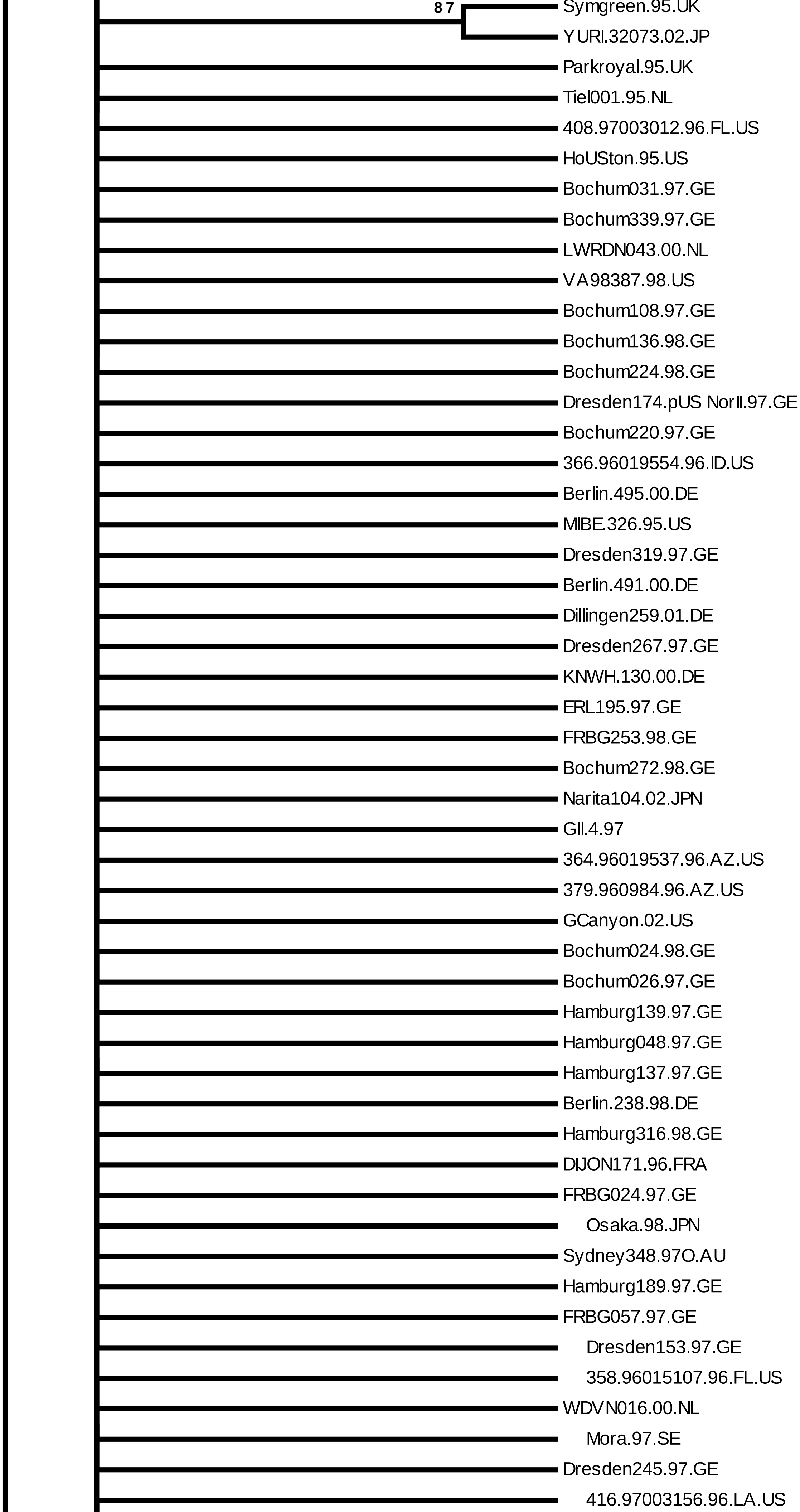

87
Symgreen.95.UK
YURI.32073.02.JP
Parkroyal.95.UK
Tiel001.95.NL
408.97003012.96.FL.US
HoUSton.95.US
Bochum031.97.GE
Bochum339.97.GE
LWRDN043.00.NL
VA98387.98.US
Bochum108.97.GE
Bochum136.98.GE
Bochum224.98.GE
Dresden174.pUS NorII.97.GE
Bochum220.97.GE
366.96019554.96.ID.US
Berlin.495.00.DE
MIBE.326.95.US
Dresden319.97.GE
Berlin.491.00.DE
Dillingen259.01.DE
Dresden267.97.GE
KNWH.130.00.DE
ERL195.97.GE
FRBG253.98.GE
Bochum272.98.GE
Narita104.02.JPN
GII.4.97
364.96019537.96.AZ.US
379.960984.96.AZ.US
GCanyon.02.US
Bochum024.98.GE
Bochum026.97.GE
Hamburg139.97.GE
Hamburg048.97.GE
Hamburg137.97.GE
Berlin.238.98.DE
Hamburg316.98.GE
DIJON171.96.FRA
FRBG024.97.GE
Osaka.98.JPN
Sydney348.97O.AU
Hamburg189.97.GE
FRBG057.97.GE
Dresden153.97.GE
358.96015107.96.FL.US
WDVN016.00.NL
Mora.97.SE
Dresden245.97.GE
416.97003156.96.LA.US

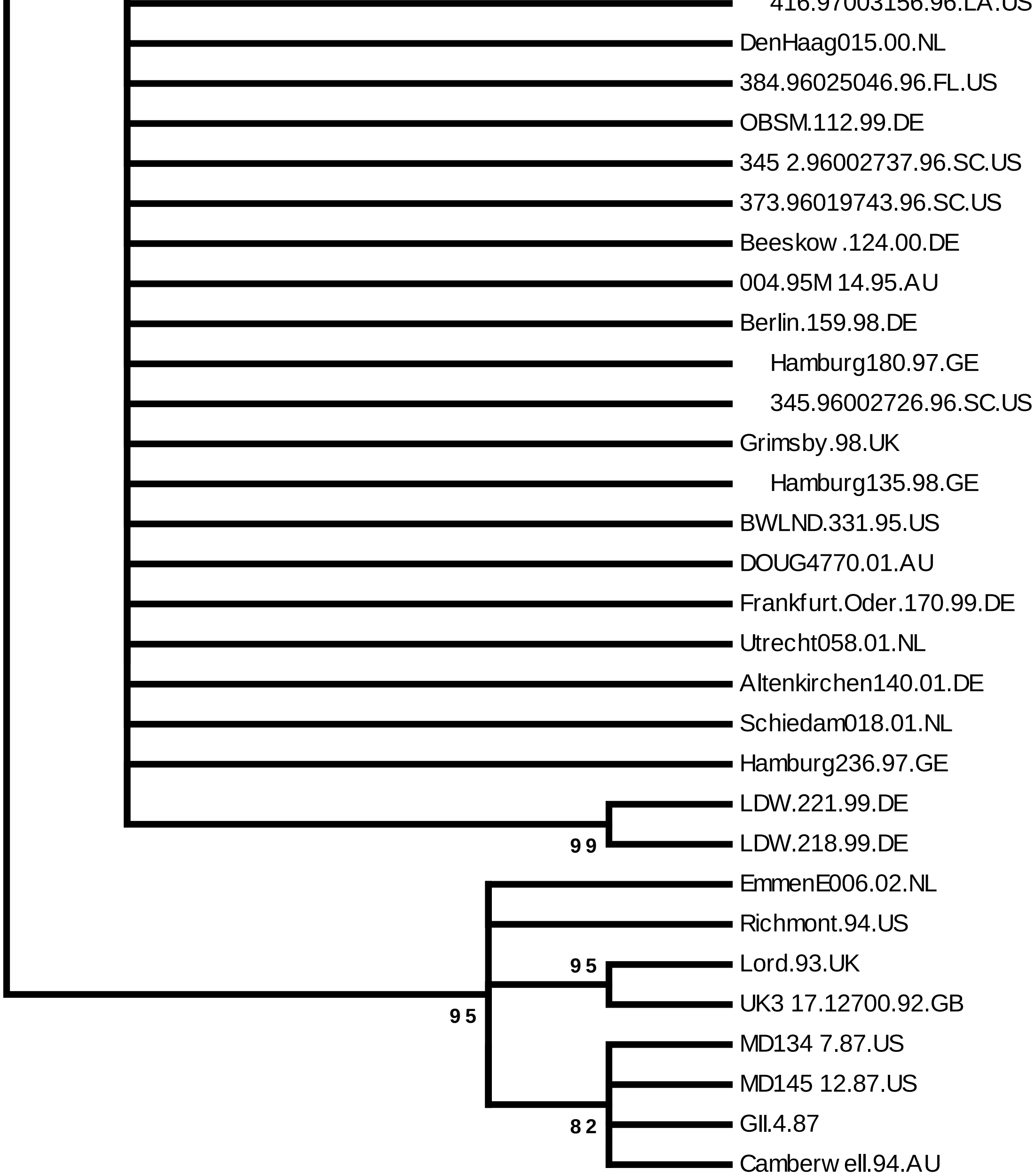
416.97003156.96.LA.US
DenHaag015.00.NL
384.96025046.96.FL.US
OBSM.112.99.DE
345 2.96002737.96.SC.US
373.96019743.96.SC.US
Beeskow .124.00.DE
004.95M 14.95.AU
Berlin.159.98.DE
Hamburg180.97.GE
345.96002726.96.SC.US
Grimsby.98.UK
Hamburg135.98.GE
BWLND.331.95.US
DOUG4770.01.AU
Frankfurt.Oder.170.99.DE
Utrecht058.01.NL
Altenkirchen140.01.DE
Schiedam018.01.NL
Hamburg236.97.GE
LDW.221.99.DE
99
LDW.218.99.DE
EmmenE006.02.NL
Richmont.94.US
95
Lord.93.UK
95
UK3 17.12700.92.GB
MD134 7.87.US
MD145 12.87.US
82
GII.4.87
Camberw ell.94.AU
