# Supplementary material for: Mechanisms of GII.4 Norovirus Persistence in Human Populations
Source: PLoS Med. 2008 Feb 12;5(2):e31. doi: 10.1371/journal.pmed.0050031 (PMC2235898; doi:10.1371/journal.pmed.0050031)
Supplement: Supplementary file 4 [file pmed.0050031.sg004.pdf]

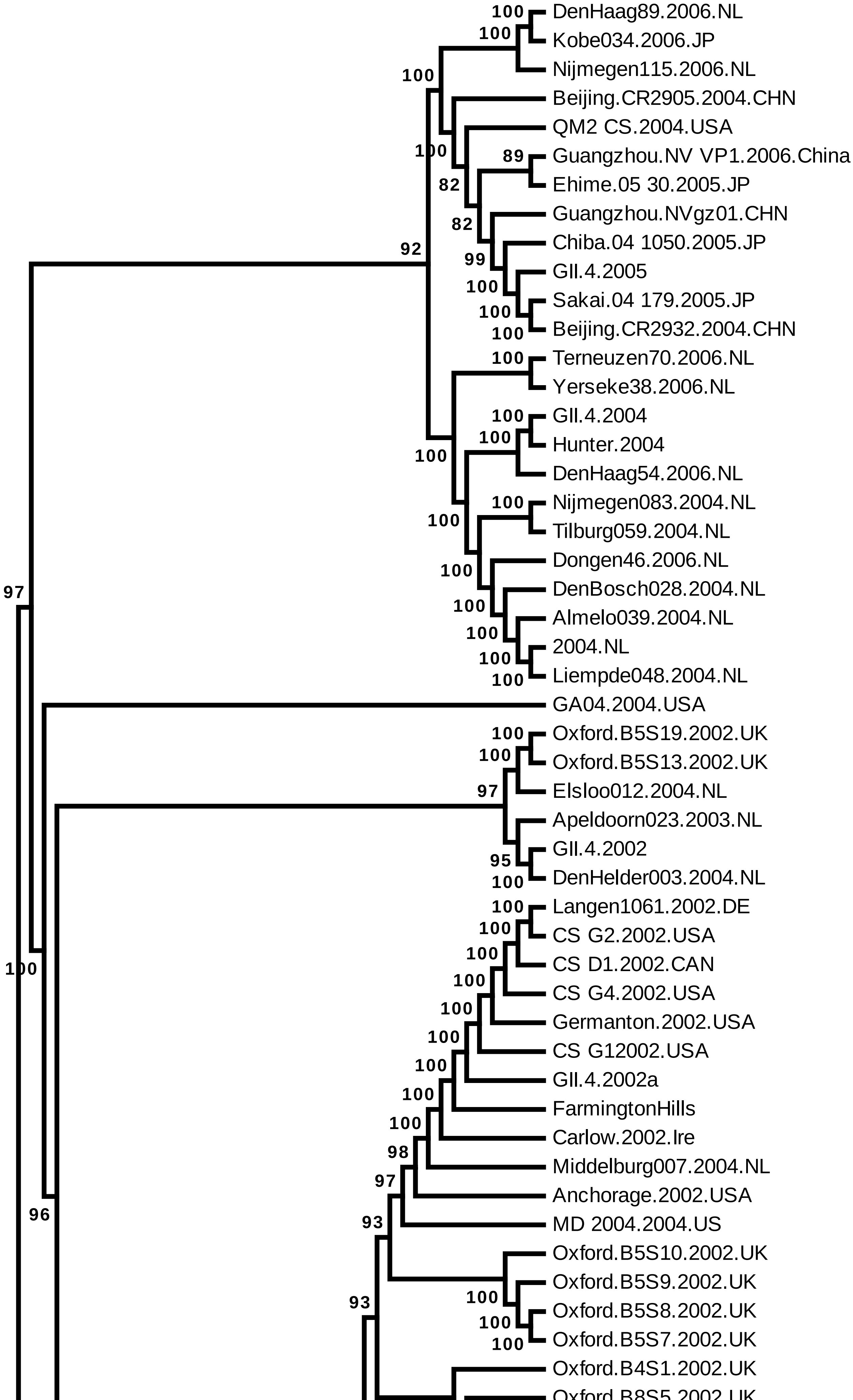
DenHaag89.2006.NL
Kobe034.2006.JP
Nijmegen115.2006.NL
Beijing.CR2905.2004.CHN
QM2 CS.2004.USA
Guangzhou.NV VP1.2006.China
Ehime.05 30.2005.JP
Guangzhou.NVgz01.CHN
Chiba.04 1050.2005.JP
GII.4.2005
Sakai.04 179.2005.JP
Beijing.CR2932.2004.CHN
Terneuzen70.2006.NL
Yerseke38.2006.NL
GII.4.2004
Hunter.2004
DenHaag54.2006.NL
Nijmegen083.2004.NL
Tilburg059.2004.NL
Dongen46.2006.NL
DenBosch028.2004.NL
Almelo039.2004.NL
2004.NL
Liempde048.2004.NL
GA04.2004.USA
Oxford.B5S19.2002.UK
Oxford.B5S13.2002.UK
Elsloo012.2004.NL
Apeldoorn023.2003.NL
GII.4.2002
DenHelder003.2004.NL
Langen1061.2002.DE
CS G2.2002.USA
CS D1.2002.CAN
CS G4.2002.USA
Germanton.2002.USA
CS G12002.USA
GII.4.2002a
FarmingtonHills
Carlow.2002.Ire
Middelburg007.2004.NL
Anchorage.2002.USA
MD 2004.2004.US
Oxford.B5S10.2002.UK
Oxford.B5S9.2002.UK
Oxford.B5S8.2002.UK
Oxford.B5S7.2002.UK
Oxford.B4S1.2002.UK

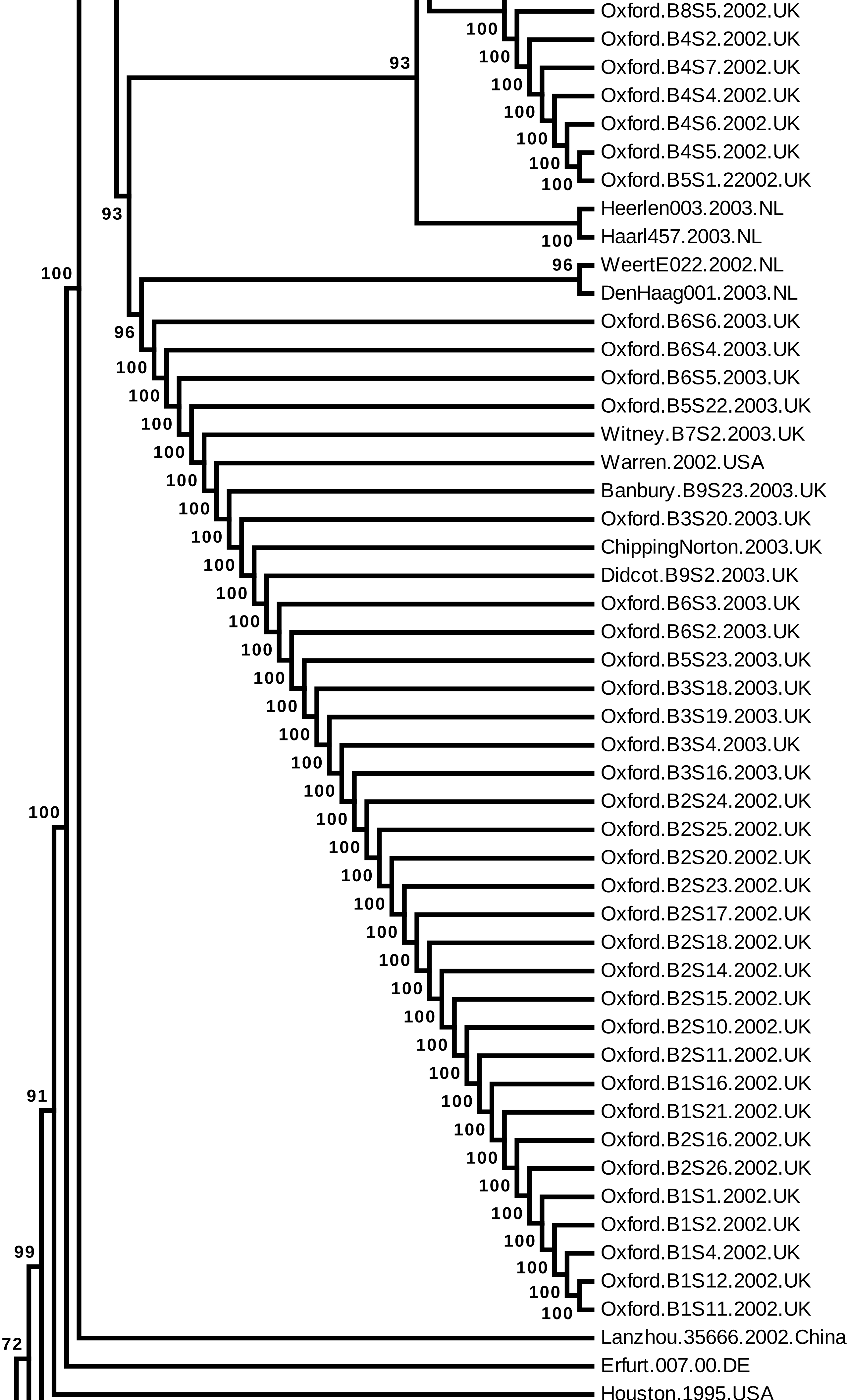
Oxford.B8S5.2002.UK
Oxford.B4S2.2002.UK
Oxford.B4S7.2002.UK
Oxford.B4S4.2002.UK
Oxford.B4S6.2002.UK
Oxford.B4S5.2002.UK
Oxford.B5S1.22002.UK
Heerlen003.2003.NL
Haarl457.2003.NL
WeertE022.2002.NL
DenHaag001.2003.NL
Oxford.B6S6.2003.UK
Oxford.B6S4.2003.UK
Oxford.B6S5.2003.UK
Oxford.B5S22.2003.UK
Witney.B7S2.2003.UK
Warren.2002.USA
Banbury.B9S23.2003.UK
Oxford.B3S20.2003.UK
ChippingNorton.2003.UK
Didcot.B9S2.2003.UK
Oxford.B6S3.2003.UK
Oxford.B6S2.2003.UK
Oxford.B5S23.2003.UK
Oxford.B3S18.2003.UK
Oxford.B3S19.2003.UK
Oxford.B3S4.2003.UK
Oxford.B3S16.2003.UK
Oxford.B2S24.2002.UK
Oxford.B2S25.2002.UK
Oxford.B2S20.2002.UK
Oxford.B2S23.2002.UK
Oxford.B2S17.2002.UK
Oxford.B2S18.2002.UK
Oxford.B2S14.2002.UK
Oxford.B2S15.2002.UK
Oxford.B2S10.2002.UK
Oxford.B2S11.2002.UK
Oxford.B1S16.2002.UK
Oxford.B1S21.2002.UK
Oxford.B2S16.2002.UK
Oxford.B2S26.2002.UK
Oxford.B1S1.2002.UK
Oxford.B1S2.2002.UK
Oxford.B1S4.2002.UK
Oxford.B1S12.2002.UK
Oxford.B1S11.2002.UK
Lanzhou.35666.2002.China
Erfurt.007.00.DE
Houston.1995.USA

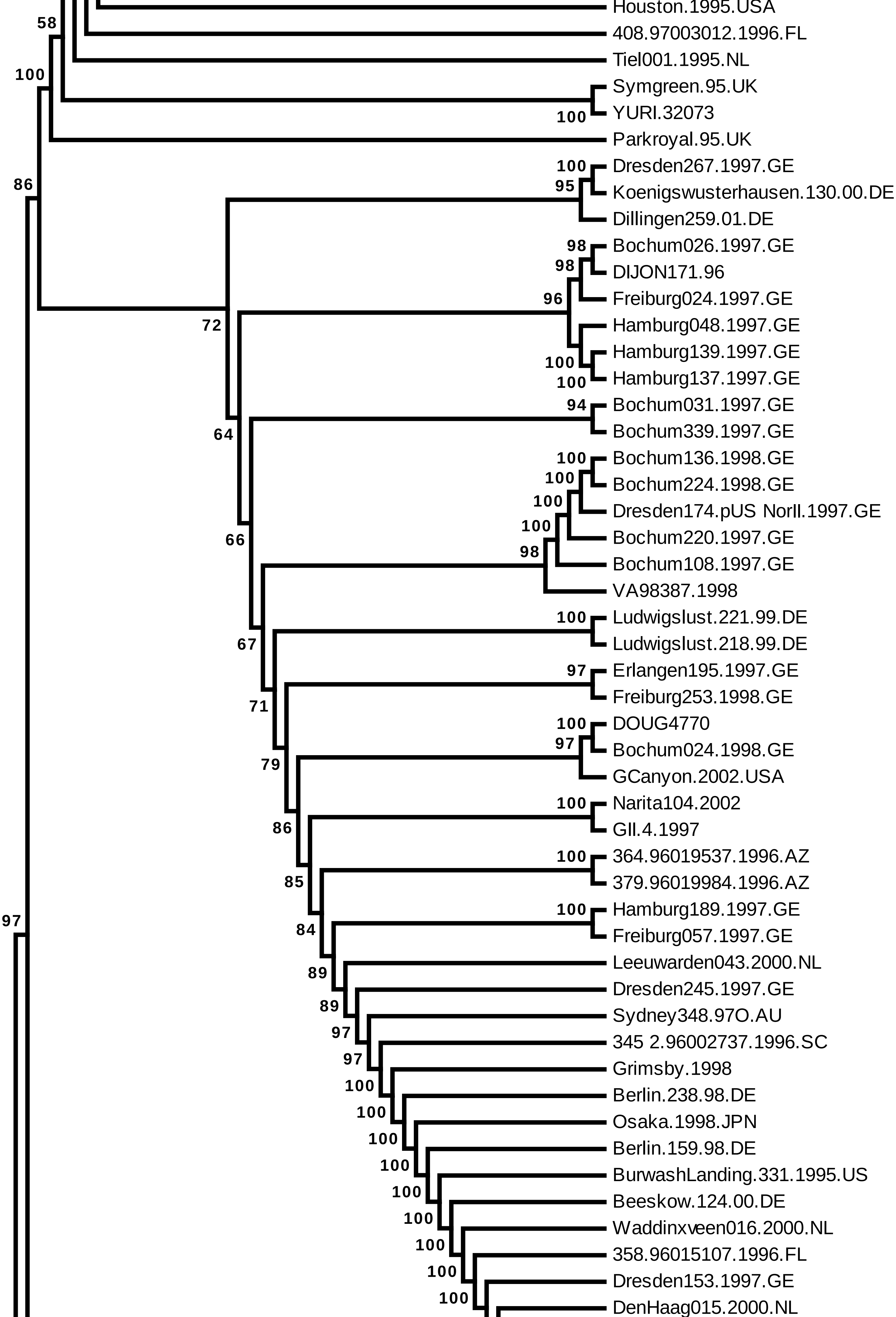
Houston.1995.USA
408.97003012.1996.FL
Tiel001.1995.NL
Symgreen.95.UK
YURI.32073
Parkroyal.95.UK
Dresden267.1997.GE
Koenigswusterhausen.130.00.DE
Dillingen259.01.DE
Bochum026.1997.GE
DIJON171.96
Freiburg024.1997.GE
Hamburg048.1997.GE
Hamburg139.1997.GE
Hamburg137.1997.GE
Bochum031.1997.GE
Bochum339.1997.GE
Bochum136.1998.GE
Bochum224.1998.GE
Dresden174.pUS NorII.1997.GE
Bochum220.1997.GE
Bochum108.1997.GE
VA98387.1998
Ludwigslust.221.99.DE
Ludwigslust.218.99.DE
Erlangen195.1997.GE
Freiburg253.1998.GE
DOUG4770
Bochum024.1998.GE
GCanyon.2002.USA
Narita104.2002
GII.4.1997
364.96019537.1996.AZ
379.96019984.1996.AZ
Hamburg189.1997.GE
Freiburg057.1997.GE
Leeuwarden043.2000.NL
Dresden245.1997.GE
Sydney348.97O.AU
345 2.96002737.1996.SC
Grimsby.1998
Berlin.238.98.DE
Osaka.1998.JPN
Berlin.159.98.DE
BurwashLanding.331.1995.US
Beeskow.124.00.DE
Waddinxveen016.2000.NL
358.96015107.1996.FL
Dresden153.1997.GE
DenHaag015.2000.NL

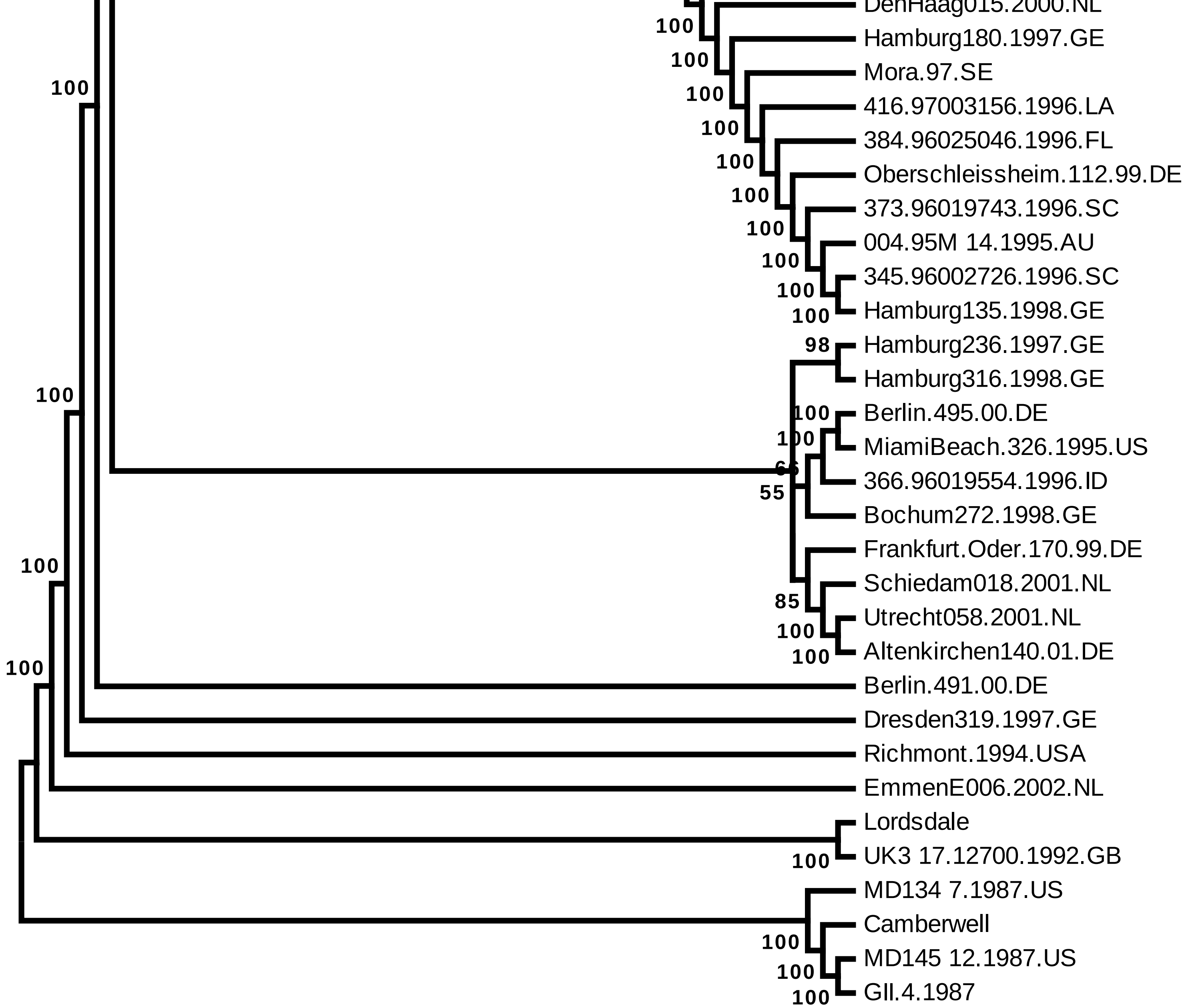

DenHaag015.2000.NL
Hamburg180.1997.GE
Mora.97.SE
416.97003156.1996.LA
384.96025046.1996.FL
Oberschleissheim.112.99.DE
373.96019743.1996.SC
004.95M 14.1995.AU
345.96002726.1996.SC
Hamburg135.1998.GE
Hamburg236.1997.GE
Hamburg316.1998.GE
Berlin.495.00.DE
MiamiBeach.326.1995.US
366.96019554.1996.ID
Bochum272.1998.GE
Frankfurt.Oder.170.99.DE
Schiedam018.2001.NL
Utrecht058.2001.NL
Altenkirchen140.01.DE
Berlin.491.00.DE
Dresden319.1997.GE
Richmont.1994.USA
EmmenE006.2002.NL
Lordsdale
UK3 17.12700.1992.GB
MD134 7.1987.US
Camberwell
MD145 12.1987.US
GII.4.1987
100
98
66
55
85
