# Supplementary material for: Mechanisms of GII.4 Norovirus Persistence in Human Populations
Source: PLoS Med. 2008 Feb 12;5(2):e31. doi: 10.1371/journal.pmed.0050031 (PMC2235898; doi:10.1371/journal.pmed.0050031)
Supplement: Supplementary file 5 [file pmed.0050031.sg005.pdf]

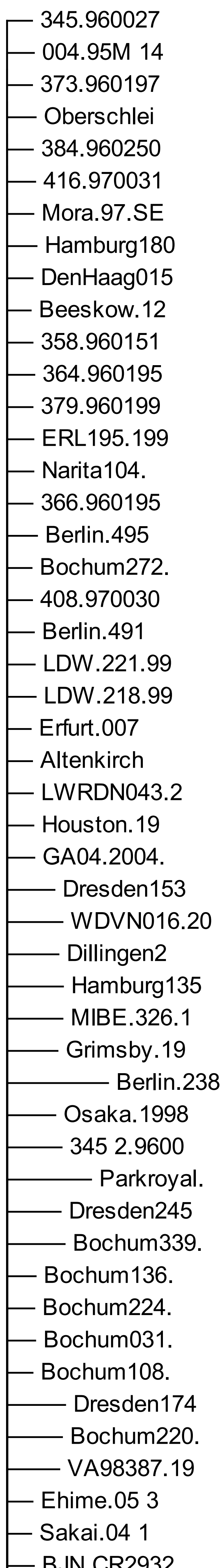

345.960027
004.95M 14
373.960197
Oberschlei
384.960250
416.970031
Mora.97.SE
Hamburg180
DenHaag015
Beeskow.12
358.960151
364.960195
379.960199
ERL195.199
Narita104.
366.960195
Berlin.495
Bochum272.
408.970030
Berlin.491
LDW.221.99
LDW.218.99
Erfurt.007
Altenkirch
LWRDN043.2
Houston.19
GA04.2004.
Dresden153
WDVN016.20
Dillingen2
Hamburg135
MIBE.326.1
Grimsby.19
Berlin.238
Osaka.1998
345 2.9600
Parkroyal.
Dresden245
Bochum339.
Bochum136.
Bochum224.
Bochum031.
Bochum108.
Dresden174
Bochum220.
VA98387.19
Ehime.05 3
Sakai.04 1

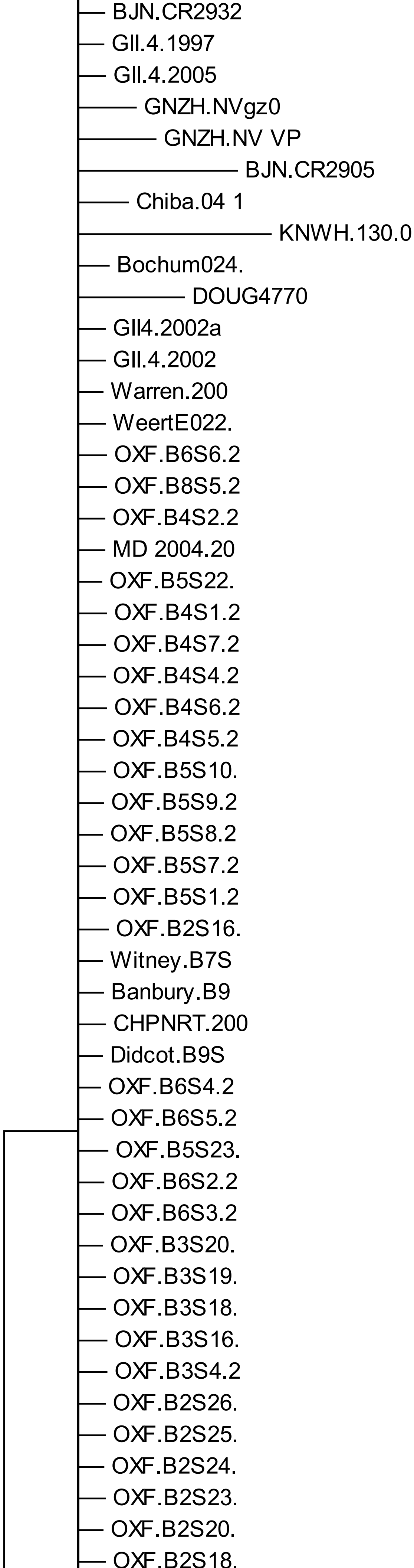
BJN.CR2932
GII.4.1997
GII.4.2005
GNZH.NVgz0
GNZH.NV VP
BJN.CR2905
Chiba.04 1
KNWH.130.0
Bochum024.
DOUG4770
GII4.2002a
GII.4.2002
Warren.200
WeertE022.
OXF.B6S6.2
OXF.B8S5.2
OXF.B4S2.2
MD 2004.20
OXF.B5S22.
OXF.B4S1.2
OXF.B4S7.2
OXF.B4S4.2
OXF.B4S6.2
OXF.B4S5.2
OXF.B5S10.
OXF.B5S9.2
OXF.B5S8.2
OXF.B5S7.2
OXF.B5S1.2
OXF.B2S16.
Witney.B7S
Banbury.B9
CHPNRT.200
Didcot.B9S
OXF.B6S4.2
OXF.B6S5.2
OXF.B5S23.
OXF.B6S2.2
OXF.B6S3.2
OXF.B3S20.
OXF.B3S19.
OXF.B3S18.
OXF.B3S16.
OXF.B3S4.2
OXF.B2S26.
OXF.B2S25.
OXF.B2S24.
OXF.B2S23.
OXF.B2S20.
OXF.B2S18.

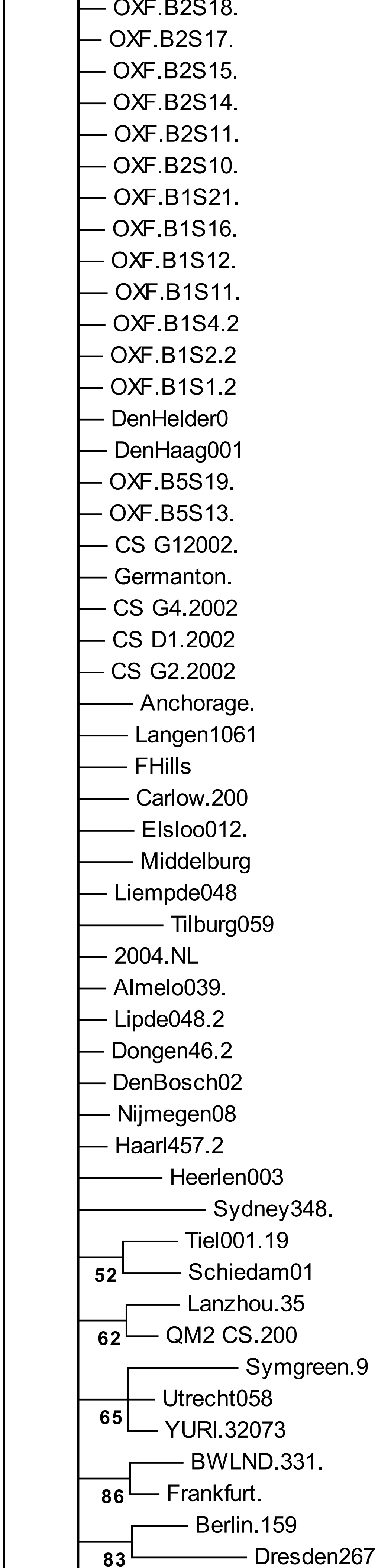
OXF.B2S18.
OXF.B2S17.
OXF.B2S15.
OXF.B2S14.
OXF.B2S11.
OXF.B2S10.
OXF.B1S21.
OXF.B1S16.
OXF.B1S12.
OXF.B1S11.
OXF.B1S4.2
OXF.B1S2.2
OXF.B1S1.2
DenHelder0
DenHaag001
OXF.B5S19.
OXF.B5S13.
CS G12002.
Germanton.
CS G4.2002
CS D1.2002
CS G2.2002
Anchorage.
Langen1061
FHills
Carlow.200
Elsloo012.
Middelburg
Liempde048
Tilburg059
2004.NL
Almelo039.
Lipde048.2
Dongen46.2
DenBosch02
Nijmegen08
Haarl457.2
Heerlen003
Sydney348.
Tiel001.19
Schiedam01
52
Lanzhou.35
QM2 CS.200
62
Symgreen.9
Utrecht058
YURI.32073
65
BWLND.331.
Frankfurt.
86
Berlin.159
Dresden267
83

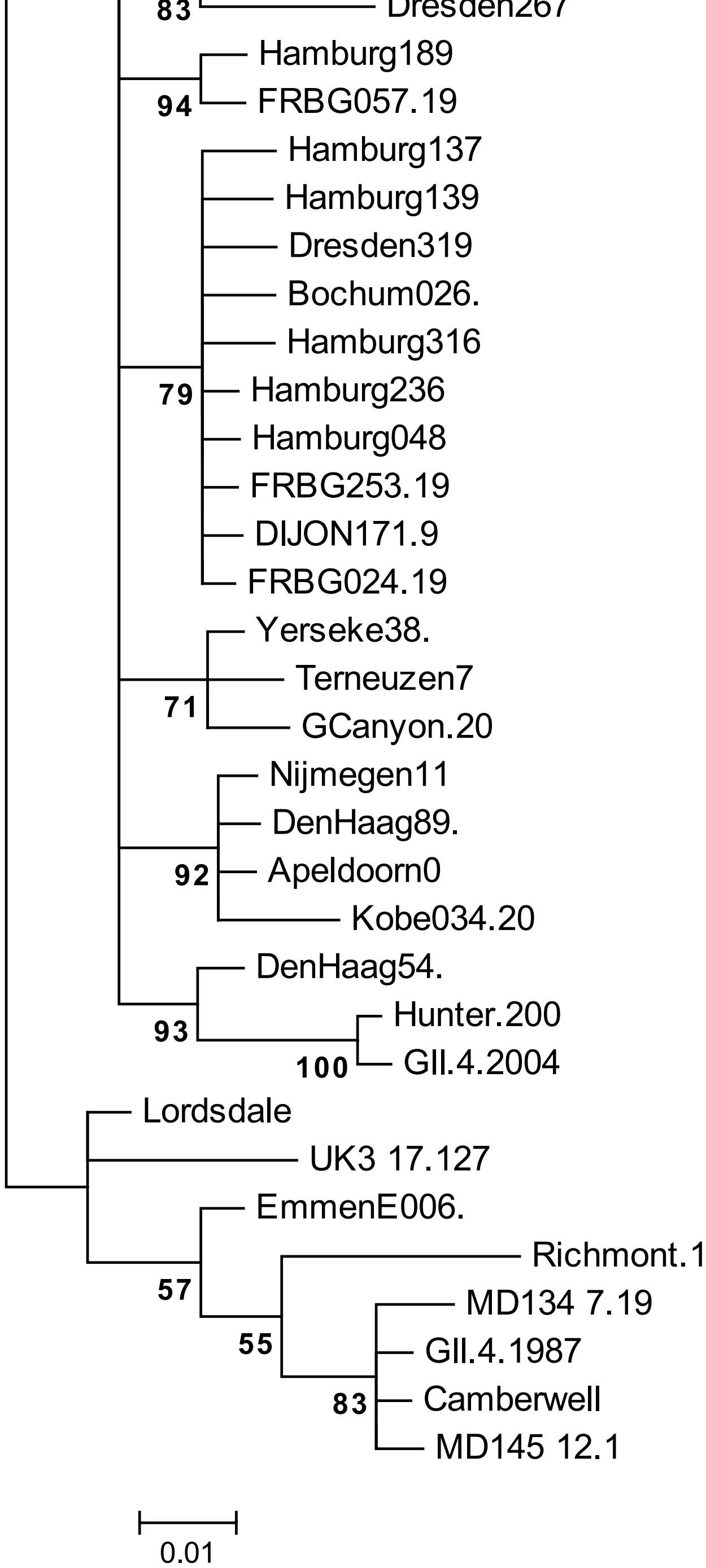

83
Dresden267
Hamburg189
94
FRBG057.19
Hamburg137
Hamburg139
Dresden319
Bochum026.
Hamburg316
79
Hamburg236
Hamburg048
FRBG253.19
DIJON171.9
FRBG024.19
Yerseke38.
Terneuzen7
71
GCanyon.20
Nijmegen11
DenHaag89.
92
Apeldoorn0
Kobe034.20
DenHaag54.
93
Hunter.200
100
GII.4.2004
Lordsdale
UK3 17.127
EmmenE006.
Richmont.1
57
MD134 7.19
55
GII.4.1987
83
Camberwell
MD145 12.1
0.01
