# Supplementary material for: Mechanisms of GII.4 Norovirus Persistence in Human Populations
Source: PLoS Med. 2008 Feb 12;5(2):e31. doi: 10.1371/journal.pmed.0050031 (PMC2235898; doi:10.1371/journal.pmed.0050031)
Supplement: Supplementary file 6 [file pmed.0050031.sg006.pdf]

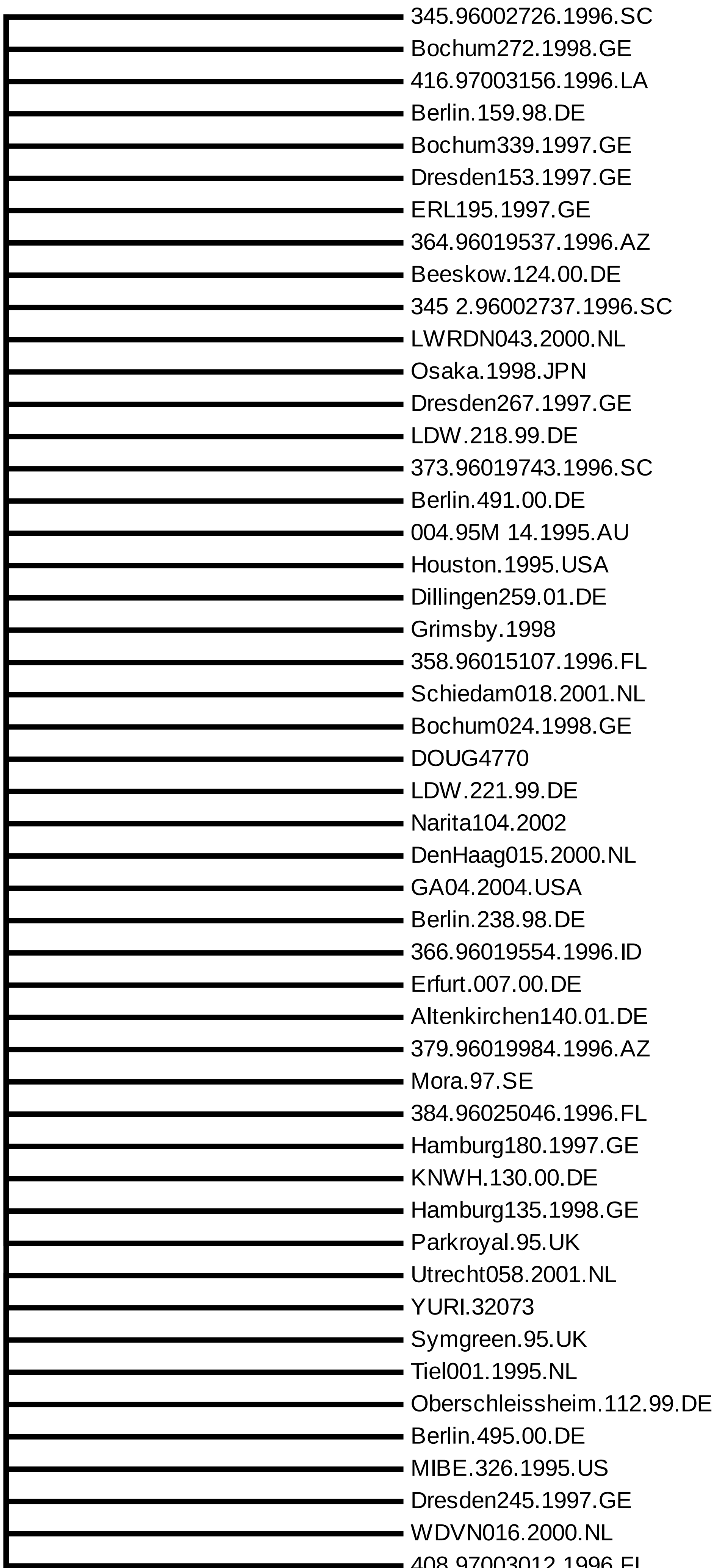
345.96002726.1996.SC
Bochum272.1998.GE
416.97003156.1996.LA
Berlin.159.98.DE
Bochum339.1997.GE
Dresden153.1997.GE
ERL195.1997.GE
364.96019537.1996.AZ
Beeskow.124.00.DE
345 2.96002737.1996.SC
LWRDN043.2000.NL
Osaka.1998.JPN
Dresden267.1997.GE
LDW.218.99.DE
373.96019743.1996.SC
Berlin.491.00.DE
004.95M 14.1995.AU
Houston.1995.USA
Dillingen259.01.DE
Grimsby.1998
358.96015107.1996.FL
Schiedam018.2001.NL
Bochum024.1998.GE
DOUG4770
LDW.221.99.DE
Narita104.2002
DenHaag015.2000.NL
GA04.2004.USA
Berlin.238.98.DE
366.96019554.1996.ID
Erfurt.007.00.DE
Altenkirchen140.01.DE
379.96019984.1996.AZ
Mora.97.SE
384.96025046.1996.FL
Hamburg180.1997.GE
KNWH.130.00.DE
Hamburg135.1998.GE
Parkroyal.95.UK
Utrecht058.2001.NL
YURI.32073
Symgreen.95.UK
Tiel001.1995.NL
Oberschleissheim.112.99.DE
Berlin.495.00.DE
MIBE.326.1995.US
Dresden245.1997.GE
WDVN016.2000.NL

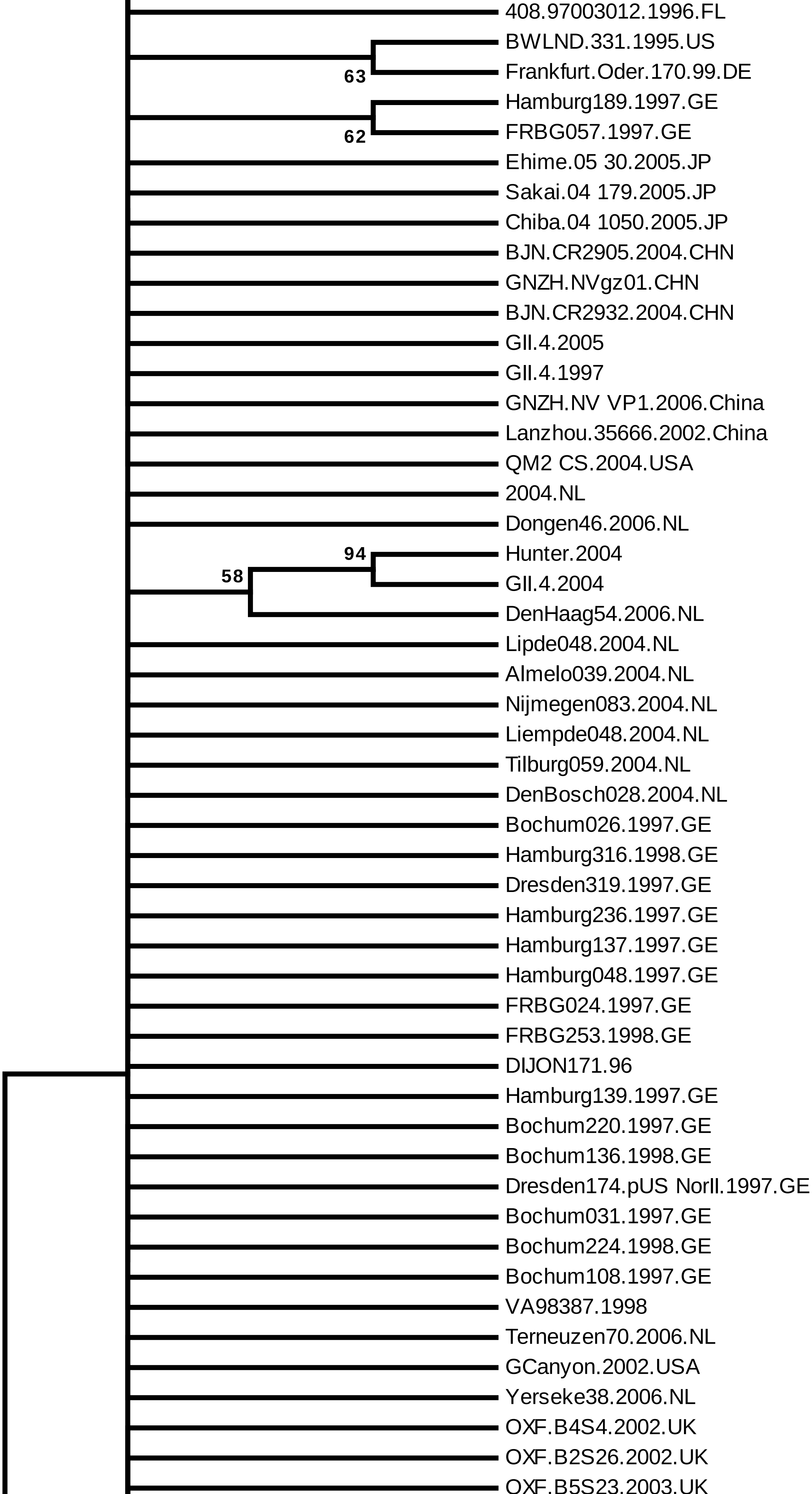
408.97003012.1996.FL
BWLND.331.1995.US
63
Frankfurt.Oder.170.99.DE
Hamburg189.1997.GE
62
FRBG057.1997.GE
Ehime.05 30.2005.JP
Sakai.04 179.2005.JP
Chiba.04 1050.2005.JP
BJN.CR2905.2004.CHN
GNZH.NVgz01.CHN
BJN.CR2932.2004.CHN
GII.4.2005
GII.4.1997
GNZH.NV VP1.2006.China
Lanzhou.35666.2002.China
QM2 CS.2004.USA
2004.NL
Dongen46.2006.NL
94
Hunter.2004
58
GII.4.2004
DenHaag54.2006.NL
Lipde048.2004.NL
Almelo039.2004.NL
Nijmegen083.2004.NL
Liempde048.2004.NL
Tilburg059.2004.NL
DenBosch028.2004.NL
Bochum026.1997.GE
Hamburg316.1998.GE
Dresden319.1997.GE
Hamburg236.1997.GE
Hamburg137.1997.GE
Hamburg048.1997.GE
FRBG024.1997.GE
FRBG253.1998.GE
DIJON171.96
Hamburg139.1997.GE
Bochum220.1997.GE
Bochum136.1998.GE
Dresden174.pUS NorII.1997.GE
Bochum031.1997.GE
Bochum224.1998.GE
Bochum108.1997.GE
VA98387.1998
Terneuzen70.2006.NL
GCanyon.2002.USA
Yerseke38.2006.NL
OXF.B4S4.2002.UK
OXF.B2S26.2002.UK
OXF.B5S23.2003.UK

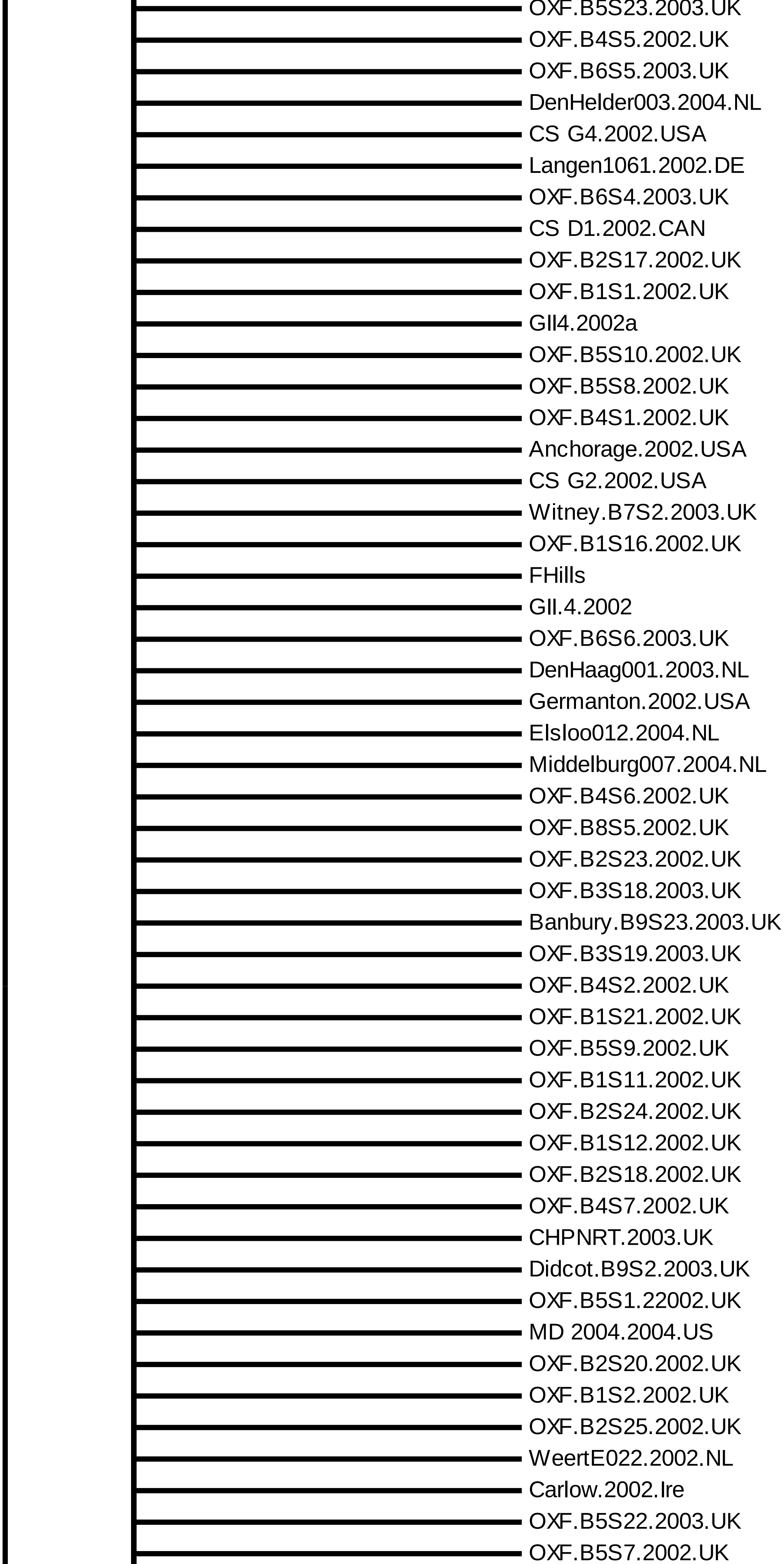
OXF.B5S23.2003.UK
OXF.B4S5.2002.UK
OXF.B6S5.2003.UK
DenHelder003.2004.NL
CS G4.2002.USA
Langen1061.2002.DE
OXF.B6S4.2003.UK
CS D1.2002.CAN
OXF.B2S17.2002.UK
OXF.B1S1.2002.UK
GII4.2002a
OXF.B5S10.2002.UK
OXF.B5S8.2002.UK
OXF.B4S1.2002.UK
Anchorage.2002.USA
CS G2.2002.USA
Witney.B7S2.2003.UK
OXF.B1S16.2002.UK
FHills
GII.4.2002
OXF.B6S6.2003.UK
DenHaag001.2003.NL
Germanton.2002.USA
Elsloo012.2004.NL
Middelburg007.2004.NL
OXF.B4S6.2002.UK
OXF.B8S5.2002.UK
OXF.B2S23.2002.UK
OXF.B3S18.2003.UK
Banbury.B9S23.2003.UK
OXF.B3S19.2003.UK
OXF.B4S2.2002.UK
OXF.B1S21.2002.UK
OXF.B5S9.2002.UK
OXF.B1S11.2002.UK
OXF.B2S24.2002.UK
OXF.B1S12.2002.UK
OXF.B2S18.2002.UK
OXF.B4S7.2002.UK
CHPNRT.2003.UK
Didcot.B9S2.2003.UK
OXF.B5S1.22002.UK
MD 2004.2004.US
OXF.B2S20.2002.UK
OXF.B1S2.2002.UK
OXF.B2S25.2002.UK
WeertE022.2002.NL
Carlow.2002.Ire
OXF.B5S22.2003.UK
OXF.B5S7.2002.UK

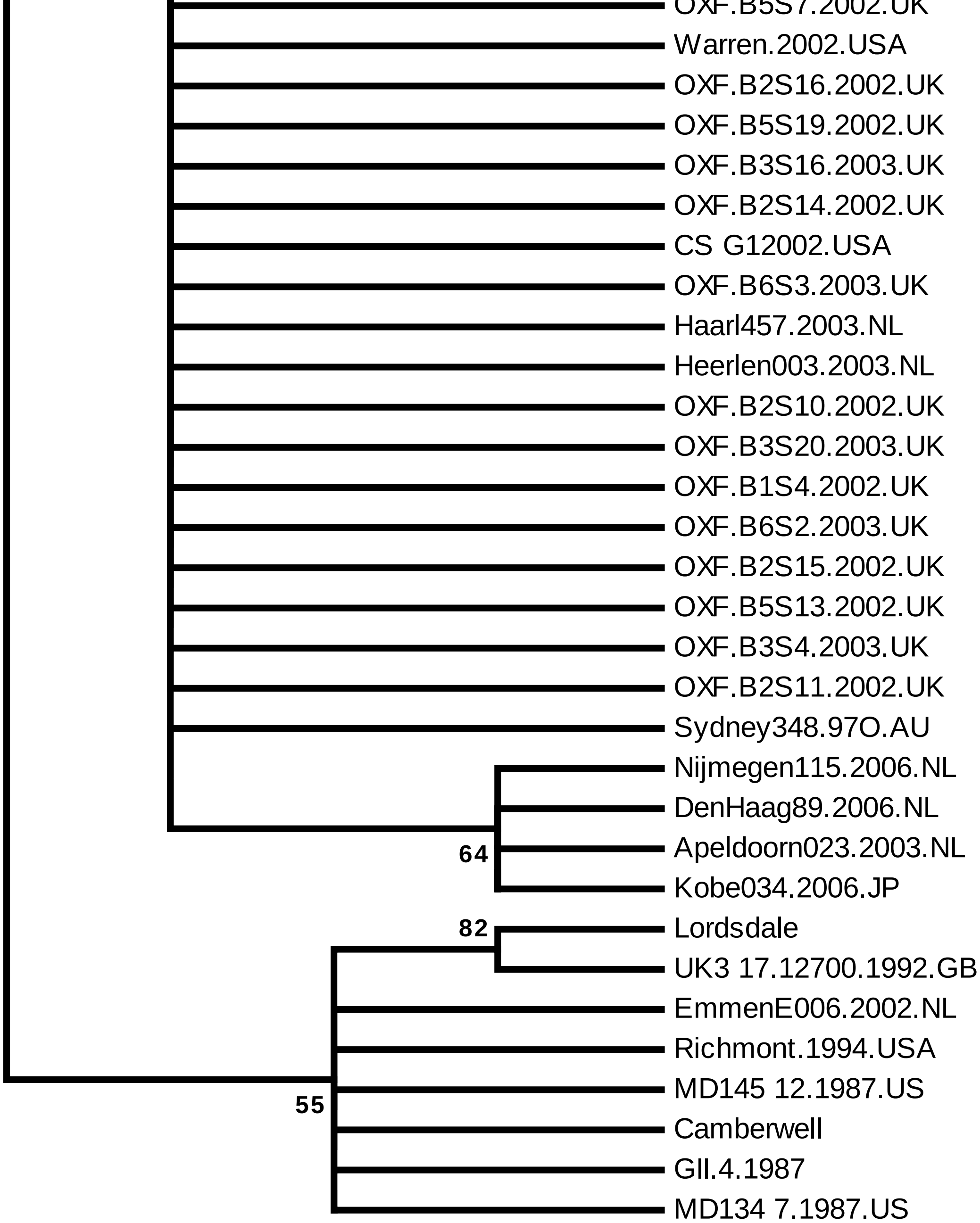
OXF.B5S7.2002.UK
Warren.2002.USA
OXF.B2S16.2002.UK
OXF.B5S19.2002.UK
OXF.B3S16.2003.UK
OXF.B2S14.2002.UK
CS G12002.USA
OXF.B6S3.2003.UK
Haarl457.2003.NL
Heerlen003.2003.NL
OXF.B2S10.2002.UK
OXF.B3S20.2003.UK
OXF.B1S4.2002.UK
OXF.B6S2.2003.UK
OXF.B2S15.2002.UK
OXF.B5S13.2002.UK
OXF.B3S4.2003.UK
OXF.B2S11.2002.UK
Sydney348.97O.AU
Nijmegen115.2006.NL
DenHaag89.2006.NL
64
Apeldoorn023.2003.NL
Kobe034.2006.JP
82
Lordsdale
UK3 17.12700.1992.GB
EmmenE006.2002.NL
Richmont.1994.USA
MD145 12.1987.US
55
Camberwell
GII.4.1987
MD134 7.1987.US
