# Supplementary material for: Mechanisms of GII.4 Norovirus Persistence in Human Populations
Source: PLoS Med. 2008 Feb 12;5(2):e31. doi: 10.1371/journal.pmed.0050031 (PMC2235898; doi:10.1371/journal.pmed.0050031)
Supplement: Supplementary file 7 [file pmed.0050031.sg007.pdf]

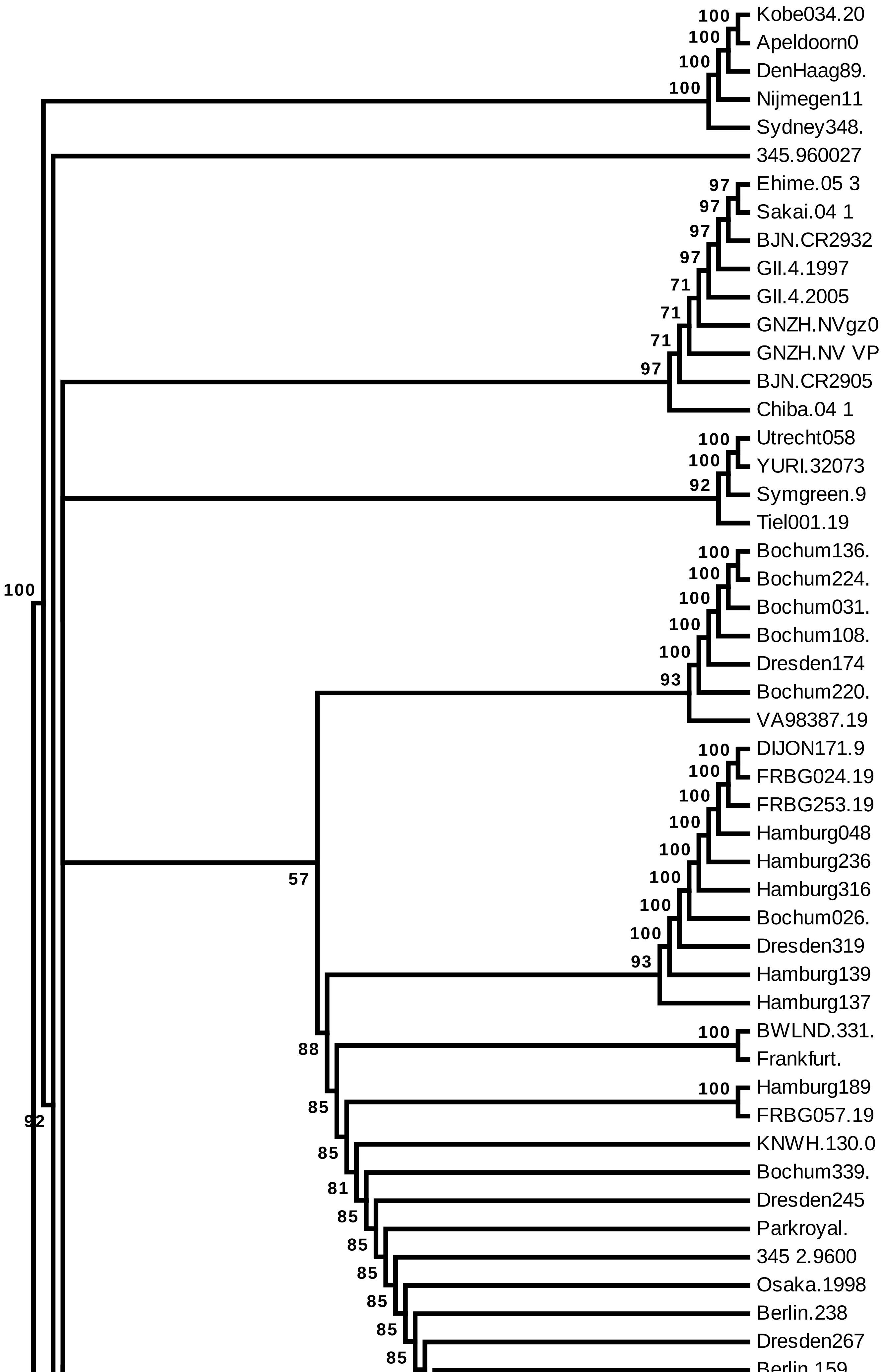

Kobe034.20
Apeldoorn0
DenHaag89.
Nijmegen11
Sydney348.
345.960027
Ehime.05 3
Sakai.04 1
BJN.CR2932
GII.4.1997
GII.4.2005
GNZH.NVgz0
GNZH.NV VP
BJN.CR2905
Chiba.04 1
Utrecht058
YURI.32073
Symgreen.9
Tiel001.19
Bochum136.
Bochum224.
Bochum031.
Bochum108.
Dresden174
Bochum220.
VA98387.19
DIJON171.9
FRBG024.19
FRBG253.19
Hamburg048
Hamburg236
Hamburg316
Bochum026.
Dresden319
Hamburg139
Hamburg137
BWLND.331.
Frankfurt.
Hamburg189
FRBG057.19
KNWH.130.0
Bochum339.
Dresden245
Parkroyal.
345 2.9600
Osaka.1998
Berlin.238
Dresden267
Berlin.159

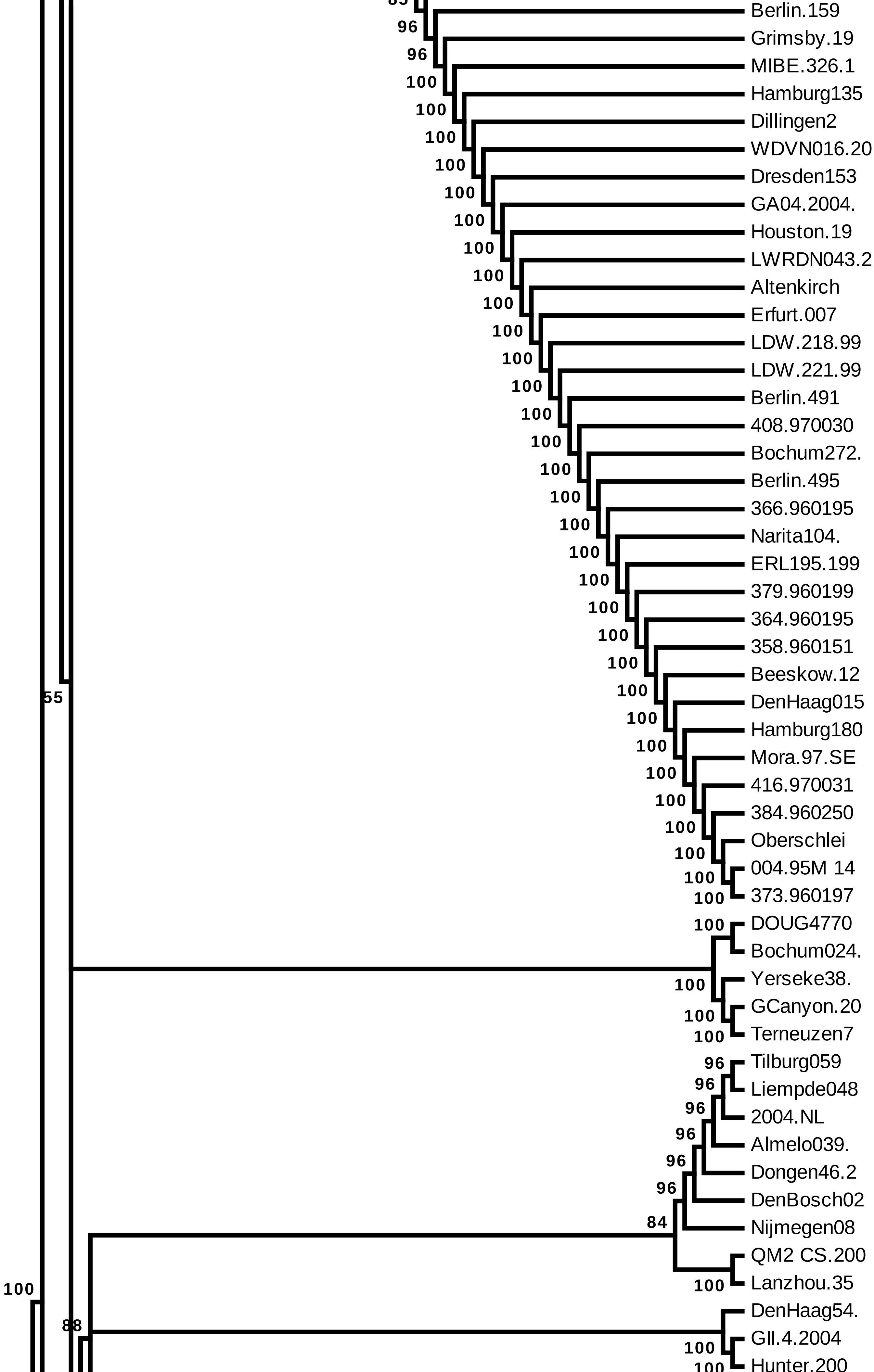
Berlin.159
Grimsby.19
MIBE.326.1
Hamburg135
Dillingen2
WDVN016.20
Dresden153
GA04.2004.
Houston.19
LWRDN043.2
Altenkirch
Erfurt.007
LDW.218.99
LDW.221.99
Berlin.491
408.970030
Bochum272.
Berlin.495
366.960195
Narita104.
ERL195.199
379.960199
364.960195
358.960151
Beeskow.12
DenHaag015
Hamburg180
Mora.97.SE
416.970031
384.960250
Oberschlei
004.95M 14
373.960197
DOUG4770
Bochum024.
Yerseke38.
GCanyon.20
Terneuzen7
Tilburg059
Liempde048
2004.NL
Almelo039.
Dongen46.2
DenBosch02
Nijmegen08
QM2 CS.200
Lanzhou.35
DenHaag54.
GII.4.2004
Hunter.200

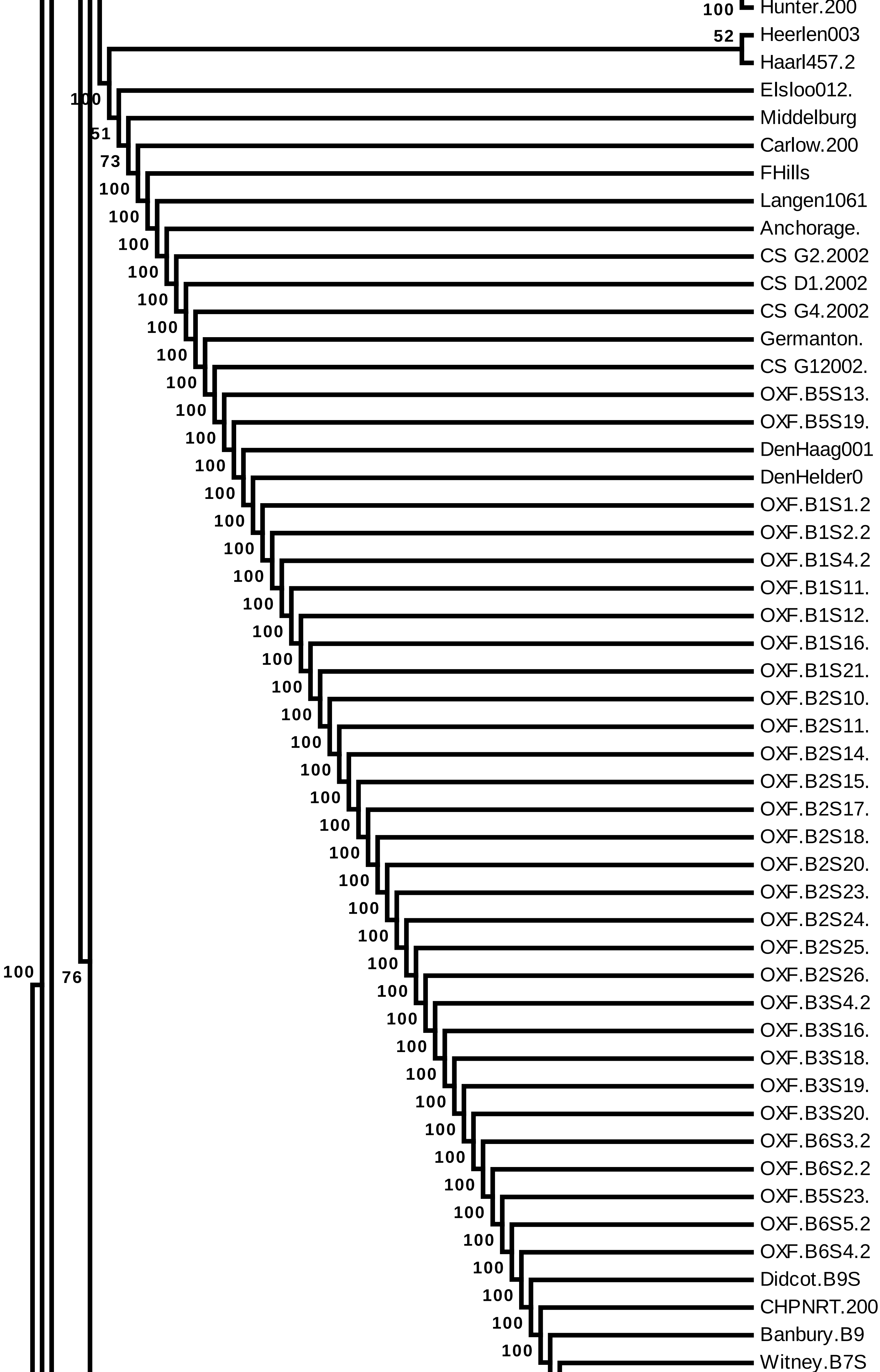
Hunter.200
Heerlen003
Haarl457.2
Elsloo012.
Middelburg
Carlow.200
FHills
Langen1061
Anchorage.
CS G2.2002
CS D1.2002
CS G4.2002
Germanton.
CS G12002.
OXF.B5S13.
OXF.B5S19.
DenHaag001
DenHelder0
OXF.B1S1.2
OXF.B1S2.2
OXF.B1S4.2
OXF.B1S11.
OXF.B1S12.
OXF.B1S16.
OXF.B1S21.
OXF.B2S10.
OXF.B2S11.
OXF.B2S14.
OXF.B2S15.
OXF.B2S17.
OXF.B2S18.
OXF.B2S20.
OXF.B2S23.
OXF.B2S24.
OXF.B2S25.
OXF.B2S26.
OXF.B3S4.2
OXF.B3S16.
OXF.B3S18.
OXF.B3S19.
OXF.B3S20.
OXF.B6S3.2
OXF.B6S2.2
OXF.B5S23.
OXF.B6S5.2
OXF.B6S4.2
Didcot.B9S
CHPNRT.200
Banbury.B9
Witney.B7S

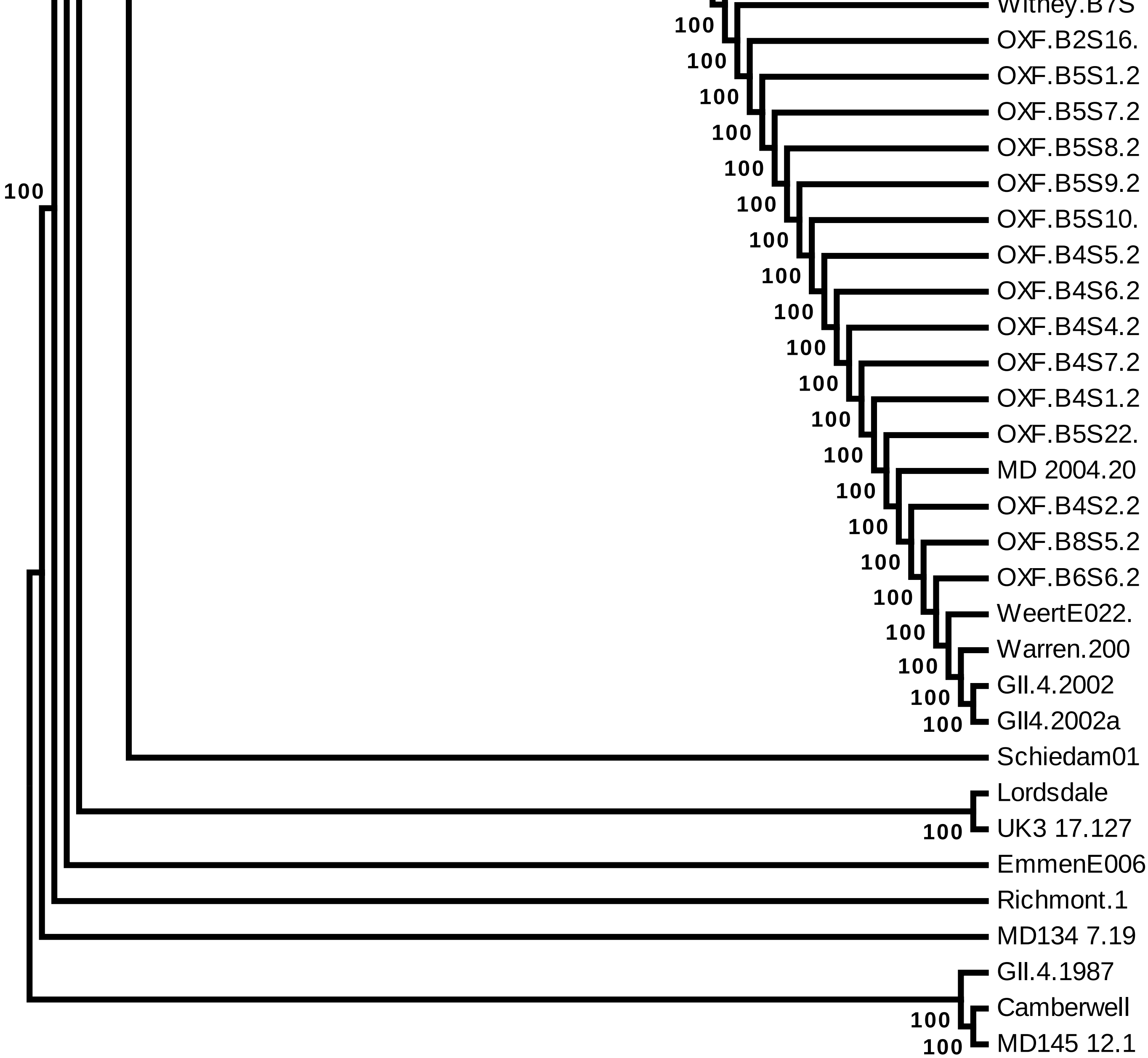

Witney.B7S
OXF.B2S16.
OXF.B5S1.2
OXF.B5S7.2
OXF.B5S8.2
OXF.B5S9.2
OXF.B5S10.
OXF.B4S5.2
OXF.B4S6.2
OXF.B4S4.2
OXF.B4S7.2
OXF.B4S1.2
OXF.B5S22.
MD 2004.20
OXF.B4S2.2
OXF.B8S5.2
OXF.B6S6.2
WeertE022.
Warren.200
GII.4.2002
GII4.2002a
Schiedam01
Lordsdale
UK3 17.127
EmmenE006.
Richmont.1
MD134 7.19
GII.4.1987
Camberwell
MD145 12.1
100
