# Supplementary material for: Mechanisms of GII.4 Norovirus Persistence in Human Populations
Source: PLoS Med. 2008 Feb 12;5(2):e31. doi: 10.1371/journal.pmed.0050031 (PMC2235898; doi:10.1371/journal.pmed.0050031)
Supplement: Supplementary file 8 [file pmed.0050031.sg008.pdf]

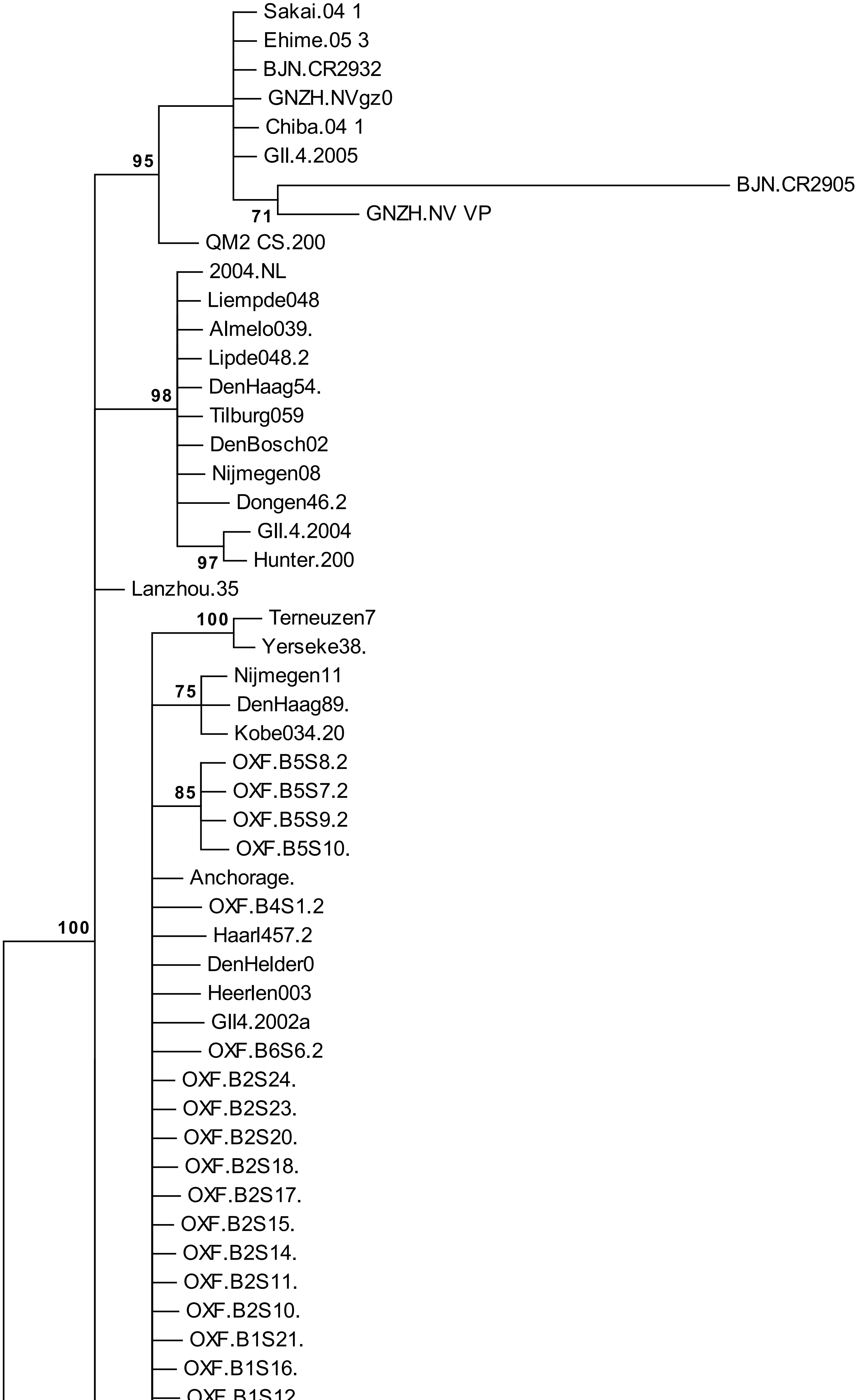

Sakai.04 1
Ehime.05 3
BJN.CR2932
GNZH.NVgz0
Chiba.04 1
GII.4.2005
95
BJN.CR2905
71
GNZH.NV VP
QM2 CS.200
2004.NL
Liempde048
Almelo039.
Lipde048.2
DenHaag54.
98
Tilburg059
DenBosch02
Nijmegen08
Dongen46.2
GII.4.2004
97
Hunter.200
Lanzhou.35
100
Terneuzen7
Yerseke38.
Nijmegen11
75
DenHaag89.
Kobe034.20
OXF.B5S8.2
85
OXF.B5S7.2
OXF.B5S9.2
OXF.B5S10.
Anchorage.
OXF.B4S1.2
100
Haarl457.2
DenHelder0
Heerlen003
GII4.2002a
OXF.B6S6.2
OXF.B2S24.
OXF.B2S23.
OXF.B2S20.
OXF.B2S18.
OXF.B2S17.
OXF.B2S15.
OXF.B2S14.
OXF.B2S11.
OXF.B2S10.
OXF.B1S21.
OXF.B1S16.

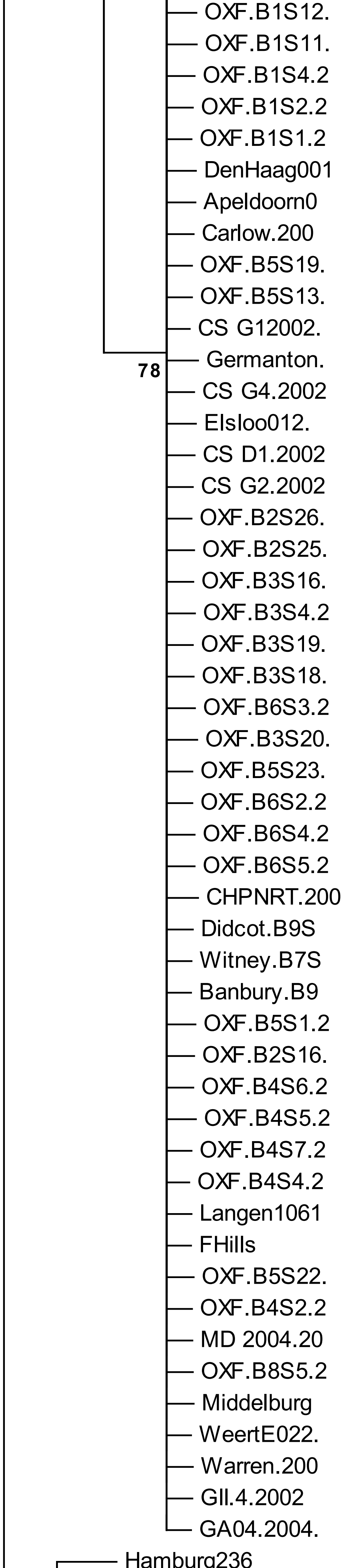
OXF.B1S12.
OXF.B1S11.
OXF.B1S4.2
OXF.B1S2.2
OXF.B1S1.2
DenHaag001
Apeldoorn0
Carlow.200
OXF.B5S19.
OXF.B5S13.
CS G12002.
Germanton.
78
CS G4.2002
Elsloo012.
CS D1.2002
CS G2.2002
OXF.B2S26.
OXF.B2S25.
OXF.B3S16.
OXF.B3S4.2
OXF.B3S19.
OXF.B3S18.
OXF.B6S3.2
OXF.B3S20.
OXF.B5S23.
OXF.B6S2.2
OXF.B6S4.2
OXF.B6S5.2
CHPNRT.200
Didcot.B9S
Witney.B7S
Banbury.B9
OXF.B5S1.2
OXF.B2S16.
OXF.B4S6.2
OXF.B4S5.2
OXF.B4S7.2
OXF.B4S4.2
Langen1061
FHills
OXF.B5S22.
OXF.B4S2.2
MD 2004.20
OXF.B8S5.2
Middelburg
WeertE022.
Warren.200
GII.4.2002
GA04.2004.
Hamburg236

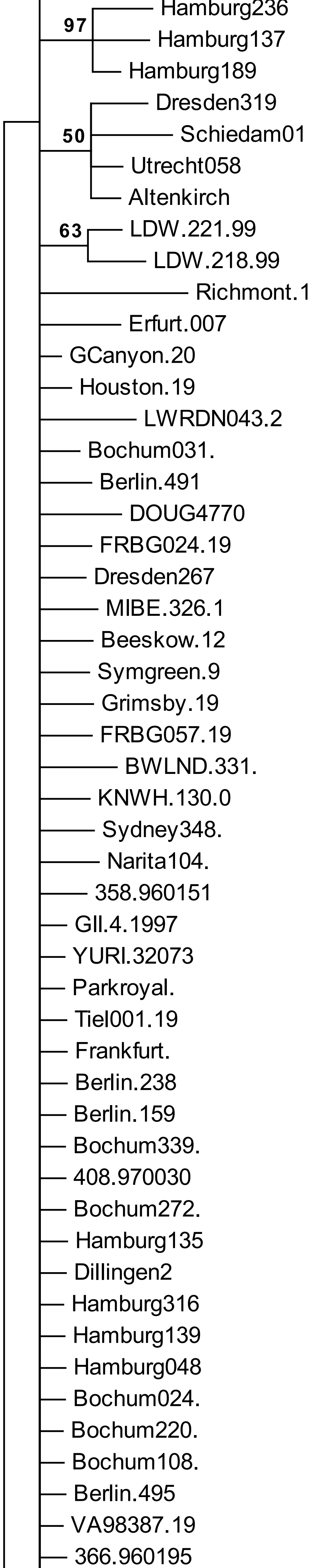
97
Hamburg236
Hamburg137
Hamburg189
50
Dresden319
Schiedam01
Utrecht058
Altenkirch
63
LDW.221.99
LDW.218.99
Richmont.1
Erfurt.007
GCanyon.20
Houston.19
LWRDN043.2
Bochum031.
Berlin.491
DOUG4770
FRBG024.19
Dresden267
MIBE.326.1
Beeskow.12
Symgreen.9
Grimsby.19
FRBG057.19
BWLND.331.
KNWH.130.0
Sydney348.
Narita104.
358.960151
GII.4.1997
YURI.32073
Parkroyal.
Tiel001.19
Frankfurt.
Berlin.238
Berlin.159
Bochum339.
408.970030
Bochum272.
Hamburg135
Dillingen2
Hamburg316
Hamburg139
Hamburg048
Bochum024.
Bochum220.
Bochum108.
Berlin.495
VA98387.19
366.960195

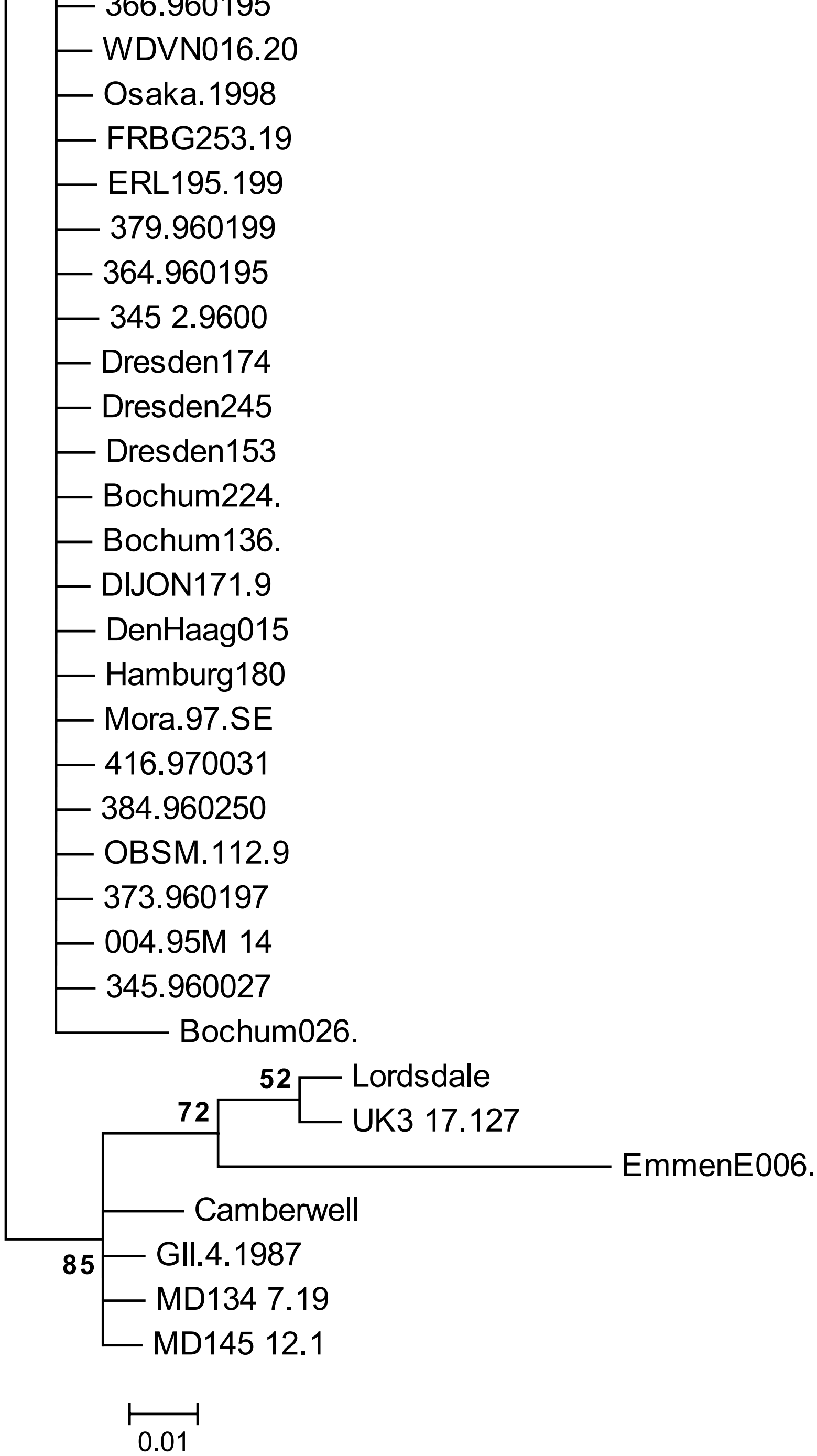
366.960195
WDVN016.20
Osaka.1998
FRBG253.19
ERL195.199
379.960199
364.960195
345 2.9600
Dresden174
Dresden245
Dresden153
Bochum224.
Bochum136.
DIJON171.9
DenHaag015
Hamburg180
Mora.97.SE
416.970031
384.960250
OBSM.112.9
373.960197
004.95M 14
345.960027
Bochum026.
52
Lordsdale
72
UK3 17.127
EmmenE006.
Camberwell
85
GII.4.1987
MD134 7.19
MD145 12.1
0.01
