# Supplementary material for: Mechanisms of GII.4 Norovirus Persistence in Human Populations
Source: PLoS Med. 2008 Feb 12;5(2):e31. doi: 10.1371/journal.pmed.0050031 (PMC2235898; doi:10.1371/journal.pmed.0050031)
Supplement: Supplementary file 9 [file pmed.0050031.sg009.pdf]

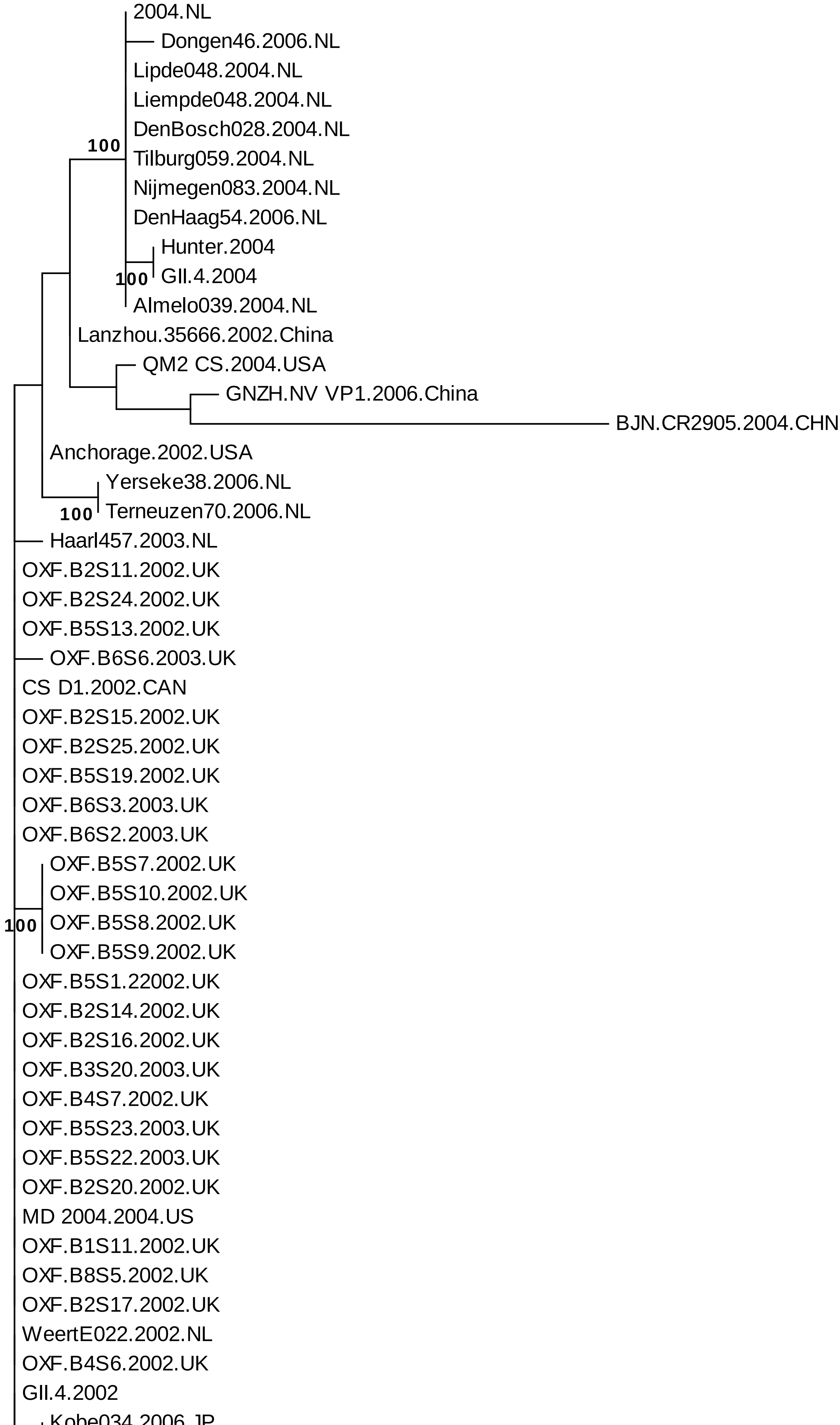

2004.NL
Dongen46.2006.NL
Lipde048.2004.NL
Liempde048.2004.NL
DenBosch028.2004.NL
Tilburg059.2004.NL
Nijmegen083.2004.NL
DenHaag54.2006.NL
Hunter.2004
GII.4.2004
Almelo039.2004.NL
Lanzhou.35666.2002.China
QM2 CS.2004.USA
GNZH.NV VP1.2006.China
BJN.CR2905.2004.CHN
Anchorage.2002.USA
Yerseke38.2006.NL
Terneuzen70.2006.NL
Haarl457.2003.NL
OXF.B2S11.2002.UK
OXF.B2S24.2002.UK
OXF.B5S13.2002.UK
OXF.B6S6.2003.UK
CS D1.2002.CAN
OXF.B2S15.2002.UK
OXF.B2S25.2002.UK
OXF.B5S19.2002.UK
OXF.B6S3.2003.UK
OXF.B6S2.2003.UK
OXF.B5S7.2002.UK
OXF.B5S10.2002.UK
OXF.B5S8.2002.UK
OXF.B5S9.2002.UK
OXF.B5S1.22002.UK
OXF.B2S14.2002.UK
OXF.B2S16.2002.UK
OXF.B3S20.2003.UK
OXF.B4S7.2002.UK
OXF.B5S23.2003.UK
OXF.B5S22.2003.UK
OXF.B2S20.2002.UK
MD 2004.2004.US
OXF.B1S11.2002.UK
OXF.B8S5.2002.UK
OXF.B2S17.2002.UK
WeertE022.2002.NL
OXF.B4S6.2002.UK
GII.4.2002
100
100
100
100

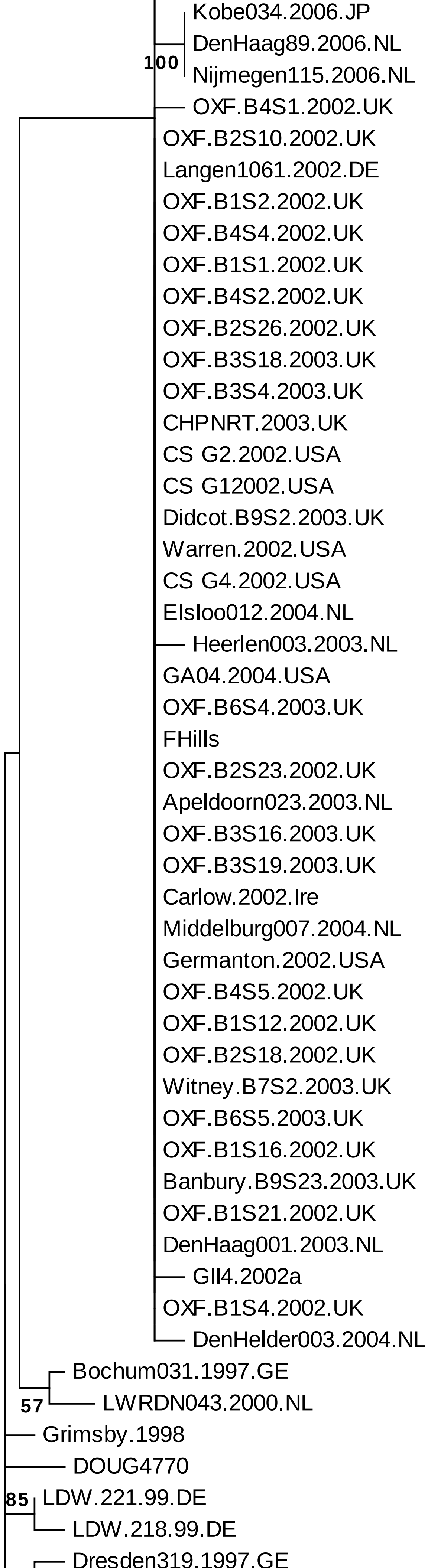
Kobe034.2006.JP
DenHaag89.2006.NL
100
Nijmegen115.2006.NL
OXF.B4S1.2002.UK
OXF.B2S10.2002.UK
Langen1061.2002.DE
OXF.B1S2.2002.UK
OXF.B4S4.2002.UK
OXF.B1S1.2002.UK
OXF.B4S2.2002.UK
OXF.B2S26.2002.UK
OXF.B3S18.2003.UK
OXF.B3S4.2003.UK
CHPNRT.2003.UK
CS G2.2002.USA
CS G12002.USA
Didcot.B9S2.2003.UK
Warren.2002.USA
CS G4.2002.USA
Elsloo012.2004.NL
Heerlen003.2003.NL
GA04.2004.USA
OXF.B6S4.2003.UK
FHills
OXF.B2S23.2002.UK
Apeldoorn023.2003.NL
OXF.B3S16.2003.UK
OXF.B3S19.2003.UK
Carlow.2002.Ire
Middelburg007.2004.NL
Germanton.2002.USA
OXF.B4S5.2002.UK
OXF.B1S12.2002.UK
OXF.B2S18.2002.UK
Witney.B7S2.2003.UK
OXF.B6S5.2003.UK
OXF.B1S16.2002.UK
Banbury.B9S23.2003.UK
OXF.B1S21.2002.UK
DenHaag001.2003.NL
GII4.2002a
OXF.B1S4.2002.UK
DenHelder003.2004.NL
Bochum031.1997.GE
57
LWRDN043.2000.NL
Grimsby.1998
DOUG4770
85
LDW.221.99.DE
LDW.218.99.DE
Dresden319.1997.GE

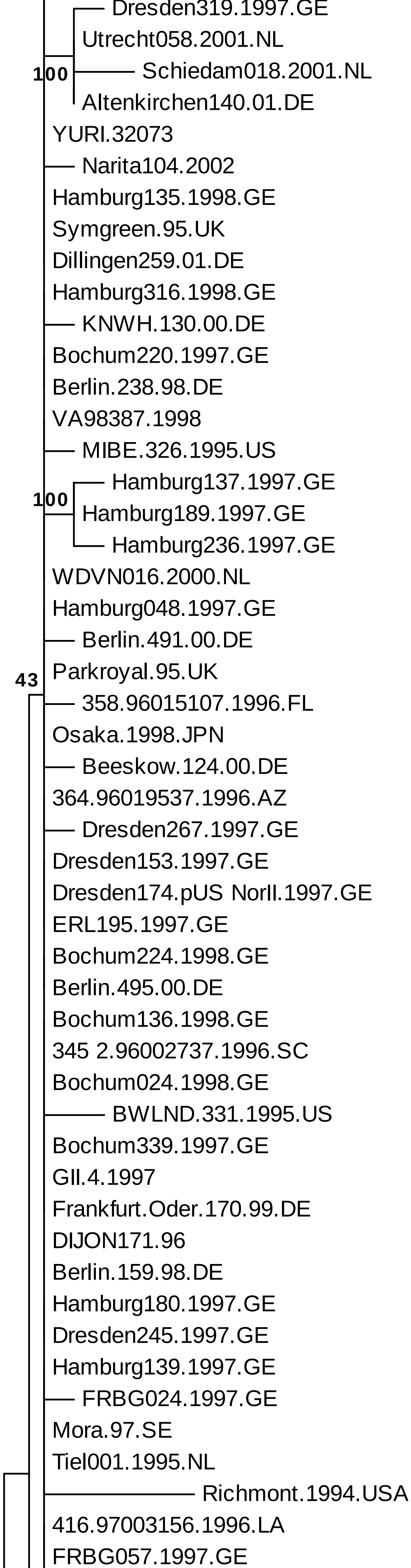
Dresden319.1997.GE
Utrecht058.2001.NL
100
Schiedam018.2001.NL
Altenkirchen140.01.DE
YURI.32073
Narita104.2002
Hamburg135.1998.GE
Symgreen.95.UK
Dillingen259.01.DE
Hamburg316.1998.GE
KNWH.130.00.DE
Bochum220.1997.GE
Berlin.238.98.DE
VA98387.1998
MIBE.326.1995.US
Hamburg137.1997.GE
100
Hamburg189.1997.GE
Hamburg236.1997.GE
WDVN016.2000.NL
Hamburg048.1997.GE
Berlin.491.00.DE
43
Parkroyal.95.UK
358.96015107.1996.FL
Osaka.1998.JPN
Beeskow.124.00.DE
364.96019537.1996.AZ
Dresden267.1997.GE
Dresden153.1997.GE
Dresden174.pUS NorII.1997.GE
ERL195.1997.GE
Bochum224.1998.GE
Berlin.495.00.DE
Bochum136.1998.GE
345 2.96002737.1996.SC
Bochum024.1998.GE
BWLND.331.1995.US
Bochum339.1997.GE
GII.4.1997
Frankfurt.Oder.170.99.DE
DIJON171.96
Berlin.159.98.DE
Hamburg180.1997.GE
Dresden245.1997.GE
Hamburg139.1997.GE
FRBG024.1997.GE
Mora.97.SE
Tiel001.1995.NL
Richmont.1994.USA
416.97003156.1996.LA
FRBG057.1997.GE

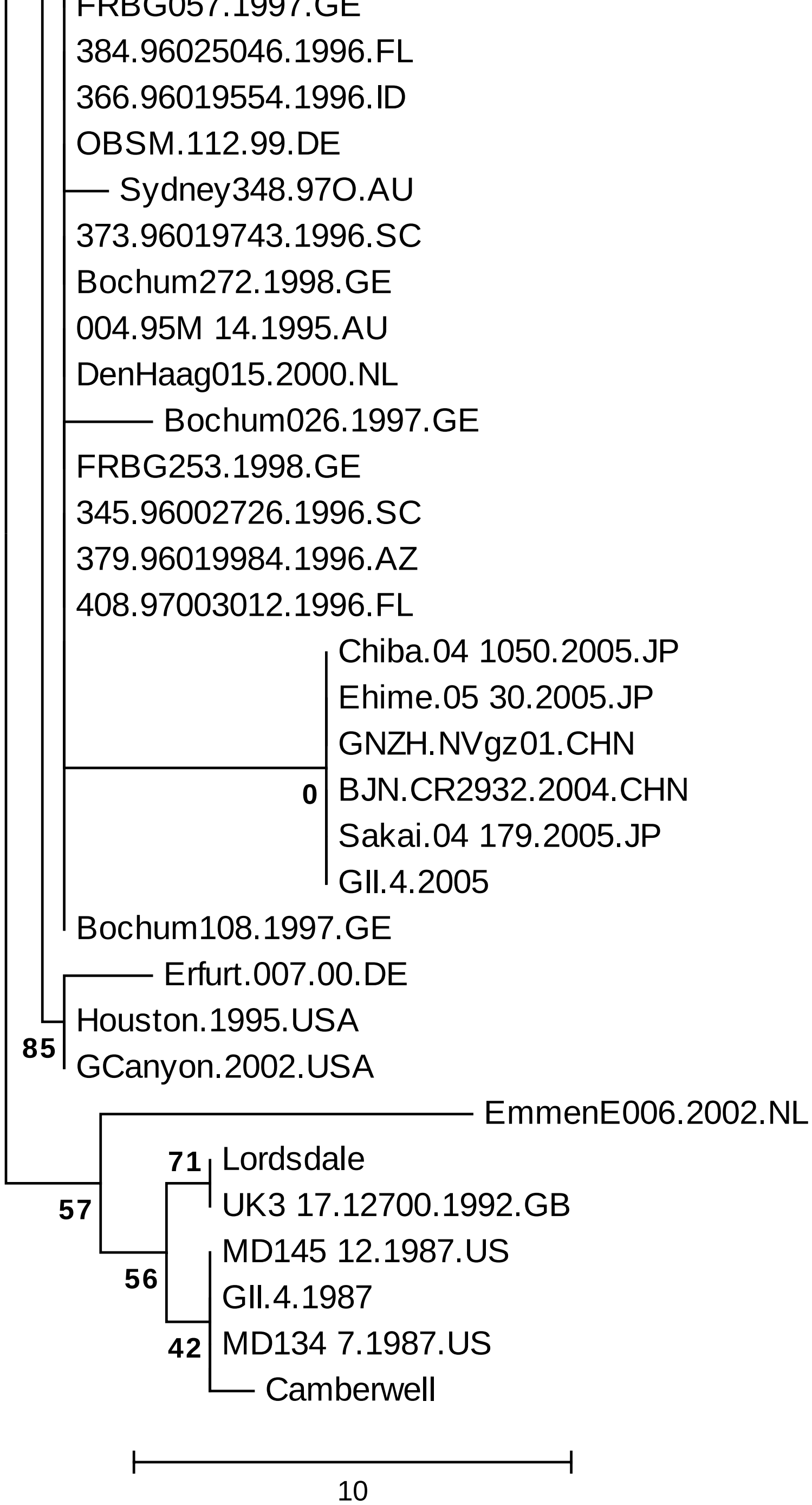
FRBG057.1997.GE
384.96025046.1996.FL
366.96019554.1996.ID
OBSM.112.99.DE
Sydney348.97O.AU
373.96019743.1996.SC
Bochum272.1998.GE
004.95M 14.1995.AU
DenHaag015.2000.NL
Bochum026.1997.GE
FRBG253.1998.GE
345.96002726.1996.SC
379.96019984.1996.AZ
408.97003012.1996.FL
Chiba.04 1050.2005.JP
Ehime.05 30.2005.JP
GNZH.NVgz01.CHN
0
BJN.CR2932.2004.CHN
Sakai.04 179.2005.JP
GII.4.2005
Bochum108.1997.GE
Erfurt.007.00.DE
Houston.1995.USA
85
GCanyon.2002.USA
EmmenE006.2002.NL
71
Lordsdale
57
UK3 17.12700.1992.GB
MD145 12.1987.US
56
GII.4.1987
42
MD134 7.1987.US
Camberwell
10
