# Supplementary material for: Mechanisms of GII.4 Norovirus Persistence in Human Populations
Source: PLoS Med. 2008 Feb 12;5(2):e31. doi: 10.1371/journal.pmed.0050031 (PMC2235898; doi:10.1371/journal.pmed.0050031)
Supplement: Supplementary file 10 [file pmed.0050031.sg010.pdf]

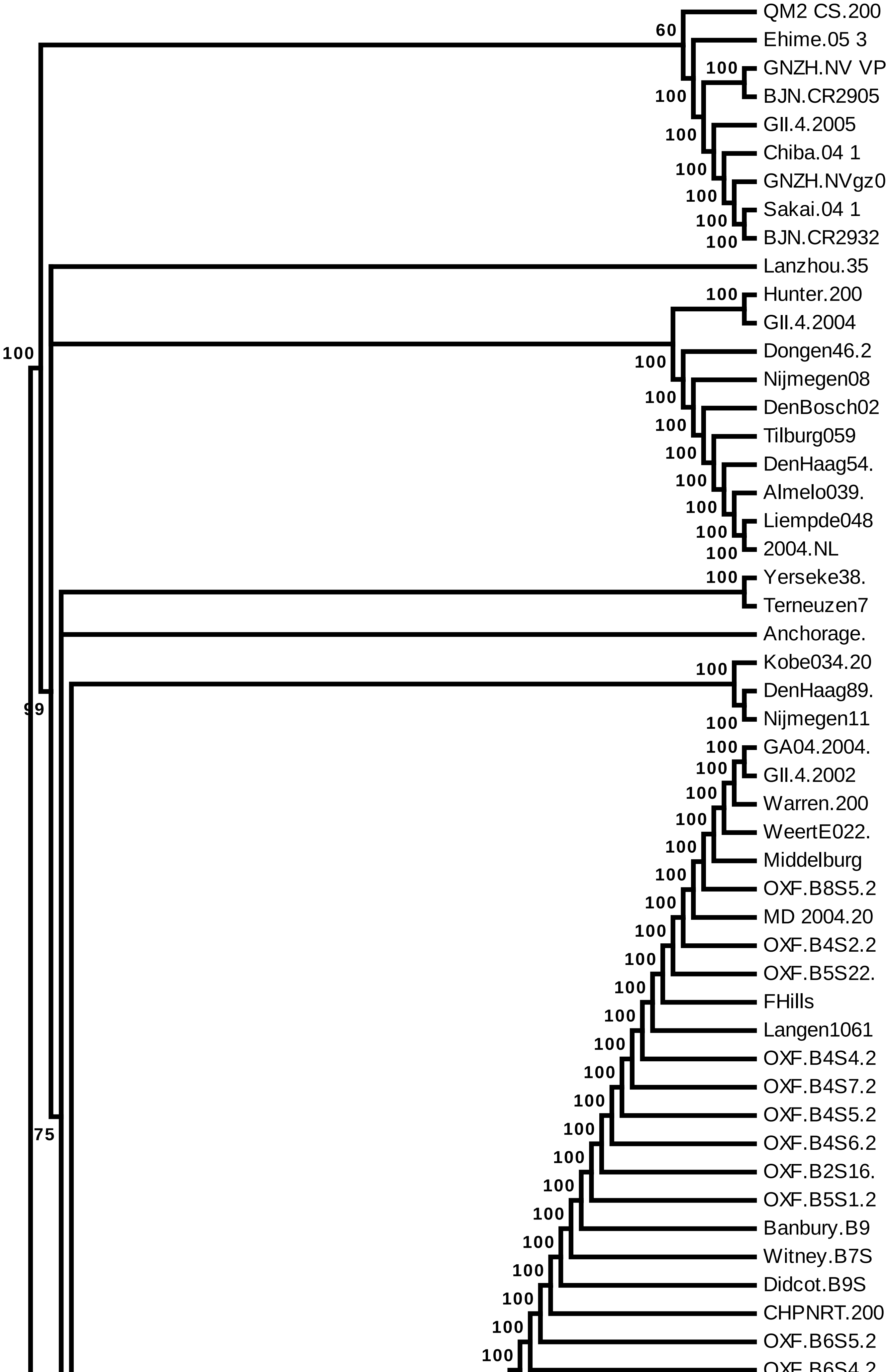

QM2 CS.200
Ehime.05 3
GNZH.NV VP
BJN.CR2905
GII.4.2005
Chiba.04 1
GNZH.NVgz0
Sakai.04 1
BJN.CR2932
Lanzhou.35
Hunter.200
GII.4.2004
Dongen46.2
Nijmegen08
DenBosch02
Tilburg059
DenHaag54.
Almelo039.
Liempde048
2004.NL
Yerseke38.
Terneuzen7
Anchorage.
Kobe034.20
DenHaag89.
Nijmegen11
GA04.2004.
GII.4.2002
Warren.200
WeertE022.
Middelburg
OXF.B8S5.2
MD 2004.20
OXF.B4S2.2
OXF.B5S22.
FHills
Langen1061
OXF.B4S4.2
OXF.B4S7.2
OXF.B4S5.2
OXF.B4S6.2
OXF.B2S16.
OXF.B5S1.2
Banbury.B9
Witney.B7S
Didcot.B9S
CHPNRT.200
OXF.B6S5.2
OXF.B6S4.2

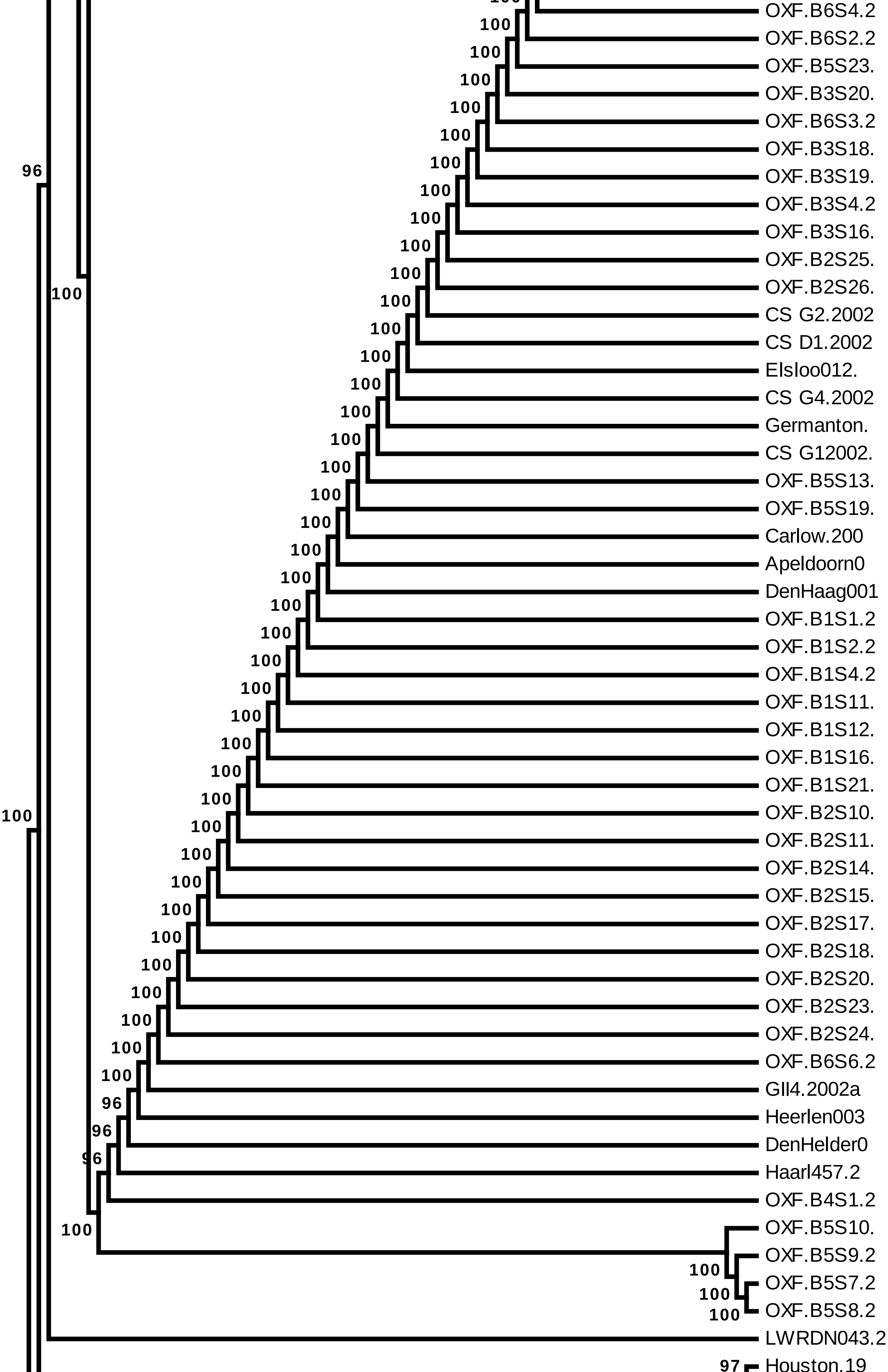
OXF.B6S4.2
OXF.B6S2.2
OXF.B5S23.
OXF.B3S20.
OXF.B6S3.2
OXF.B3S18.
OXF.B3S19.
OXF.B3S4.2
OXF.B3S16.
OXF.B2S25.
OXF.B2S26.
CS G2.2002
CS D1.2002
Elsloo012.
CS G4.2002
Germanton.
CS G12002.
OXF.B5S13.
OXF.B5S19.
Carlow.200
Apeldoorn0
DenHaag001
OXF.B1S1.2
OXF.B1S2.2
OXF.B1S4.2
OXF.B1S11.
OXF.B1S12.
OXF.B1S16.
OXF.B1S21.
OXF.B2S10.
OXF.B2S11.
OXF.B2S14.
OXF.B2S15.
OXF.B2S17.
OXF.B2S18.
OXF.B2S20.
OXF.B2S23.
OXF.B2S24.
OXF.B6S6.2
GII4.2002a
Heerlen003
DenHelder0
Haarl457.2
OXF.B4S1.2
OXF.B5S10.
OXF.B5S9.2
OXF.B5S7.2
OXF.B5S8.2
LWRDN043.2
Houston.19
96
100
100
96
96
96
97

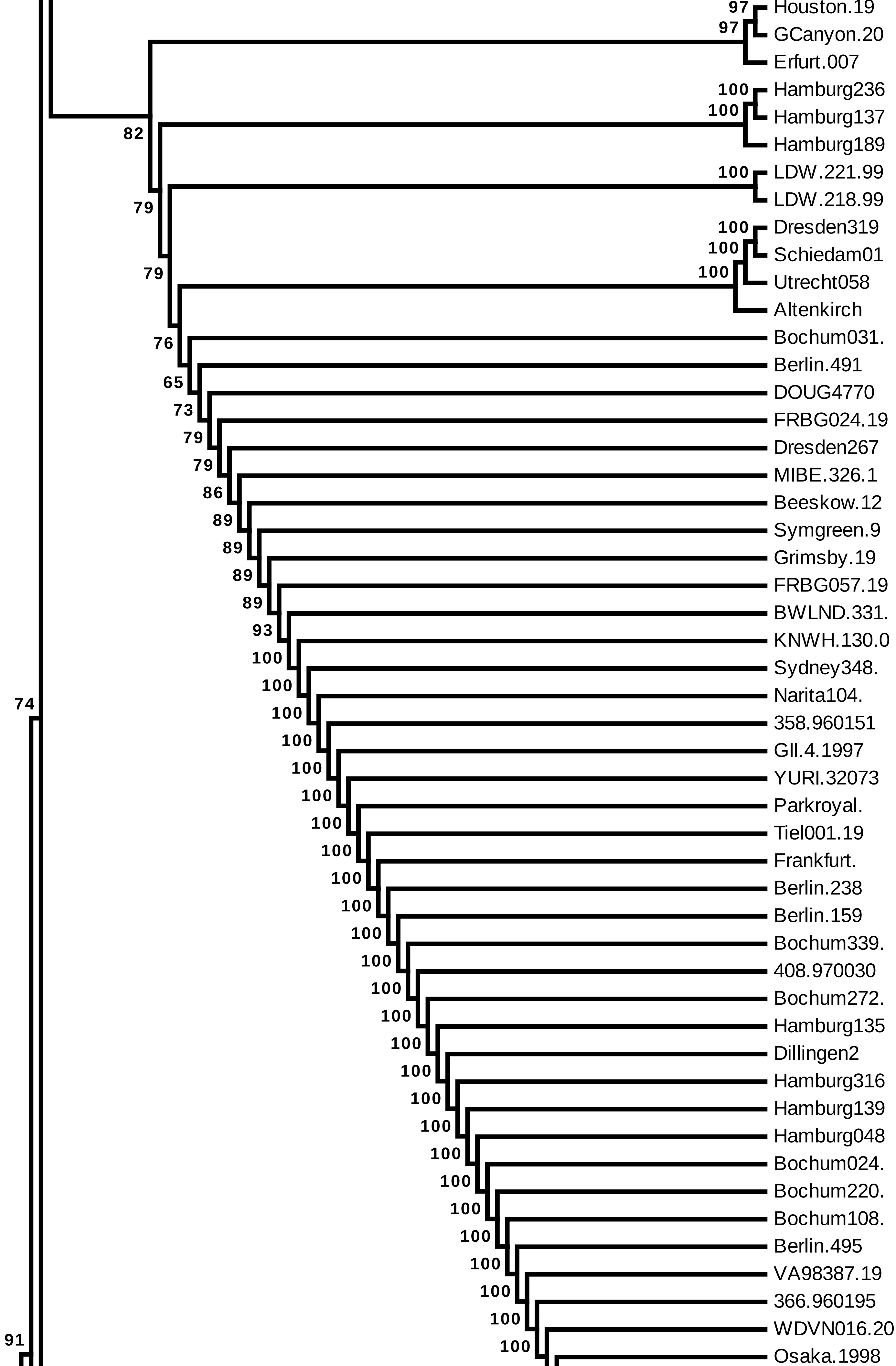

Houston.19
GCanyon.20
Erfurt.007
Hamburg236
Hamburg137
Hamburg189
LDW.221.99
LDW.218.99
Dresden319
Schiedam01
Utrecht058
Altenkirch
Bochum031.
Berlin.491
DOUG4770
FRBG024.19
Dresden267
MIBE.326.1
Beeskow.12
Symgreen.9
Grimsby.19
FRBG057.19
BWLND.331.
KNWH.130.0
Sydney348.
Narita104.
358.960151
GII.4.1997
YURI.32073
Parkroyal.
Tiel001.19
Frankfurt.
Berlin.238
Berlin.159
Bochum339.
408.970030
Bochum272.
Hamburg135
Dillingen2
Hamburg316
Hamburg139
Hamburg048
Bochum024.
Bochum220.
Bochum108.
Berlin.495
VA98387.19
366.960195
WDVN016.20
Osaka.1998
97
97
100
100
100
100
100
100
82
79
79
76
65
73
79
79
86
89
89
89
89
93
100
74
91

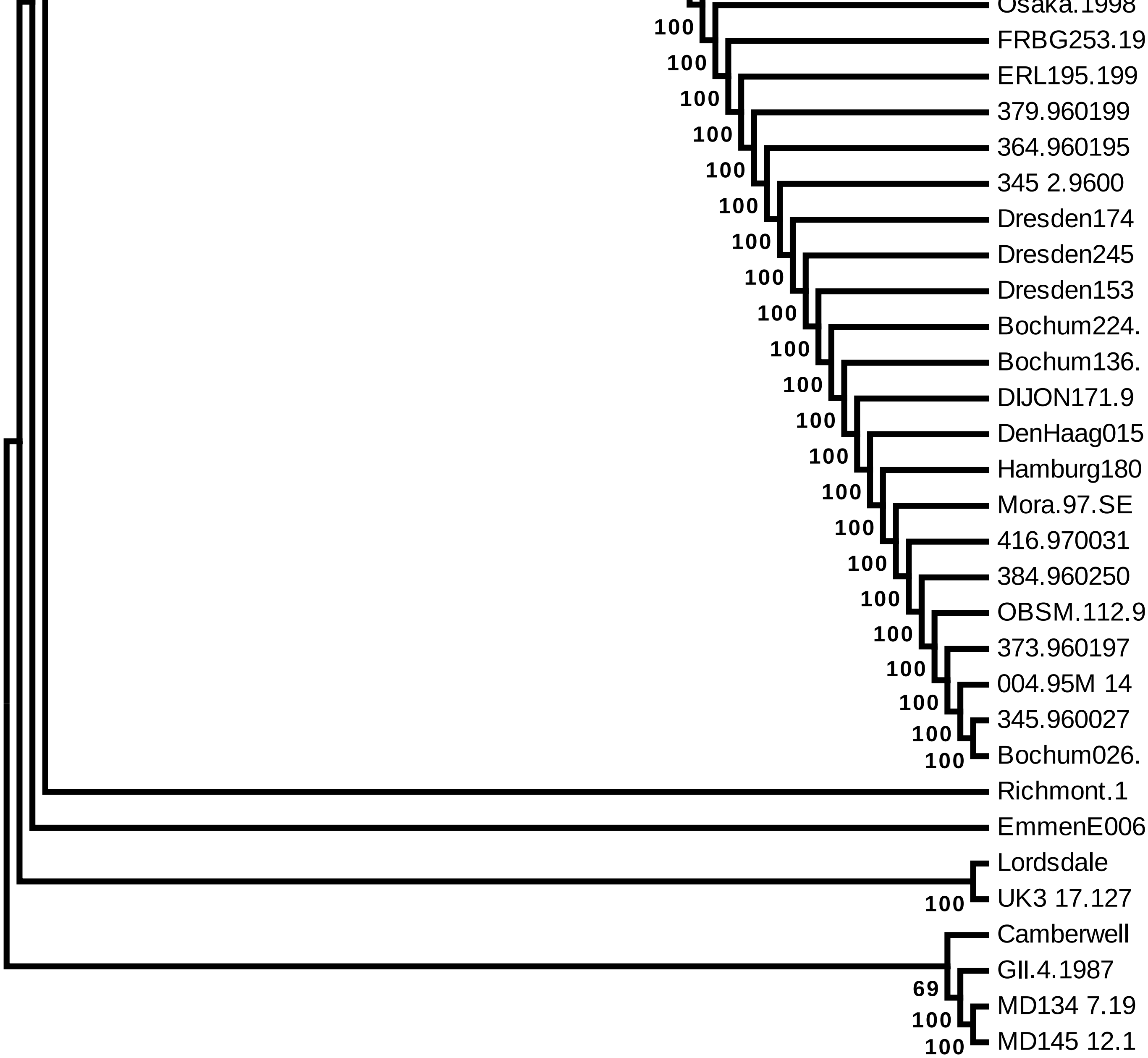
Osaka.1998
FRBG253.19
ERL195.199
379.960199
364.960195
345 2.9600
Dresden174
Dresden245
Dresden153
Bochum224.
Bochum136.
DIJON171.9
DenHaag015
Hamburg180
Mora.97.SE
416.970031
384.960250
OBSM.112.9
373.960197
004.95M 14
345.960027
Bochum026.
Richmont.1
EmmenE006.
Lordsdale
UK3 17.127
Camberwell
GII.4.1987
MD134 7.19
MD145 12.1
100
69
