# Supplementary material for: Mechanisms of GII.4 Norovirus Persistence in Human Populations
Source: PLoS Med. 2008 Feb 12;5(2):e31. doi: 10.1371/journal.pmed.0050031 (PMC2235898; doi:10.1371/journal.pmed.0050031)
Supplement: Supplementary file 11 [file pmed.0050031.sg011.pdf]

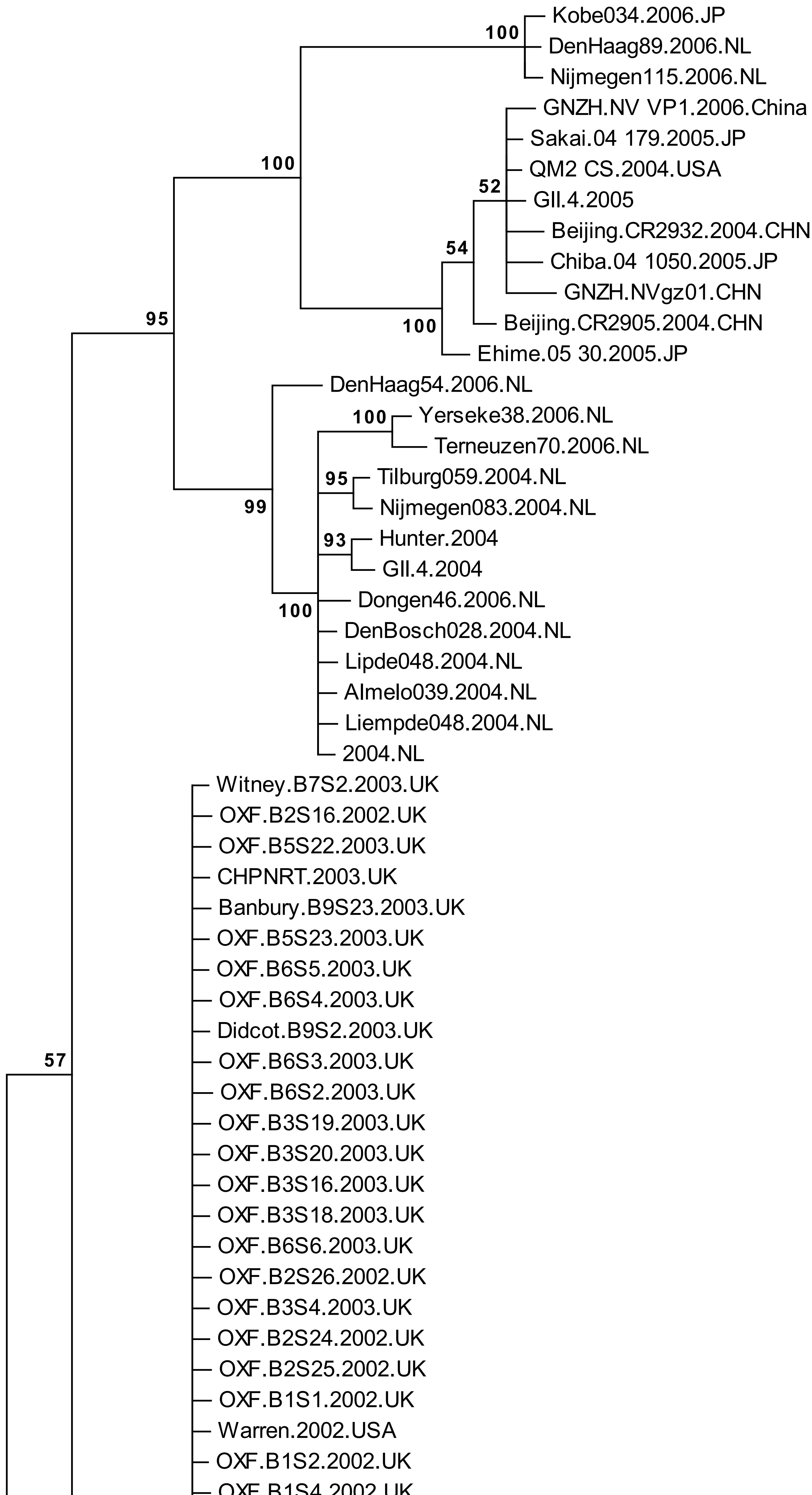
Kobe034.2006.JP
DenHaag89.2006.NL
Nijmegen115.2006.NL
100
GNZH.NV VP1.2006.China
Sakai.04 179.2005.JP
QM2 CS.2004.USA
GII.4.2005
Beijing.CR2932.2004.CHN
Chiba.04 1050.2005.JP
GNZH.NVgz01.CHN
Beijing.CR2905.2004.CHN
Ehime.05 30.2005.JP
100
52
54
100
95
DenHaag54.2006.NL
Yerseke38.2006.NL
Terneuzen70.2006.NL
Tilburg059.2004.NL
Nijmegen083.2004.NL
Hunter.2004
GII.4.2004
Dongen46.2006.NL
DenBosch028.2004.NL
Lipde048.2004.NL
Almelo039.2004.NL
Liempde048.2004.NL
2004.NL
100
95
93
99
100
Witney.B7S2.2003.UK
OXF.B2S16.2002.UK
OXF.B5S22.2003.UK
CHPNRT.2003.UK
Banbury.B9S23.2003.UK
OXF.B5S23.2003.UK
OXF.B6S5.2003.UK
OXF.B6S4.2003.UK
Didcot.B9S2.2003.UK
OXF.B6S3.2003.UK
OXF.B6S2.2003.UK
OXF.B3S19.2003.UK
OXF.B3S20.2003.UK
OXF.B3S16.2003.UK
OXF.B3S18.2003.UK
OXF.B6S6.2003.UK
OXF.B2S26.2002.UK
OXF.B3S4.2003.UK
OXF.B2S24.2002.UK
OXF.B2S25.2002.UK
OXF.B1S1.2002.UK
Warren.2002.USA
OXF.B1S2.2002.UK
OXF.B1S4.2002.UK
57

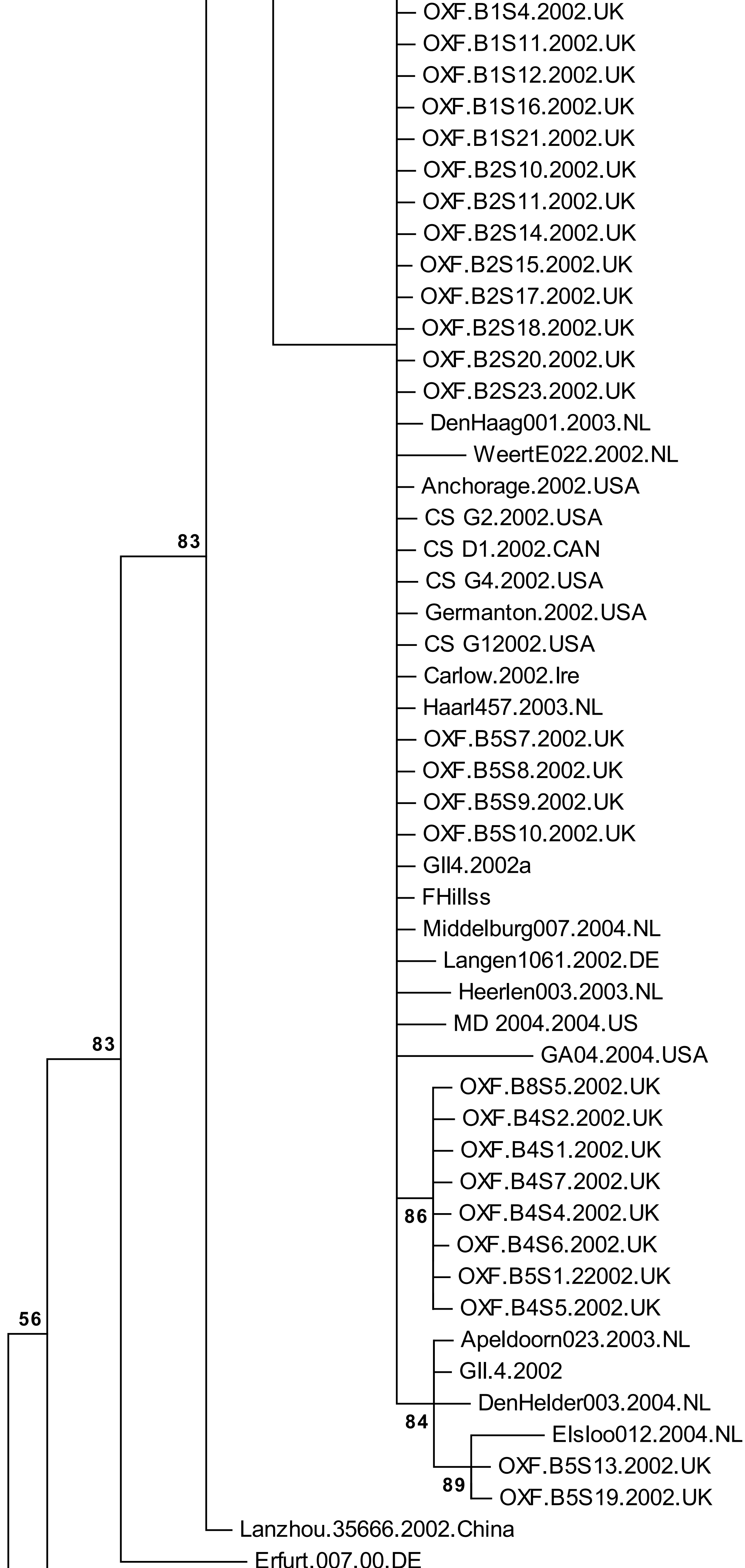

OXF.B1S4.2002.UK
OXF.B1S11.2002.UK
OXF.B1S12.2002.UK
OXF.B1S16.2002.UK
OXF.B1S21.2002.UK
OXF.B2S10.2002.UK
OXF.B2S11.2002.UK
OXF.B2S14.2002.UK
OXF.B2S15.2002.UK
OXF.B2S17.2002.UK
OXF.B2S18.2002.UK
OXF.B2S20.2002.UK
OXF.B2S23.2002.UK
DenHaag001.2003.NL
WeertE022.2002.NL
Anchorage.2002.USA
CS G2.2002.USA
83
CS D1.2002.CAN
CS G4.2002.USA
Germanton.2002.USA
CS G12002.USA
Carlow.2002.Ire
Haarl457.2003.NL
OXF.B5S7.2002.UK
OXF.B5S8.2002.UK
OXF.B5S9.2002.UK
OXF.B5S10.2002.UK
GII4.2002a
FHillss
Middelburg007.2004.NL
Langen1061.2002.DE
Heerlen003.2003.NL
MD 2004.2004.US
83
GA04.2004.USA
OXF.B8S5.2002.UK
OXF.B4S2.2002.UK
OXF.B4S1.2002.UK
OXF.B4S7.2002.UK
86
OXF.B4S4.2002.UK
OXF.B4S6.2002.UK
OXF.B5S1.22002.UK
56
OXF.B4S5.2002.UK
Apeldoorn023.2003.NL
GII.4.2002
DenHelder003.2004.NL
84
Elsloo012.2004.NL
OXF.B5S13.2002.UK
89
OXF.B5S19.2002.UK
Lanzhou.35666.2002.China
Erfurt.007.00.DE

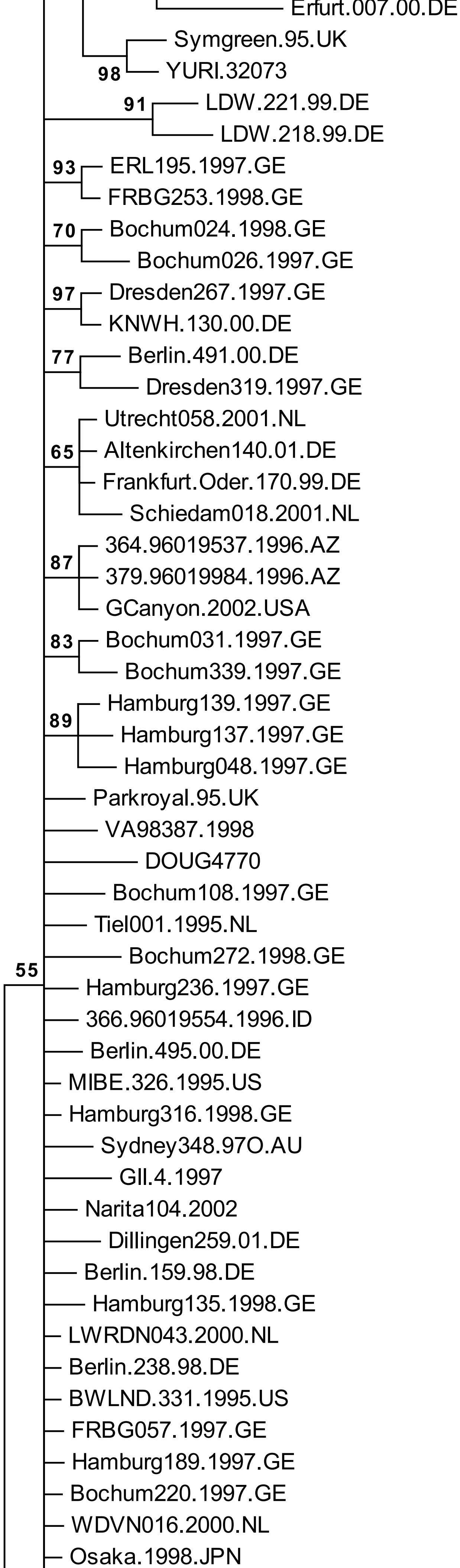

Erfurt.007.00.DE
Symgreen.95.UK
98
YURI.32073
91
LDW.221.99.DE
LDW.218.99.DE
93
ERL195.1997.GE
FRBG253.1998.GE
70
Bochum024.1998.GE
Bochum026.1997.GE
97
Dresden267.1997.GE
KNWH.130.00.DE
77
Berlin.491.00.DE
Dresden319.1997.GE
Utrecht058.2001.NL
65
Altenkirchen140.01.DE
Frankfurt.Oder.170.99.DE
Schiedam018.2001.NL
87
364.96019537.1996.AZ
379.96019984.1996.AZ
GCanyon.2002.USA
83
Bochum031.1997.GE
Bochum339.1997.GE
89
Hamburg139.1997.GE
Hamburg137.1997.GE
Hamburg048.1997.GE
Parkroyal.95.UK
VA98387.1998
DOUG4770
Bochum108.1997.GE
Tiel001.1995.NL
Bochum272.1998.GE
55
Hamburg236.1997.GE
366.96019554.1996.ID
Berlin.495.00.DE
MIBE.326.1995.US
Hamburg316.1998.GE
Sydney348.97O.AU
GII.4.1997
Narita104.2002
Dillingen259.01.DE
Berlin.159.98.DE
Hamburg135.1998.GE
LWRDN043.2000.NL
Berlin.238.98.DE
BWLND.331.1995.US
FRBG057.1997.GE
Hamburg189.1997.GE
Bochum220.1997.GE
WDVN016.2000.NL
Osaka.1998.JPN

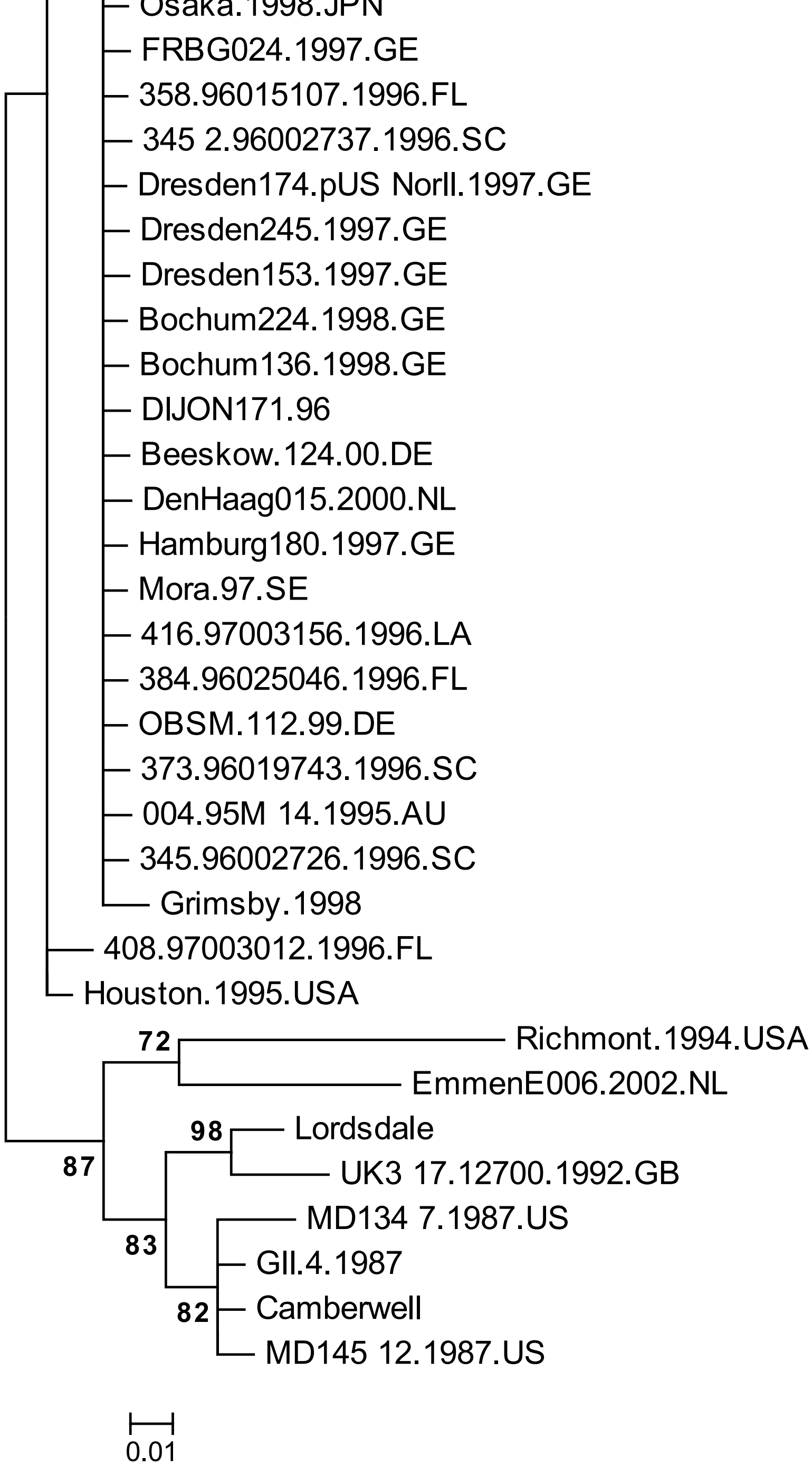
Osaka.1998.JPN
FRBG024.1997.GE
358.96015107.1996.FL
345 2.96002737.1996.SC
Dresden174.pUS NorII.1997.GE
Dresden245.1997.GE
Dresden153.1997.GE
Bochum224.1998.GE
Bochum136.1998.GE
DIJON171.96
Beeskow.124.00.DE
DenHaag015.2000.NL
Hamburg180.1997.GE
Mora.97.SE
416.97003156.1996.LA
384.96025046.1996.FL
OBSM.112.99.DE
373.96019743.1996.SC
004.95M 14.1995.AU
345.96002726.1996.SC
Grimsby.1998
408.97003012.1996.FL
Houston.1995.USA
72
Richmont.1994.USA
EmmenE006.2002.NL
98
Lordsdale
87
UK3 17.12700.1992.GB
MD134 7.1987.US
83
GII.4.1987
Camberwell
82
MD145 12.1987.US
0.01
