# Supplementary material for: Mechanisms of GII.4 Norovirus Persistence in Human Populations
Source: PLoS Med. 2008 Feb 12;5(2):e31. doi: 10.1371/journal.pmed.0050031 (PMC2235898; doi:10.1371/journal.pmed.0050031)
Supplement: Supplementary file 12 [file pmed.0050031.sg012.pdf]

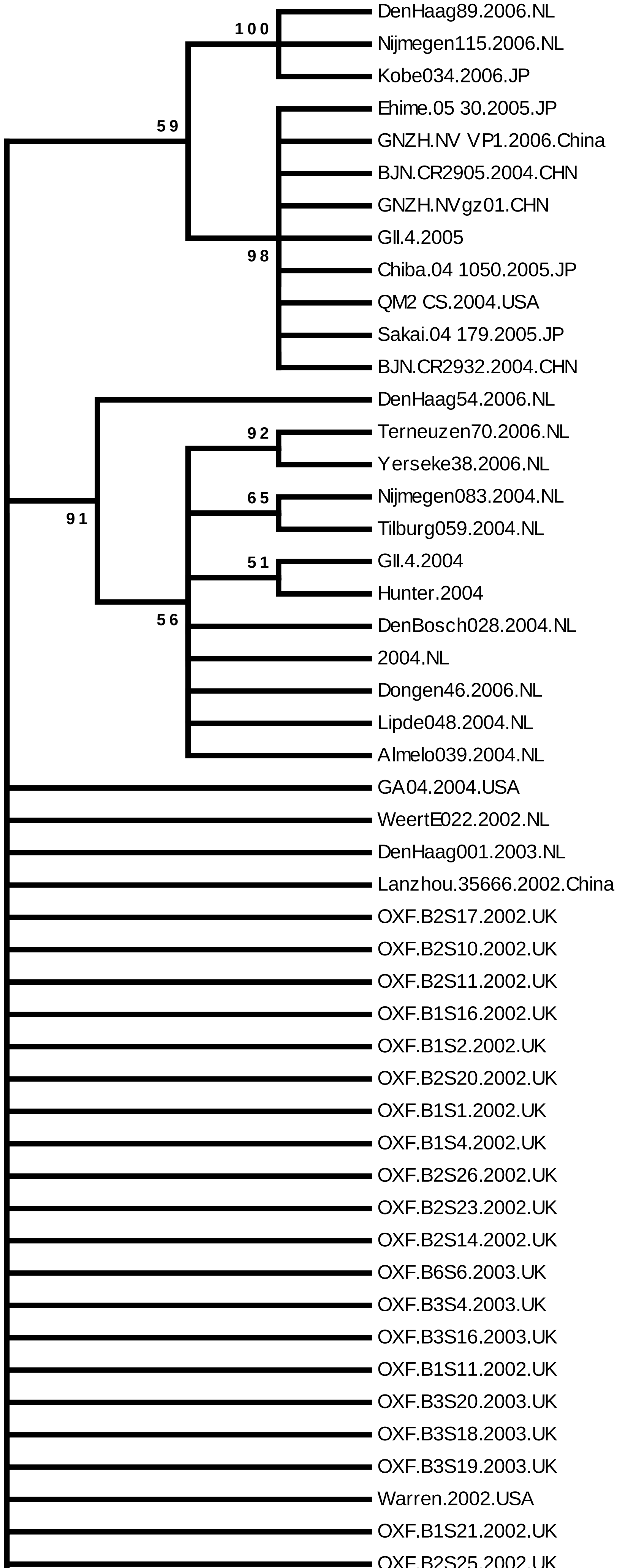
100
59
98
92
65
91
51
56
DenHaag89.2006.NL
Nijmegen115.2006.NL
Kobe034.2006.JP
Ehime.05 30.2005.JP
GNZH.NV VP1.2006.China
BJN.CR2905.2004.CHN
GNZH.NVgz01.CHN
GII.4.2005
Chiba.04 1050.2005.JP
QM2 CS.2004.USA
Sakai.04 179.2005.JP
BJN.CR2932.2004.CHN
DenHaag54.2006.NL
Terneuzen70.2006.NL
Yerseke38.2006.NL
Nijmegen083.2004.NL
Tilburg059.2004.NL
GII.4.2004
Hunter.2004
DenBosch028.2004.NL
2004.NL
Dongen46.2006.NL
Lipde048.2004.NL
Almelo039.2004.NL
GA04.2004.USA
WeertE022.2002.NL
DenHaag001.2003.NL
Lanzhou.35666.2002.China
OXF.B2S17.2002.UK
OXF.B2S10.2002.UK
OXF.B2S11.2002.UK
OXF.B1S16.2002.UK
OXF.B1S2.2002.UK
OXF.B2S20.2002.UK
OXF.B1S1.2002.UK
OXF.B1S4.2002.UK
OXF.B2S26.2002.UK
OXF.B2S23.2002.UK
OXF.B2S14.2002.UK
OXF.B6S6.2003.UK
OXF.B3S4.2003.UK
OXF.B3S16.2003.UK
OXF.B1S11.2002.UK
OXF.B3S20.2003.UK
OXF.B3S18.2003.UK
OXF.B3S19.2003.UK
Warren.2002.USA
OXF.B1S21.2002.UK
OXF.B2S25.2002.UK

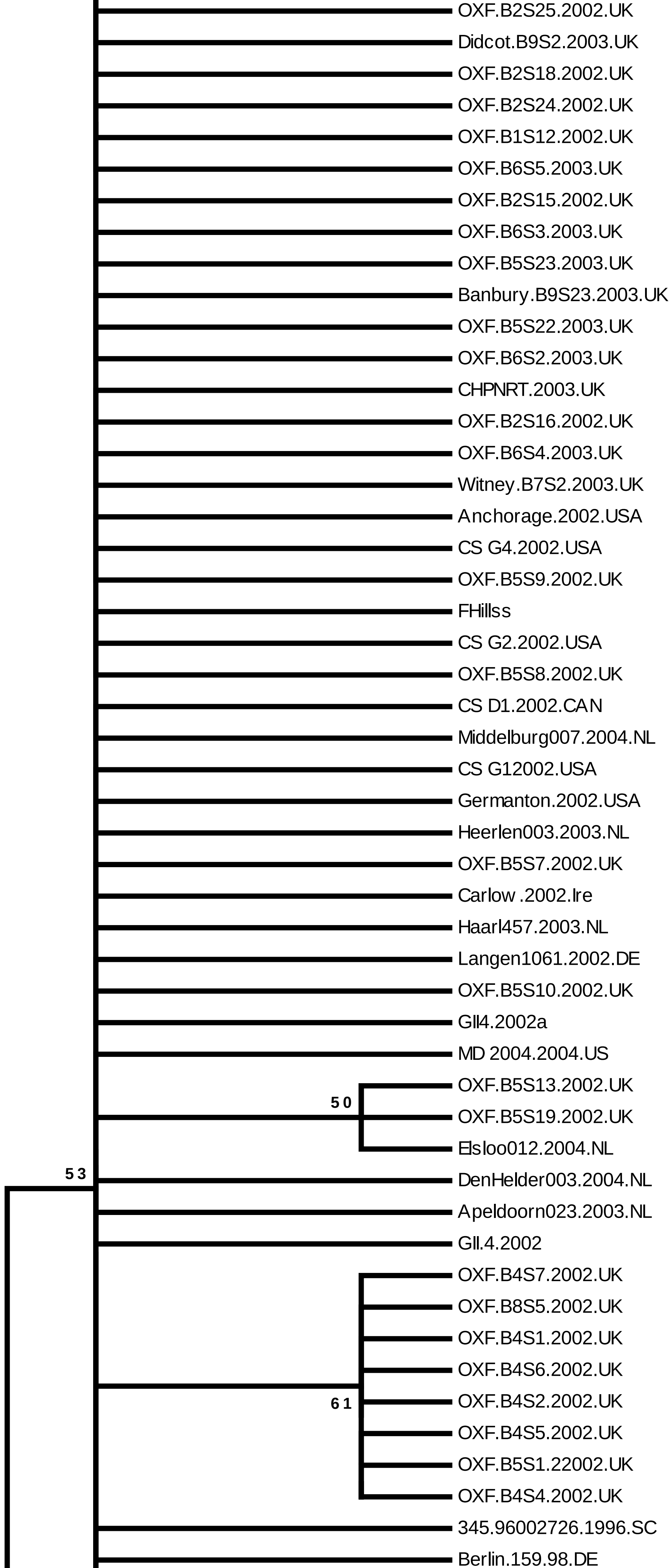
OXF.B2S25.2002.UK
Didcot.B9S2.2003.UK
OXF.B2S18.2002.UK
OXF.B2S24.2002.UK
OXF.B1S12.2002.UK
OXF.B6S5.2003.UK
OXF.B2S15.2002.UK
OXF.B6S3.2003.UK
OXF.B5S23.2003.UK
Banbury.B9S23.2003.UK
OXF.B5S22.2003.UK
OXF.B6S2.2003.UK
CHPNRT.2003.UK
OXF.B2S16.2002.UK
OXF.B6S4.2003.UK
Witney.B7S2.2003.UK
Anchorage.2002.USA
CS G4.2002.USA
OXF.B5S9.2002.UK
FHillss
CS G2.2002.USA
OXF.B5S8.2002.UK
CS D1.2002.CAN
Middelburg007.2004.NL
CS G12002.USA
Germanton.2002.USA
Heerlen003.2003.NL
OXF.B5S7.2002.UK
Carlow .2002.Ire
Haarl457.2003.NL
Langen1061.2002.DE
OXF.B5S10.2002.UK
GII4.2002a
MD 2004.2004.US
50
OXF.B5S13.2002.UK
OXF.B5S19.2002.UK
Elsloo012.2004.NL
53
DenHelder003.2004.NL
Apeldoorn023.2003.NL
GII.4.2002
OXF.B4S7.2002.UK
OXF.B8S5.2002.UK
OXF.B4S1.2002.UK
OXF.B4S6.2002.UK
61
OXF.B4S2.2002.UK
OXF.B4S5.2002.UK
OXF.B5S1.22002.UK
OXF.B4S4.2002.UK
345.96002726.1996.SC
Berlin.159.98.DE

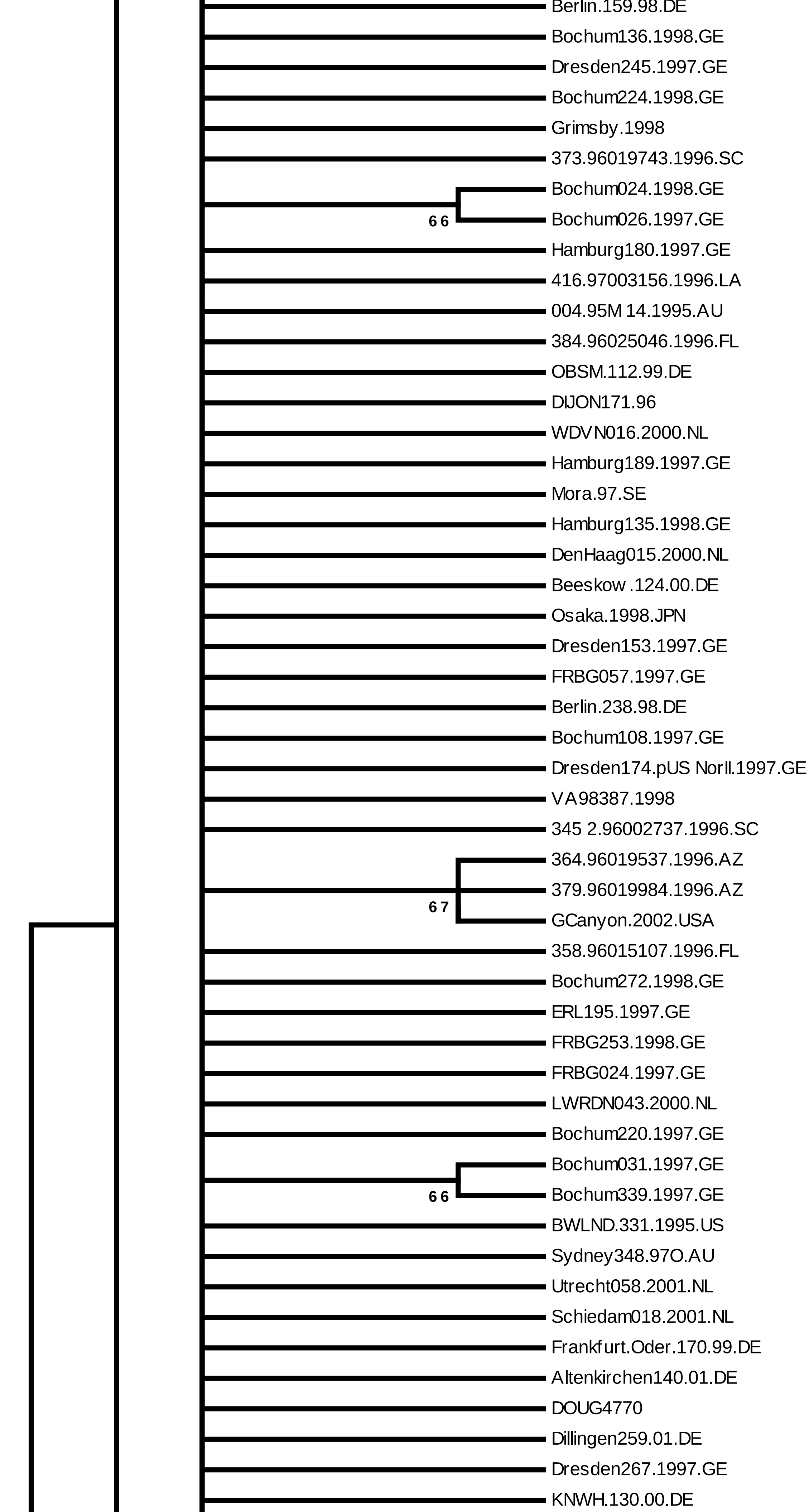
Berlin.159.98.DE
Bochum136.1998.GE
Dresden245.1997.GE
Bochum224.1998.GE
Grimsby.1998
373.96019743.1996.SC
Bochum024.1998.GE
66
Bochum026.1997.GE
Hamburg180.1997.GE
416.97003156.1996.LA
004.95M 14.1995.AU
384.96025046.1996.FL
OBSM.112.99.DE
DIJON171.96
WDVN016.2000.NL
Hamburg189.1997.GE
Mora.97.SE
Hamburg135.1998.GE
DenHaag015.2000.NL
Beeskow .124.00.DE
Osaka.1998.JPN
Dresden153.1997.GE
FRBG057.1997.GE
Berlin.238.98.DE
Bochum108.1997.GE
Dresden174.pUS NorII.1997.GE
VA98387.1998
345 2.96002737.1996.SC
364.96019537.1996.AZ
379.96019984.1996.AZ
67
GCanyon.2002.USA
358.96015107.1996.FL
Bochum272.1998.GE
ERL195.1997.GE
FRBG253.1998.GE
FRBG024.1997.GE
LWRDN043.2000.NL
Bochum220.1997.GE
Bochum031.1997.GE
66
Bochum339.1997.GE
BWLND.331.1995.US
Sydney348.97O.AU
Utrecht058.2001.NL
Schiedam018.2001.NL
Frankfurt.Oder.170.99.DE
Altenkirchen140.01.DE
DOUG4770
Dillingen259.01.DE
Dresden267.1997.GE
KNWH.130.00.DE

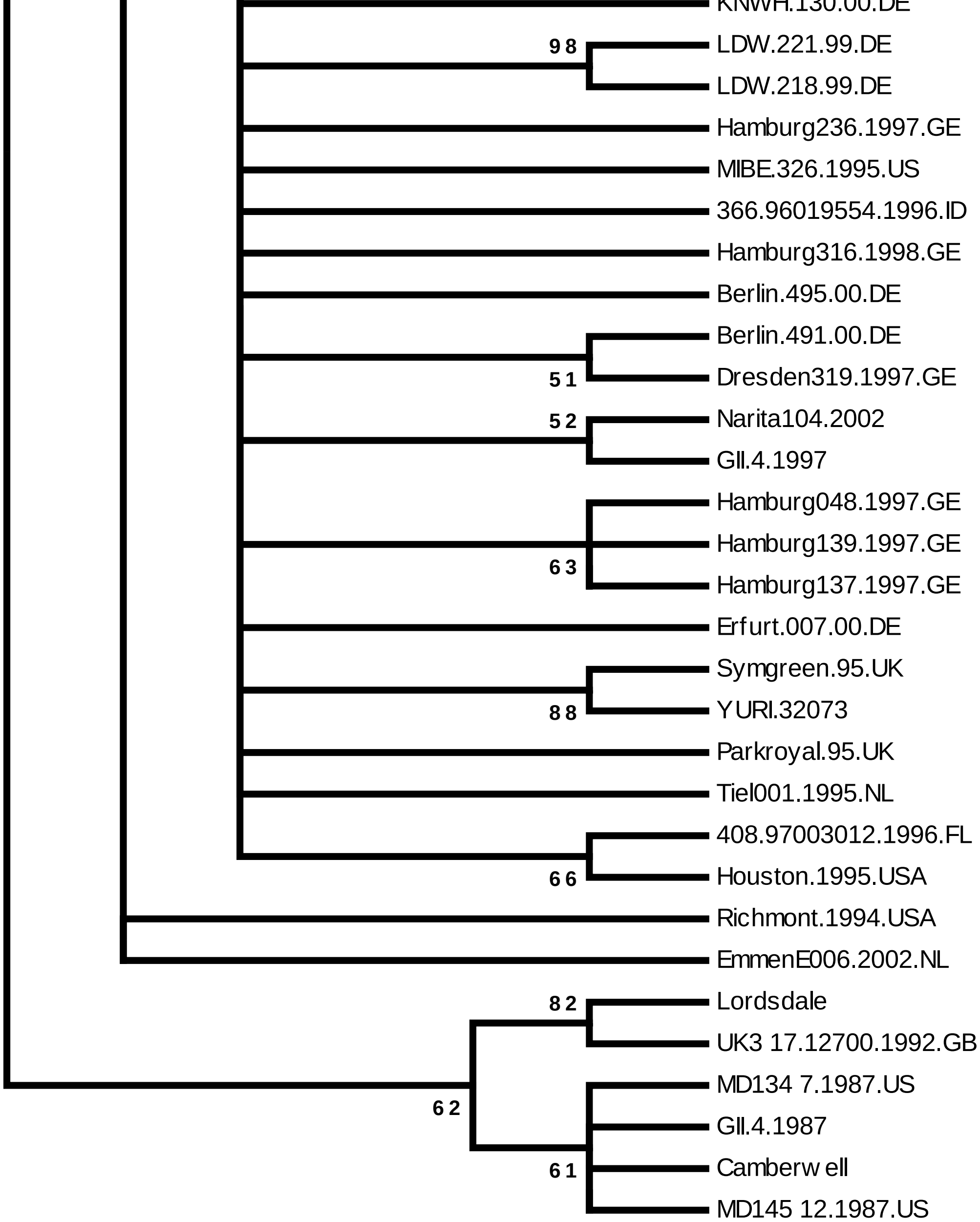
KNWH.130.00.DE
98
LDW.221.99.DE
LDW.218.99.DE
Hamburg236.1997.GE
MIBE.326.1995.US
366.96019554.1996.ID
Hamburg316.1998.GE
Berlin.495.00.DE
Berlin.491.00.DE
51
Dresden319.1997.GE
52
Narita104.2002
GII.4.1997
Hamburg048.1997.GE
Hamburg139.1997.GE
63
Hamburg137.1997.GE
Erfurt.007.00.DE
Symgreen.95.UK
88
YURI.32073
Parkroyal.95.UK
Tiel001.1995.NL
408.97003012.1996.FL
66
Houston.1995.USA
Richmont.1994.USA
EmmenE006.2002.NL
82
Lordsdale
UK3 17.12700.1992.GB
62
MD134 7.1987.US
GII.4.1987
61
Camberw ell
MD145 12.1987.US
