# Supplementary material for: Mechanisms of GII.4 Norovirus Persistence in Human Populations
Source: PLoS Med. 2008 Feb 12;5(2):e31. doi: 10.1371/journal.pmed.0050031 (PMC2235898; doi:10.1371/journal.pmed.0050031)
Supplement: Supplementary file 13 [file pmed.0050031.sg013.pdf]

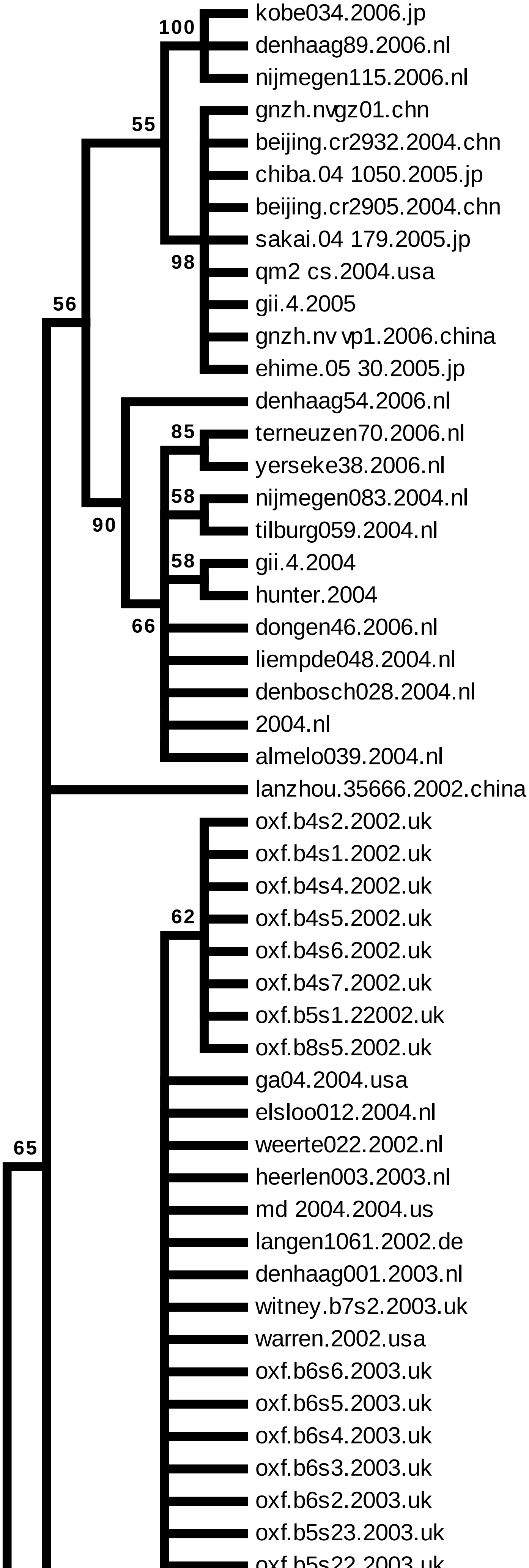
100
55
98
56
85
90
58
58
66
62
65
kobe034.2006.jp
denhaag89.2006.nl
nijmegen115.2006.nl
gnzh.nvgz01.chn
beijing.cr2932.2004.chn
chiba.04 1050.2005.jp
beijing.cr2905.2004.chn
sakai.04 179.2005.jp
qm2 cs.2004.usa
gii.4.2005
gnzh.nv vp1.2006.china
ehime.05 30.2005.jp
denhaag54.2006.nl
terneuzen70.2006.nl
yerseke38.2006.nl
nijmegen083.2004.nl
tilburg059.2004.nl
gii.4.2004
hunter.2004
dongen46.2006.nl
liempde048.2004.nl
denbosch028.2004.nl
2004.nl
almelo039.2004.nl
lanzhou.35666.2002.china
oxf.b4s2.2002.uk
oxf.b4s1.2002.uk
oxf.b4s4.2002.uk
oxf.b4s5.2002.uk
oxf.b4s6.2002.uk
oxf.b4s7.2002.uk
oxf.b5s1.22002.uk
oxf.b8s5.2002.uk
ga04.2004.usa
elsloo012.2004.nl
weerte022.2002.nl
heerlen003.2003.nl
md 2004.2004.us
langen1061.2002.de
denhaag001.2003.nl
witney.b7s2.2003.uk
warren.2002.usa
oxf.b6s6.2003.uk
oxf.b6s5.2003.uk
oxf.b6s4.2003.uk
oxf.b6s3.2003.uk
oxf.b6s2.2003.uk
oxf.b5s23.2003.uk

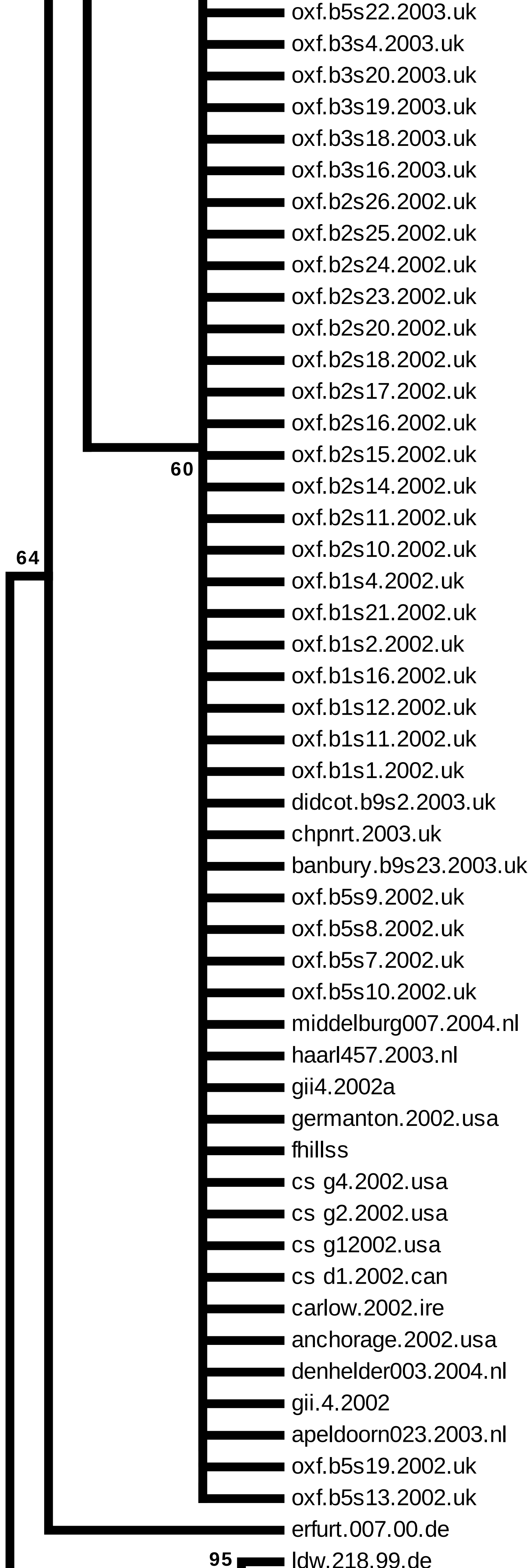
oxf.b5s22.2003.uk
oxf.b3s4.2003.uk
oxf.b3s20.2003.uk
oxf.b3s19.2003.uk
oxf.b3s18.2003.uk
oxf.b3s16.2003.uk
oxf.b2s26.2002.uk
oxf.b2s25.2002.uk
oxf.b2s24.2002.uk
oxf.b2s23.2002.uk
oxf.b2s20.2002.uk
oxf.b2s18.2002.uk
oxf.b2s17.2002.uk
oxf.b2s16.2002.uk
oxf.b2s15.2002.uk
60
oxf.b2s14.2002.uk
oxf.b2s11.2002.uk
64
oxf.b2s10.2002.uk
oxf.b1s4.2002.uk
oxf.b1s21.2002.uk
oxf.b1s2.2002.uk
oxf.b1s16.2002.uk
oxf.b1s12.2002.uk
oxf.b1s11.2002.uk
oxf.b1s1.2002.uk
didcot.b9s2.2003.uk
chpnrt.2003.uk
banbury.b9s23.2003.uk
oxf.b5s9.2002.uk
oxf.b5s8.2002.uk
oxf.b5s7.2002.uk
oxf.b5s10.2002.uk
middelburg007.2004.nl
haarl457.2003.nl
gii4.2002a
germanton.2002.usa
fhillss
cs g4.2002.usa
cs g2.2002.usa
cs g12002.usa
cs d1.2002.can
carlow.2002.ire
anchorage.2002.usa
denhelder003.2004.nl
gii.4.2002
apeldoorn023.2003.nl
oxf.b5s19.2002.uk
oxf.b5s13.2002.uk
erfurt.007.00.de
95
ldw.218.99.de

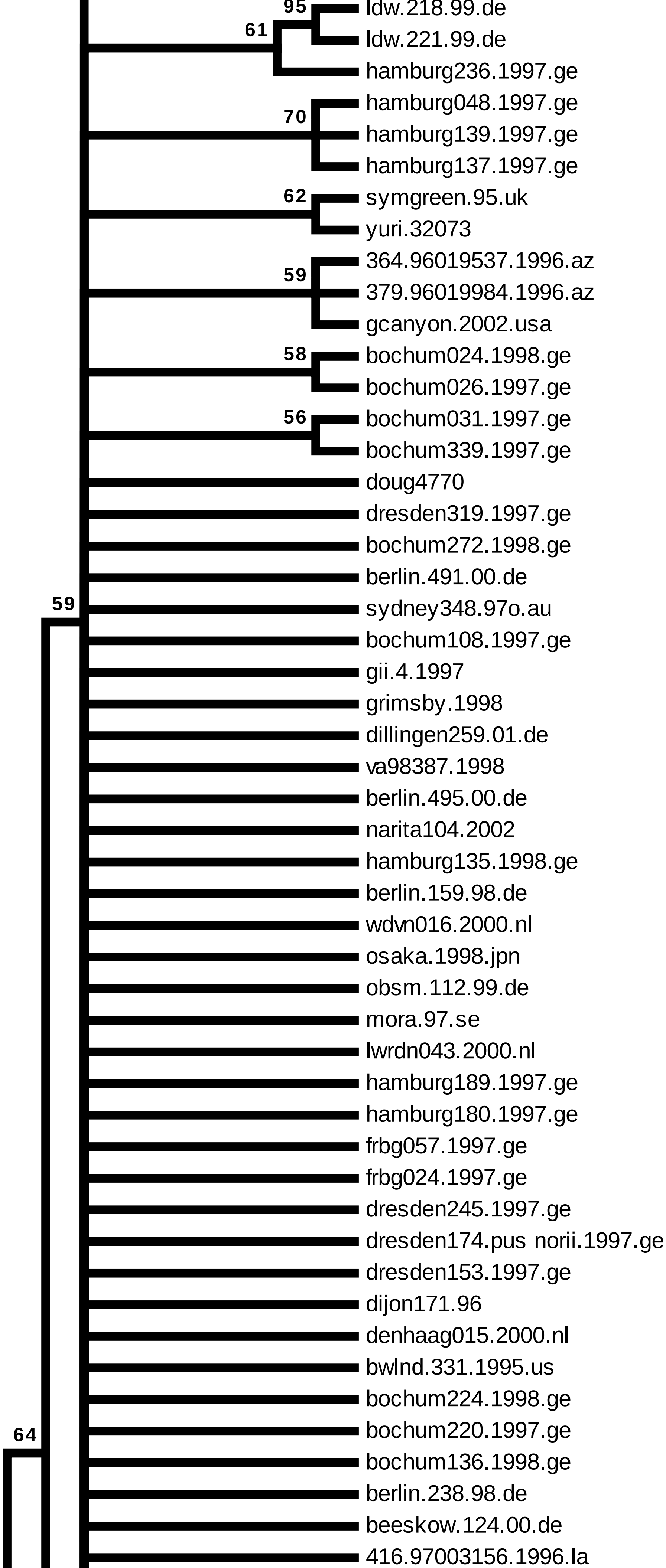
95
61
ldw.218.99.de
ldw.221.99.de
hamburg236.1997.ge
70
hamburg048.1997.ge
hamburg139.1997.ge
hamburg137.1997.ge
62
symgreen.95.uk
yuri.32073
59
364.96019537.1996.az
379.96019984.1996.az
gcanyon.2002.usa
58
bochum024.1998.ge
bochum026.1997.ge
56
bochum031.1997.ge
bochum339.1997.ge
doug4770
dresden319.1997.ge
bochum272.1998.ge
berlin.491.00.de
59
sydney348.97o.au
bochum108.1997.ge
gii.4.1997
grimsby.1998
dillingen259.01.de
va98387.1998
berlin.495.00.de
narita104.2002
hamburg135.1998.ge
berlin.159.98.de
wdvn016.2000.nl
osaka.1998.jpn
obsm.112.99.de
mora.97.se
lwrdn043.2000.nl
hamburg189.1997.ge
hamburg180.1997.ge
frbg057.1997.ge
frbg024.1997.ge
dresden245.1997.ge
dresden174.pus norii.1997.ge
dresden153.1997.ge
dijon171.96
denhaag015.2000.nl
bwlnd.331.1995.us
bochum224.1998.ge
64
bochum220.1997.ge
bochum136.1998.ge
berlin.238.98.de
beeskow.124.00.de
416.97003156.1996.la

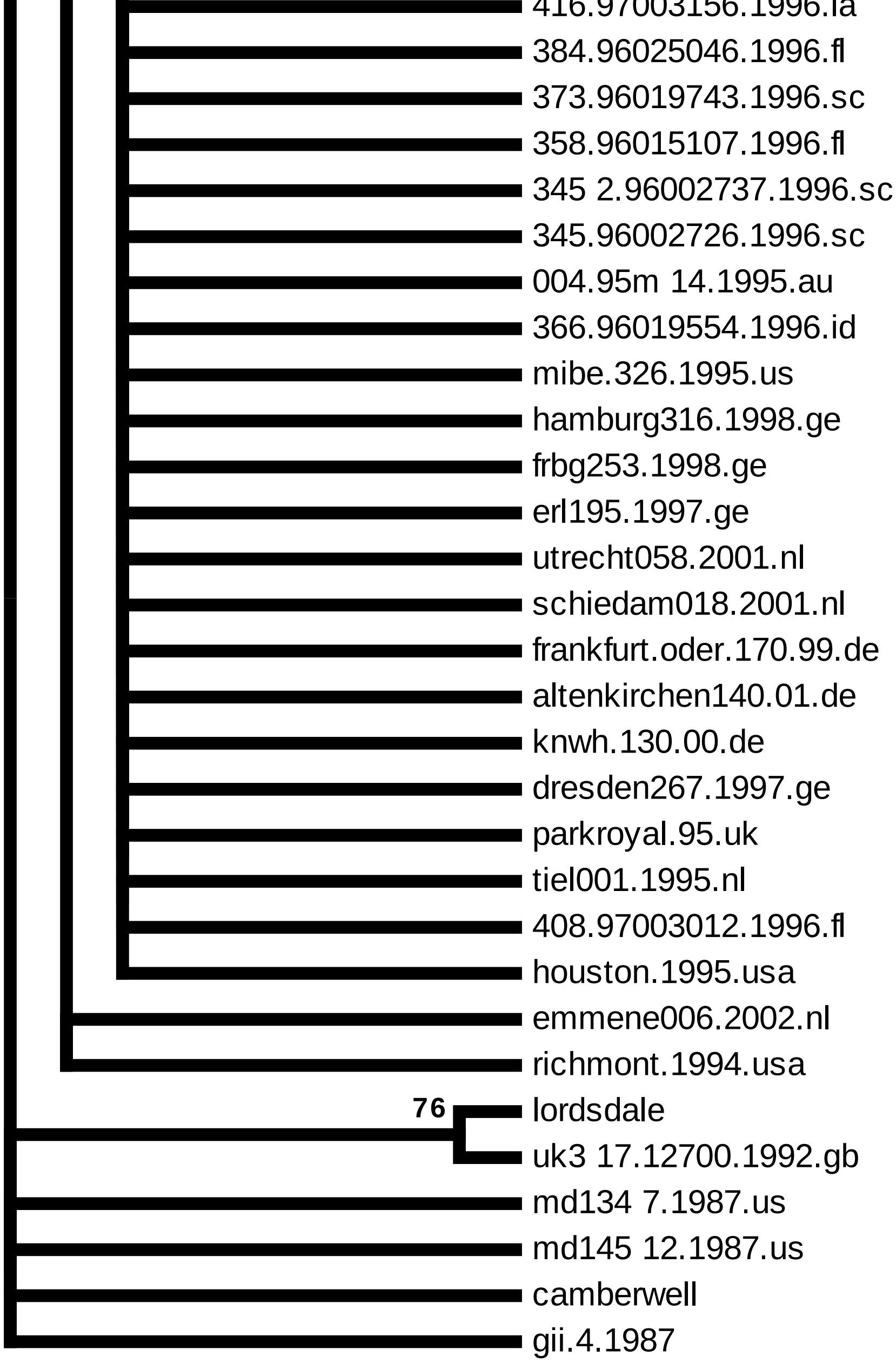
416.97003156.1996.la
384.96025046.1996.fl
373.96019743.1996.sc
358.96015107.1996.fl
345 2.96002737.1996.sc
345.96002726.1996.sc
004.95m 14.1995.au
366.96019554.1996.id
mibe.326.1995.us
hamburg316.1998.ge
frbg253.1998.ge
erl195.1997.ge
utrecht058.2001.nl
schiedam018.2001.nl
frankfurt.oder.170.99.de
altenkirchen140.01.de
knwh.130.00.de
dresden267.1997.ge
parkroyal.95.uk
tiel001.1995.nl
408.97003012.1996.fl
houston.1995.usa
emmene006.2002.nl
richmont.1994.usa
76
lordsdale
uk3 17.12700.1992.gb
md134 7.1987.us
md145 12.1987.us
camberwell
gii.4.1987
