# Supplementary material for: Mechanisms of GII.4 Norovirus Persistence in Human Populations
Source: PLoS Med. 2008 Feb 12;5(2):e31. doi: 10.1371/journal.pmed.0050031 (PMC2235898; doi:10.1371/journal.pmed.0050031)
Supplement: Supplementary file 16 [file pmed.0050031.sg016.pdf]

A

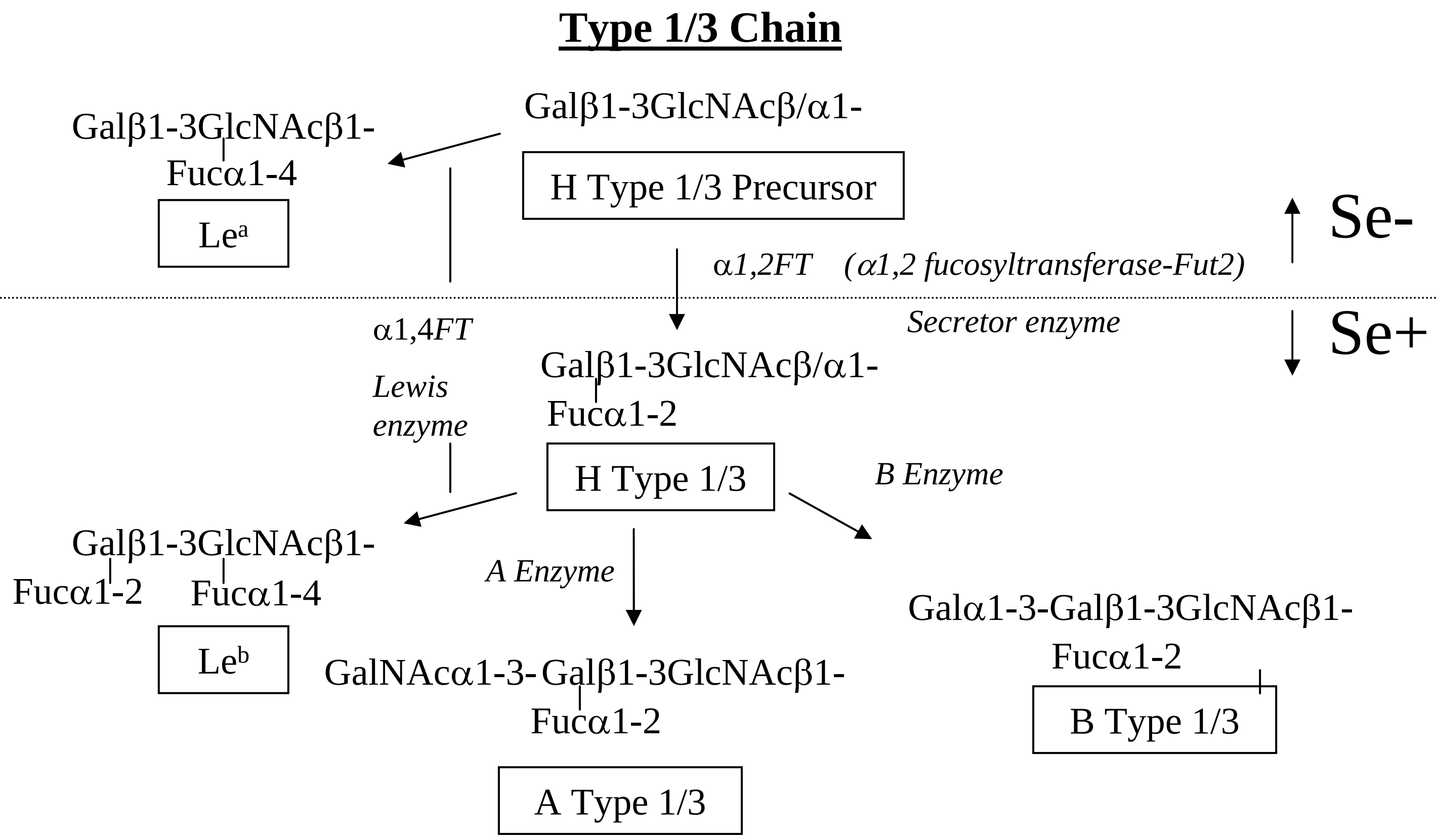

Type 1/3 Chain
Galβ1-3GlcNAcβ/α1-
H Type 1/3 Precursor
Galβ1-3GlcNAcβ1-
Fucα1-4
Le^a
α1,2FT (α1,2 fucosyltransferase-Fut2)
Secretor enzyme
Se-
Se+
α1,4FT
Lewis enzyme
Galβ1-3GlcNAcβ/α1-
Fucα1-2
H Type 1/3
B Enzyme
A Enzyme
Galβ1-3GlcNAcβ1-
Fucα1-2
Fucα1-4
Le^b
GalNAcα1-3-Galβ1-3GlcNAcβ1-
Fucα1-2
A Type 1/3
Galα1-3-Galβ1-3GlcNAcβ1-
Fucα1-2
B Type 1/3

B

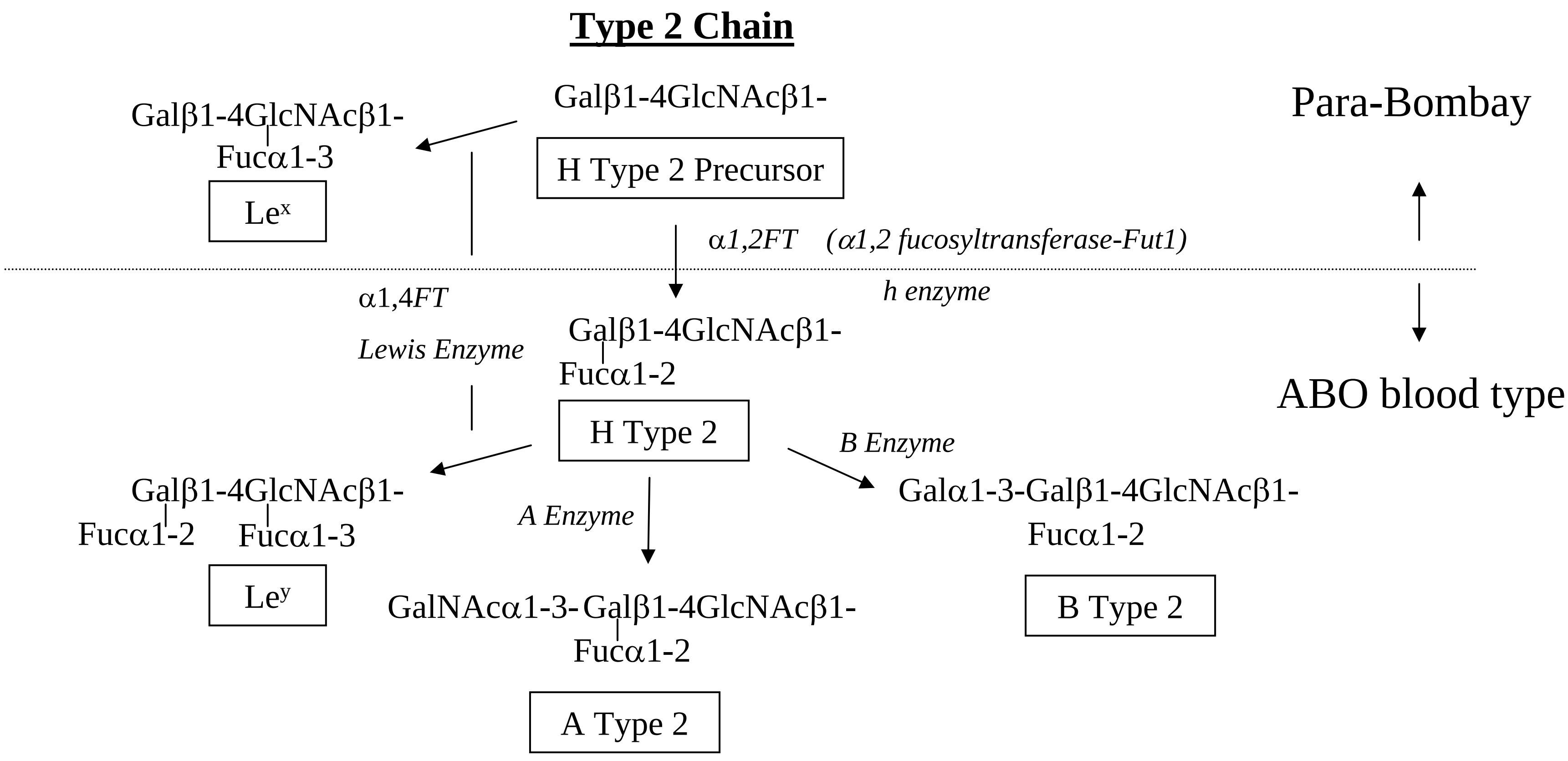
Type 2 Chain
Galβ1-4GlcNAcβ1-
Fucα1-3
Le^x
Galβ1-4GlcNAcβ1-
H Type 2 Precursor
Para-Bombay
α1,2FT (α1,2 fucosyltransferase-Fut1)
h enzyme
α1,4FT
Lewis Enzyme
Galβ1-4GlcNAcβ1-
Fucα1-2
H Type 2
ABO blood type
B Enzyme
Galβ1-4GlcNAcβ1-
Fucα1-2
Fucα1-3
Le^y
A Enzyme
Galα1-3-Galβ1-4GlcNAcβ1-
Fucα1-2
B Type 2
GalNAcα1-3-Galβ1-4GlcNAcβ1-
Fucα1-2
A Type 2
