# Supplementary material for: Mechanisms of GII.4 Norovirus Persistence in Human Populations
Source: PLoS Med. 2008 Feb 12;5(2):e31. doi: 10.1371/journal.pmed.0050031 (PMC2235898; doi:10.1371/journal.pmed.0050031)
Supplement: Supplementary file 17 [file pmed.0050031.sg017.pdf]

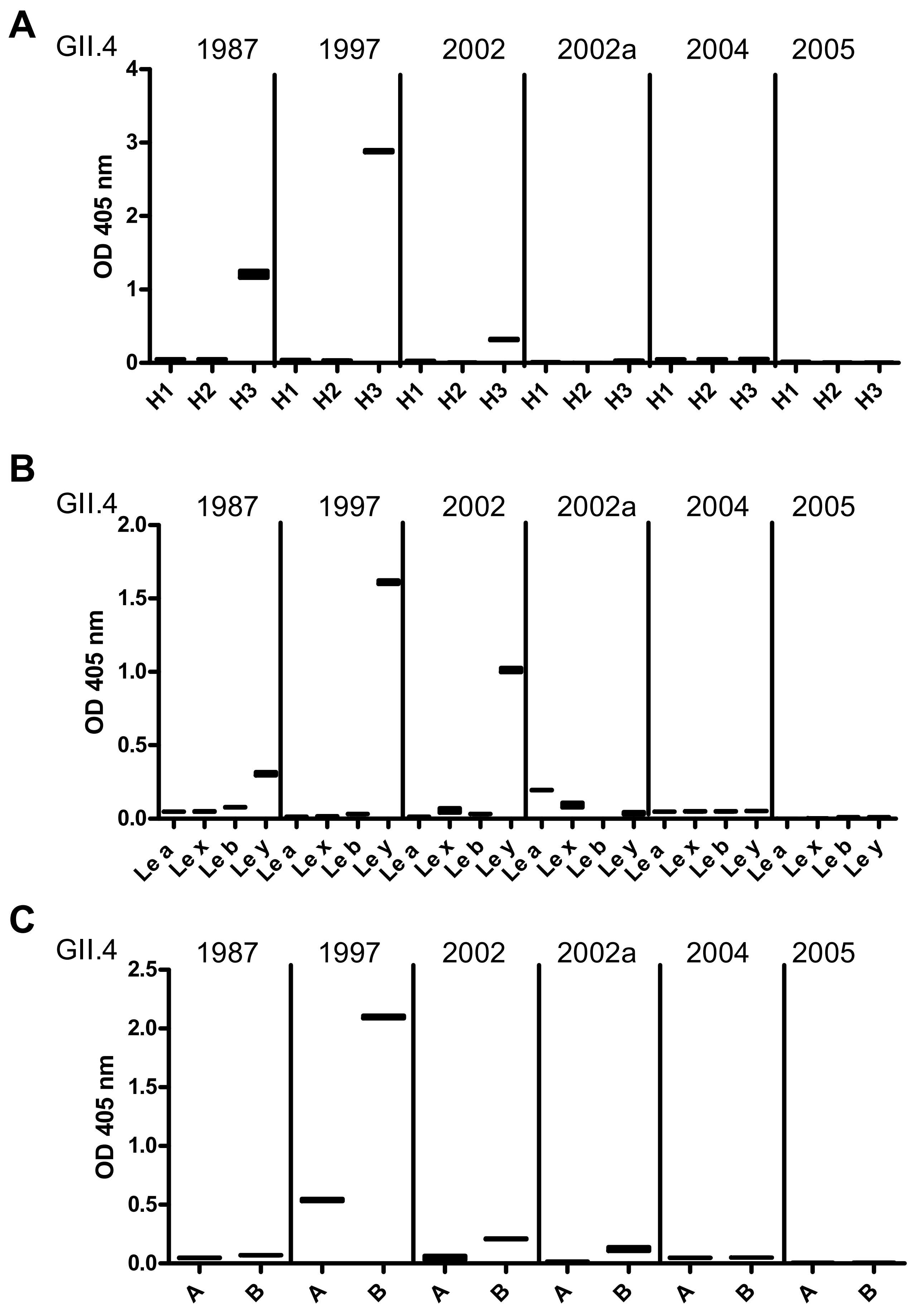
A
GII.4
1987
1997
2002
2002a
2004
2005
OD 405 nm
H1
H2
H3
B
Lea
Lex
Leb
Ley
C
A
B
