# Supplementary material for: Mechanisms of GII.4 Norovirus Persistence in Human Populations
Source: PLoS Med. 2008 Feb 12;5(2):e31. doi: 10.1371/journal.pmed.0050031 (PMC2235898; doi:10.1371/journal.pmed.0050031)
Supplement: Supplementary file 18 [file pmed.0050031.sg018.pdf]

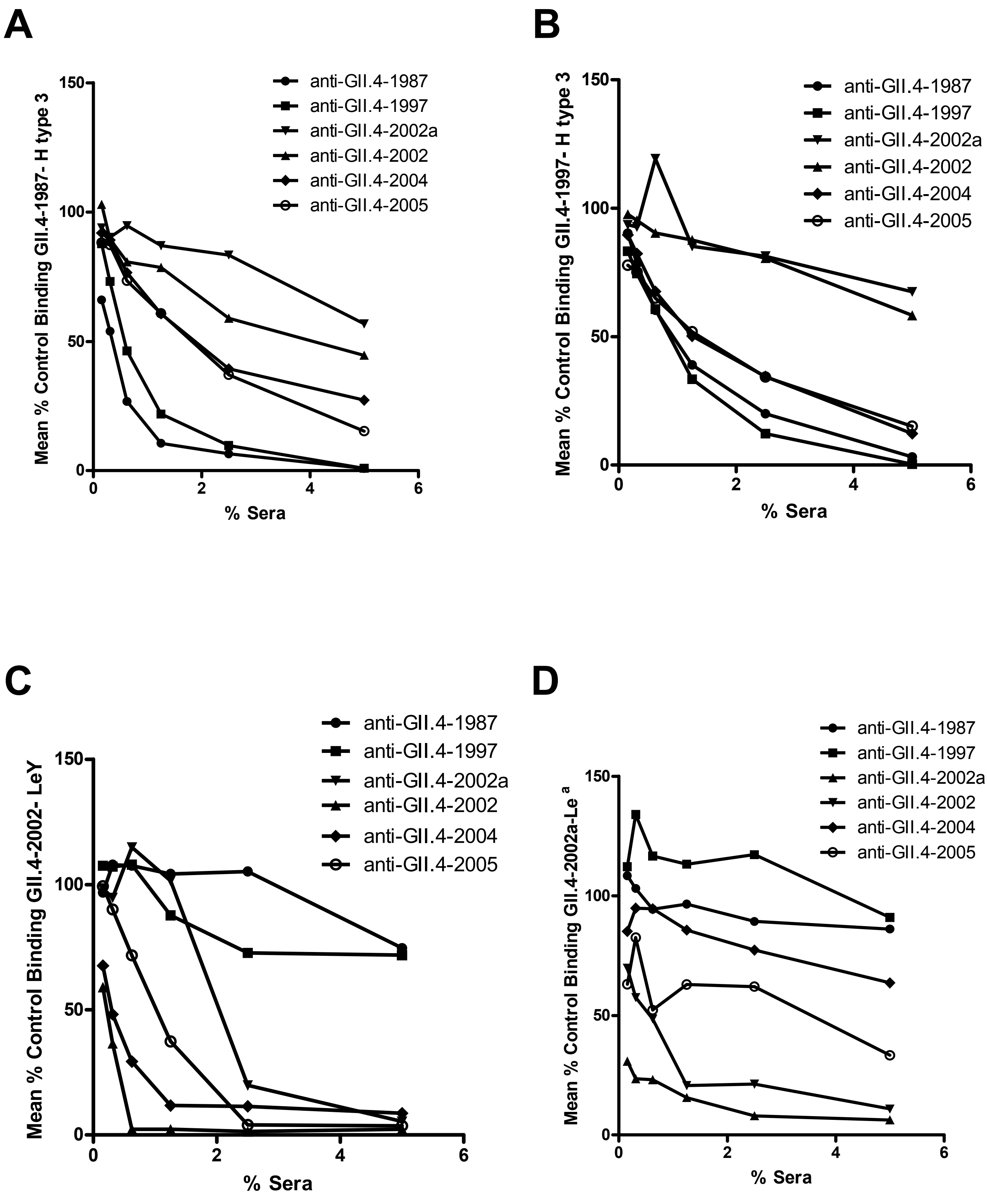
A
Mean % Control Binding GII.4-1987- H type 3
% Sera
anti-GII.4-1987
anti-GII.4-1997
anti-GII.4-2002a
anti-GII.4-2002
anti-GII.4-2004
anti-GII.4-2005
B
Mean % Control Binding GII.4-1997- H type 3
% Sera
anti-GII.4-1987
anti-GII.4-1997
anti-GII.4-2002a
anti-GII.4-2002
anti-GII.4-2004
anti-GII.4-2005
C
Mean % Control Binding GII.4-2002- LeY
% Sera
anti-GII.4-1987
anti-GII.4-1997
anti-GII.4-2002a
anti-GII.4-2002
anti-GII.4-2004
anti-GII.4-2005
D
Mean % Control Binding GII.4-2002a-Le a
% Sera
anti-GII.4-1987
anti-GII.4-1997
anti-GII.4-2002a
anti-GII.4-2002
anti-GII.4-2004
anti-GII.4-2005
